# Automated Active Space Selection with Dipole Moments

**DOI:** 10.1021/acs.jctc.2c01128

**Published:** 2023-04-11

**Authors:** Benjamin W. Kaufold, Nithin Chintala, Pratima Pandeya, Sijia S. Dong

**Affiliations:** † Department of Chemistry and Chemical Biology, Northeastern University, Boston, Massachusetts 02115, United States; ‡ Department of Physics and Department of Chemical Engineering, Northeastern University, Boston, Massachusetts 02115, United States; § The Institute for Experiential AI, Northeastern University, Boston, Massachusetts 02115, United States

## Abstract

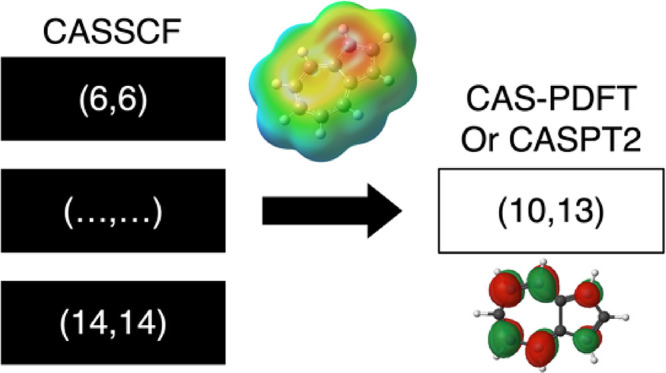

Multireference calculations can provide accurate information of systems with strong correlation, which have increasing importance in the development of new molecules and materials. However, selecting a suitable active space for multireference calculations is nontrivial, and the selection of an unsuitable active space can sometimes lead to results that are not physically meaningful. Active space selection often requires significant human input, and the selection that leads to reasonable results often goes beyond chemical intuition. In this work, we have developed and evaluated two protocols for automated selection of the active space for multireference calculations based on a simple physical observable, the dipole moment, for molecules with nonzero ground-state dipole moments. One protocol is based on the ground-state dipole moment, and the other is based on the excited-state dipole moments. To evaluate the protocols, we constructed a dataset of 1275 active spaces from 25 molecules, each with 51 active space sizes considered, and have mapped out the relationship between the active space, dipole moments, and vertical excitation energies. We have demonstrated that, within this dataset, our protocols allow one to choose among a number of accessible active spaces one that is likely to give reasonable vertical excitation energies, especially for the first three excitations, with no parameters manually decided by the user. We show that, with large active spaces removed from consideration, the accuracy is similar and the time-to-solution can be reduced by more than 10 fold. We also show that the protocols can be applied to potential energy surface scans and determining the spin states of transition metal oxides.

## 1 Introduction

Strong correlation is prevalent in many chemical systems of modern importance. For example, accurate descriptions of the electronically excited states in organic diradicals for applications in organic electronics and the spin states of transition metal complexes for catalysis require accurate treatment of strong correlation. Multireference methods are usually necessary for treating systems with strong correlation. However, commonly used multireference methods, such as complete-active-space (CAS) second-order perturbation theory (PT2),^1^ are active-space-dependent, meaning that their reference wave function is dependent on the selection of an active space, which has been a challenge for the field. Traditionally, active space selection is a complex and time-consuming procedure that relies on manual selection based on chemical intuition, but chemical intuition cannot always capture a suitable active space. Choosing to correlate the unsuitable orbitals and/or number of electrons in a multireference calculation can harm the accuracy of these methods and, at worst, make the result physically meaningless. Due to the limitations in the manual selection of the active space and the increasing need of high-throughput multireference calculations, several schemes of systematic and/or automated active space selection schemes for specific classes of systems have been proposed.^2−14^

When selecting the active space, it is important to balance the computational cost and accuracy. An active space in the context of the complete active space self-consistent field (CASSCF) method,^15−17^ the most commonly used method to generate the reference wave functions for multireference methods, is usually written as (*n*_e_, *n*_o_), where *n*_e_ is the number of electrons and *n*_o_ is the number of orbitals. This annotation describes the size of the active space, but which orbitals are included in the active space can also affect the accuracy. The most accurate configuration space is always the full space of electrons and molecular orbitals, but correlating this entire space via full configuration interaction (FCI)^18^ is only practical for very small molecules and basis sets and is not achievable for most practical calculations.^19^ For molecules on the order of five or more atoms with triple-ζ basis sets, complete active spaces with up to only 22 electrons and 22 orbitals are affordable and usually with special algorithmic improvements.^20^ Based on chemical intuition, when active spaces are chosen manually, all electrons and orbitals from π bonds and lone pairs, as well as any correlating antibonding orbitals and bonds liable to break, are suggested for inclusion;^21−25^ including all valence electrons and orbitals has also been suggested.^15,26^ Unfortunately, choosing an active space that falls within the (22, 22) boundary and follows these rules of intuition will not necessarily result in accurate calculations, and sometimes, the chemical intuition would result in a larger active space that is not computationally affordable. For example, even the first five electronic excited states for a certain molecule may involve transitions to Rydberg orbitals and require active spaces with orbitals beyond the valence,^27,75^ but there is no systematic way to choose these orbitals.

For an automated selection scheme, one could start from a less expensive level of theory, such as an unrestricted Hartree–Fock^76^ or restricted active space self-consistent field (RASSCF)^28^ calculation to determine which orbitals and how many electrons should be included in the active space of the final CASSCF calculation.^2,3^ Some other methods involve using molecular fragments and orbital localization to adapt intuitive rules to larger systems^4^ or calculating orbital entropy and entanglement to determine which individual or pairs of orbitals are most relevant to describe correlation.^5,77^ Machine learning has also been applied to this problem, in particular to determine the best active space to describe diatomic bond dissociations^6^ and for certain transition metal complexes.^9^ Finally, methods based on the occupation numbers of orbitals have been used as far back as 1988, when Pulay and Hamilton proposed including orbitals with unrestricted Hartree–Fock natural orbital occupation numbers far from 2 or 0 in the active space.^12^ The use of occupation numbers for this purpose was expanded by Khedkar and Roemelt,^13,14^ who use *n*-electron valence state second-order perturbation theory (NEVPT2)^29−32^ occupation numbers to expand the active space from an initial guess, and King and Gagliardi,^10^ who use a ranked orbital approach to build an active space that does not exceed a certain threshold in size.

In this work, we propose an alternative strategy to select the active space, which is to identify and use physical observables that can reflect the quality of the reference wave function. The use of certain physical observables allows one to have a selection criterion whose values can in principle be obtained or verified experimentally. Here, we construct and evaluate protocols that use a simple physical observable, namely, the dipole moment, to select the active space. Previously, some of the authors found that dipole moments from time-dependent density functional theory (TDDFT)^33,34^ can be used to guide the selection of the active space for the retinal Schiff base^35^ for calculating vertical excitation energies with multiconfiguration pair-density functional theory (MC-PDFT).^36,37^ MC-PDFT is a multireference method whose reference wave function can be from multiconfigurational methods such as CASSCF or density matrix renormalization group (DMRG)^38−40^ and whose dynamic correlation is calculated based on the pair-density functional theory with an on-top density functional. This previous work focused on the version of MC-PDFT that uses CASSCF reference wave functions, which is called complete active space pair-density functional theory (CAS-PDFT).^36,37^ This finding is not only useful but logical as the PDFT calculation in MC-PDFT depends on electron densities computed from the reference wave function, and the dipole moment is the first moment of the electron density. An accurate dipole moment suggests an accurate electron density and potentially an accurate wave function.

In the current work, we use the finding discussed above^35^ as the basis for our hypothesis that an active space that describes the dipole moment well for any molecule, at the CASSCF level of theory, should give reasonably accurate excitation energies with CAS-PDFT and CASPT2. From this hypothesis, we design two protocols using the accuracy of the dipole moment as a guide to select one active space from two or more possible choices, such as a set of small active spaces with various sizes. This removes the need of manual selection of orbitals while allowing for a suitable active space not unnecessarily large to be chosen, and it is applicable, in principle, to any system with a nonzero dipole moment. It also provides a certain level of guarantee that the active space selected is reasonable, which addresses the issue that one may not know whether the results of their active-space-dependent multireference calculation are physically meaningful when experimental excitation energy data are unavailable. Although this method does require one to carry out multiple CASSCF calculations, these calculations can be done fully in parallel and the time-to-solution of using this method can be much lower than using the largest active space possible. It is particularly suitable for cases where chemical intuition cannot result in reasonable active spaces. We focus on the performance of our protocols for CAS-PDFT and CASPT2 in this work, but in principle, the protocols can be directly applied to other active-space-dependent multireference methods to select the active space.

## 2 Methods

### 2.1 Molecular Dataset

**Figure 1:**
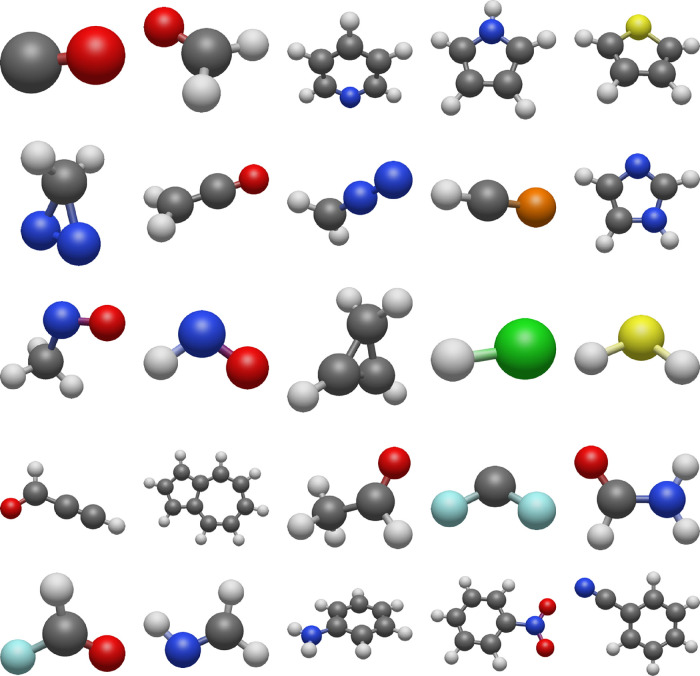
Molecules in NAQ, with M06-2X/ANO-RCC-VTZP-optimized geometries. Gray: carbon, blue: nitrogen, red: oxygen, yellow: sulfur, bright green: chlorine, yellowish green: fluorine, orange: phosphorus, white: hydrogen. Coordinates of all molecules are presented in the SI Section “Cartesian Coordinates”. Structures are visualized with Avogadro2.^78^

To evaluate our active space selection protocols, we constructed a dataset of 25 molecules, as shown in Figure 1. To ensure consistency and to allow the systematic testing of multiple molecules and active spaces, we impose the following constraints in the selection of the molecules:

- The experimental ground-state dipole moment can be found in the NIST database.^41^
- At least one reference excitation energy is available in the QUEST database (QUESTDB).^27,42,43^
- The ground state is singlet.
- The molecule is charge neutral.
- Total ground-state dipole moment must be nonzero.

The final dataset consists of those molecules conforming to the above rules, where complete data for dipole moments and excitation energies up to S_5_ (if the reference values are available) were obtained. For convenience, we also refer to this dataset as the NAQ (NIST and QUEST) set.

The protocols that we test here are, in principle, applicable to molecules outside of these constraints. However, species with other multiplicities require a different set of active spaces to test, while charged molecules require one to establish a center of charge consistent with experiment, or between multiple software packages. The constraints were imposed to ease the mass-testing of active spaces and establish the potential usefulness of the protocols with automated tools. Finally, we developed the protocols for molecules that have nonzero ground-state dipole moments.

Prior to any multireference calculation, a ground-state geometry optimization of each molecule was performed using Kohn–Sham density functional theory (KS-DFT)^79^ at the M06-2X/ANO-RCC-VTZP level of theory with Gaussian 16.^44^ We perform these optimizations instead of using QUESTDB’s provided geometries to ensure that the protocols can be extended to systems outside the dataset, where the ground-state geometry may be experimental or predicted with a different computational method. M06-2X^45^ was selected for its well-rounded high performance for both ground-state and excited-state properties,^42,45^ and it is one of a set of density functionals that give more accurate excited-state dipole moments, given that excited-state dipole moments are always more inaccurate than ground-state ones.^46^ The ANO-RCC-VTZP^47^ basis set was also used for its high accuracy in ground- and excited-state calculations. For calculations in Gaussian, ANO-RCC-VTZP was obtained through the Basis Set Exchange.^48^ We confirmed that molecules were optimized to a local minimum by frequency analysis, except for aniline, which was minimized with the constraint that *C*_2*v*_ symmetry must be maintained (a first-order transition state) for ease of comparison with QUESTDB, which also uses this symmetry. For all minimizations, the maximum symmetry was detected and preserved so that the symmetry constraints are the same as those used in QUESTDB.

Multireference calculations were carried out using OpenMolcas 22.02.^49^ The second-order Douglas–Kroll–Hess Hamiltonian^50−52^ was used. The initial orbitals of CASSCF calculations were generated using KS-DFT at the level of M06-2X/ANO-RCC-VTZP. An ultrafine two-electron integral grid was used. An example input file is provided in the Supporting Information (SI) Section “Example OpenMolcas Input File”.

For each molecule, we carried out state-averaged (SA)-CASSCF calculations over six states on an array of active spaces given in Table 1. We refer to this set of active spaces as the PASS+ (parallel active space scan plus) set. Furthermore, we define a set of active spaces as PASS (parallel active space scan) where active spaces with only four electrons or two virtual orbitals are removed, as shown in Table 1. In Section 3.1, we discuss our motivation for constructing PASS.

**Table 1:**
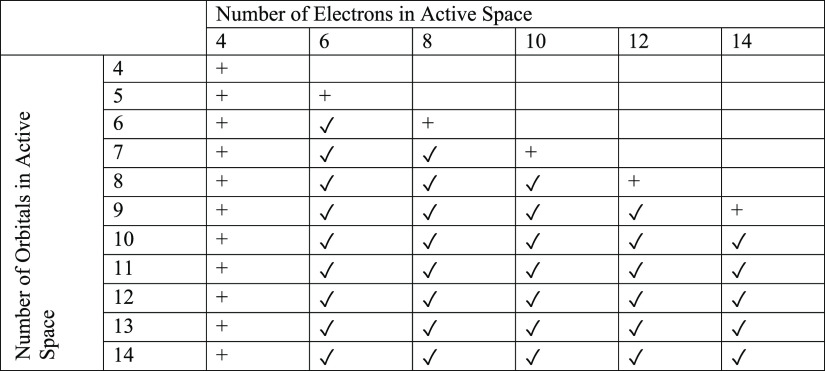
Active Spaces Tested in Our Multireference Calculations on Molecules from QUESTDB^a^

a Active spaces marked with a check symbol are in PASS and PASS+. Active spaces marked with a plus symbol are in PASS+ but not PASS.

Dynamic correlation is computed in CAS-PDFT and CASPT2 based on CASSCF reference wave functions. For CAS-PDFT, the translated functional of choice was tPBE.^36,53^ For CASPT2 calculations, an IPEA shift of 0.25 a.u. was used.^54,55^

### 2.2 Automated Active Space Selection Protocols

Computing dipole moments is central to the evaluation of both of our protocols. Here, we use the total dipole moment for each electronic state, which is given by , where each of ⟨μ_*n*_⟩ is the expectation value of the dipole moment along each Cartesian axis for the electronic state of interest. We have also tested the use of the dipole moment as a vector, μ⃗ = [μ_*x*_ μ_*y*_ μ_*z*_].

**Figure 2:**
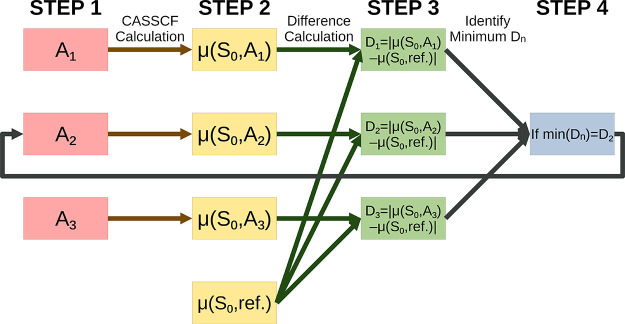
Flowchart representation of the GDM-AS protocol.

**Figure 3:**
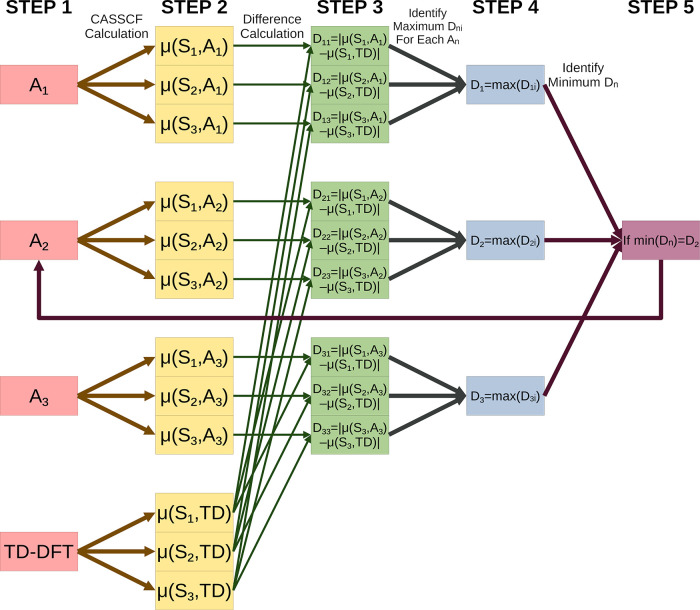
Flowchart representation of the EDM-AS protocol.

We construct two protocols for automated active space selection based on dipole moments. We name them “ground-state dipole moment” active-space selection (GDM-AS) and “excited-state dipole moment” active-space selection (EDM-AS). In each case, the minimum error in dipole moment was used as a criterion for selection. The workflows of GDM-AS, EDM-AS, and their variations that consider the directions of dipole moments, vGDM-AS and vEDM-AS, are described in detail below. Flowchart schematics of GDM-AS and EDM-AS are provided in Figures 2 and 3, respectively.

#### 2.2.1 GDM-AS

To select an active space according to GDM-AS, the following steps are taken:

(1) Identify a set of candidate active spaces, here labeled A*_n_*. This need not be all of the active spaces that we have discussed in this paper.
(2) Conduct a CASSCF calculation, which includes computing the dipole moments for the ground state, S_0_, on each of A_*n*_. We label the computed dipole moments μ(S_0_,A_*n*_). Determine the reference ground-state dipole moment values to be used as a point of comparison. We label the reference dipole moments μ(S_0_,ref.). In this work, we have tested the use of experimental dipole moment values as well as those computed using DFT with a variety of density functionals.
(3) Take the absolute difference in the dipole moment values between the CASSCF-calculated ones for each active space and the reference values. We define them as *D_n_* = |μ(S_0_,A_*n*_) – μ(S_0_,ref.)|.
(4) Identify the active space that gives the smallest *D_n_*. If *D_m_* = min(*D_n_*), then A_*m*_ is the active space chosen by GDM-AS.

In GDM-AS, the ground state (S_0_) dipole moment is calculated with CASSCF at each active space one might want to test and is compared to a reference dipole moment value—a known accurate ground-state dipole moment either from experimental measurements or from simulations. In this work, we test using experimental dipole moments provided by the NIST database, as well as calculated dipole moments with a series of density functionals, those being CAM-B3LYP,^56^ ωB97-xD,^57^ PBE0,^53,58,59^ HSE06,^60−66^ LC-ωHPBE,^67,68^ M06-HF,^59^ and M06-2X itself. The active space selected is the one that has the lowest absolute error between the CASSCF-calculated and the reference dipole moments.

#### 2.2.2 vGDM-AS

The procedure for vGDM-AS is the same as that of GDM-AS, but we compute the dipole moment error *D_n_* according to .

#### 2.2.3 EDM-AS

To select an active space according to EDM-AS, the following steps are taken:

(1) Identify a set of candidate active spaces, here labeled A_*n*_. This need not be all of the active spaces that we have discussed in this paper.
(2) Conduct a CASSCF calculation, which includes computing the dipole moments for each state, S_*i*_, on each of A_*n*_. We label the computed dipole moments μ(S_*i*_,A_*n*_). Compute the dipole moment values for each excited state up to the excited state of interest with TDDFT. We label them as μ(S_*i*_,TD). We have tested the use of reference excited-state dipole moments provided by TDDFT with a variety of density functionals, but the user only needs to choose one density functional.
(3) For each active space A_*n*_ and excited state S_*i*_, take the absolute difference in the dipole moment calculated by CASSCF and that calculated by TDDFT. Here, these differences are labeled *D_n_*_*i*_. We define *D_n_*_*i*_ = |μ(S_*i*_,A_*n*_) – μ(S_*i*_,TD)|.
(4) For each active space A_*n*_, record the largest dipole moment difference *D_n_*_*i*_ for any of the excited states. We define *D_n_* = max(*D_n_*_*i*_).
(5) Identify the active space that gives the smallest *D_n_*. If *D_m_* = min(*D_n_*), then A_*m*_ is the active space chosen by EDM-AS.

As shown in the algorithm, EDM-AS selects the active space with the “minimum maximum” dipole moment error. In other words, the largest dipole moment error among all excited states of the selected active space is smaller than the largest dipole moment error of every other active space. We have included a movie in the SI to demonstrate how an excited state error vs dipole moment error plot would change during this process until an active space is selected. In the TDDFT calculations of excited-state dipole moments in this work, we test the density functionals also tested for GDM-AS.

#### 2.2.4 vEDM-AS

The procedure for vEDM-AS is the same as that of EDM-AS, but we compute the dipole moment error *D*_*ni*_ according to .

#### 2.2.5 Multireference Calculations with the Selected Active Space

Each protocol above would select one active space among a set of active spaces of interest. Then, the reference wave function from the CASSCF calculation with only the selected active space can be used in any subsequent multireference calculations that account for dynamic correlation.

In this work, for benchmarking purposes, CAS-PDFT (and CASPT2) vertical excitation energies were calculated at all available active spaces described in Section 2.1 above. These excitation energies were used to map out the relationship between dipole moment errors and excitation energy errors and to determine how well the active spaces selected by the protocol performed relative to others. Sometimes CASSCF and CAS-PDFT or CASPT2 give different orderings of the electronic states. We have ordered the excitation energies based on CAS-PDFT or CASPT2 energetics, depending on whether we are testing the protocols for CAS-PDFT or for CASPT2.

### 2.3 Comparison with Intuitive or Large Active Spaces

It was necessary to determine the performance of the active spaces chosen by the protocols discussed in Section 2.2 over active spaces that a researcher may choose without them. Among the 25 molecules in our dataset, for 18 molecules, there existed an active space that could be chosen by chemical intuition (detailed below) that is also within our set of tested active spaces. To compare between the active space chosen by GDM-AS, the active space chosen by EDM-AS, the (14,14) active space, and the active space chosen by chemical intuition, for each method, the excitation energy errors for each excited state were averaged over all 18 molecules. Four average excitation energies were calculated, using the following:

- The active space chosen by GDM-AS for each molecule
- The active space chosen by EDM-AS for each molecule
- (14, 14), the largest active space tested, for each molecule
- The active space chosen by chemical intuition for each molecule

Note that in sections that do not focus on the comparison between GDM-AS or EDM-AS and the intuition active space, all 25 molecules are used in the analysis; not every molecule has data for every excited state because the reference data for some states are not available.

The chemically intuitive active space for each molecule was chosen according to the following procedure, which attempts to account for all rules typically used to decide active spaces discussed prior and accounts for the importance of Rydberg orbitals:

(1) Count the number of electrons and orbitals in the full valence and then double the number of orbitals (full valence + one Rydberg orbital per valence orbital to accommodate more excitations)
(2) If the resulting active space is not in PASS, remove the Rydberg orbitals (full valence only)
(3) If the resulting active space is still not in PASS, count all σ and π bonds not involving hydrogen, adding two electrons and two orbitals each, and then add two electrons and one orbital per lone pair (most useful for strained compounds as they are likely to have labile σ bonds)
(4) If the resulting active space is still not in PASS, only add two electrons and orbitals per π bond and two electrons and one orbital per lone pair
(5) If the resulting active space is still not in PASS, do not include this molecule in the performance test that compares GDM-AS and EDM-AS with intuitive active space selection

Molecules with an intuitive active space in PASS are denoted by the set name “INAQ” (intuitive NAQ) when applicable. The complete list of molecules in INAQ is given in the SI Table S1.

These steps are ordered such that the largest valid intuitive active space smaller than or equal to (14,14) would be chosen. No largest intuitive active space is smaller than (8,7). Note that this was only used to decide the size of the intuitive active space, and the aforementioned orbitals are not enforced to be included in the active space.

## 3 Results and Discussion

To evaluate the performance of each of GDM-AS and EDM-AS in its ability to select the active space that can give reasonable excitation energies, we applied each one of them to our dataset of molecules. We focus our analysis on the use of experimental reference dipole moments in GDM-AS and excited-state dipole moments calculated with TDDFT with the M06-2X functional in EDM-AS; however, we have tested the use of reference dipole moments from other density functionals to show the flexibility of the protocol and the results are discussed in Sections 3.2 and 3.3. Unless otherwise specified, all dipole moment errors are absolute differences between CASSCF and NIST (for S_0_) or CASSCF and TD-M06-2X (for S_1_ and up) dipole moments, and all excitation energy errors are absolute differences between CAS-PDFT (or CASPT2) and QUESTDB excitation energies. A complete discussion on the methods used to find the highly accurate reference excitation energies in QUESTDB and a list of excitation energies used as reference is provided in the SI Section “QUESTDB Reference Excitation Energies” and Table S2.

### 3.1 Range of Active Spaces

**Figure 4:**
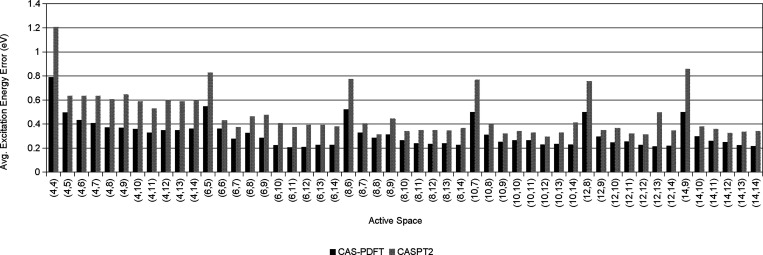
Average excitation energy error, per active space, for all excitations in our dataset (PASS+) as calculated using CAS-PDFT and CASPT2.

We have computed the average excitation energy error for all active spaces in PASS+, and the results are shown in Figure 4. We have made two observations based on Figure 4. First, we find that for all active spaces, excitation energy errors do not monotonically decrease when more electrons or more orbitals are added. To better understand the relationship between excitation energy errors and the active space size, we plotted the average excitation energy errors vs the number of determinants in each active space in Figure S1 in the SI. This shows that, as the number of determinants increases, the excitation energy errors roughly first decrease and then plateau as the number of determinants increases beyond 10,000, and it is not monotonic if every active space in the set is considered. This bolsters the importance of developing a systematic method to select the active space as simply choosing the largest affordable active space does not guarantee the best results and smaller active spaces may give similar or better results while requiring much less computing time. Second, the average excitation energy errors for some active spaces (*n*_e_*,n*_o_) in PASS+, when *n*_e_ = 4 and *n*_o_ is any allowed value in PASS+, denoted (4,*m*), or when *n*_e_ is even and *n*_o_ = *n*_e_/2 + 2, denoted (2*n*,*n* + 2), are notably greater than those for all other active spaces. This suggests that, to reduce the number of active spaces to screen in applying our protocols, it may be cost-effective if these active spaces are not considered. Therefore, we construct PASS by removing (4,*m*) and (2*n*,*n* + 2) from PASS+.

**Figure 5:**
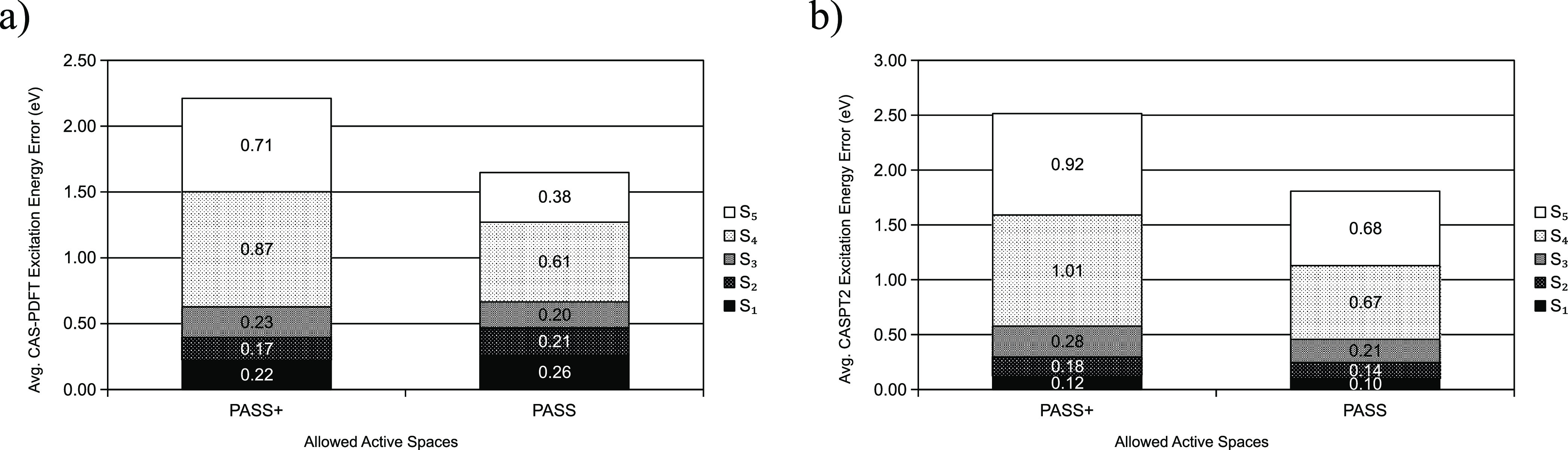
Average excitation energy error for all molecules in our dataset, with active spaces chosen by GDM-AS, when PASS is allowed vs when PASS+ is allowed. (a) gives CAS-PDFT excitation energy errors, while (b) gives CASPT2 excitation energy errors.

To evaluate the performance of PASS compared to PASS+, we apply GDM-AS using reference dipole moments from NIST to all molecules in the dataset (i.e., the NAQ set). We find that the overall performance of the protocol is about the same for PASS and PASS+, within the first three excitations, as illustrated in Figure 5. Therefore, we focus on using PASS in our analysis in this work.

### 3.2 Evaluation of GDM-AS and vGDM-AS

**Figure 6:**
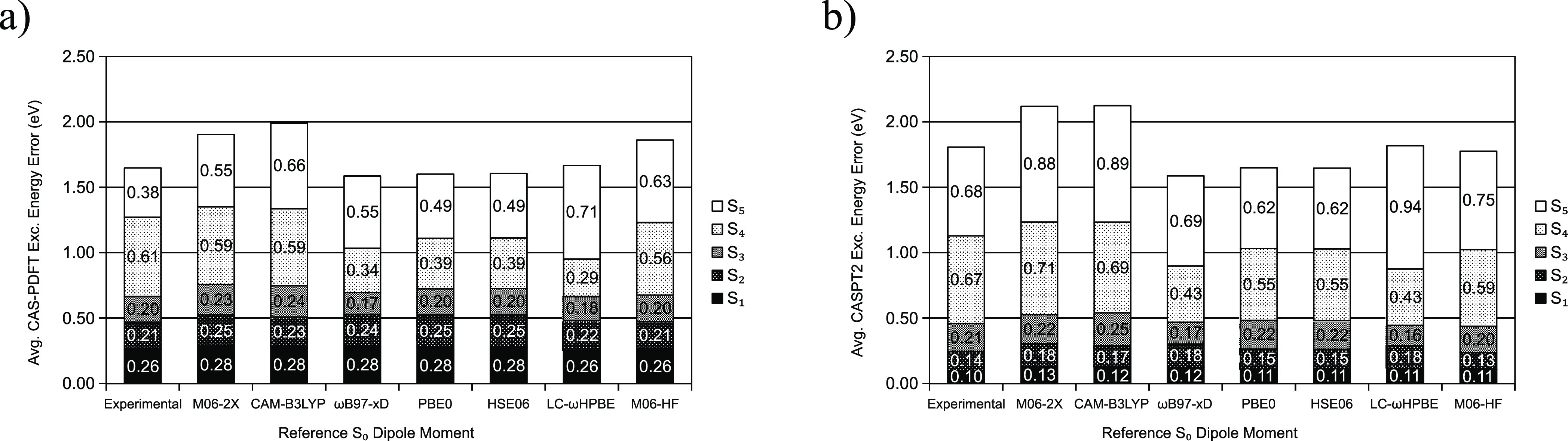
Average (a) CAS-PDFT and (b) CASPT2 excitation energy errors for active spaces chosen by GDM-AS using experimental reference dipole moments as well as those provided by a series of density functionals. The ANO-RCC-VTZP basis set is always used.

To establish the usefulness of GDM-AS as described here, we evaluate the excitation energy errors for all excitations available for molecules in the NAQ set calculated with the active space chosen by GDM-AS for each molecule. We see in Figure 6 that the average excitation energy error for the first three excitations, whether CAS-PDFT or CASPT2 excitation energies are sought, is “satisfactory” (within 0.3 eV). This holds true when experimental dipole moments are used to provide the reference values and also when any of the density functionals tested are used instead. Although using ωB97-xD to provide reference S_0_ dipole moments to GDM-AS gives the best overall performance, we also emphasize that all reference S_0_ dipole moments considered in this work allow GDM-AS to perform well (within the first three excitations).

It can be seen in the SI Table S3 that individual excitation energy errors for active spaces chosen by GDM-AS using experimental dipole moments tend to fall below or near 0.3 eV, and the first three average excitation energy errors across all molecules is always below 0.3 eV. Excitation energy errors far above this threshold tend to be isolated incidents, i.e., molecules with some highly erroneous excitation energy for a certain state have more accurate excitation energies for other states. Difluorocarbene stands out as having large excitation energy error even for S_1_, but this is known to be an inherently challenging case for calculating CAS-PDFT excitation energies, as demonstrated in the previous literature.^69^ These results suggest that the ground-state dipole moment is a good indication of the quality of the wave function for the first four electronic states (S_0_–S_3_) but not higher-lying excited states.

GDM-AS tends to find an active space in PASS that gives useful excitation energies, regardless of the overall correlation between dipole moment error and excitation energy error for individual molecules. The plots of excitation energy error with respect to dipole moment error for all molecules in the dataset are provided in Figures S2–S51 in the SI; the active space chosen by GDM-AS with experimental reference dipole moments is labeled with a green cross. Only a few cases, like hydrogen chloride (CAS-PDFT excitation energies) and aniline (both CAS-PDFT and CASPT2 excitation energies) show a clear positive correlation between the dipole moment error and excitation energy error. However, there exist cases like hydrogen cyaphide and acetaldehyde (CAS-PDFT excitation energies) or nitrosyl hydride and cyclopropene (CASPT2 excitation energies) where there is no obvious correlation between errors in dipole moment and excitation energy error, but the active space chosen still performs well relative to others.

In addition to the numerical values of the excitation energy, the active spaces selected by GDM-AS are capable of giving excitations that have characters matching those in QUESTDB. Examples are shown in Tables S4 and S5 in the SI. They are analyses of the first three excitations of nitrosyl hydride and the first for methanimine from using active spaces identified by GDM-AS.

vGDM-AS achieves similar performance to GDM-AS and is a valid protocol to use. One does need to ensure that the orientation of the molecule remains the same when the CASSCF-computed dipole moments are compared with the reference. A schematic of this procedure is given in Figure S52 in the SI. Results are given in Figures S53 and S54, and test molecules with different active spaces chosen in GDM-AS and vGDM-AS are given in Table S6.

### 3.3 Evaluation of EDM-AS and vEDM-AS

**Figure 7:**
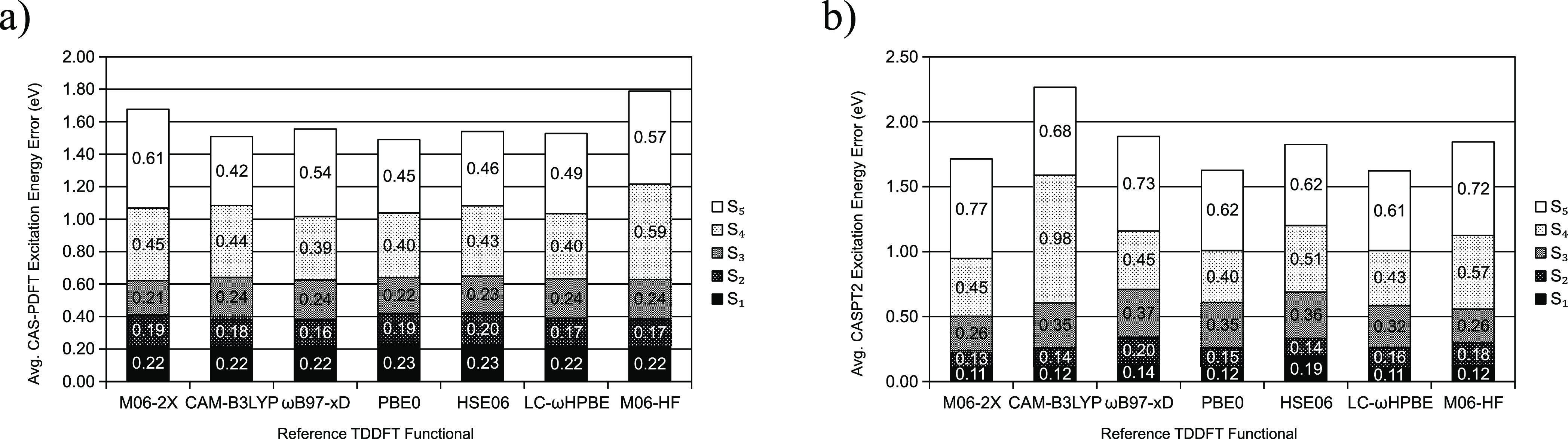
Average (a) CAS-PDFT and (b) CASPT2 excitation energy errors for active spaces chosen by EDM-AS using reference dipole moments provided by a series of density functionals. The ANO-RCC-VTZP basis set is always used.

Observations for EDM-AS are largely the same as those for GDM-AS. When any density functional is used to provide reference dipole moments (in this case, excited-state dipole moments as opposed to ground-state ones), the average CAS-PDFT excitation energy error for the first three excitations is satisfactory for our entire dataset. Average CAS-PDFT and CASPT2 excitation energy errors for the entire dataset are provided in Figure 7, and individual excitation energy errors are provided in the SI Table S7. The primary difference with respect to observations for GDM-AS is that the average CASPT2 excitation energy error is generally larger for active spaces selected by using any functional tested in this work and is less than 0.3 eV only for M06-2X and M06-HF. Therefore, we recommend M06-2X of M06-HF for EDM-AS. In any case, the protocol is adaptable to a variety of density functionals when finding the first three CAS-PDFT excitation energies or the first two CASPT2 excitation energies.

The plots of dipole moment error with respect to excitation energy error for the entire dataset are provided in the SI Figures S55–S104. High dipole moment errors were typically seen more often with higher excitation energies, even when the absolute error of the excitation energy is low. However, removing the dipole moment errors for the S_4_ and S_5_ from consideration in EDM-AS would give active spaces that have higher excitation energy errors than the original EDM-AS protocol, as seen in Figures S105 and S106 in the SI. This suggests that there may be a systematic error in the dipole moment difference between CASSCF (with active space from EDM-AS) and TDDFT. Because this is a systematic error, it does not adversely affect the performance of EDM-AS.

Since there are concerns on TDDFT’s ability to model double excitations, we test EDM-AS’s performance on choosing active spaces that can describe double excitations. The quality of the active space chosen is unlikely to be negatively affected as EDM-AS uses dipole moment information from all excitations to find the active space best suited to describe the system and would only be more likely to be problematic in the rare case that the majority of low-lying excitations are double excitations. We have found that for the only two systems in our dataset with low-lying double excitations, namely, nitrosyl hydride and nitrosomethane, EDM-AS gives reasonable active spaces whether or not the states involved in the double excitation is considered in computing dipole moment errors, as shown in Table S8 of the SI. Note that the performance of GDM-AS is completely unaffected by the ability of TDDFT to model double excitations since only ground-state dipole moment information is used as the input.

vEDM-AS performs similar to EDM-AS but does improve CASPT2 S_3_ excitation energies when ωB97-xD and LC-ωHPBE are used to provide reference dipole moments. Average excitation energy errors for active spaces chosen by vEDM-AS when different density functionals are used are given in Figures S107 and S108 of the SI, and the molecules with different active spaces chosen between EDM-AS and vEDM-AS are given in Table S9 of the SI.

### 3.4 Comparison between GDM-AS and EDM-AS

GDM-AS recommends, on average, 10.08 electrons and 11.44 orbitals when experimental dipole moments are used as reference for our dataset of molecules that on average have 20.88 valence electrons. EDM-AS recommends, on average, 10.72 electrons and 11.20 orbitals when TD-M06-2X dipole moments are used as the reference. Active spaces chosen by EDM-AS tend to include more or the same number of orbitals of Rydberg-type than the active space chosen by GDM-AS, regardless of the number of virtual orbitals in the active space recommended by each protocol. The specific orbitals involved in the active spaces is highly individual. As demonstrated in Figures S109 and S110 of the SI, two active spaces that ostensibly differ by only a single orbital have more subtle differences. Orbital shapes can change significantly, and removing an orbital from an active space does not necessarily mean that the intuitively high-energy Rydberg orbitals will be deleted. Valence orbitals may be removed instead. This only affirms the importance of using an automated selection scheme guided by means beyond the chemical intuition to choose a suitable active space.

### 3.5 Comparison with the (14,14) Active Space

When comparing the active space selected by GDM-AS and EDM-AS to the largest active space that we have evaluated, (14,14), excitation energy errors change minimally, but the speed improves significantly due to their use of smaller active spaces. A (14,14) active space corresponds to about 1.18 × 10^7^ Slater determinants, while a (10,12) active space corresponds to only 6.27 × 10^5^ Slater determinants, 1/19 of those for (14,14). The median of the number of Slater determinants corresponding to the active space from GDM-AS is only 213,444, and the median of the number of Slater determinants corresponding to the active space from EDM-AS is only 81,796. This means that GDM-AS and EDM-AS have a much shorter time-to-solution and are particularly useful in scenarios where a large number of multireference calculations need to be done for the same types of molecules, such as in the case of nonadiabatic molecular dynamics simulations, or when (14,14) is not affordable.

In our experience of organic molecules with tens of atoms, (14,14) is the largest practical active space affordable for multireference calculations. With molecules up to 10 nonhydrogen atoms in size, CASSCF calculations with the (14,14) alone approach 10 h of wall time, and CASPT2 or CAS-PDFT calculations will add up to 4 h or half an hour, respectively. Reducing the number of electrons and orbitals in the active space reduces the number of determinants needed to represent it factorially,^70^ resulting in sharp reductions in computational time. Although our protocols require multiconfigurational calculations (e.g., CASSCF) on more than one active space, these calculations can be done simultaneously with overall shorter computing time (capped by the largest active space considered) and relatively low memory requirement, while a single (14,14) CASSCF calculation would require computing nodes that meet specific memory requirements and would need longer computing time than any of the smaller active space.

**Figure 8:**
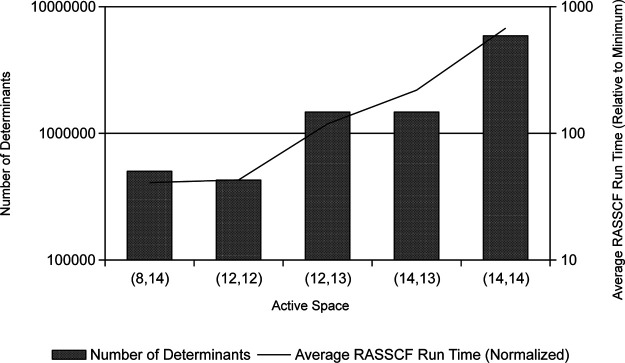
Number of determinants (bars) and average run time for the *RASSCF* module in OpenMolcas (line) for the largest active spaces in PASS. The run time is normalized relative to the fastest active space for each molecule. A logarithmic scale is used on the *y* axis.

Since scanning all active spaces up to (14,14) in size would need at least the same amount of time as using (14,14) alone, we have evaluated the performance of our protocols when large active spaces are removed. We demonstrate in Table S10 and Figure S111 of the SI that average excitation energy errors do not significantly suffer when the large active spaces (10,13), (10,14), (12,13), (12,14), (14,13), and (14,14) are removed from the set of active spaces to scan, while the time-to-solution needed is only 1/16 of that if these large active spaces were included. Figure 8 further illustrates the potential time savings for trimming the largest active spaces from scanning, leaving (8,14) and (12,12) as the largest active spaces (if (10,13), (10,14), (12,13), (12,14), (14,13), and (14,14) are all removed) or (12,13) and (14,13) as the largest active spaces (if only (10,14), (12,14), and (14,14) are removed).

### 3.6 Comparison with Active Spaces Selected by Chemical Intuition

Both GDM-AS and EDM-AS, on average, improve on the excitation energies over the intuitive active space selection for S_1_ through S_3_. For CAS-PDFT, deterioration is only seen for S_4_ from GDM-AS and both S_4_ and S_5_ from EDM-AS; for CASPT2, deterioration is seen for S_4_ and S_5_ from GDM-AS and only S_5_ from EDM-AS.

**Figure 9:**
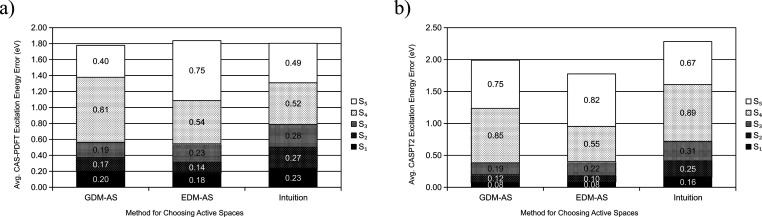
Average (a) CAS-PDFT and (b) CASPT2 excitation energy errors for all molecules in INAQ with active spaces in PASS as chosen by, from left to right, GDM-AS, EDM-AS, and intuitive rules. Experimental reference dipole moments are used in GDM-AS, and TD-M06-2X reference dipole moments are used in EDM-AS.

Figure 9 shows the average excitation energy error for all molecules in INAQ. Table S1 in the SI shows the active spaces chosen by intuition for each molecule in INAQ and the resulting CAS-PDFT and CASPT2 excitation energy errors, respectively. The intuitive active spaces are worse than GDM-AS and EDM-AS for the first three excited states and only outperform GDM-AS and EDM-AS at finding S_4_ and S_5_ excitation energies as described above. There are few data available at these excitations (only five data points for S_4_ and four data points for S_5_), and the excitation energies found by the intuitive active spaces for these excitations are still usually unsatisfactory, with errors significantly higher than S_1_–S_3_, so this may reflect an inherent difficulty in finding excitation energies for these higher roots as opposed to a strength in using the intuitive active spaces.

As mentioned previously, intuitive rules were used to decide the size of the active space, but the actual molecular orbitals specified in the intuitive rules were not enforced into the active space when we started the CASSCF calculations. As a test, for a subset of molecules in our dataset, we included specific orbitals in the intuitive active spaces using symmetry restrictions to ensure the same orbitals remain in the active space. As shown in Table S11 in the SI, this leads to drastically high excitation energy errors. The differences in the active orbitals that may contribute to the large error for the example in Table S11 are shown in Figures S112 and S113 in the SI.

**Figure 10:**
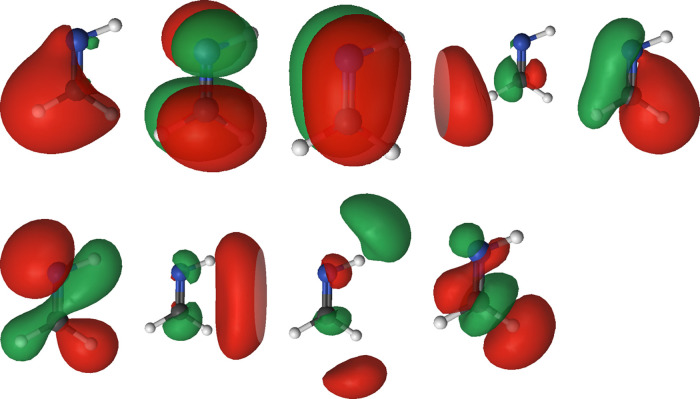
Molecular orbitals in the active space from the methanimine SA6-CAS(8,9)SCF calculations. The active space is selected by GDM-AS. Three of the orbitals here have a strong Rydberg character despite 11 valence orbitals being available. Visualized with Luscus^72^ at isovalue 0.04.

Often, chemical intuition used to decide the active space revolve around the valence orbitals. So far, we are not aware of a chemical-intuition-based rule to follow for choosing Rydberg orbitals to include into the active space. However, many low-lying excitations can be Rydberg-type, as shown in QUESTDB.^27^ Including the necessary number of Rydberg orbitals is not straightforward as including all Rydberg orbitals corresponding to a valence orbital tends to make active spaces too large; usually, including the proper number of these orbitals requires knowing the character of the excitations that one desires to study in the first place.^71^ GDM-AS and EDM-AS free users from this dilemma, by providing guidance for choosing the active space when the characters of the excitations are not known. They can choose a balanced active space that includes the necessary Rydberg orbitals, possibly at the expense of some unnecessary valence orbitals, so the relevant excitations can be described. Even when multiple valence orbitals are available, the active spaces chosen by GDM-AS and EDM-AS tend to include some orbitals with Rydberg characters instead of purely valence orbitals, as illustrated with the case of methanimine in Figure 10.

### 3.7 Anomalies

Figure 6 and Figure 7 show the average excitation energy error of all molecules in NAQ for each excitation and active space selection method. Figure 9 shows how these excitation energies compare to using intuition to select the active space size for INAQ. They all suggest that GDM-AS and EDM-AS are useful for finding up to the first three excitation energies. The errors for S_4_ and S_5_ are larger not only for active spaces selected by GDM-AS and EDM-AS, but also for (14,14) and active spaces selected by chemical intuition, as shown in Tables S1 and S10 in the SI. This is true for both CAS-PDFT and CASPT2. The large errors for S_4_ and S_5_ from GDM-AS and EDM-AS can be due to CASSCF predicting higher-lying excited states to become S_4_ and S_5_, resulting in the dipole moment errors to be computed between different states. CAS-PDFT and CASPT2 will include energy corrections from dynamic correlation and correct for the ordering of the states, but they do not necessarily give low excitation energy errors for higher-lying excitations. The anomaly of S_4_ and S_5_ may also be a result of the lack of S_4_ and S_5_ data in QUESTDB. For example, out of the 18 molecules considered in Figure 9, only five has S_4_ data and only four has S_5_ data.

In the dataset, two molecules give overall high CAS-PDFT excitation energy errors for most active spaces, including ones selected by GDM-AS or EDM-AS. Difluorocarbene was previously discussed as an inherently difficult case for excited-state calculations with CAS-PDFT. Carbon monoxide is another example of a molecule with high CAS-PDFT excitation energy errors. However, the active spaces selected by GDM-AS and EDM-AS give more accurate CASPT2 excitation energies (Tables S3 and S7 of the SI). This again suggests that the unsatisfactory excitation energies seen in these molecules are not indicative of a failure in the protocol, but a reflection of the accuracy of CAS-PDFT.

### 3.8 Use Cases for the Protocols

As discussed in the previous sections, we envision our protocols to be useful to find the low-lying excitation energies of molecules with nonzero dipole moments when we do not have much chemical insights in terms of which active space to choose, when the active space that would lead to good results would go beyond a human’s chemical insights, or chemical insights lead to active spaces that are too large to be affordable. The user would screen a set of active spaces of interest using only CASSCF and would choose a single active space to use in follow-up calculations using CAS-PDFT, CASPT2, or some other level of theory to account for dynamic correlation. While the user would, of course, be required to scan a set of candidate active spaces at the CASSCF level of theory, these calculations can be done completely in parallel, and the time-to-solution is limited only by the largest active space in the set. No visualization or selection of specific orbitals is needed. In addition, the user would only be required to apply CAS-PDFT or CASPT2 corrections to the active space chosen by the protocols.

Another potential use for the protocol is to choose a single active space for performing a potential energy scan or dynamics. As often done in other existing protocols, one could choose a geometry in the range of the geometries one might want to scan, such as the equilibrium geometry of the ground state, and use the active space chosen for this geometry for all geometries in the potential energy scan. Similarly, we can apply GDM-AS or EDM-AS on or around the equilibrium geometry of the molecule to choose an active space and then apply this same active space to other geometries in the potential energy scan. This would allow a user to choose an active space that is reasonably accurate and smaller than the maximally affordable active space without having to run GDM-AS or EDM-AS at every point and would significantly increase the cost–benefit of applying our protocols. If a user encounters the case where a discontinuity is found at a certain geometry, they may rerun GDM-AS or EDM-AS at that point and potentially obtain a new active space. One could then test whether this new active space gives a smooth curve, and this process can be repeated. Alternatively, one may compare the original active space and the new active space and identify their common orbitals and different orbitals and rationally construct a third active space to be applied on all geometries. Another possibility would be to use our protocols with iCAS,^4^ which could potentially ensure the same active space during a potential energy scan.

**Figure 11:**
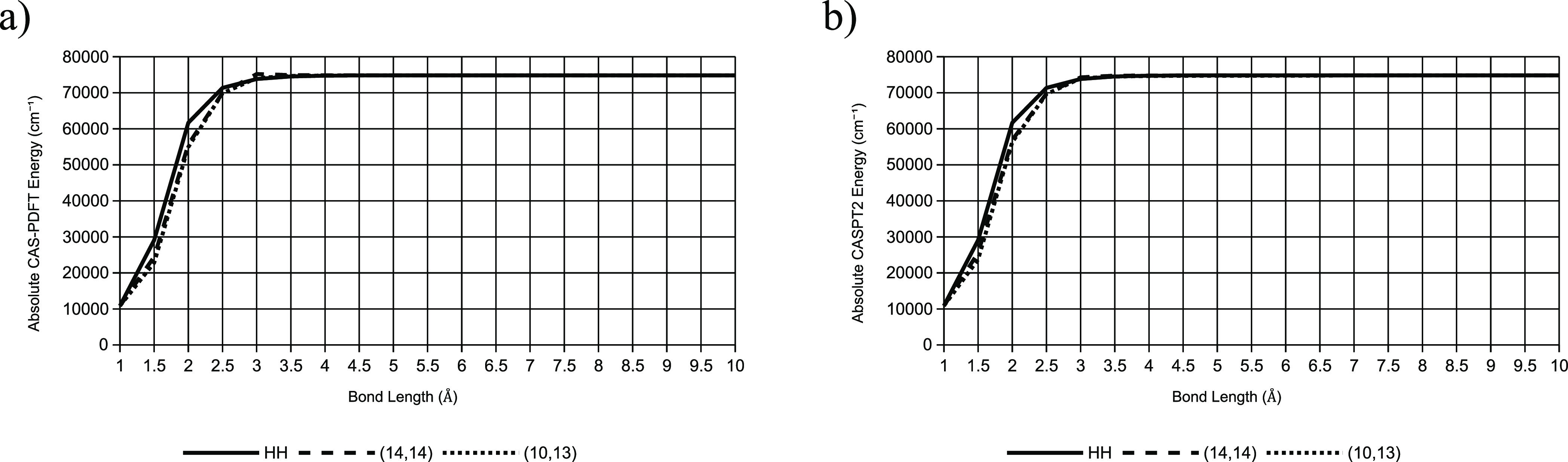
Potential energy curves of CO bond dissociation as calculated by the active spaces chosen by GDM-AS with (a) CAS-PDFT and (b) CASPT2, as compared to that calculated by the Hulburt–Hirschfelder equation. The curves from CAS-PDFT and CASPT2 have been scaled and shifted so that they can be compared directly to the Hulburt–Hirschfelder curve.

Here, we demonstrate this application by applying GDM-AS to choose the active space to scan the potential energy surface of the bond dissociation of carbon monoxide. The true equilibrium bond length of carbon monoxide is 1.128 Å;^6^ the active spaces chosen by GDM-AS at the nearest points in the potential energy scan, 1.0 and 1.5 Å, are (14,14) and (10,13), respectively. As seen in Figure 11, the potential energy curves for carbon monoxide bond dissociation solved with both of these active spaces, with CAS-PDFT and CASPT2 corrections, are smooth and qualitatively agree with the Hulburt–Hirschfelder curve,^6,73^ a reliable potential energy curve that depends only on parameters that are physical observables. The CASPT2 curves slightly outperform the CAS-PDFT curve in terms of smoothness. We note that we took additional care in ensuring the smoothness of the curves by using the final CASSCF orbitals from each step in the scan as the initial orbitals for the next step, using M06-2X to generate orbitals at the first step, and we advise users to do the same if GDM-AS is used for this purpose.

Although we have demonstrated that it is not necessary to run GDM-AS for every geometry of the potential energy scan, we have tested running GDM-AS for every geometry and the results show that, along with the obvious drawback in efficiency, choosing a different active space at each point of the potential energy curve results in a decrease in smoothness and qualitative correctness, as seen in the SI Figure S114.

Other than organic molecules, our protocols can also be applied on transition metal systems. We demonstrate that GDM-AS could theoretically be applied to finding the ground-state spin state of transition metal oxides in Table S12 of the SI.

### 3.9 Automation

To aid users in applying these protocols and truly fulfill our goal of automating their application, we provide a set of open-source Python scripts, hosted at https://github.com/sdonglab/DM-AS-Chooser. The code makes use of MolExtract,^74^ a modular parser for computational chemistry output files, which allows straightforward adaptation of the code for software packages beyond those used in this work.

## 4 Conclusions

We have designed two protocols, GDM-AS and EDM-AS, to automate the selection of appropriate active spaces for multireference calculations for molecules with nonzero dipole moments. They use the dipole moments of the system of interest as a guidance of the quality of the reference wave function. We have demonstrated that they are effective on a set of test molecules for finding the first three excitation energies using CAS-PDFT and CASPT2. In particular, our protocols find active spaces that give good excitation energies and are small and accessible, with significantly reduced time-to-solution and memory requirement than directly using a large active space such as (14,14), and without the need of manual selection or any iterative selection process as one usually faces in selecting the active space using chemical intuition.

We have demonstrated that a user can use the protocols designed in this work to efficiently find a useful active space with a size greater than (4,4) and smaller than (14,14). In addition, we have demonstrated that certain large active spaces from the set can be removed from consideration when these protocols are used, thus reducing the number of active spaces one needs to do CASSCF calculations with and reducing the time-to-solution needed to obtain reasonable results.

Although our evaluation of the protocols was done using CAS-PDFT and CASPT2, the protocol can in principle be used with any multireference method, including those whose reference wave functions are not from CASSCF, such as from RASSCF or DMRG.

A prominent advantage to using GDM-AS or EDM-AS is their highly automatic nature. Many existing protocols depend on manual analysis and setup of parameters. This can be beneficial if the focus is a few specific molecules at a few specific geometries, but it is detrimental to high-throughput applications where individual results cannot be practically analyzed. GDM-AS and EDM-AS do require a set of multiconfigurational calculations to be carried out, but the set of calculations can be done fully in parallel, and the protocols require no manual analysis beyond that required to properly interpret a single multireference calculation. All preceding calculations can be set up automatically with a workflow, and data processing is straightforward. We have provided the scripts for this automation. As such, these protocols can be applied to large datasets to select accurate and efficient active spaces tailored to each input system without user intervention.

In summary, we have demonstrated that one may use an easily obtainable physical observable, the dipole moment, to infer the quality of the wave function and to guide the selection of the active space for multireference methods. We anticipate this to expand the class of methods for automated active space selection and to lead to exciting science enabled by high-throughput multireference calculations.

## Supporting Information Available

Cartesian coordinates of all molecules studied; an example input file; additional figures and tables showing the performance of the active space selection protocols (active spaces chosen by intuition for eligible molecules, QUESTDB reference excitation energies, average excitation energy error with respect to the number of determinants, plots of CAS-PDFT and CASPT2 excitation energy errors with respect to dipole moment errors and application of GDM-AS and EDM-AS, comparisons of the excitation characters to those in QUESTDB, vGDM-AS and vEDM-AS (GDM-AS and EDM-AS with the direction of dipole moments considered), active spaces chosen by GDM-AS with experimental and DFT reference dipole moments, active spaces chosen by EDM-AS with TDDFT reference dipole moments, performance of EDM-AS with modifications and on double excitations, molecular orbital examples, active spaces chosen by the protocols compared to (14,14), excitation energy errors of protocols with respect to reduced active space sizes, comparison of intuitive active space with specific orbitals vs. with “automatic” active spaces, Hulburt–Hirschfelder curve of CO bond dissociation with GDM-AS run at every point, and performance of GDM-AS on transition metal oxides) (PDF)
Movie demonstrating the use of EDM-AS on an example molecule, hydrogen cyaphide (MP4)

## References

[ref1] Roos B. O.; Linse P.; Siegbahn P. E. M.; Blomberg M. R. A. A Simple Method for the Evaluation of the Second-Order-Perturbation Energy from External Double-Excitations with a CASSCF Reference Wavefunction. Chem. Phys. 1982, 66, 197–207. 10.1016/0301-0104(82)88019-1.

[ref2] Bao J. J.; Dong S. S.; Gagliardi L.; Truhlar D. G. Automatic Selection of an Active Space for Calculating Electronic Excitation Spectra by MS-CASPT2 or MC-PDFT. J. Chem. Theory Comput. 2018, 14, 2017–2025. 10.1021/acs.jctc.8b00032. 29486125

[ref3] Bao J. J.; Truhlar D. G. Automatic Active Space Selection for Calculating Electronic Excitation Energies Based on High-Spin Unrestricted Hartree–Fock Orbitals. J. Chem. Theory Comput. 2019, 15, 5308–5318. 10.1021/acs.jctc.9b00535. 31411880

[ref4] Lei Y.; Suo B.; Liu W. iCAS: Imposed Automatic Selection and Localization of Complete Active Spaces. J. Chem. Theory Comput. 2021, 17, 4846–4859. 10.1021/acs.jctc.1c00456. 34314180

[ref5] Stein C. J.; Reiher M. AutoCAS: A Program for Fully Automated Multiconfigurational Calculations. J. Comput. Chem. 2019, 40, 2216–2226. 10.1002/jcc.25869. 31173388

[ref6] Jeong W.; Stoneburner S. J.; King D.; Li R.; Walker A.; Lindh R.; Gagliardi L. Automation of Active Space Selection for Multireference Methods via Machine Learning on Chemical Bond Dissociation. J. Chem. Theory Comput. 2020, 16, 2389–2399. 10.1021/acs.jctc.9b01297. 32119542

[ref7] Sayfutyarova E. R.; Sun Q.; Chan G. K.-L.; Knizia G. Automated Construction of Molecular Active Spaces from Atomic Valence Orbitals. J. Chem. Theory Comput. 2017, 13, 4063–4078. 10.1021/acs.jctc.7b00128. 28731706

[ref8] Sayfutyarova E. R.; Hammes-Schiffer S. Constructing Molecular π-Orbital Active Spaces for Multireference Calculations of Conjugated Systems. J. Chem. Theory Comput. 2019, 1679 10.1021/acs.jctc.8b01196. 30689378 PMC6526033

[ref9] Golub P.; Antalik A.; Veis L.; Brabec J. Machine Learning-Assisted Selection of Active Spaces for Strongly Correlated Transition Metal Systems. J. Chem. Theory Comput. 2021, 17, 6053–6072. 10.1021/acs.jctc.1c00235. 34570505

[ref10] King D. S.; Gagliardi L. A Ranked-Orbital Approach to Select Active Spaces for High-Throughput Multireference Computation. J. Chem. Theory Comput. 2021, 17, 2817–2831. 10.1021/acs.jctc.1c00037. 33860669

[ref11] Bao J. L.; Sand A.; Gagliardi L.; Truhlar D. G. Correlated-Participating-Orbitals Pair-Density Functional Method and Application to Multiplet Energy Splittings of Main-Group Divalent Radicals. J. Chem. Theory Comput. 2016, 12, 4274–4283. 10.1021/acs.jctc.6b00569. 27438755

[ref12] Pulay P.; Hamilton T. P. UHF Natural Orbitals for Defining and Starting MC-SCF Calculations. J. Chem. Phys. 1988, 88, 4926–4933. 10.1063/1.454704.

[ref13] Khedkar A.; Roemelt M. Active Space Selection Based on Natural Orbital Occupation Numbers from N-Electron Valence Perturbation Theory. J. Chem. Theory Comput. 2019, 15, 3522–3536. 10.1021/acs.jctc.8b01293. 31059643

[ref14] Khedkar A.; Roemelt M. Extending the ASS1ST Active Space Selection Scheme to Large Molecules and Excited States. J. Chem. Theory Comput. 2020, 16, 4993–5005. 10.1021/acs.jctc.0c00332. 32644789

[ref15] Eade R. H. A.; Robb M. A. Direct Minimization in MC SCF Theory. The Quasi-Newton Method. Chem. Phys. Lett. 1981, 83, 362–368. 10.1016/0009-2614(81)85480-2.

[ref16] Roos B. O.; Taylor P. R.; Sigbahn P. E. M. A Complete Active Space SCF Method (CASSCF) Using a Density Matrix Formulated Super-CI Approach. Chem. Phys. 1980, 48, 157–173. 10.1016/0301-0104(80)80045-0.

[ref17] Hegarty D.; Robb M. A. Application of Unitary Group Methods to Configuration Interaction Calculations. Mol. Phys. 1979, 38, 1795–1812. 10.1080/00268977900102871.

[ref18] Knowles P. J.; Handy N. C. Unlimited Full Configuration Interaction Calculations. J. Chem. Phys. 1989, 91, 2396–2398. 10.1063/1.456997.

[ref19] Zimmerman P. M. Incremental Full Configuration Interaction. J. Chem. Phys. 2017, 146, 104102 10.1063/1.4977727. 28298122

[ref20] Vogiatzis K. D.; Ma D.; Olsen J.; Gagliardi L.; de Jong W. A. Pushing Configuration-Interaction to the Limit: Towards Massively Parallel MCSCF Calculations. J. Chem. Phys. 2017, 147, 184111 10.1063/1.4989858. 29141437

[ref21] Boggio-Pasqua M.; Groenhof G. On the Use of Reduced Active Space in CASSCF Calculations. Comput. Theor. Chem. 2014, 1040-1041, 6–13. 10.1016/j.comptc.2014.03.017.

[ref22] Krausbeck F.; Mendive-Tapia D.; Thom A. J. W.; Bearpark M. J. Choosing RASSCF Orbital Active Spaces for Multiple Electronic States. Comput. Theor. Chem. 2014, 1040-1041, 14–19. 10.1016/j.comptc.2014.03.030.

[ref23] Veryazov V.; Malmqvist P. Å.; Roos B. O. How to Select Active Space for Multiconfigurational Quantum Chemistry?. Int. J. Quantum Chem. 2011, 111, 3329–3338. 10.1002/qua.23068.

[ref24] Ma J.; Li S.; Li W. A Multireference Configuration Interaction Method Based on the Separated Electron Pair Wave Functions. J. Comput. Chem. 2006, 27, 39–47. 10.1002/jcc.20319.

[ref25] Cárdenas G.; Nogueira J. J. An Algorithm to Correct for the CASSCF Active Space in Multiscale QM/MM Calculations Based on Geometry Ensembles. Int. J. Quantum Chem. 2021, 121, e26533 10.1002/qua.26533.

[ref26] Cramer C. J.; Dulles F. J.; Storer J. W.; Worthington S. E. Full Valence Complete Active Space SCF, Multireference CI, and Density Functional Calculations of ^1^A_1_—^3^B_1_ Singlet—Triplet Gaps for the Valence-Isoelectronic Series BH^-^_2_, CH_2_, NH^+^_2_, AlH^-^_2_, SiH_2_, PH^+^_2_, GaH^-^_2_, GeH_2_, and AsH^+^_2_. Chem. Phys. Lett. 1994, 218, 387–394. 10.1016/0009-2614(94)00030-1.

[ref27] Véril M.; Scemama A.; Caffarel M.; Lipparini F.; Boggio-Pasqua M.; Jacquemin D.; Loos P.-F. QUESTDB: A Database of Highly Accurate Excitation Energies for the Electronic Structure Community. WIREs Comput. Mol. Sci. 2021, 11, e1517 10.1002/wcms.1517.

[ref75] Pastore M.; Angeli C.; Cimiraglia R. The Vertical Electronic Spectrum of Pyrrole: A Second and Third Order n-Electron Valence State Perturbation Theory Study. Chem. Phys. Lett. 2006, 422 (4), 522–528. 10.1016/j.cplett.2006.03.011.

[ref76] aRoothaan C. C. J. New Developments in Molecular Orbital Theory. Rev. Mod. Phys. 1951, 23, 69 10.1103/RevModPhys.23.69.

[ref28] Malmqvist P. Å.; Rendell A.; Roos B. O. The Restricted Active Space Self-Consistent-Field Method, Implemented with a Split Graph Unitary Group Approach. J. Phys. Chem. B 1990, 94, 5477–5482. 10.1021/j100377a011.

[ref77] Stein C. J.; Reiher M. Automated Selection of Active Orbital Spaces. J. Chem. Theory Comput. 2016, 12 (4), 1760–1771. 10.1021/acs.jctc.6b00156. 26959891

[ref78] Hanwell M. D; Curtis D. E; Lonie D. C; Vandermeersch T.; Zurek E.; Hutchison G. R Avogadro: An Advanced Semantic Chemical Editor, Visualization, and Analysis Platform. J. Cheminform. 2012, 4, 17 10.1186/1758-2946-4-17. 22889332 PMC3542060

[ref29] Angeli C.; Cimiraglia R.; Evangelisti S.; Leininger T.; Malrieu J.-P. Introduction of *n*-Electron Valence States for Multireference Perturbation Theory. J. Chem. Phys. 2001, 114, 10252–10264. 10.1063/1.1361246.

[ref30] Angeli C.; Cimiraglia R.; Malrieu J.-P. *N*-Electron Valence State Perturbation Theory: A Fast Implementation of the Strongly Contracted Variant. Chem. Phys. Lett. 2001, 350, 297–305. 10.1016/S0009-2614(01)01303-3.

[ref31] Angeli C.; Cimiraglia R.; Malrieu J.-P. *N*-Electron Valence State Perturbation Theory: A Spinless Formulation and an Efficient Implementation of the Strongly Contracted and of the Partially Contracted Variants. J. Chem. Phys. 2002, 117, 9138–9153. 10.1063/1.1515317.

[ref32] Angeli C.; Borini S.; Cestari M.; Cimiraglia R. A Quasidegenerate Formulation of the Second Order *n*-Electron Valence State Perturbation Theory Approach. J. Chem. Phys. 2004, 121, 4043–4049. 10.1063/1.1778711. 15332949

[ref33] Bauernschmitt R.; Ahlrichs R. Treatment of Electronic Excitations within the Adiabatic Approximation of Time Dependent Density Functional Theory. Chem. Phys. Lett. 1996, 256, 454–464. 10.1016/0009-2614(96)00440-X.

[ref34] Jamorski C.; Casida M. E.; Salahub D. R. Dynamic Polarizabilities and Excitation Spectra from a Molecular Implementation of Time-dependent Density-functional Response Theory: N_2_ as a Case Study. J. Chem. Phys. 1996, 104, 5134–5147. 10.1063/1.471140.

[ref35] Dong S. S.; Gagliardi L.; Truhlar D. G. Excitation Spectra of Retinal by Multiconfiguration Pair-Density Functional Theory. Phys. Chem. Chem. Phys. 2018, 20, 7265–7276. 10.1039/C7CP07275A. 29484326

[ref36] Li Manni G.; Carlson R. K.; Luo S.; Ma D.; Olsen J.; Truhlar D. G.; Gagliardi L. Multiconfiguration Pair-Density Functional Theory. J. Chem. Theory Comput. 2014, 10, 3669–3680. 10.1021/ct500483t. 26588512

[ref37] Carlson R. K.; Li Manni G.; Sonnenberger A. L.; Truhlar D. G.; Gagliardi L. Multiconfiguration Pair-Density Functional Theory: Barrier Heights and Main Group and Transition Metal Energetics. J. Chem. Theory Comput. 2015, 11, 82–90. 10.1021/ct5008235. 26574206

[ref38] Keller S.; Dolfi M.; Troyer M.; Reiher M. An Efficient Matrix Product Operator Representation of the Quantum Chemical Hamiltonian. J. Chem. Phys. 2015, 143, 244118 10.1063/1.4939000. 26723662

[ref39] Keller S.; Reiher M. Spin-Adapted Matrix Product States and Operators. J. Chem. Phys. 2016, 144, 134101 10.1063/1.4944921. 27059556

[ref40] Knecht S.; Hedegård E. D.; Keller S.; Kovyrshin A.; Ma Y.; Muolo A.; Stein C. J.; Reiher M. New Approaches for Ab Initio Calculations of Molecules with Strong Electron Correlation. Chimia 2016, 70, 244 10.2533/chimia.2016.244. 27131108

[ref41] NIST Computational Chemistry Comparison and Benchmark Database, NIST Standard Reference Database Number 101; Johnson R. D. III, Ed.; 2022.

[ref42] Loos P.-F.; Comin M.; Blase X.; Jacquemin D. Reference Energies for Intramolecular Charge-Transfer Excitations. J. Chem. Theory Comput. 2021, 17, 3666–3686. 10.1021/acs.jctc.1c00226. 33955742

[ref43] Loos P.-F.; Jacquemin D. A Mountaineering Strategy to Excited States: Highly Accurate Energies and Benchmarks for Bicyclic Systems. J. Phys. Chem. A 2021, 125, 10174–10188. 10.1021/acs.jpca.1c08524. 34792354

[ref79] aHohenberg P.; Kohn W. Inhomogenous Electron Gas. Phys. Rev. 1964, 136 (3B), B864–B871. 10.1103/PhysRev.136.B864.

[ref44] Frisch M. J.; Trucks G. W.; Cheeseman J. R.; Scalmani G.; Marenich A. V.; Zheng J.; Schlegel H. B.; Scuseria G. E.; Robb M. A.; Barone V.; Petersson G. A.; Nakatsuji H.; Li X.; Caricato M.; Bloino J.; Janesko B. G.; Gomperts R.; Mennucci B.; Hratchian H. P.; Ortiz J. V.; Izmaylov A. F.; Sonnenberg J. L.; Williams-Young D.; Ding F.; Lipparini F.; Egidi F.; Goings J.; Peng B.; Petrone A.; Henderson T.; Ranasinghe D.; Zakrzewski V. G.; Gao J.; Rega N.; Zheng G.; Liang W.; Hada M.; Ehara M.; Toyota K.; Fukuda R.; Hasegawa J.; Ishida M.; Nakajima T.; Honda Y.; Kitao O.; Nakai H.; Vreven T.; Throssell K.; Montgomery J. A. Jr.; Peralta J. E.; Ogliaro F.; Bearpark M. J.; Heyd J. J.; Brothers E. N.; Kudin K. N.; Staroverov V. N.; Keith T. A.; Kobayashi R.; Normand J.; Raghavachari K.; Rendell A. P.; Burant J. C.; Iyengar S. S.; Tomasi J.; Cossi M.; Millam J. M.; Klene M.; Adamo C.; Cammi R.; Ochterski J. W.; Martin R. L.; Morokuma K.; Farkas O.; Foresman J. B.; Fox D. J.; Gaussian 16, Revision A.03; Gaussian, Inc.: Wallingford CT, 2016.

[ref45] Zhao Y.; Truhlar D. G. The M06 Suite of Density Functionals for Main Group Thermochemistry, Thermochemical Kinetics, Noncovalent Interactions, Excited States, and Transition Elements: Two New Functionals and Systematic Testing of Four M06-Class Functionals and 12 Other Functionals. Theor. Chem. Account. 2008, 120, 215–241. 10.1007/s00214-007-0310-x.

[ref46] Sarkar R.; Boggio-Pasqua M.; Loos P.-F.; Jacquemin D. Benchmarking TD-DFT and Wave Function Methods for Oscillator Strengths and Excited-State Dipole Moments. J. Chem. Theory Comput. 2021, 17, 1117–1132. 10.1021/acs.jctc.0c01228. 33492950

[ref47] Roos B. O.; Lindh R.; Malmqvist P.-Å.; Veryazov V.; Widmark P.-O. Main Group Atoms and Dimers Studied with a New Relativistic ANO Basis Set. J. Phys. Chem. A 2004, 108, 2851–2858. 10.1021/jp031064+.

[ref48] Pritchard B. P.; Altarawy D.; Didier B.; Gibson T. D.; Windus T. L. New Basis Set Exchange: An Open, Up-to-Date Resource for the Molecular Sciences Community. J. Chem. Inf. Model. 2019, 59, 4814–4820. 10.1021/acs.jcim.9b00725. 31600445

[ref49] Fdez. Galván I.; Vacher M.; Alavi A.; Angeli C.; Aquilante F.; Autschbach J.; Bao J. J.; Bokarev S. I.; Bogdanov N. A.; Carlson R. K.; Chibotaru L. F.; Creutzberg J.; Dattani N.; Delcey M. G.; Dong S. S.; Dreuw A.; Freitag L.; Frutos L. M.; Gagliardi L.; Gendron F.; Giussani A.; González L.; Grell G.; Guo M.; Hoyer C. E.; Johansson M.; Keller S.; Knecht S.; Kovačević G.; Källman E.; Li Manni G.; Lundberg M.; Ma Y.; Mai S.; Malhado J. P.; Malmqvist P. Å.; Marquetand P.; Mewes S. A.; Norell J.; Olivucci M.; Oppel M.; Phung Q. M.; Pierloot K.; Plasser F.; Reiher M.; Sand A. M.; Schapiro I.; Sharma P.; Stein C. J.; Sørensen L. K.; Truhlar D. G.; Ugandi M.; Ungur L.; Valentini A.; Vancoillie S.; Veryazov V.; Weser O.; Wesołowski T. A.; Widmark P.-O.; Wouters S.; Zech A.; Zobel J. P.; Lindh R. OpenMolcas: From Source Code to Insight. J. Chem. Theory Comput. 2019, 15, 5925–5964. 10.1021/acs.jctc.9b00532. 31509407

[ref50] Douglas M.; Kroll N. M. Quantum Electrodynamical Corrections to the Fine Structure of Helium. Ann. Phys. 1974, 82, 89–155. 10.1016/0003-4916(74)90333-9.

[ref51] Hess B. A. Applicability of the No-Pair Equation with Free-Particle Projection Operators to Atomic and Molecular Structure Calculations. Phys. Rev. A 1985, 32, 756–763. 10.1103/PhysRevA.32.756. 9896123

[ref52] Hess B. A. Relativistic Electronic-Structure Calculations Employing a Two-Component No-Pair Formalism with External-Field Projection Operators. Phys. Rev. A 1986, 33, 3742–3748. 10.1103/PhysRevA.33.3742. 9897114

[ref53] Perdew J. P.; Burke K.; Ernzerhof M. Generalized Gradient Approximation Made Simple. Phys. Rev. Lett. 1996, 77, 3865–3868. 10.1103/PhysRevLett.77.3865. 10062328

[ref54] Ghigo G.; Roos B. O.; Malmqvist P.-Å. A Modified Definition of the Zeroth-Order Hamiltonian in Multiconfigurational Perturbation Theory (CASPT2). Chem. Phys. Lett. 2004, 396, 142–149. 10.1016/j.cplett.2004.08.032.

[ref55] Zobel J. P.; Nogueira J. J.; González L. The IPEA Dilemma in CASPT2. Chem. Sci. 2017, 8, 1482–1499. 10.1039/C6SC03759C. 28572908 PMC5452265

[ref56] Yanai T.; Tew D. P.; Handy N. C. A New Hybrid Exchange–Correlation Functional Using the Coulomb-Attenuating Method (CAM-B3LYP). Chem. Phys. Lett. 2004, 393, 51–57. 10.1016/j.cplett.2004.06.011.

[ref57] Chai J.-D.; Head-Gordon M. Long-Range Corrected Hybrid Density Functionals with Damped Atom–Atom Dispersion Corrections. Phys. Chem. Chem. Phys. 2008, 10, 6615–6620. 10.1039/B810189B. 18989472

[ref58] Perdew J. P.; Burke K.; Ernzerhof M. Generalized Gradient Approximation Made Simple [Phys. Rev. Lett. 77, 3865 (1996)]. Phys. Rev. Lett. 1997, 78, 1396–1396. 10.1103/PhysRevLett.78.1396. 10062328

[ref59] Adamo C.; Barone V. Toward Reliable Density Functional Methods without Adjustable Parameters: The PBE0 Model. J. Chem. Phys. 1999, 110, 6158–6170. 10.1063/1.478522.

[ref60] Heyd J.; Scuseria G. E. Efficient Hybrid Density Functional Calculations in Solids: Assessment of the Heyd–Scuseria–Ernzerhof Screened Coulomb Hybrid Functional. J. Chem. Phys. 2004, 121, 1187–1192. 10.1063/1.1760074. 15260659

[ref61] Heyd J.; Scuseria G. E. Assessment and Validation of a Screened Coulomb Hybrid Density Functional. J. Chem. Phys. 2004, 120, 7274–7280. 10.1063/1.1668634. 15267636

[ref62] Heyd J.; Peralta J. E.; Scuseria G. E.; Martin R. L. Energy Band Gaps and Lattice Parameters Evaluated with the Heyd-Scuseria-Ernzerhof Screened Hybrid Functional. J. Chem. Phys. 2005, 123, 174101 10.1063/1.2085170. 16375511

[ref63] Heyd J.; Scuseria G. E.; Ernzerhof M. Erratum: “Hybrid Functionals Based on a Screened Coulomb Potential” [J. Chem. Phys. 118, 8207 (2003)]. J. Chem. Phys 2006, 124, 219906 10.1063/1.2204597.

[ref64] Henderson T. M.; Izmaylov A. F.; Scalmani G.; Scuseria G. E. Can Short-Range Hybrids Describe Long-Range-Dependent Properties?. J. Chem. Phys. 2009, 131, 044108 10.1063/1.3185673. 19655838

[ref65] Izmaylov A. F.; Scuseria G. E.; Frisch M. J. Efficient Evaluation of Short-Range Hartree-Fock Exchange in Large Molecules and Periodic Systems. J. Chem. Phys. 2006, 125, 104103 10.1063/1.2347713. 16999511

[ref66] Krukau A. V.; Vydrov O. A.; Izmaylov A. F.; Scuseria G. E. Influence of the Exchange Screening Parameter on the Performance of Screened Hybrid Functionals. J. Chem. Phys. 2006, 125, 224106 10.1063/1.2404663. 17176133

[ref67] Zhao Y.; Truhlar D. G. Comparative DFT Study of van Der Waals Complexes: Rare-Gas Dimers, Alkaline-Earth Dimers, Zinc Dimer, and Zinc-Rare-Gas Dimers. J. Phys. Chem. A 2006, 110, 5121–5129. 10.1021/jp060231d. 16610834

[ref68] Zhao Y.; Truhlar D. G. Density Functional for Spectroscopy: No Long-Range Self-Interaction Error, Good Performance for Rydberg and Charge-Transfer States, and Better Performance on Average than B3LYP for Ground States. J. Phys. Chem. A 2006, 110, 13126–13130. 10.1021/jp066479k. 17149824

[ref69] King D. S.; Hermes M. R.; Truhlar D. G.; Gagliardi L. Large-Scale Benchmarking of Multireference Vertical-Excitation Calculations via Automated Active-Space Selection. J. Chem. Theory Comput. 2022, 18, 6065–6076. 10.1021/acs.jctc.2c00630. 36112354 PMC9558375

[ref70] Bearpark M. J.; Ogliaro F.; Vreven T.; Boggio-Pasqua M.; Frisch M. J.; Larkin S. M.; Morrison M.; Robb M. A. CASSCF Calculations for Photoinduced Processes in Large Molecules: Choosing When to Use the RASSCF, ONIOM and MMVB Approximations. J. Photochem. Photobiol. 2007, 190, 207–227. 10.1016/j.jphotochem.2007.05.008.

[ref71] Nakano H.; Tsuneda T.; Hashimoto T.; Hirao K. Theoretical Study of the Excitation Spectra of Five-membered Ring Compounds: Cyclopentadiene, Furan, and Pyrrole. J. Chem. Phys. 1996, 104, 2312–2320. 10.1063/1.470926.

[ref72] Kovačević G.; Veryazov V. Luscus: Molecular Viewer and Editor for MOLCAS. J. Cheminform. 2015, 7, 16 10.1186/s13321-015-0060-z. 25984240 PMC4432095

[ref73] Hulburt H. M.; Hirschfelder J. O. Potential Energy Functions for Diatomic Molecules. J. Chem. Phys. 1941, 9, 61–69. 10.1063/1.1750827.

[ref74] Chintala N.; Dong S. S. MolExtract v1.0.0: Modular Parser of Computational Chemistry Output Files. 2023, 10.5281/zenodo.7700453.

