# Supplementary material for: Automated Active Space Selection with Dipole Moments
Source: J Chem Theory Comput. 2023 Apr 11;19(9):2469–83. doi: 10.1021/acs.jctc.2c01128 (PMC10629219; doi:10.1021/acs.jctc.2c01128)
Supplement: Supplementary file 1 — ct2c01128_si_001.pdf [file ct2c01128_si_001.pdf]

Supporting Information

Automated Active Space Selection with Dipole Moments

Benjamin W. Kaufold,[a] Nithin Chintala,[a] Pratima Pandeya,[a,c] Sijia S. Dong*[a,b]

a. Department of Chemistry and Chemical Biology, Northeastern University, Boston, Massachusetts 02115, United States

b. Department of Physics and Department of Chemical Engineering, Northeastern University, Boston, Massachusetts 02115, United States

c. The Institute for Experiential AI, Northeastern University, Boston, Massachusetts 02115, United States

Table of Contents

Cartesian Coordinates 3

Example OpenMolcas Input File 7

Active Spaces Chosen by Intuition for Eligible Molecules 8

QUESTDB Reference Excitation Energies 9

Average Excitation Energy Error with Respect to Number of Determinants 12

Active Spaces Chosen by GDM-AS with Experimental and DFT Reference Dipole Moments 13

Plots of CAS-PDFT Excitation Energy Error w.r.t. S_0 CASSCF Dipole Moment Error at All Active Spaces; Application of GDM-AS 15

Plots of CASPT2 Excitation Energy Error w.r.t. S_0 CASSCF Dipole Moment Error at All Active Spaces; Application of GDM-AS 28

Comparisons of the Excitation Characters to Those in QUESTDB 41

vGDM-AS (GDM-AS with the Direction of Dipole Moments Considered) 41

Active Spaces Chosen by EDM-AS with TDDFT Reference Dipole Moments 44

Plots of CAS-PDFT Excitation Energy Error w.r.t. S_n (n>0) CASSCF Dipole Moment Error at All Active Spaces; Application of EDM-AS 46

Plots of CASPT2 Excitation Energy Error w.r.t. S_n (n>0) CASSCF Dipole Moment Error at All Active Spaces; Application of EDM-AS 59

Performance of EDM-AS with S_4 and S_5 Excitations Ignored 72

Performance of EDM-AS on Describing Double Excitations 73

vEDM-AS (EDM-AS with the Direction of Dipole Moments Considered) 73

Molecular Orbital Examples 76

Active Spaces Chosen by Protocols Compared to (14,14) 76
Excitation Energy Errors of Protocols with Respect to Reduced Active Space Sizes.................. 78
Comparison of Intuitive Active Space with Specific Orbitals vs. with "Automatic" Active Spaces 78
Hulburt-Hirschfelder Curve of CO Bond Dissociation with GDM-AS Run at Every Point........ 81
Performance of GDM-AS on Transition Metal Oxides... 81
References.. 82

Cartesian Coordinates

All coordinates are presented in units of Å.

Acetaldehyde

H	1.368812223660	-0.243494045287	0.000000000000
C	0.376429078210	0.247019016948	0.000000000000
O	0.298194902898	1.443637060131	0.000000000000
C	-0.802790204404	-0.678554441781	0.000000000000
H	-1.730549086219	-0.114212997427	0.000000000000
H	-0.752652823991	-1.325805958066	0.877016976379
H	-0.752652823991	-1.325805958066	-0.877016976379

Aniline

H	0.858685846194	0.000000000000	3.158936109916
H	-0.858685846194	0.000000000000	3.158936109916
N	0.000000000000	0.000000000000	2.644400952959
H	2.140048245157	0.000000000000	1.095015520573
C	0.000000000000	0.000000000000	1.269549094876
H	-2.140048245157	0.000000000000	1.095015520573
C	1.201688459590	0.000000000000	0.555176912286
C	-1.201688459590	0.000000000000	0.555176912286
C	1.194655433658	0.000000000000	-0.829257134100
C	-1.194655433658	0.000000000000	-0.829257134100
H	2.136938037988	0.000000000000	-1.360178885424
C	0.000000000000	0.000000000000	-1.536714058406
H	-2.136938037988	0.000000000000	-1.360178885424
H	0.000000000000	0.000000000000	-2.616621035930

Azulene

H	0.000000000000	0.000000000000	3.824088705904
H	2.167728607119	0.000000000000	2.284844015314
C	0.000000000000	0.000000000000	2.744646494774
H	-2.167728607119	0.000000000000	2.284844015314
C	1.144843357253	0.000000000000	1.943420647644
C	-1.144843357253	0.000000000000	1.943420647644
H	2.646142336907	0.000000000000	-0.265464401139
C	0.744703713350	0.000000000000	0.603596215302
C	-0.744703713350	0.000000000000	0.603596215302
H	-2.646142336907	0.000000000000	-0.265464401139
C	1.585626982783	0.000000000000	-0.495771754542
C	-1.585626982783	0.000000000000	-0.495771754542
C	1.259297233112	0.000000000000	-1.846403409966
C	-1.259297233112	0.000000000000	-1.846403409966
H	2.096727537884	0.000000000000	-2.531993466625
C	0.000000000000	0.000000000000	-2.434755787724
H	-2.096727537884	0.000000000000	-2.531993466625
H	0.000000000000	0.000000000000	-3.518435104954

Carbon Monoxide

C	0.000000000000	0.000000000000	0.560423219362
O	0.000000000000	0.000000000000	-0.560423219362

Nitrosomethane

C	-0.939125054773	0.000000000000	-0.569866000582
N	0.002137910618	0.000000000000	0.565552541214
O	1.146502260348	0.000000000000	0.235703548864
H	-0.411882539770	0.000000000000	-1.521642900337
H	-1.572181288190	0.879410331944	-0.452853589604
H	-1.572181288190	-0.879410331944	0.452853589604

Cyclopropene

C	0.000000000000	0.000000000000	-0.878335688759
C	0.000000000000	0.642296753031	0.477616143336
C	0.000000000000	-0.642296753031	0.477616143336
H	0.911368785064	0.000000000000	-1.468538171568
H	-0.911368785064	0.000000000000	-1.468538171568
H	0.000000000000	1.571763912969	1.014509022612
H	0.000000000000	-1.571763912969	1.014509022612

Diazirine

H	0.000000000000	0.929579141370	0.662303608837
H	0.000000000000	-0.929579141370	0.662303608837
C	0.000000000000	0.000000000000	0.115337040766
N	0.604676073361	0.000000000000	-1.219366447783
N	-0.604676073361	0.000000000000	-1.219366447783

Diazomethane

N	0.000000000000	0.000000000000	1.772864269520
N	0.000000000000	0.000000000000	0.648251019908
C	0.000000000000	0.000000000000	-0.642437246369
H	0.951876106251	0.000000000000	-1.139339021529
H	-0.951876106251	0.000000000000	-1.139339021529

Difluorocarbene

C	0.000000000000	0.000000000000	0.194385386842
F	1.022941747563	0.000000000000	-0.597192693421
F	-1.022941747563	0.000000000000	-0.597192693421

Formaldehyde

O	0.000000000000	0.000000000000	1.186392529214
C	0.000000000000	0.000000000000	-0.009101051864
H	0.938275661672	0.000000000000	-0.588645738675
H	-0.938275661672	0.000000000000	-0.588645738675

Formamide

H	-1.227400354310	-0.415041806094	0.000000000000
C	-0.279229142039	0.145468224966	0.000000000000
O	-0.237630270322	1.349672014892	0.000000000000
N	0.807109394629	-0.663738077694	0.000000000000
H	1.726052501329	-0.254376351039	0.000000000000
H	0.711097870713	-1.661984005032	0.000000000000

Formyl Fluoride

H	-0.956206524026	-0.572547111987	0.000000000000
C	-0.062585662330	0.056463540780	0.000000000000
O	-0.009595364814	1.228019127349	0.000000000000
F	1.028387551170	-0.711935556142	0.000000000000

Hydrogen Chloride

H	0.000000000000	0.000000000000	0.638531546993
Cl	0.000000000000	0.000000000000	-0.638531546993

Hydrogen Cyaphide

H	0.000000000000	0.000000000000	1.056801735039
C	0.000000000000	0.000000000000	-0.014498252428
P	0.000000000000	0.000000000000	-1.542303482612

Hydrogen Sulfide

S	0.000000000000	0.000000000000	0.783794900443
H	0.964131503231	0.000000000000	-0.141897450221
H	-0.964131503231	0.000000000000	-0.141897450221

Imidazole

N	0.025811213622	0.969820115808	0.000000000000
H	-2.074721214555	0.555856774529	0.000000000000
C	-1.076817510721	0.154288021596	0.000000000000
C	1.053751043201	0.164990244595	0.000000000000
H	2.089249346252	0.459904643111	0.000000000000
C	-0.701677799795	-1.156875349903	0.000000000000
N	0.669972480205	-1.137621881399	0.000000000000
H	-1.262718281651	-2.072688134996	0.000000000000
H	1.277150723442	-1.937674433341	0.000000000000

Ketene

O	0.000000000000	0.000000000000	1.817239844581
C	0.000000000000	0.000000000000	0.663737931444
C	0.000000000000	0.000000000000	-0.643431975255
H	0.939615119541	0.000000000000	-1.168772900385
H	-0.939615119541	0.000000000000	-1.168772900385

Methanimine

H	0.973012217330	1.075702800871	0.000000000000
N	0.020458397670	0.712371417790	0.000000000000
C	0.019564459321	-0.548035630286	0.000000000000
H	0.919748403390	-1.165875957006	0.000000000000
H	-0.932783477711	-1.074162631369	0.000000000000

Nitrobenzene

O	1.072260421710	0.000000000000	3.235437273537
O	-1.072260421710	0.000000000000	3.235437273537
N	0.000000000000	0.000000000000	2.676184898282
H	2.131179218489	0.000000000000	1.105044919983
C	0.000000000000	0.000000000000	1.200148047573
H	-2.131179218489	0.000000000000	1.105044919983
C	1.214061916502	0.000000000000	0.537341236364
C	-1.214061916502	0.000000000000	0.537341236364
C	1.205517271217	0.000000000000	-0.848489950755
C	-1.205517271217	0.000000000000	-0.848489950755
H	2.141067602936	0.000000000000	-1.388582622519
C	0.000000000000	0.000000000000	-1.538482623862
H	-2.141067602936	0.000000000000	-1.388582622519
H	0.000000000000	0.000000000000	-2.619352035210

Nitrosyl Hydride

H	-0.894963592927	-0.501138297460	0.000000000000
N	-0.030803278534	0.104369786031	0.000000000000
O	0.925766871461	-0.603231488570	0.000000000000

Benzonitrile

N	0.000000000000	0.000000000000	3.864825101739
C	0.000000000000	0.000000000000	2.717409607418
H	2.139361506726	0.000000000000	1.138982015574
C	0.000000000000	0.000000000000	1.280933567431
H	-2.139361506726	0.000000000000	1.138982015574
C	1.209629827247	0.000000000000	0.588966517606
C	-1.209629827247	0.000000000000	0.588966517606
C	1.203775805919	0.000000000000	-0.795993563532
C	-1.203775805919	0.000000000000	-0.795993563532
H	2.140234996646	0.000000000000	-1.334933503764
C	0.000000000000	0.000000000000	-1.488184021595
H	-2.140234996646	0.000000000000	-1.334933503764
H	0.000000000000	0.000000000000	-2.569027186760

Propynal

H	0.980106830962	1.740653046470	0.000000000000
O	-1.018039342159	1.947136637692	0.000000000000
C	-0.020067355944	1.282096885689	0.000000000000
C	-0.023502274908	-0.171178570699	0.000000000000

```
C          0.027411205546     -1.367763263728      0.000000000000
H          0.054090936503     -2.430944735425      0.000000000000
```

Pyridine

```
H         -0.000000000013      0.000000000000      2.405890757755
H          2.145253651425      0.000000000000      1.123038704381
C         -0.000000000007      0.000000000000      1.324556533810
H         -2.145253651437      0.000000000000      1.123038704359
C          1.191076262927      0.000000000000      0.616600630036
C         -1.191076262934      0.000000000000      0.616600630023
C          1.136062365819      0.000000000000     -0.770256412152
C         -1.136062365811      0.000000000000     -0.770256412164
H          2.049168422468      0.000000000000     -1.353340113761
N          0.000000000007      0.000000000000     -1.462532908504
H         -2.049168422454      0.000000000000     -1.353340113783
```

Pyrrole

```
H          0.000000000000      0.000000000000      2.189484522197
H          2.102164562642      0.000000000000      0.833832814324
N          0.000000000000      0.000000000000      1.186285410999
H         -2.102164562642      0.000000000000      0.833832814324
C          1.118064391725      0.000000000000      0.400972136393
C         -1.118064391725      0.000000000000      0.400972136393
C          0.710154054147      0.000000000000     -0.906637120571
C         -0.710154054147      0.000000000000     -0.906637120571
H          1.357110796041      0.000000000000     -1.766052796775
H         -1.357110796041      0.000000000000     -1.766052796775
```

Thiophene

```
H          2.265475229227      0.000000000000      0.741574853245
S          0.000000000000      0.000000000000      1.636414986184
H         -2.265475229227      0.000000000000      0.741574853245
C          1.229366727934      0.000000000000      0.449744711258
C         -1.229366727934      0.000000000000      0.449744711258
C          0.710872038385      0.000000000000     -0.808778475165
C         -0.710872038385      0.000000000000     -0.808778475165
H          1.317791241796      0.000000000000     -1.700748582427
H         -1.317791241796      0.000000000000     -1.700748582427
```

Example OpenMolcas Input File

OpenMolcas Multireference Calculations Using the Active Space (10,10)

```
&GATEWAY
    Title = Multireference
    Coord = $CurrDir/optimized.xyz
    Basis = ANO-RCC-VTZP
    Group = NoSym
```

```
&SEWARD
    grid input
    grid=ultrafine
    end of grid input

&SCF
     KSDFT = M062X

&RASSCF
    nActEl = 10
    Ras2 = 10
    Charge = 0
    Spin = 1
    CIRoot = 6 6 1
    OutOrbital = Natural; 6
    LevShft = 1.0

&CASPT2
     Prop
     MaxIter = 10000

&MCPDFT
     KSDFT = T:PBE

&RASSI
```

Active Spaces Chosen by Intuition for Eligible Molecules

Table S1: Active spaces chosen by intuitive rules for molecules in INAQ, and excitation energy errors for these active spaces as calculated by CAS-PDFT and CASPT2.

Molecule	Active Space	CAS-PDFT Excitation Energy Error (eV)					CASPT2 Excitation Energy Error (eV)				
		S_1	S_2	S_3	S_4	S_5	S_1	S_2	S_3	S_4	S_5
Formaldehyde	(12,10)	0.05	0.31	0.36	0.70	0.31	0.01	0.25	0.12	0.94	0.56
Pyridine	(8,7)	0.17	0.37	0.49	0.57	0.58	0.17	0.20	0.05	0.43	0.41
Diazirine	(12,10)	0.26	0.11	0.18	0.11		0.04	0.10	0.06	0.25	
Ketene	(12,10)	0.40	1.13	0.37	0.64		0.11	1.32	0.82	1.96	
Diazomethane	(12,10)	0.41	0.42	0.42			0.05	0.09	0.20		
Hydrogen Cyaphide	(10,9)	0.10	0.16	0.16			0.19	0.13	0.13		
Nitrosyl Hydride	(12,9)	0.07	0.04	0.04			0.12	0.09	0.47		
Nitrosomethane	(12,9)	0.04	0.02	0.04			0.17	0.02	0.15		
Cyclopropene	(12,12)	0.33	0.15				0.34	0.64			
Hydrogen Sulfide	(8,12)	0.33	0.15				0.13	0.10			
Propynal	(10,8)	0.24	0.02				0.10	0.29			
Acetaldehyde	(10,8)	0.17					0.01				
Formamide	(12,9)	0.56					0.94				
Hydrogen Chloride	(8,10)	0.20	0.19				0.08	0.06			
Methanimine	(12,11)	0.12					0.08				
Aniline	(8,7)			0.19					0.34		
Azulene	(10,10)	0.27	0.46	0.58	0.60	0.60	0.02	0.01	0.72	0.86	0.84
Benzonitrile	(12,11)					0.48					0.89
Average		0.23	0.27	0.28	0.52	0.49	0.16	0.25	0.31	0.89	0.67

QUESTDB Reference Excitation Energies

Accurate vertical excitation energy data were obtained from the QUEST database (QUESTDB). The reference QUEST#5[1] was used except for molecules exhibiting intramolecular charge transfer excitations; data for these molecules was obtained from QUEST#6[2] instead. Only for the molecule azulene, data was obtained from QUEST#7.[3] Degenerate excitations were separated into individual excitations with identical energies. The specific method used to obtain accurate excitation energy data is given in Table S2 for each molecule. A variety of methods and basis sets were used, but the most relevant methods, all using the frozen core approximation, are as follows. Method abbreviations are exactly as they are in the corresponding QUEST references, except for reference energies from QUEST#6, where “CC-CBS/aug-cc-pVQZ" refers to a sequentially corrected coupled cluster method.

- For molecules in QUEST#5, when computationally affordable, the determinant-driven configuration interaction using a perturbative selection made iteratively (CIPSI) method was used to obtain variational energies, and a second-order perturbation theory correction was applied to estimate the contribution of orbitals outside the active space. This correction was linearly extrapolated to 0 to obtain excitation energies with nearly full-CI accuracy. This is abbreviated in Table S2 as simply "FCI”.
- For molecules in QUEST#5 where the above method was not affordable, linear-response (LR) coupled cluster theory was used instead with full inclusion of up to triples (abbreviated here as CCSDT, as in QUEST) or quadruples (CCSDTQ).
- Additionally, in QUEST#5, excitation energies may be obtained by combining initial energies calculated at an expensive method and small basis set with corrections using a cheaper method and larger basis set. This is abbreviated in Table S2 as the addition and subtraction of methods: e.g., "CCSDTQ/6-31+G(d) + [CCSDT/aug-cc-pVTZ – CCSDT/6-

31+G(d)] means that excitation energies are calculated at the CCSDTQ/6-31+G(d) level of theory and corrected using the CCSDT method to expand 6-31+G(d) results to aug-cc-pVTZ quality.

- For molecules in reference QUEST#6, an incremental approach is used, where an initial guess generated at the equation-of-motion (EOM) CCSDT or CCSDT-3 level with the cc-pVDZ basis set is corrected using multiple CC methods and basis sets to approach the CCSDT at the complete basis set (CBS) limit. This is abbreviated in Table S2 as "CC-CBS/aug-cc-pVQZ". Please refer to QUEST#6 for the complete procedure.

Table S2: Reference excitation energies from QUESTDB and methods used to obtain them.

Molecule	Excitation Energy (eV)				
	S_1	S_2	S_3	S_4	S_5
Acetaldehyde	4.31 FCI/aug-cc-pVTZ				
Aniline			5.48 CC-CBS/aug-cc-pVQZ		
Azulene	2.161 CCSDT/6-31+G(d) + [CC3/aug-cc-PVTZ - CC3/6-31+G(d)]	3.849 CCSDT/6-31+G(d) + [CC3/aug-cc-PVTZ - CC3/6-31+G(d)]	4.510 CCSDT/6-31+G(d) + [CC3/aug-cc-PVTZ - CC3/6-31+G(d)]	4.874 CCSDT/6-31+G(d) + [CC3/aug-cc-PVTZ - CC3/6-31+G(d)]	4.956 CCSDT/6-31+G(d) + [CC3/aug-cc-PVTZ - CC3/6-31+G(d)]
Benzonitrile					7.05 CC-CBS/aug-cc-pVQZ
Carbon Monoxide	8.49 FCI/aug-cc-pVTZ	8.49 FCI/aug-cc-pVTZ	9.92 FCI/aug-cc-pVTZ	10.06 FCI/aug-cc-pVTZ	10.06 FCI/aug-cc-pVTZ
Cyclopropene	6.68 CCSDT/aug-cc-pVTZ	6.79 FCI/aug-cc-pVDZ + [CCSDT/aug-cc-pVTZ − CCSDT/aug-cc-pVDZ]			
Diazirine	4.09 CCSDTQ/aug-cc-pVDZ + [CCSDT/aug-cc-pVTZ	7.27 CCSDTQ/aug-cc-pVDZ + [CCSDT/aug-cc-pVTZ	7.44 CCSDTQ/aug-cc-pVDZ + [CCSDT/aug-cc-pVTZ	8.03 CCSDTQ/aug-cc-pVDZ + [CCSDT/aug-cc-pVTZ	

	− CCSDT/aug-cc-pVDZ]	− CCSDT/aug-cc-pVDZ]	− CCSDT/aug-cc-pVDZ]	− CCSDT/aug-cc-pVDZ]	
Diazomethane	3.14 FCI/aug-cc-pVTZ	5.54 FCI/aug-cc-pVTZ	5.90 FCI/aug-cc-pVTZ		
Difluorocarbene	5.09 FCI/aug-cc-pVTZ				
Formaldehyde	3.98 FCI/aug-cc-pVTZ	7.23 FCI/aug-cc-pVTZ	8.13 FCI/aug-cc-pVTZ	8.23 FCI/aug-cc-pVTZ	8.67 FCI/aug-cc-pVTZ
Formamide	5.65 FCI/aug-cc-pVDZ + [CCSDT/aug-cc-pVTZ − CCSDT/aug-cc-pVDZ]				
Formyl Fluoride	5.96 FCI/aug-cc-pVDZ + [CCSDT/aug-cc-pVTZ − CCSDT/aug-cc-pVDZ]				
Hydrogen Chloride	7.84 FCI/aug-cc-pVTZ	7.84 FCI/aug-cc-pVTZ			
Hydrogen Cyaphide	4.84 FCI/aug-cc-pVTZ	5.15 FCI/aug-cc-pVTZ	5.15 FCI/aug-cc-pVTZ		
Hydrogen Sulfide	6.18 FCI/aug-cc-pVTZ	6.24 FCI/aug-cc-pVTZ			
Imidazole	5.71 CCSDT/aug-cc-pVDZ + [CC3/aug-cc-pVTZ − CC3/aug-cc-pVDZ]	6.41 CCSDT/aug-cc-pVDZ + [CC3/aug-cc-pVTZ − CC3/aug-cc-pVDZ]	6.50 CCSDT/aug-cc-pVDZ + [CC3/aug-cc-pVTZ − CC3/aug-cc-pVDZ]		
Ketene	3.85 FCI/aug-cc-pVDZ + [CCSDT/aug-cc-pVTZ − CCSDT/aug-cc-pVDZ]	6.01 FCI/aug-cc-pVTZ	7.18 FCI/aug-cc-pVTZ	7.25 CCSDTQ/aug-cc-pVDZ + [CCSDT/aug-cc-pVTZ − CCSDT/aug-cc-pVDZ]	
Methanimine	5.23 FCI/aug-cc-pVTZ				
Nitrobenzene				5.39 CC-CBS/aug-cc-pVQZ	
Nitrosomethane	1.96	4.76	6.29		

	FCI/aug-cc-pVDZ + [CCSDT/aug-cc-pVTZ − CCSDT/aug-cc-pVDZ]	FCI/aug-cc-pVTZ	CCSDTQ/aug-cc-pVDZ + [CCSDT/aug-cc-pVTZ − CCSDT/aug-cc-pVDZ]		
Nitrosyl Hydride	1.74 FCI/aug-cc-pVTZ	4.33 FCI/aug-cc-pVTZ	6.27 CCSDTQ/aug-cc-pVDZ + [CCSDT/aug-cc-pVTZ − CCSDT/aug-cc-pVDZ]		
Propynal	3.84 CCSDT/aug-cc-pVTZ	5.54 CCSDT/aug-cc-pVTZ			
Pyridine	4.95 CCSDT/aug-cc-pVDZ + [CC3/aug-cc-pVTZ − CC3/aug-cc-pVDZ]	5.14 CCSDT/aug-cc-pVDZ + [CC3/aug-cc-pVTZ − CC3/aug-cc-pVDZ]	5.40 CCSDT/aug-cc-pVDZ + [CC3/aug-cc-pVTZ − CC3/aug-cc-pVDZ]	6.62 CCSDT/aug-cc-pVDZ + [CC3/aug-cc-pVTZ − CC3/aug-cc-pVDZ]	6.76 CCSDT/aug-cc-pVDZ + [CC3/aug-cc-pVTZ − CC3/aug-cc-pVDZ]
Pyrrole	5.24 CCSDT/aug-cc-pVTZ	6.00 CCSDT/aug-cc-pVTZ	6.00 CCSDT/aug-cc-pVDZ + [CC3/aug-cc-pVTZ − CC3/aug-cc-pVDZ]	6.26 CCSDT/aug-cc-pVTZ	6.30 CCSDT/aug-cc-pVTZ
Thiophene	5.64 CCSDT/aug-cc-pVTZ	5.98 CCSDT/aug-cc-pVTZ	6.14 CCSDT/aug-cc-pVTZ	6.14 CCSDT/aug-cc-pVTZ	6.21 CCSDT/aug-cc-pVTZ

Average Excitation Energy Error with Respect to Number of Determinants

a)

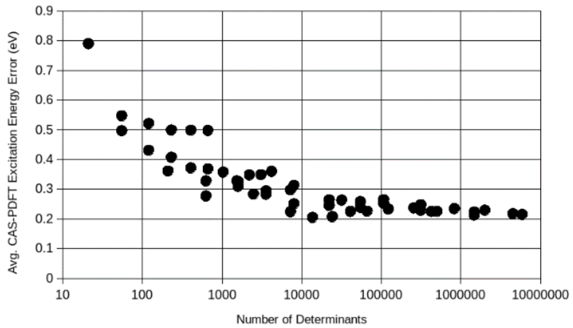

b)

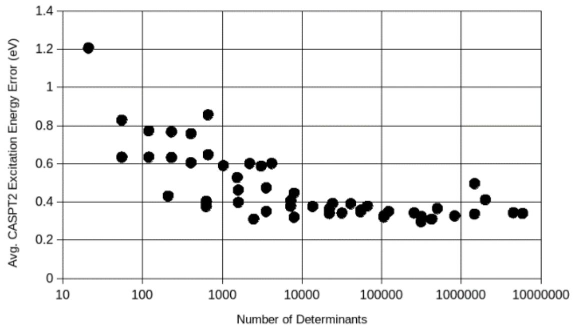

Figure S1: Average excitation energy error, per active space, for all excitations in our dataset (PASS+) as calculated using (a) CAS-PDFT and (b) CASPT2. Each active space is represented by the number of Slater determinants required to build it.

Active Spaces Chosen by GDM-AS with Experimental and DFT Reference Dipole Moments

Table S3: Active spaces chosen by GDM-AS for all molecules in the dataset, and the resulting CAS-PDFT and CASPT2 excitation energy errors. Experimental reference dipole moments are provided by NIST. DFT reference dipole moments are calculated with Gaussian.

Experimental

Molecule	Active Space	CAS-PDFT Excitation Energy Error (eV)					CASPT2 Excitation Energy Error (eV)				
		S_1	S_2	S_3	S_4	S_5	S_1	S_2	S_3	S_4	S_5
Carbon Monoxide	(12,13)	0.64	0.64	0.21	0.40	0.40	0.21	0.21	0.31	0.27	0.27
Formaldehyde	(6,7)	0.15	0.20	0.26	0.63	0.20	0.07	0.02	0.11	1.00	0.67
Pyridine	(14,14)	0.00	0.22	0.01	0.40	0.28	0.04	0.07	0.21	0.25	0.27
Pyrrole	(6,10)	0.33	0.45	0.36	0.35	0.20	0.18	0.27	0.33	0.15	0.95
Thiophene	(12,11)	0.32	0.06	0.12	0.18	0.44	0.20	0.40	0.25	0.42	0.52
Diazirine	(14,14)	0.08	0.05	0.21	0.29		0.00	0.01	0.03	0.21	
Ketene	(10,8)	0.59	0.39	0.18	2.26		0.10	0.27	0.22	1.95	
Diazomethane	(8,12)	0.35	0.48	0.39			0.08	0.17	0.06		
Hydrogen Cyaphide	(12,13)	0.09	0.12	0.12			0.13	0.03	0.04		
Imidazole	(10,14)	0.04	0.12	0.14			0.19	0.02	0.22		
Nitrosyl Hydride	(8,7)	0.05	0.03	0.00			0.09	0.17	0.21		
Nitrosomethane	(12,11)	0.04	0.05	0.01			0.13	0.10	0.05		
Cyclopropene	(12,14)	0.12	0.16				0.01	0.31			
Hydrogen Sulfide	(6,11)	0.28	0.21				0.00	0.09			
Propynal	(10,14)	0.29	0.02				0.22	0.17			
Acetaldehyde	(14,10)	0.07					0.01				
Difluorocarbene	(10,11)	1.26					0.16				
Formamide	(12,11)	0.43					0.12				
Formyl Fluoride	(14,13)	0.00					0.05				
Hydrogen Chloride	(8,14)	0.25	0.23				0.07	0.07			
Methanimine	(8,9)	0.04					0.08				
Aniline	(10,10)			0.25					0.22		
Azulene	(10,13)	0.34	0.08	0.47	0.49	0.52	0.07	0.09	0.73	0.86	0.84
Nitrobenzene	(8,13)				0.45					0.93	
Benzonitrile	(6,9)					0.60					1.23
Average		0.26	0.21	0.20	0.61	0.38	0.10	0.14	0.21	0.67	0.68

PBE0

Molecule	Active Space	CAS-PDFT Excitation Energy Error (eV)					CASPT2 Excitation Energy Error (eV)				
		S_1	S_2	S_3	S_4	S_5	S_1	S_2	S_3	S_4	S_5
Carbon Monoxide	(8,10)	0.64	0.64	0.17	0.37	0.37	0.20	0.20	0.28	0.27	0.27
Formaldehyde	(8,11)	0.03	0.28	0.36	0.70	0.62	0.04	0.08	0.03	1.13	0.80
Pyridine	(14,14)	0.00	0.22	0.01	0.40	0.28	0.04	0.07	0.21	0.25	0.27
Pyrrole	(8,14)	0.34	0.43	0.38	0.28	0.27	0.18	0.24	0.28	0.16	0.85
Thiophene	(6,6)	0.64	0.05	0.09	0.21	0.98	0.11	0.11	0.16	0.24	0.69
Diazirine	(10,11)	0.01	0.35	0.16	0.24		0.02	0.09	0.07	0.32	
Ketene	(8,11)	0.50	0.10	0.01	0.11		0.03	0.14	0.16	0.66	
Diazomethane	(10,9)	0.43	0.52	0.40			0.07	0.16	0.22		
Hydrogen Cyaphide	(12,13)	0.09	0.12	0.12			0.13	0.03	0.04		
Imidazole	(10,14)	0.04	0.12	0.14			0.19	0.02	0.22		
Nitrosyl Hydride	(10,14)	0.04	0.05	0.24			0.03	0.15	0.27		
Nitrosomethane	(10,11)	0.11	0.03	0.03			0.17	0.04	0.02		
Cyclopropene	(6,14)	0.18	0.31				0.61	0.88			
Hydrogen Sulfide	(6,6)	0.35	0.29				0.07	0.08			
Propynal	(8,14)	0.23	0.04				0.14	0.17			
Acetaldehyde	(8,11)	0.63					0.09				
Difluorocarbene	(12,14)	0.80					0.05				
Formamide	(8,8)	0.44					0.02				
Formyl Fluoride	(8,10)	0.00					0.09				
Hydrogen Chloride	(8,14)	0.25	0.23				0.07	0.07			
Methanimine	(8,9)	0.04					0.08				
Aniline	(8,11)			0.15					0.39		
Azulene	(12,12)	0.29	0.41	0.56	0.58	0.58	0.01	0.05	0.72	0.85	0.83
Nitrobenzene	(6,14)				0.58					1.08	
Benzonitrile	(6,8)					0.33					
Average		0.28	0.25	0.20	0.39	0.49	0.11	0.15	0.22	0.55	0.62

M06-2X

Molecule	Active Space	CAS-PDFT Excitation Energy Error (eV)					CASPT2 Excitation Energy Error (eV)				
		S_1	S_2	S_3	S_4	S_5	S_1	S_2	S_3	S_4	S_5
Carbon Monoxide	(10,14)	0.63	0.63	0.23	0.43	0.43	0.18	0.18	0.29	0.25	0.25
Formaldehyde	(6,8)	0.17	0.41	0.47	0.48	1.89	0.12	0.04	0.08	1.14	2.31
Pyridine	(14,14)	0.00	0.22	0.01	0.40	0.28	0.04	0.07	0.21	0.25	0.27
Pyrrole	(6,10)	0.33	0.45	0.36	0.35	0.20	0.18	0.27	0.33	0.15	0.95
Thiophene	(14,13)	0.26	0.06	0.06	0.06	0.24	0.05	0.37	0.22	0.35	0.60
Diazirine	(14,12)	0.10	0.06	0.23	0.27		0.01	0.07	0.08	0.29	
Ketene	(8,7)	0.41	0.49	0.47	2.30		0.10	0.25	0.29	2.00	
Diazomethane	(10,10)	0.47	0.50	0.39			0.11	0.13	0.10		
Hydrogen Cyaphide	(12,13)	0.09	0.12	0.12			0.13	0.03	0.04		
Imidazole	(10,14)	0.04	0.12	0.14			0.19	0.02	0.22		
Nitrosyl Hydride	(6,6)	0.04	0.07	0.05			0.10	0.12	0.23		
Nitrosomethane	(10,11)	0.11	0.03	0.03			0.17	0.04	0.02		
Cyclopropene	(6,14)	0.18	0.31				0.61	0.88			
Hydrogen Sulfide	(6,6)	0.35	0.29				0.07	0.08			
Propynal	(8,14)	0.23	0.04				0.14	0.17			
Acetaldehyde	(12,10)	0.18					0.04				
Difluorocarbene	(8,7)	1.19					0.15				
Formamide	(8,8)	0.44					0.02				
Formyl Fluoride	(12,13)	0.05					0.10				
Hydrogen Chloride	(8,14)	0.25	0.23				0.07	0.07			
Methanimine	(12,13)	0.25					0.08				
Aniline	(8,12)			0.26					0.30		
Azulene	(6,9)	0.37	0.14	0.46	0.48	0.50	0.13	0.21	0.71	0.86	0.86
Nitrobenzene	(6,14)				0.58					1.08	
Benzonitrile	(8,12)					0.32					0.96
Average		0.28	0.25	0.23	0.59	0.55	0.13	0.18	0.22	0.71	0.88

HSE06

Molecule	Active Space	CAS-PDFT Excitation Energy Error (eV)					CASPT2 Excitation Energy Error (eV)				
		S_1	S_2	S_3	S_4	S_5	S_1	S_2	S_3	S_4	S_5
Carbon Monoxide	(8,10)	0.64	0.64	0.17	0.37	0.37	0.20	0.20	0.28	0.27	0.27
Formaldehyde	(8,11)	0.03	0.28	0.36	0.70	0.62	0.04	0.08	0.03	1.13	0.80
Pyridine	(14,14)	0.00	0.22	0.01	0.40	0.28	0.04	0.07	0.21	0.25	0.27
Pyrrole	(8,14)	0.34	0.43	0.38	0.28	0.27	0.18	0.24	0.28	0.16	0.85
Thiophene	(6,6)	0.64	0.05	0.09	0.21	0.98	0.11	0.11	0.16	0.24	0.69
Diazirine	(10,11)	0.01	0.35	0.16	0.24		0.02	0.09	0.07	0.32	
Ketene	(8,11)	0.50	0.10	0.01	0.11		0.03	0.14	0.16	0.66	
Diazomethane	(10,9)	0.43	0.52	0.40			0.07	0.16	0.22		
Hydrogen Cyaphide	(12,13)	0.09	0.12	0.12			0.13	0.03	0.04		
Imidazole	(10,14)	0.04	0.12	0.14			0.19	0.02	0.22		
Nitrosyl Hydride	(10,14)	0.04	0.05	0.24			0.03	0.15	0.27		
Nitrosomethane	(10,11)	0.11	0.03	0.03			0.17	0.04	0.02		
Cyclopropene	(6,14)	0.18	0.31				0.61	0.88			
Hydrogen Sulfide	(6,6)	0.35	0.29				0.07	0.08			
Propynal	(8,14)	0.23	0.04				0.14	0.17			
Acetaldehyde	(8,11)	0.63					0.09				
Difluorocarbene	(12,14)	0.80					0.05				
Formamide	(8,8)	0.44					0.02				
Formyl Fluoride	(8,10)	0.00					0.09				
Hydrogen Chloride	(8,14)	0.25	0.23				0.07	0.07			
Methanimine	(8,9)	0.04					0.08				
Aniline	(8,11)			0.15					0.39		
Azulene	(12,11)	0.27	0.44	0.57	0.59	0.60	0.04	0.01	0.72	0.84	0.84
Nitrobenzene	(6,14)				0.58					1.08	
Benzonitrile	(6,8)					0.33					
Average		0.28	0.25	0.20	0.39	0.49	0.11	0.15	0.22	0.55	0.62

CAM-B3LYP

Molecule	Active Space	CAS-PDFT Excitation Energy Error (eV)					CASPT2 Excitation Energy Error (eV)				
		S_1	S_2	S_3	S_4	S_5	S_1	S_2	S_3	S_4	S_5
Carbon Monoxide	(10,12)	0.63	0.63	0.21	0.40	0.40	0.21	0.21	0.31	0.27	0.27
Formaldehyde	(6,8)	0.17	0.41	0.47	0.48	1.89	0.12	0.04	0.08	1.14	2.31
Pyridine	(14,14)	0.00	0.22	0.01	0.40	0.28	0.04	0.07	0.21	0.25	0.27
Pyrrole	(6,10)	0.33	0.45	0.36	0.35	0.20	0.18	0.27	0.33	0.15	0.95
Thiophene	(6,6)	0.64	0.05	0.09	0.21	0.98	0.11	0.11	0.16	0.24	0.69
Diazirine	(12,10)	0.26	0.11	0.18	0.11		0.04	0.10	0.06	0.25	
Ketene	(8,7)	0.41	0.49	0.47	2.30		0.10	0.25	0.29	2.00	
Diazomethane	(14,13)	0.31	0.21	0.54			0.06	0.05	0.38		
Hydrogen Cyaphide	(12,13)	0.09	0.12	0.12			0.13	0.03	0.04		
Imidazole	(10,13)	0.03	0.10	0.15			0.20	0.16	0.23		
Nitrosyl Hydride	(10,8)	0.08	0.03	0.04			0.12	0.11	0.44		
Nitrosomethane	(10,14)	0.06	0.04	0.01			0.12	0.10	0.01		
Cyclopropene	(6,14)	0.18	0.31				0.61	0.88			
Hydrogen Sulfide	(6,6)	0.35	0.29				0.07	0.08			
Propynal	(8,14)	0.23	0.04				0.14	0.17			
Acetaldehyde	(8,7)	0.03					0.02				
Difluorocarbene	(8,11)	0.95					0.00				
Formamide	(6,7)	0.48					0.01				
Formyl Fluoride	(12,13)	0.05					0.10				
Hydrogen Chloride	(6,11)	0.24	0.22				0.03	0.02			
Methanimine	(12,13)	0.25					0.08				
Aniline	(8,12)			0.26					0.30		
Azulene	(6,9)	0.37	0.14	0.46	0.48	0.50	0.13	0.21	0.71	0.86	0.86
Nitrobenzene	(6,14)				0.58					1.08	
Benzonitrile	(6,8)					0.33					
Average		0.28	0.23	0.24	0.59	0.66	0.12	0.17	0.25	0.69	0.89

LC-ωHPBE

Molecule	Active Space	CAS-PDFT Excitation Energy Error (eV)					CASPT2 Excitation Energy Error (eV)				
		S_1	S_2	S_3	S_4	S_5	S_1	S_2	S_3	S_4	S_5
Carbon Monoxide	(8,10)	0.64	0.64	0.17	0.37	0.37	0.20	0.20	0.28	0.27	0.27
Formaldehyde	(6,8)	0.17	0.41	0.47	0.48	1.89	0.12	0.04	0.08	1.14	2.31
Pyridine	(14,14)	0.00	0.22	0.01	0.40	0.28	0.04	0.07	0.21	0.25	0.27
Pyrrole	(8,14)	0.34	0.43	0.38	0.28	0.27	0.18	0.24	0.28	0.16	0.85
Thiophene	(12,11)	0.32	0.06	0.12	0.18	0.44	0.20	0.40	0.25	0.42	0.52
Diazirine	(12,10)	0.26	0.11	0.18	0.11		0.04	0.10	0.06	0.25	
Ketene	(8,11)	0.50	0.10	0.01	0.11		0.03	0.14	0.16	0.66	
Diazomethane	(8,11)	0.40	0.53	0.53			0.07	0.16	0.03		
Hydrogen Cyaphide	(12,13)	0.09	0.12	0.12			0.13	0.03	0.04		
Imidazole	(10,13)	0.03	0.10	0.15			0.20	0.16	0.23		
Nitrosyl Hydride	(6,12)	0.04	0.02	0.16			0.07	0.11	0.22		
Nitrosomethane	(10,14)	0.06	0.04	0.01			0.12	0.10	0.01		
Cyclopropene	(6,14)	0.18	0.31				0.61	0.88			
Hydrogen Sulfide	(6,12)	0.17	0.06				0.01	0.11			
Propynal	(8,14)	0.23	0.04				0.14	0.17			
Acetaldehyde	(8,7)	0.03					0.02				
Difluorocarbene	(12,13)	0.93					0.07				
Formamide	(6,7)	0.48					0.01				
Formyl Fluoride	(14,10)	0.11					0.11				
Hydrogen Chloride	(6,11)	0.24	0.22				0.03	0.02			
Methanimine	(8,14)	0.02					0.02				
Aniline	(8,12)			0.26					0.30		
Azulene	(10,8)	0.39	0.40	0.02	0.42	1.43	0.04	0.07	0.04	0.43	1.44
Nitrobenzene	(14,12)				0.23					0.31	
Benzonitrile	(6,8)					0.33					
Average		0.26	0.22	0.18	0.29	0.71	0.11	0.18	0.16	0.43	0.94

ωB97-xD

Molecule	Active Space	CAS-PDFT Excitation Energy Error (eV)					CASPT2 Excitation Energy Error (eV)				
		S_1	S_2	S_3	S_4	S_5	S_1	S_2	S_3	S_4	S_5
Carbon Monoxide	(12,12)	0.60	0.60	0.26	0.45	0.45	0.19	0.19	0.32	0.28	0.28
Formaldehyde	(10,12)	0.03	0.26	0.36	0.86	0.63	0.04	0.11	0.01	1.06	0.75
Pyridine	(14,14)	0.00	0.22	0.01	0.40	0.28	0.04	0.07	0.21	0.25	0.27
Pyrrole	(8,14)	0.34	0.43	0.38	0.28	0.27	0.18	0.24	0.28	0.16	0.85
Thiophene	(12,11)	0.32	0.06	0.12	0.18	0.44	0.20	0.40	0.25	0.42	0.52
Diazirine	(10,10)	0.06	0.32	0.17	0.14		0.01	0.11	0.10	0.32	
Ketene	(8,11)	0.50	0.10	0.01	0.11		0.03	0.14	0.16	0.66	
Diazomethane	(8,9)	0.53	0.61	0.48			0.10	0.18	0.15		
Hydrogen Cyaphide	(12,13)	0.09	0.12	0.12			0.13	0.03	0.04		
Imidazole	(10,13)	0.03	0.10	0.15			0.20	0.16	0.23		
Nitrosyl Hydride	(8,7)	0.05	0.03	0.00			0.09	0.17	0.21		
Nitrosomethane	(10,11)	0.11	0.03	0.03			0.17	0.04	0.02		
Cyclopropene	(6,14)	0.18	0.31				0.61	0.88			
Hydrogen Sulfide	(6,6)	0.35	0.29				0.07	0.08			
Propynal	(8,14)	0.23	0.04				0.14	0.17			
Acetaldehyde	(6,9)	0.44					0.07				
Difluorocarbene	(12,13)	0.93					0.07				
Formamide	(6,7)	0.48					0.01				
Formyl Fluoride	(10,11)	0.10					0.14				
Hydrogen Chloride	(6,11)	0.24	0.22				0.03	0.02			
Methanimine	(12,13)	0.25					0.08				
Aniline	(6,10)			0.26					0.32		
Azulene	(8,7)	0.38	0.37	0.00	0.40	1.47	0.06	0.10	0.03	0.42	1.47
Nitrobenzene	(14,12)				0.23					0.31	
Benzonitrile	(6,8)					0.33					
Average		0.28	0.24	0.17	0.34	0.55	0.12	0.18	0.17	0.43	0.69

M06-HF

Molecule	Active Space	CAS-PDFT Excitation Energy Error (eV)					CASPT2 Excitation Energy Error (eV)				
		S_1	S_2	S_3	S_4	S_5	S_1	S_2	S_3	S_4	S_5
Carbon Monoxide	(8,14)	0.62	0.62	0.11	0.33	0.36	0.28	0.28	0.25	0.25	0.25
Formaldehyde	(6,12)	0.08	0.03	0.11	0.82	0.59	0.18	0.20	0.32	1.18	0.91
Pyridine	(10,10)	0.32	0.24	0.72	0.51	0.40	0.23	0.10	0.10	0.34	0.22
Pyrrole	(6,10)	0.33	0.45	0.36	0.35	0.20	0.18	0.27	0.33	0.15	0.95
Thiophene	(6,6)	0.64	0.05	0.09	0.21	0.98	0.11	0.11	0.16	0.24	0.69
Diazirine	(8,10)	0.06	0.32	0.31	0.32		0.02	0.07	0.08	0.24	
Ketene	(6,10)	0.30	0.12	0.05	0.05		0.09	0.16	0.22	0.48	
Diazomethane	(12,12)	0.26	0.42	0.46			0.08	0.05	0.26		
Hydrogen Cyaphide	(12,13)	0.09	0.12	0.12			0.13	0.03	0.04		
Imidazole	(10,13)	0.03	0.10	0.15			0.20	0.16	0.23		
Nitrosyl Hydride	(8,9)	0.10	0.04	0.06			0.08	0.12	0.31		
Nitrosomethane	(12,10)	0.10	0.07	0.04			0.17	0.01	0.23		
Cyclopropene	(12,14)	0.12	0.16				0.01	0.31			
Hydrogen Sulfide	(6,7)	0.27	0.21				0.06	0.08			
Propynal	(8,14)	0.23	0.04				0.14	0.17			
Acetaldehyde	(8,8)	0.16					0.05				
Difluorocarbene	(6,14)	0.87					0.02				
Formamide	(10,14)	0.30					0.02				
Formyl Fluoride	(6,9)	0.12					0.17				
Hydrogen Chloride	(6,11)	0.24	0.22				0.03	0.02			
Methanimine	(8,8)	0.04					0.08				
Aniline	(10,10)			0.25					0.22		
Azulene	(10,8)	0.39	0.40	0.02	0.42	1.43	0.04	0.07	0.04	0.43	1.44
Nitrobenzene	(6,6)				2.00					1.97	
Benzonitrile	(8,14)					0.45					0.81
Average		0.26	0.21	0.20	0.56	0.63	0.11	0.13	0.20	0.59	0.75

Plots of CAS-PDFT Excitation Energy Error w.r.t. S_0 CASSCF Dipole Moment Error at All Active Spaces; Application of GDM-AS

All active space sizes considered in this work (PASS+) are shown in the plots although only active spaces in PASS will be chosen by GDM-AS. Active spaces chosen by GDM-AS are labeled with green crosses. Experimental dipole moments are used to provide reference data.

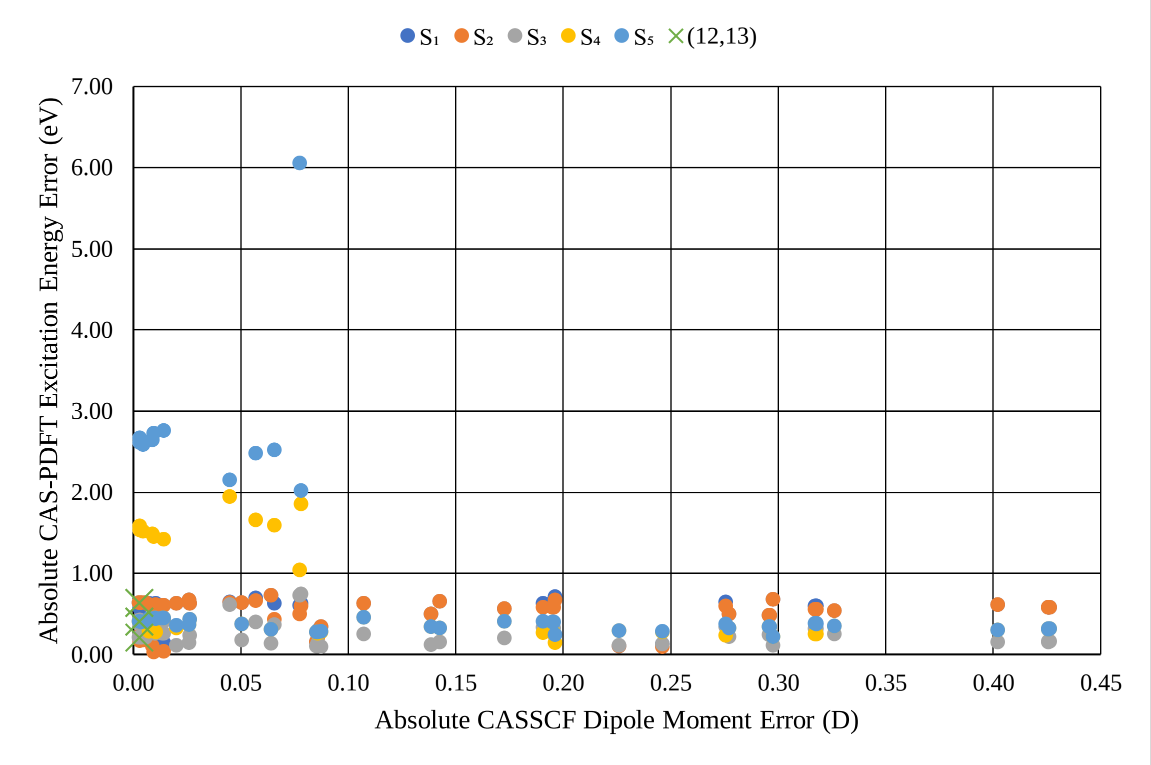

Figure S2: Plot of CAS-PDFT excitation energy error with respect to ground-state CASSCF dipole moment error for carbon monoxide.

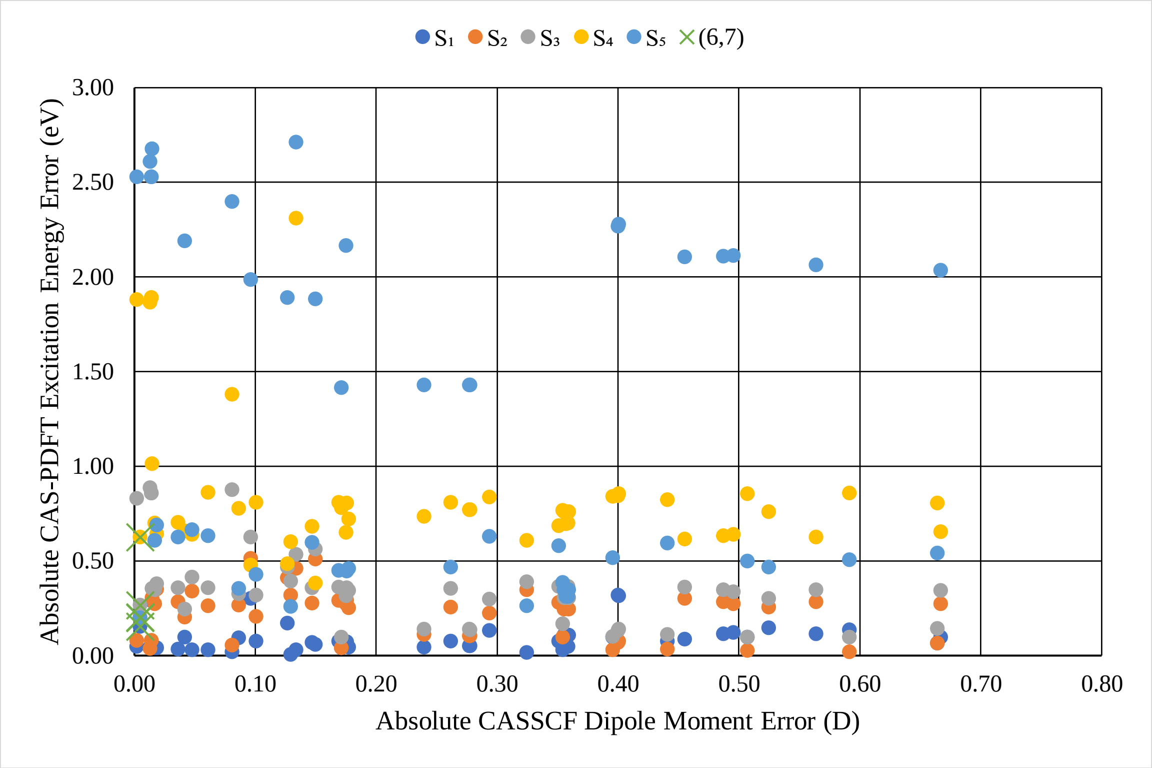

Figure S3: Plot of CAS-PDFT excitation energy error with respect to ground-state CASSCF dipole moment error for formaldehyde.

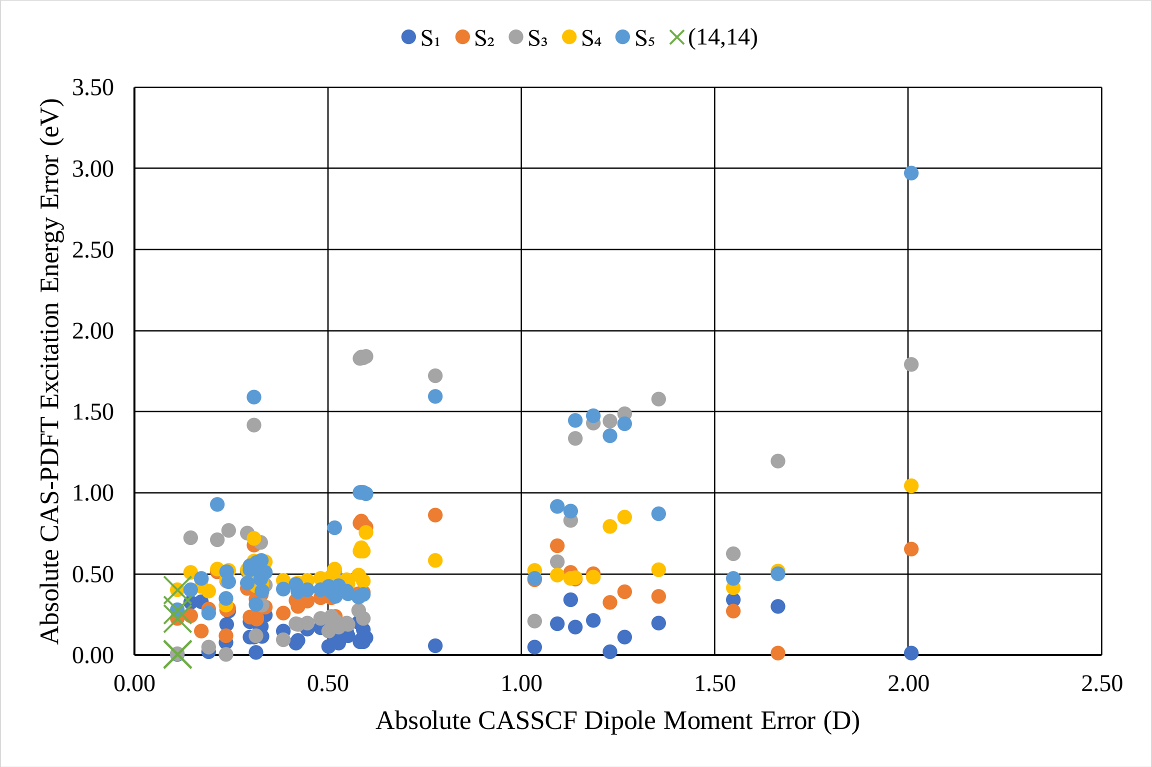

Figure S4: Plot of CAS-PDFT excitation energy error with respect to ground-state CASSCF dipole moment error for pyridine.

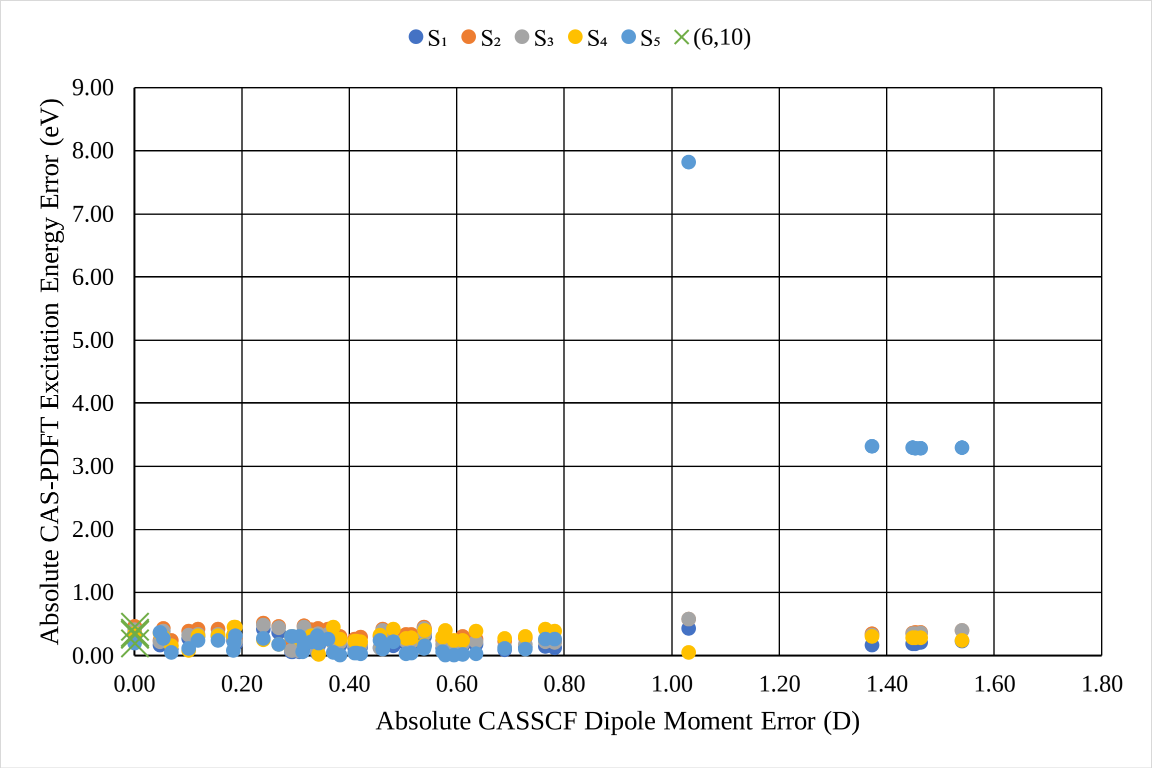

Figure S5: Plot of CAS-PDFT excitation energy error with respect to ground-state CASSCF dipole moment error for pyrrole.

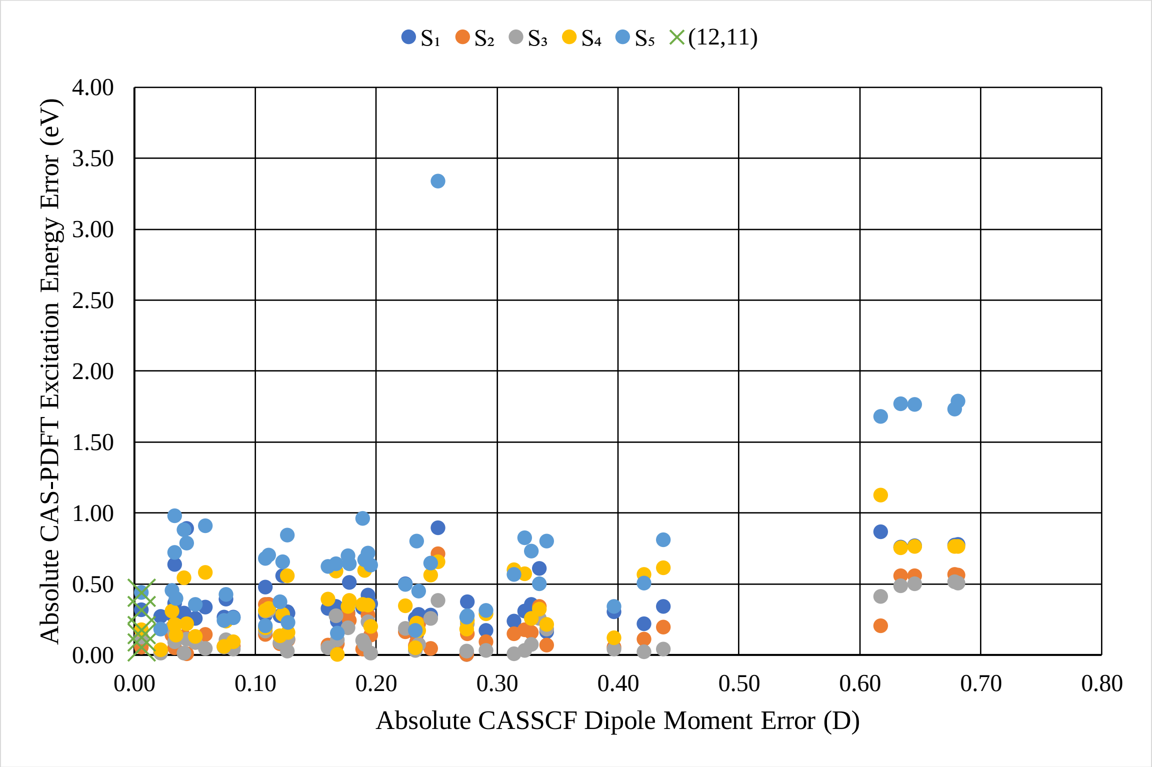

Figure S6: Plot of CAS-PDFT excitation energy error with respect to ground-state CASSCF dipole moment error for thiophene.

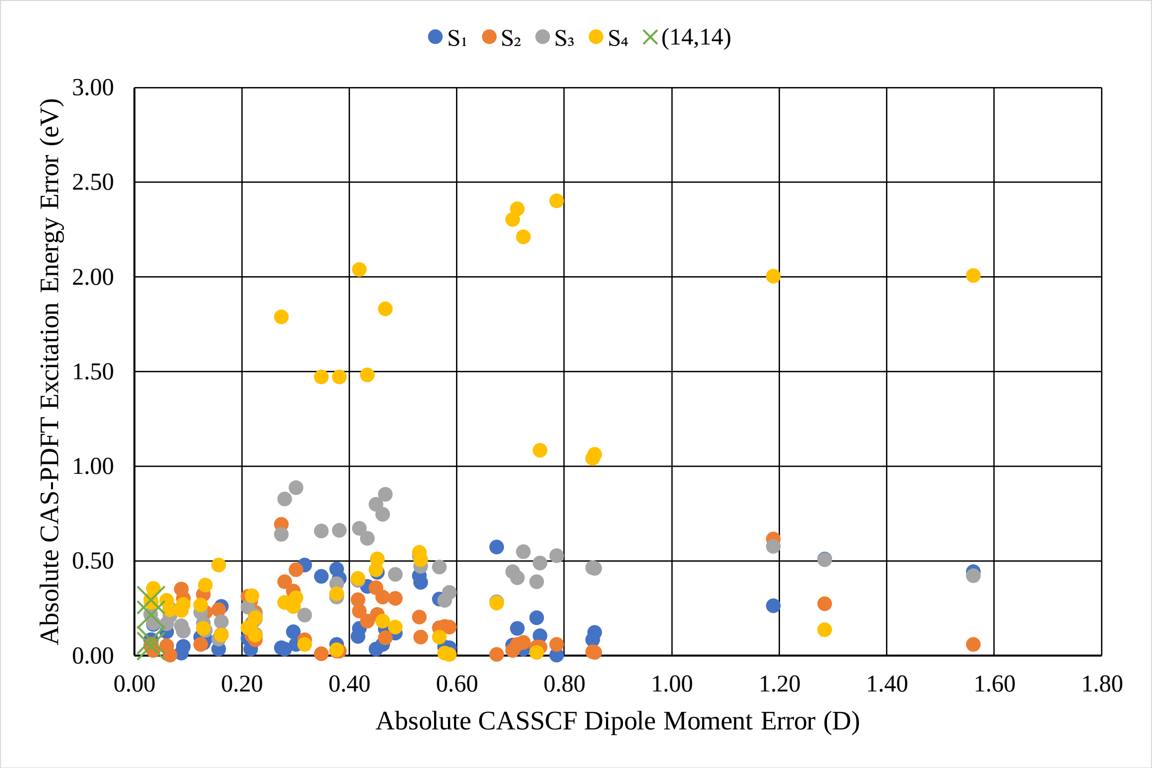

Figure S7: Plot of CAS-PDFT excitation energy error with respect to ground-state CASSCF dipole moment error for diazirine.

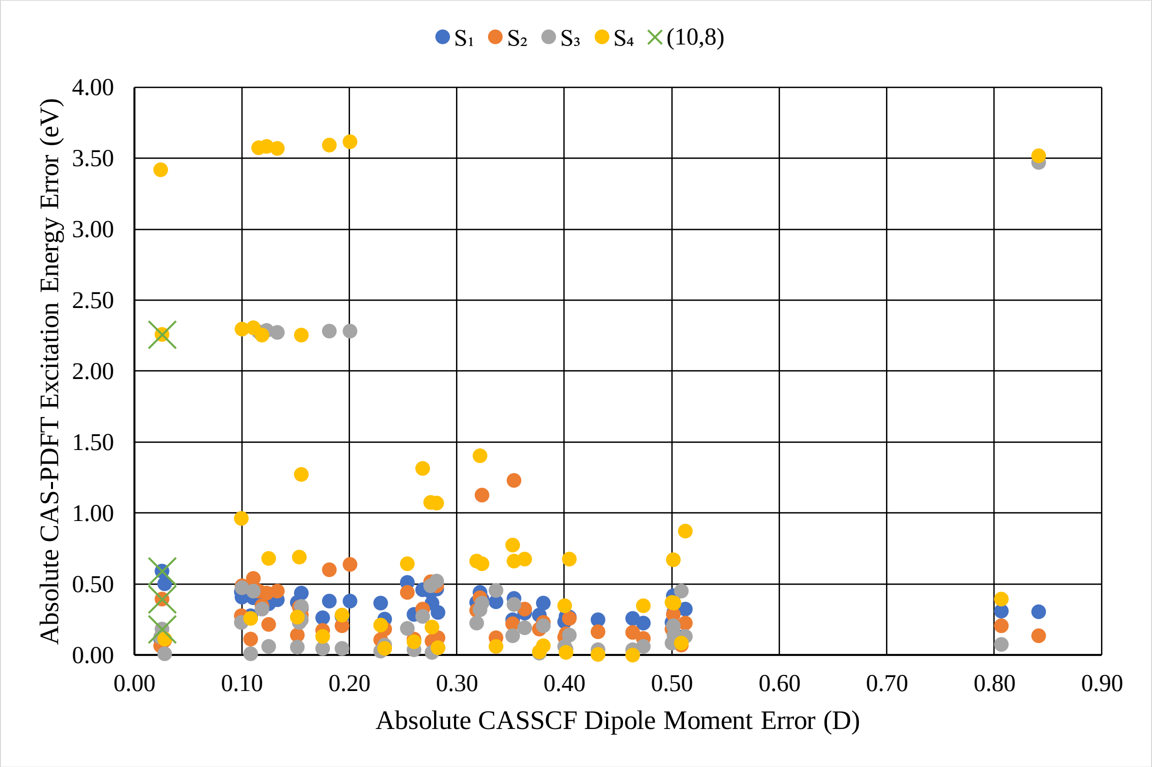

Figure S8: Plot of CAS-PDFT excitation energy error with respect to ground-state CASSCF dipole moment error for ketene.

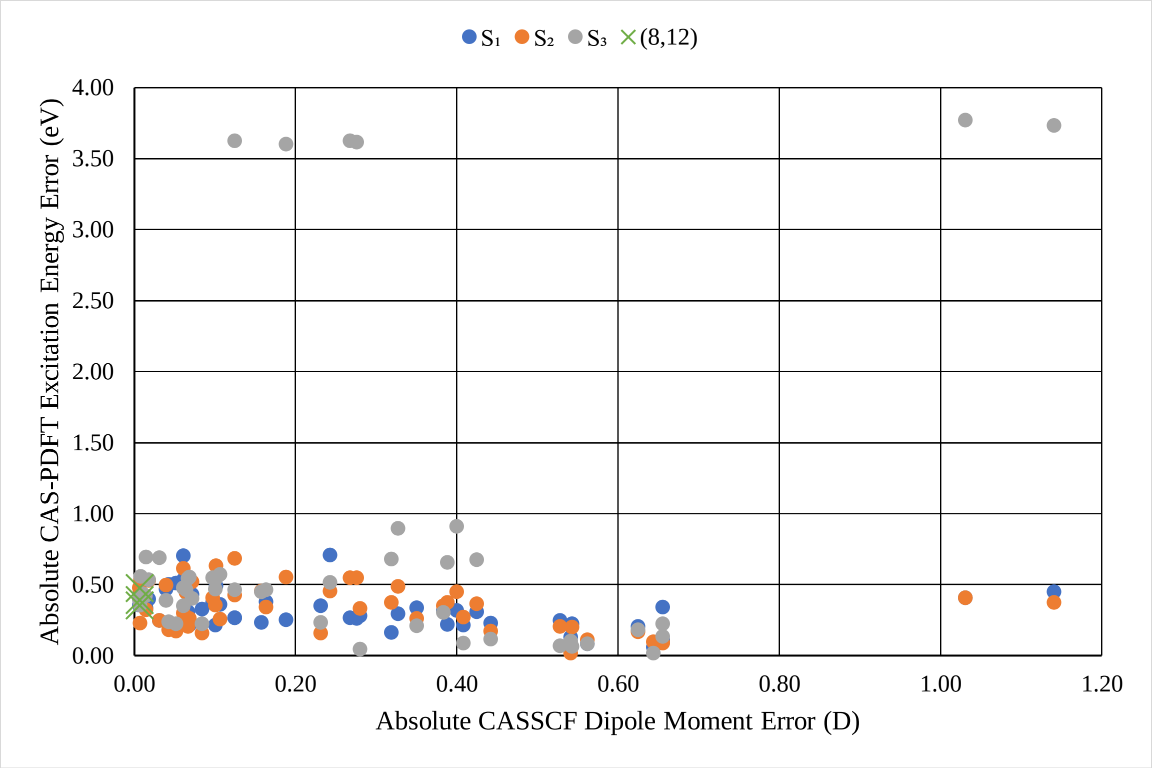

Figure S9: Plot of CAS-PDFT excitation energy error with respect to ground-state CASSCF dipole moment error for diazomethane.

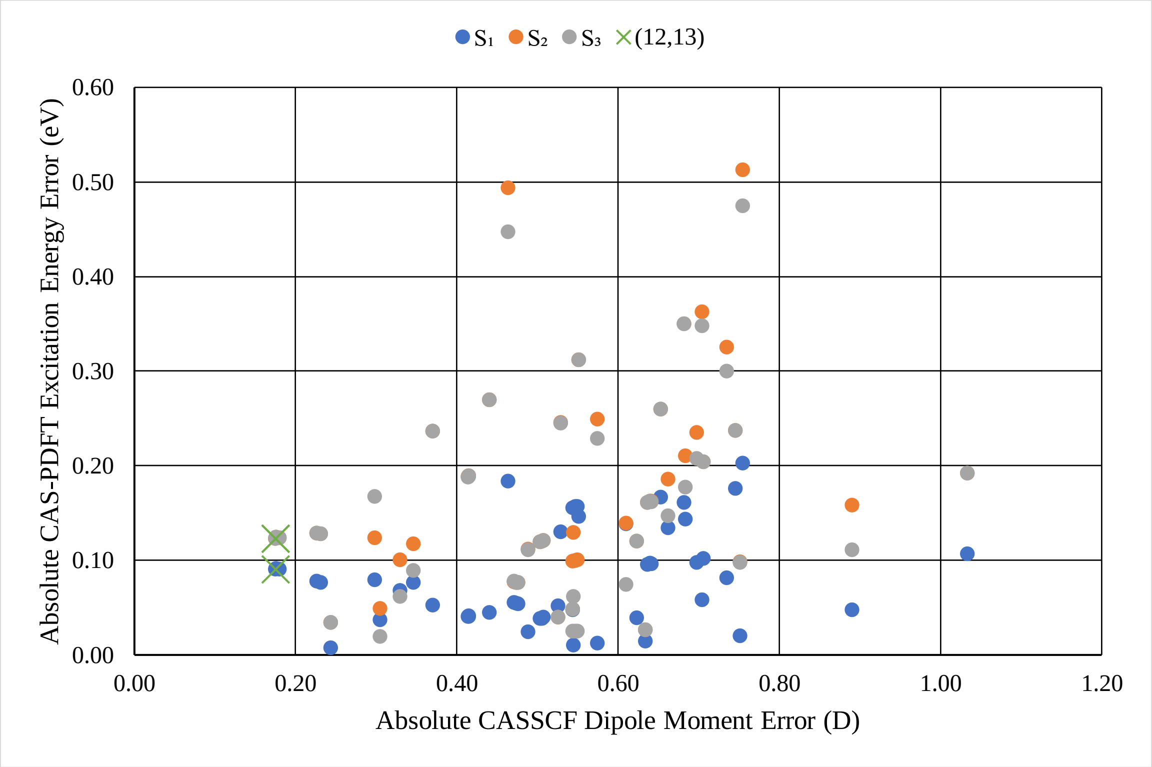

Figure S10: Plot of CAS-PDFT excitation energy error with respect to ground-state CASSCF dipole moment error for hydrogen cyaphide.

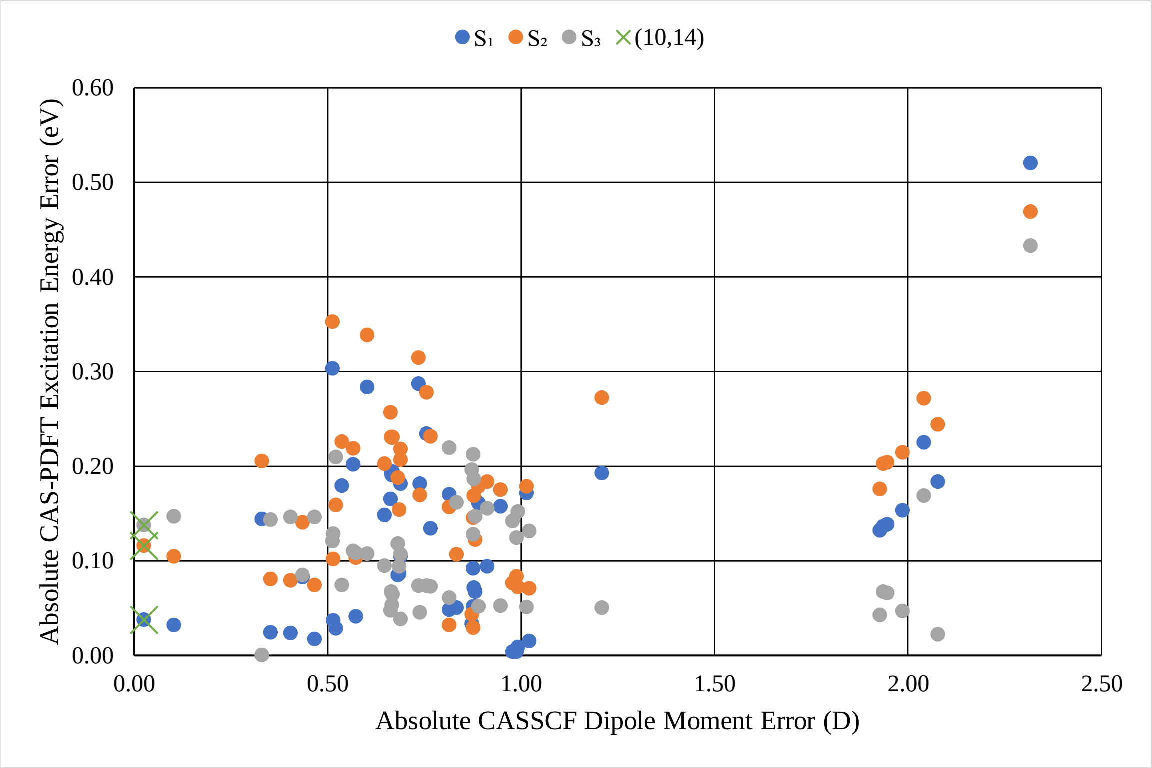

Figure S11: Plot of CAS-PDFT excitation energy error with respect to ground-state CASSCF dipole moment error for imidazole.

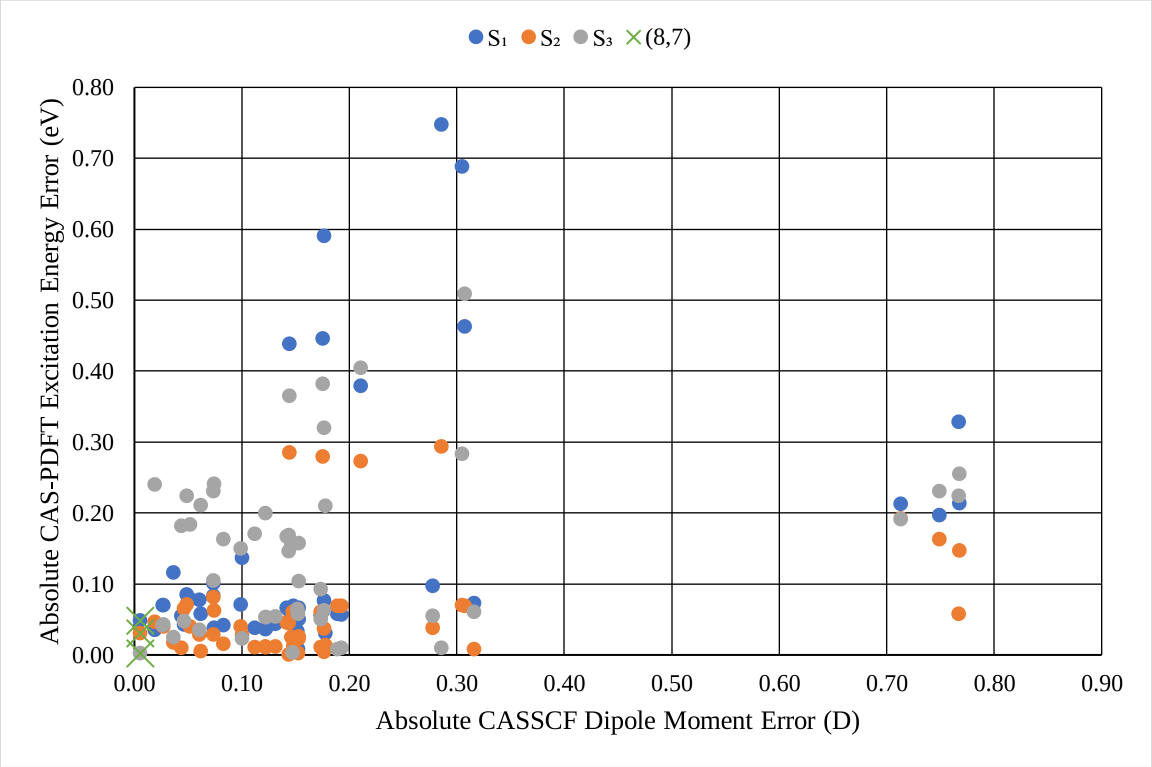

Figure S12: Plot of CAS-PDFT excitation energy error with respect to ground-state CASSCF dipole moment error for nitrosyl hydride.

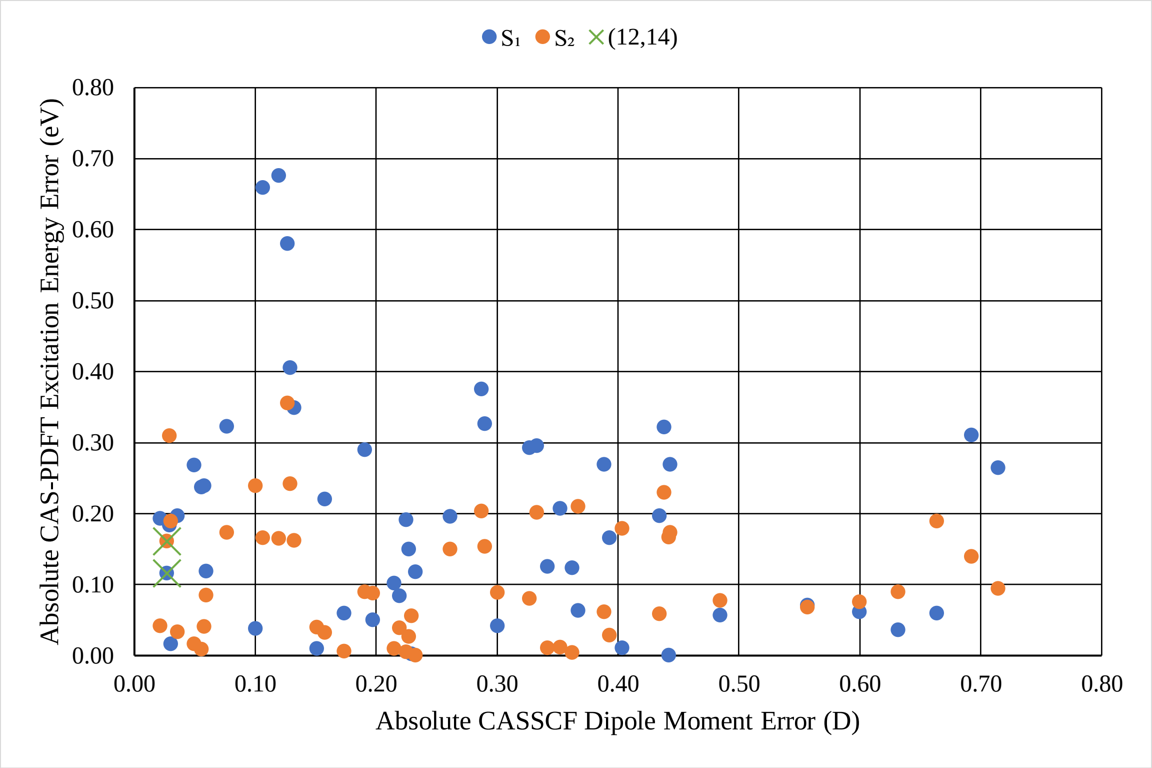

Figure S13: Plot of CAS-PDFT excitation energy error with respect to ground-state CASSCF dipole moment error for cyclopropene.

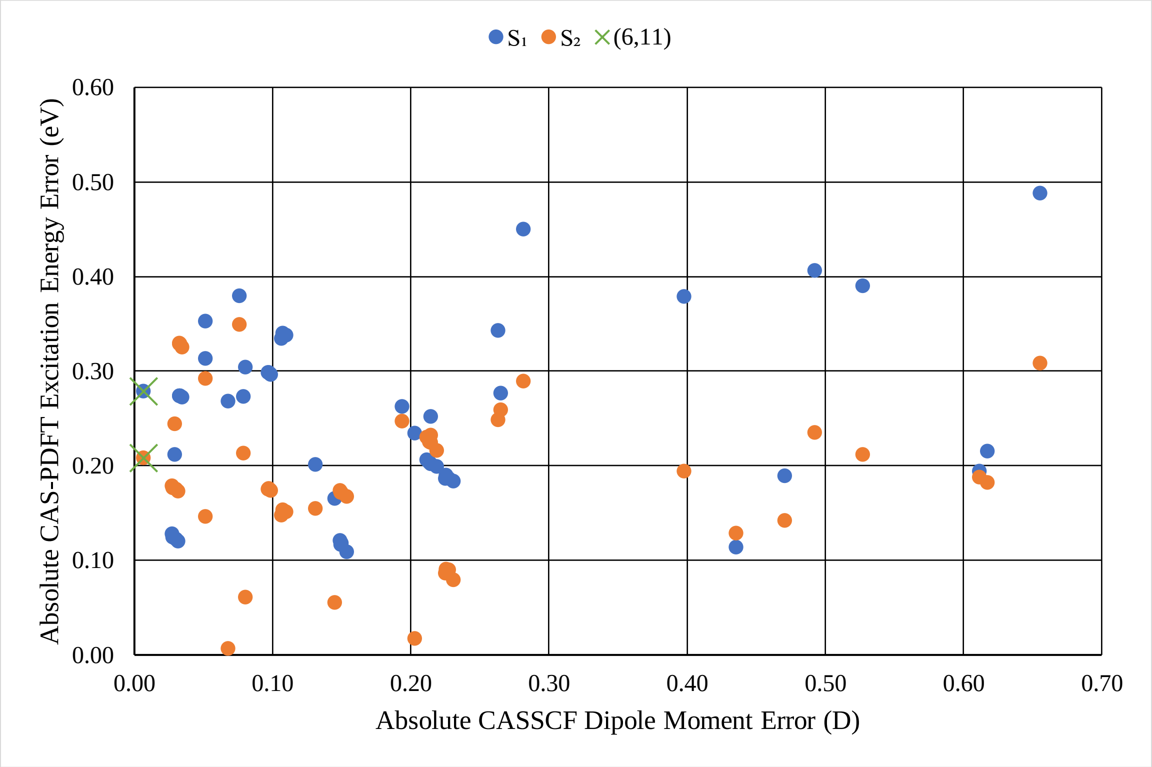

Figure S14: Plot of CAS-PDFT excitation energy error with respect to ground-state CASSCF dipole moment error for hydrogen sulfide.

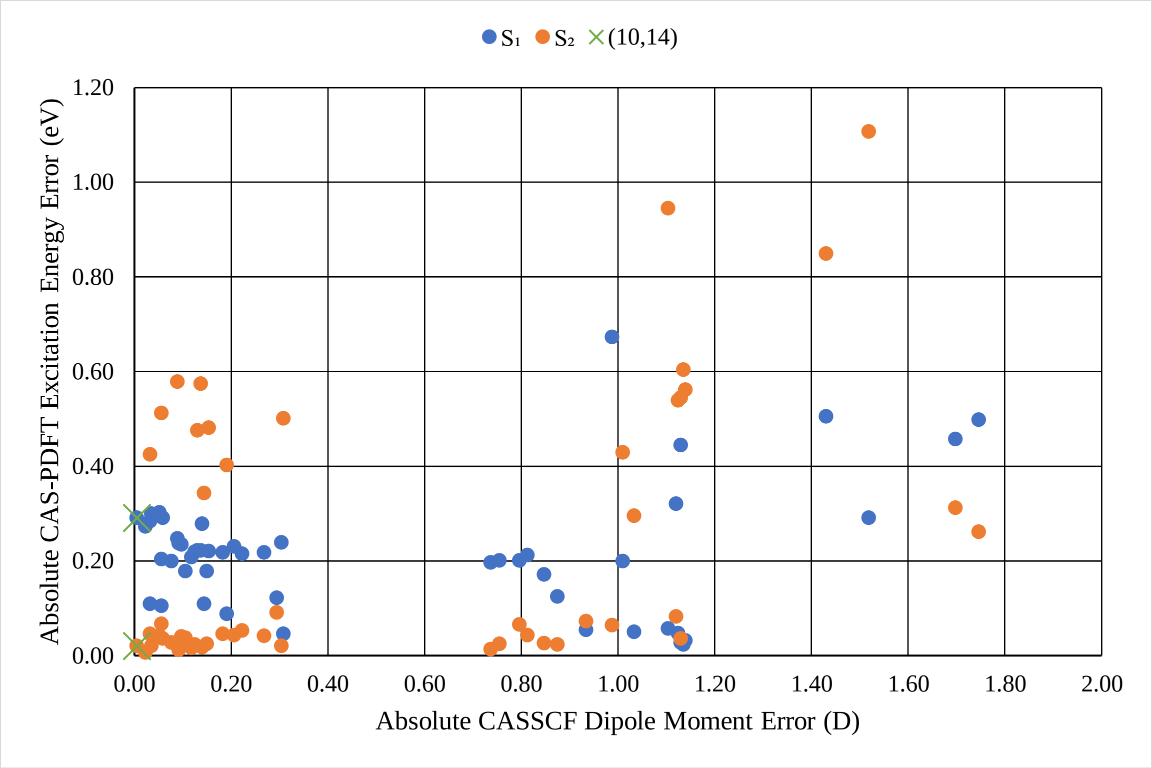

Figure S15: Plot of CAS-PDFT excitation energy error with respect to ground-state CASSCF dipole moment error for propynal.

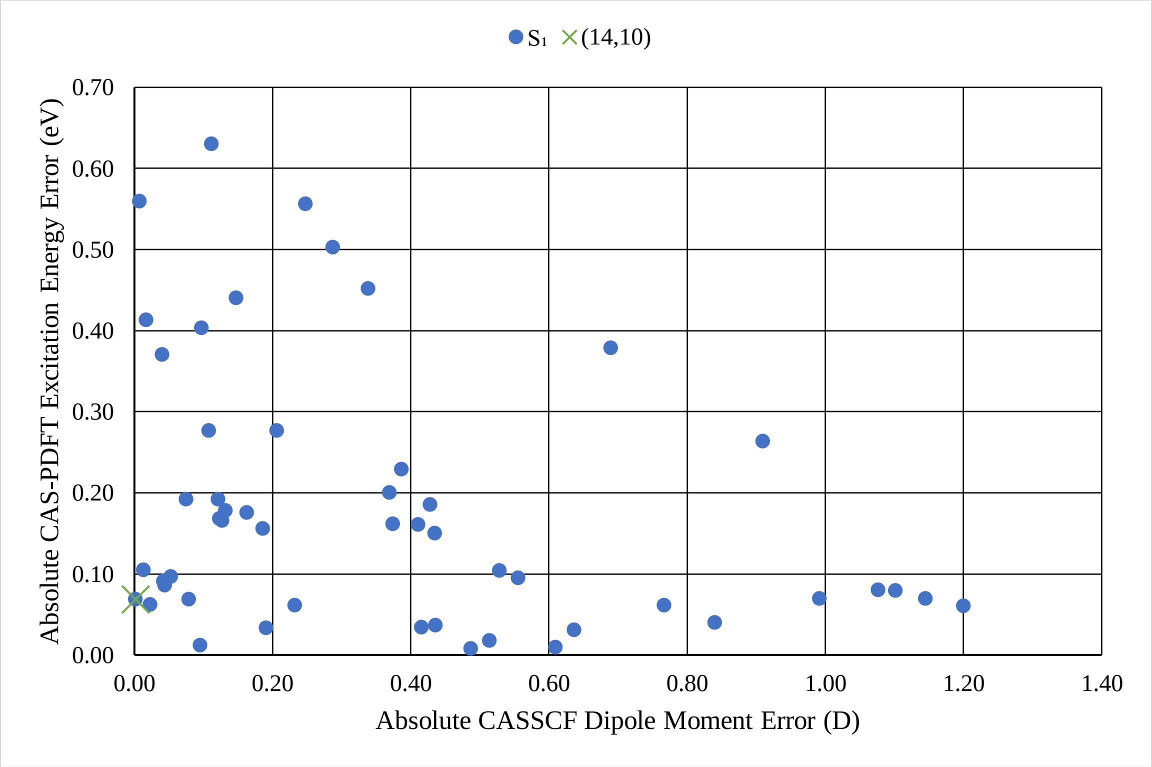

Figure S16: Plot of CAS-PDFT excitation energy error with respect to ground-state CASSCF dipole moment error for acetaldehyde.

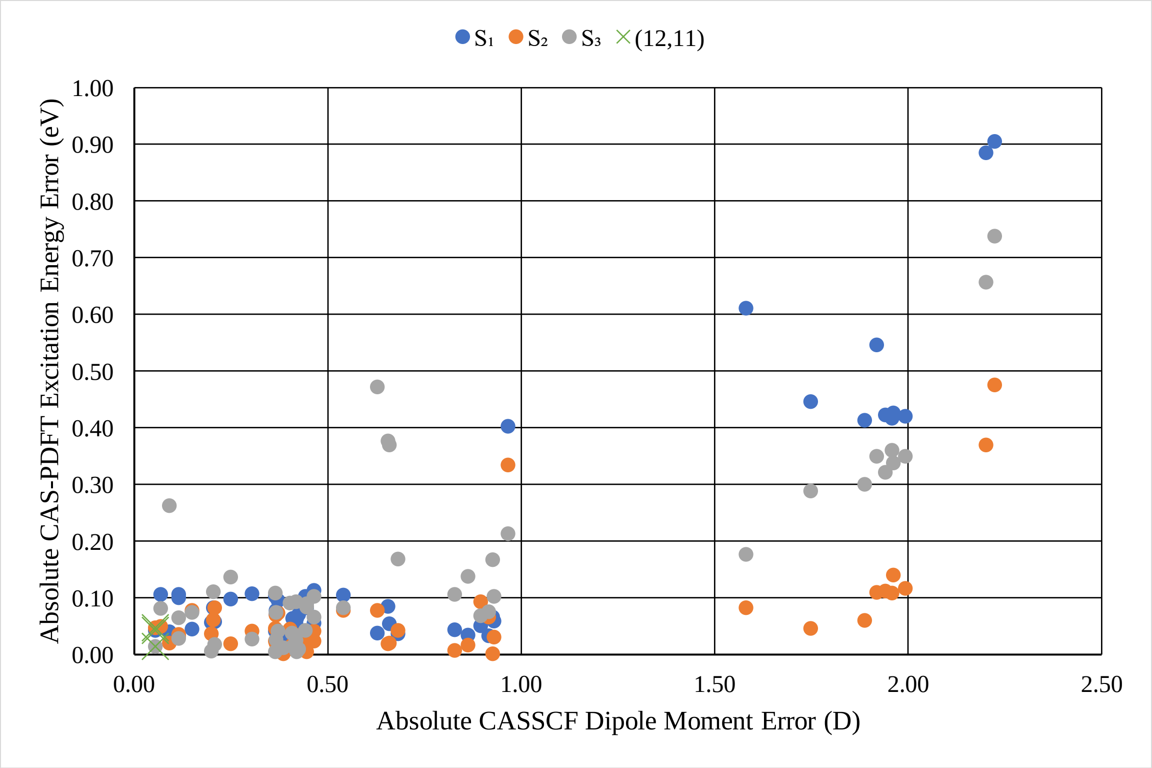

Figure S17: Plot of CAS-PDFT excitation energy error with respect to ground-state CASSCF dipole moment error for nitrosomethane.

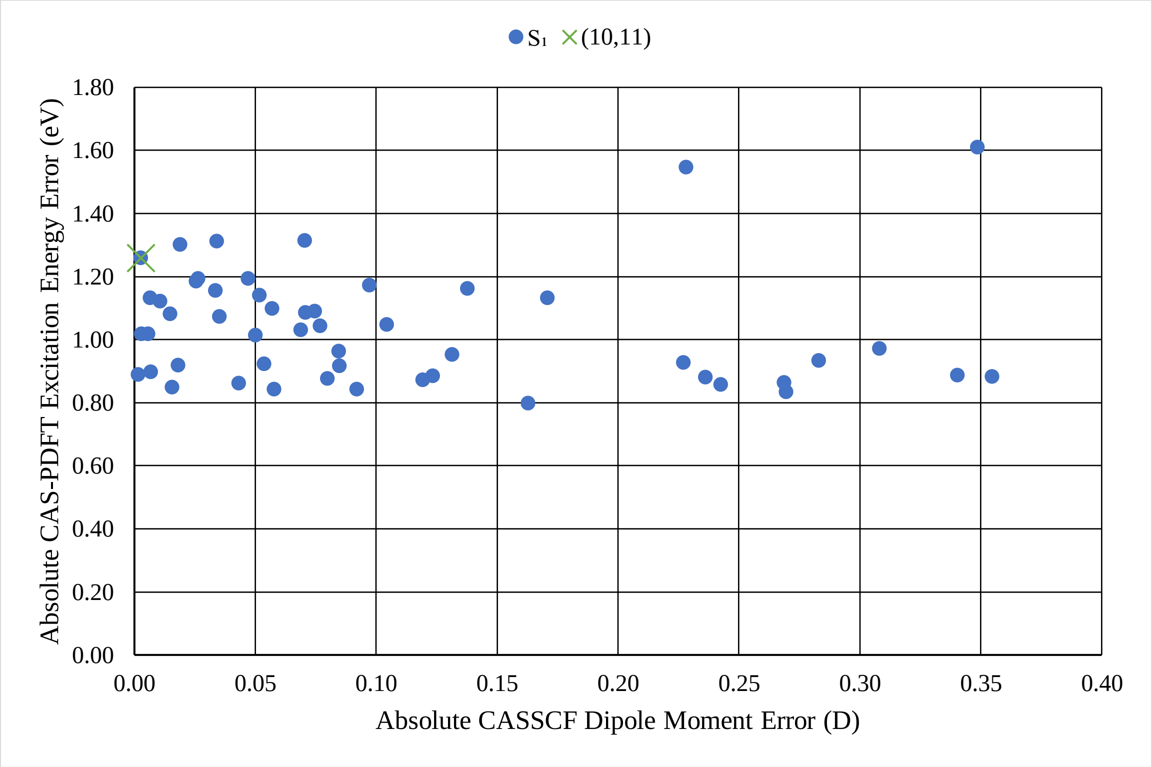

Figure S18: Plot of CAS-PDFT excitation energy error with respect to ground-state CASSCF dipole moment error for difluorocarbene.

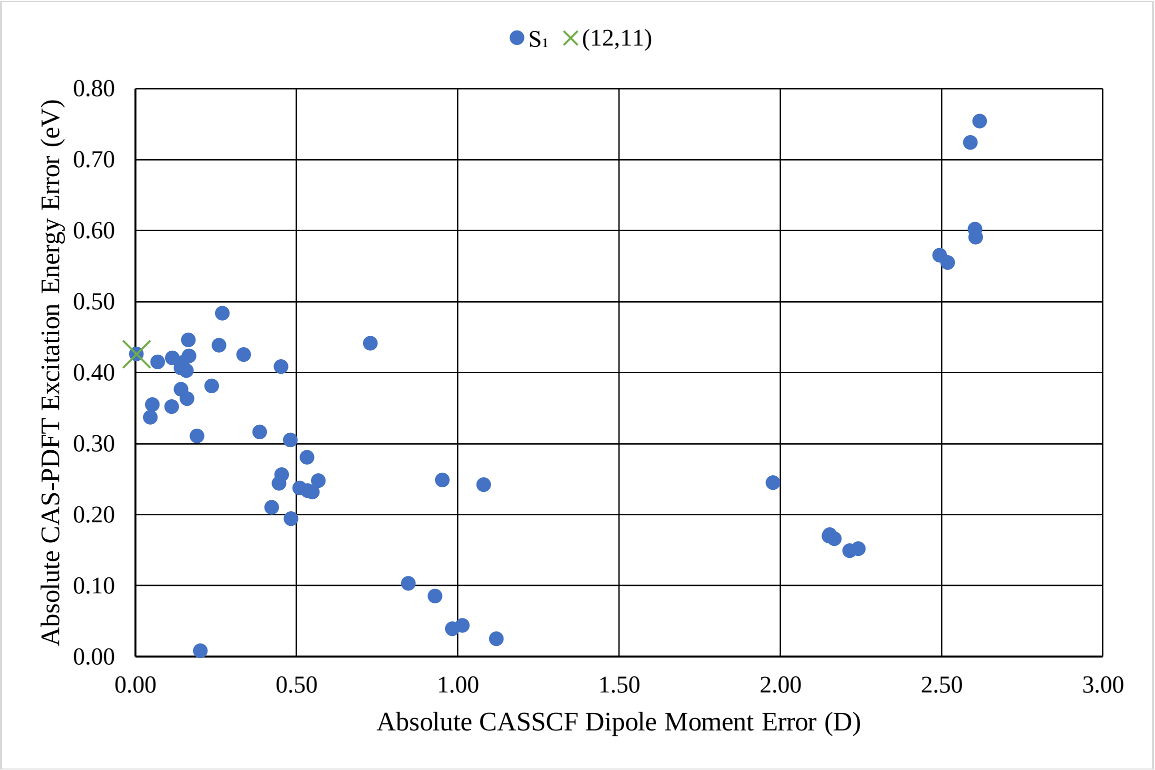

Figure S19: Plot of CAS-PDFT excitation energy error with respect to ground-state CASSCF dipole moment error for formamide.

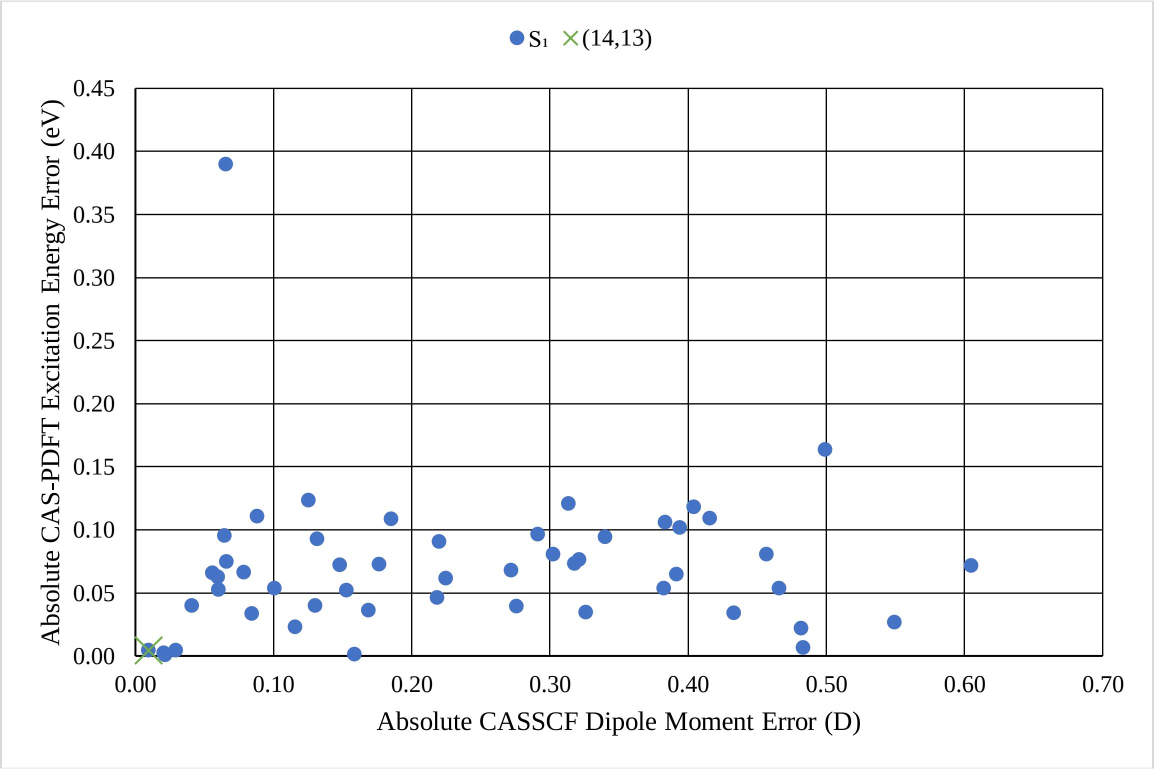

Figure S20: Plot of CAS-PDFT excitation energy error with respect to ground-state CASSCF dipole moment error for formyl fluoride.

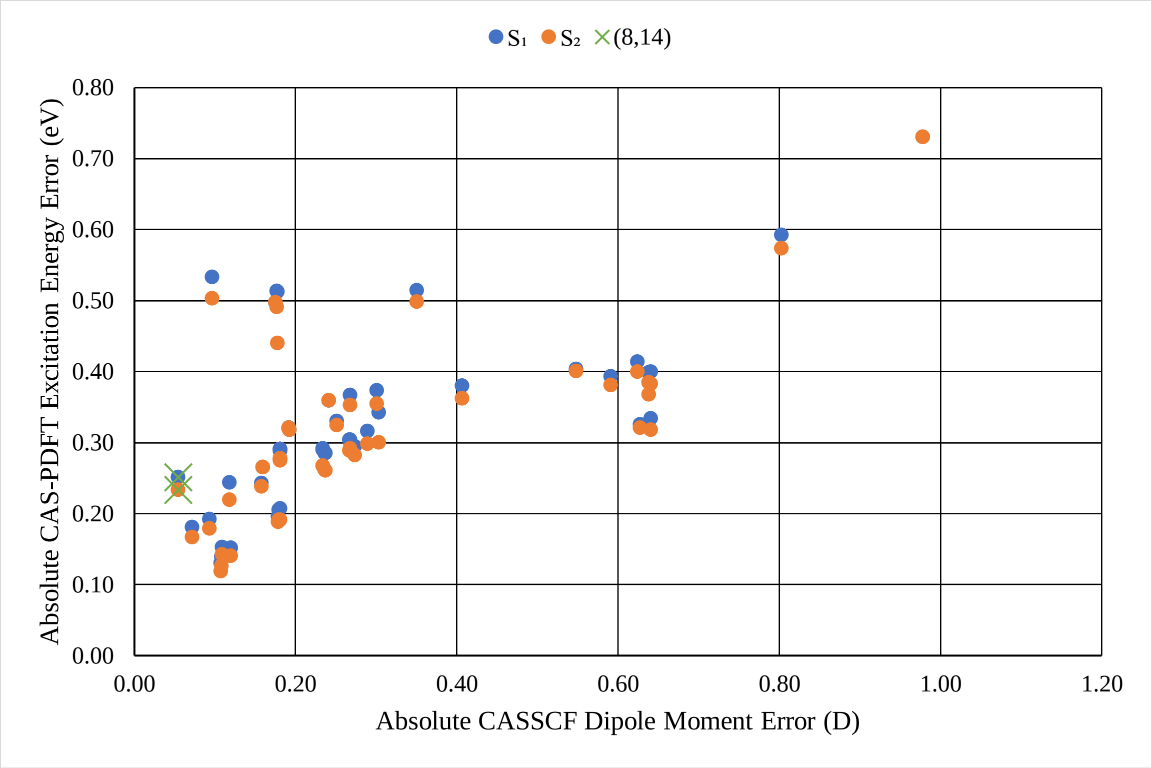

Figure S21: Plot of CAS-PDFT excitation energy error with respect to ground-state CASSCF dipole moment error for hydrogen chloride.

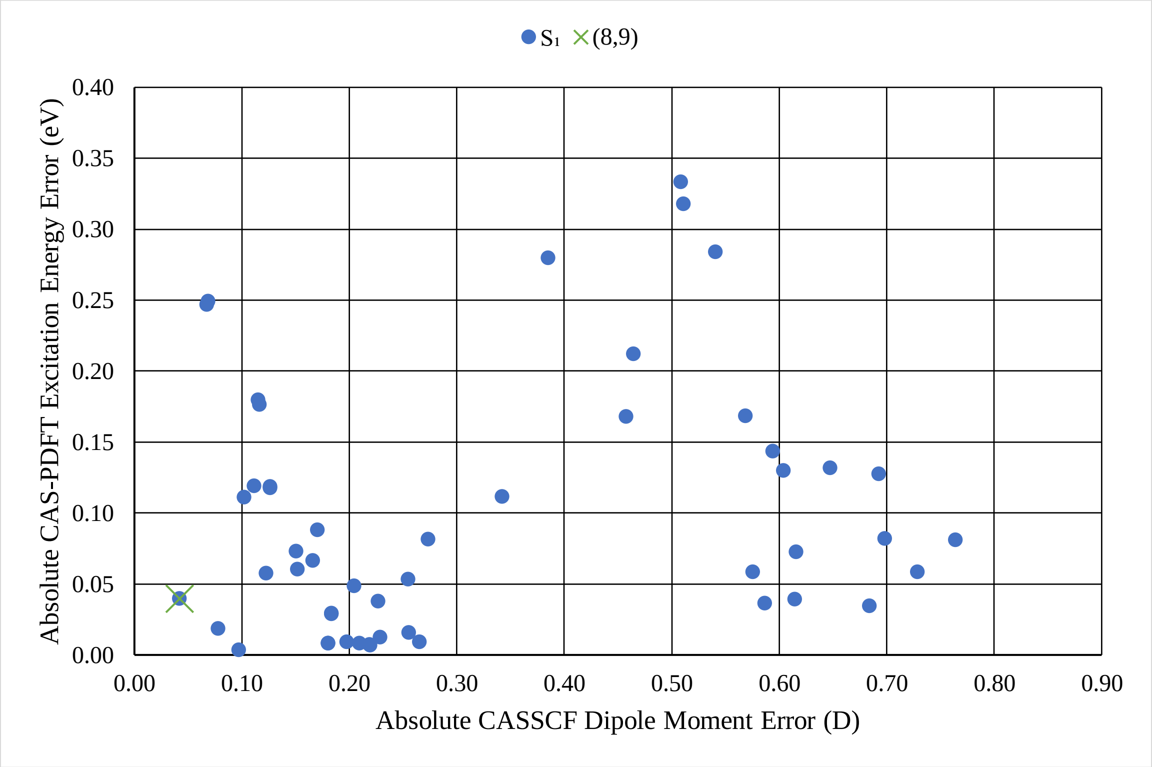

Figure S22: Plot of CAS-PDFT excitation energy error with respect to ground-state CASSCF dipole moment error for methanimine.

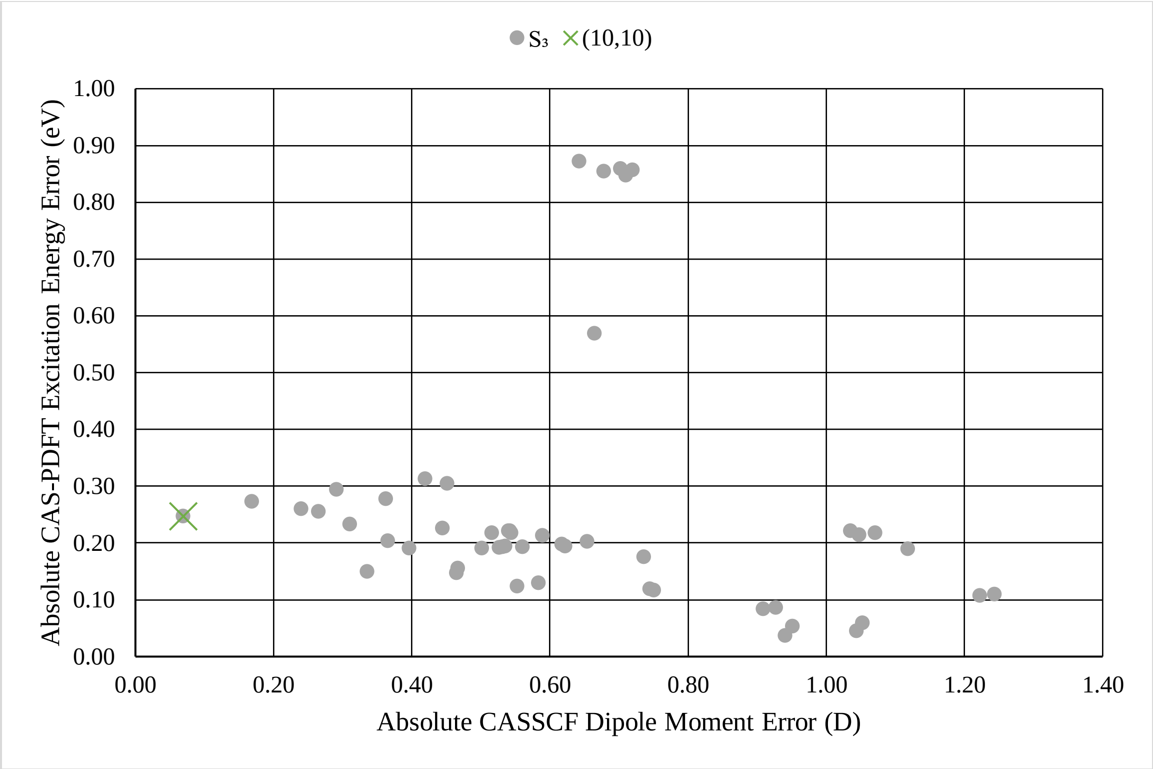

Figure S23: Plot of CAS-PDFT excitation energy error with respect to ground-state CASSCF dipole moment error for aniline.

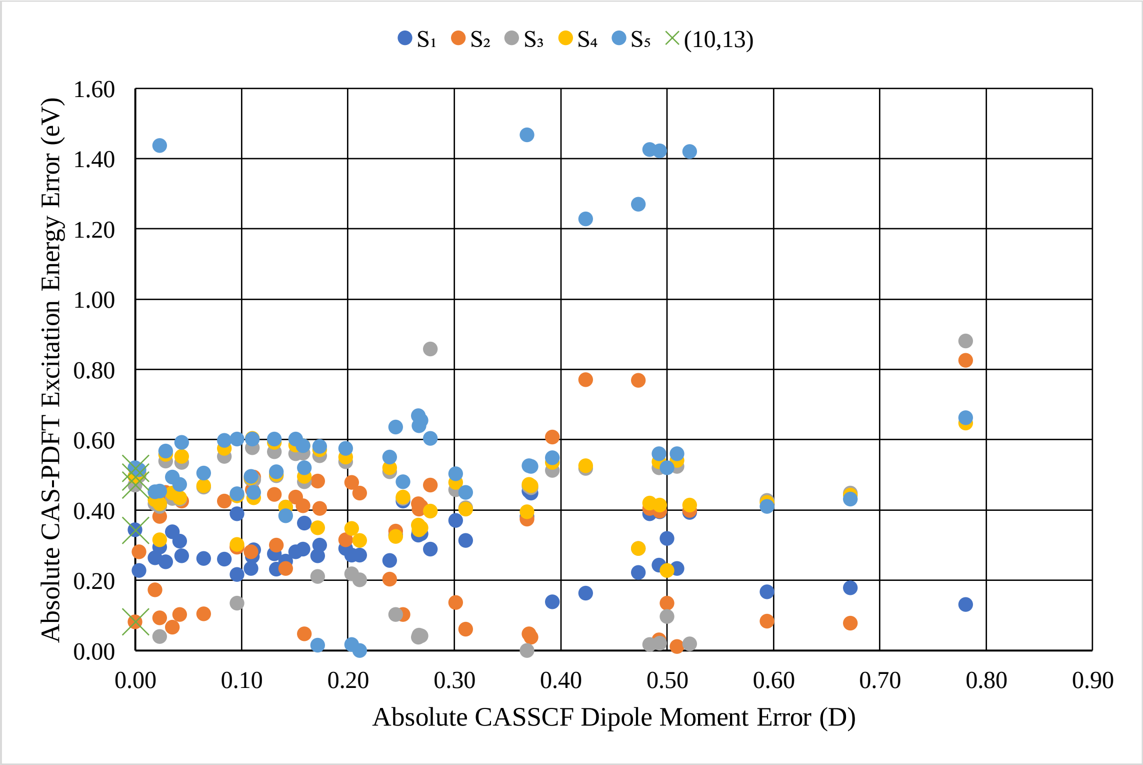

Figure S24: Plot of CAS-PDFT excitation energy error with respect to ground-state CASSCF dipole moment error for azulene.

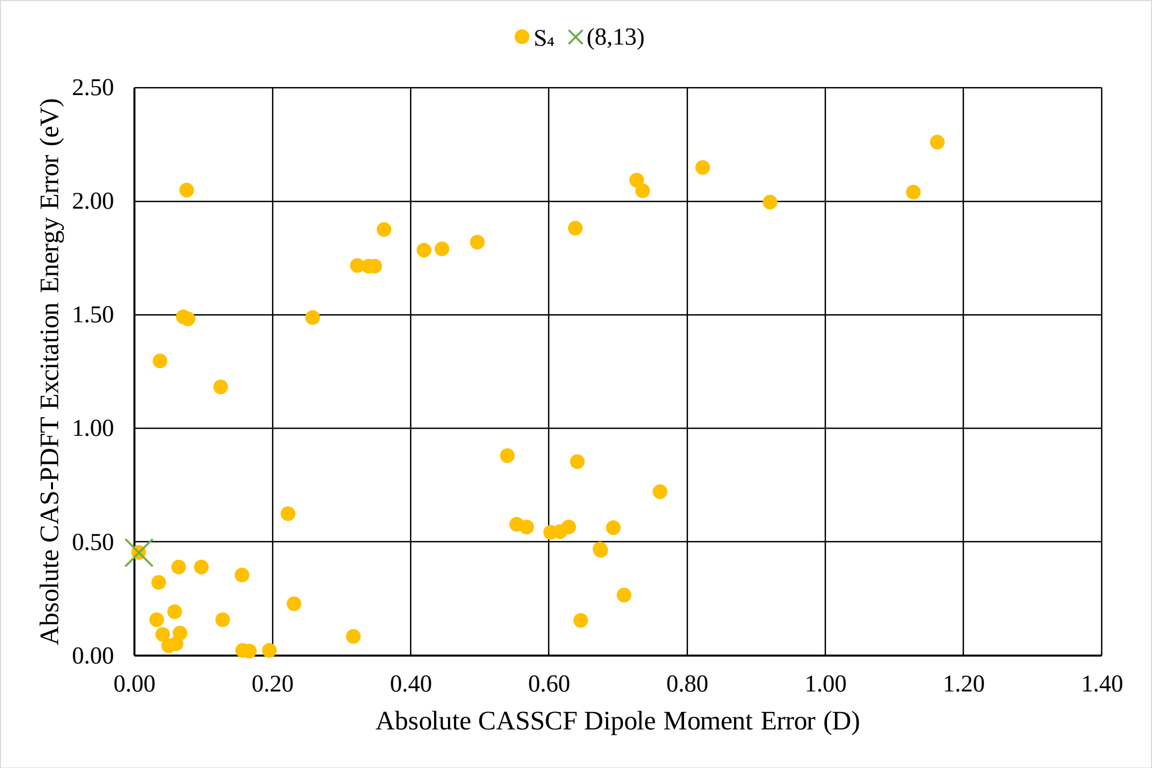

Figure S25: Plot of CAS-PDFT excitation energy error with respect to ground-state CASSCF dipole moment error for nitrobenzene.

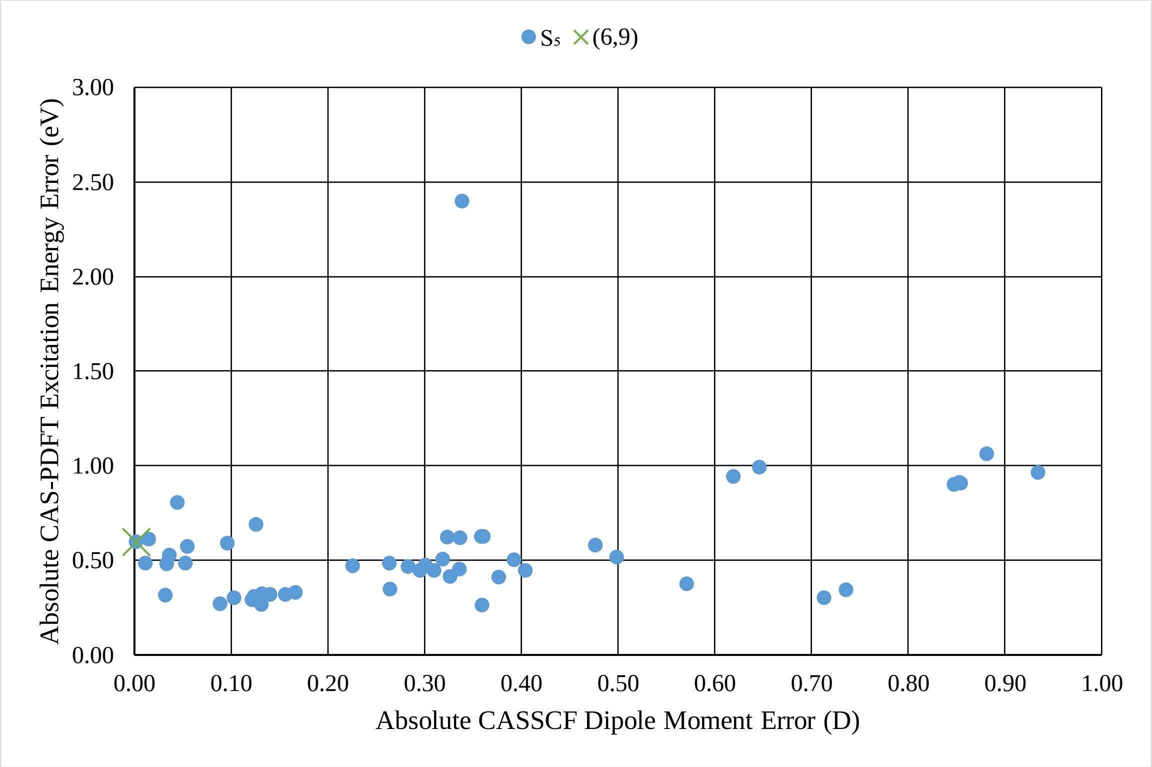

Figure S26: Plot of CAS-PDFT excitation energy error with respect to ground-state CASSCF dipole moment error for benzonitrile.

Plots of CASPT2 Excitation Energy Error w.r.t. S_0 CASSCF Dipole Moment Error at All Active Spaces; Application of GDM-AS

All active space sizes considered in this work (PASS+) are shown in the plots although only active spaces in PASS will be chosen by GDM-AS. Active spaces chosen by GDM-AS are labeled with green crosses.

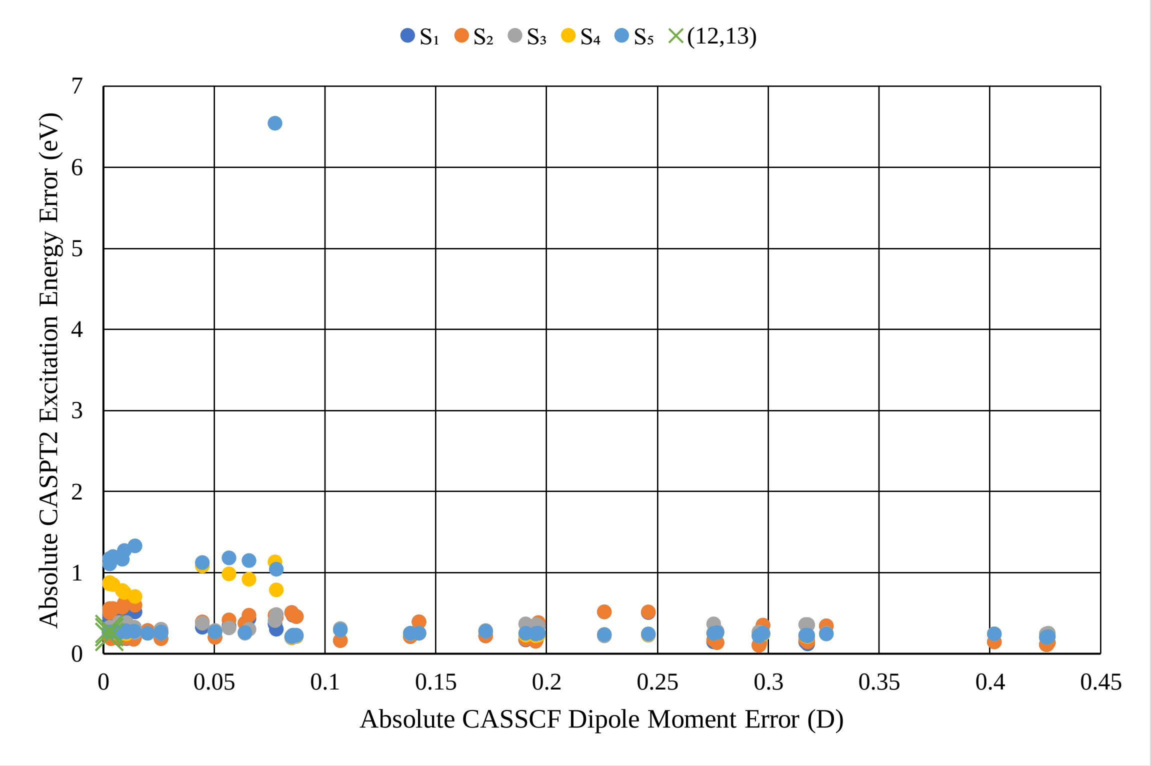

Figure S27: Plot of CASPT2 excitation energy error with respect to ground-state CASSCF dipole moment error for carbon monoxide.

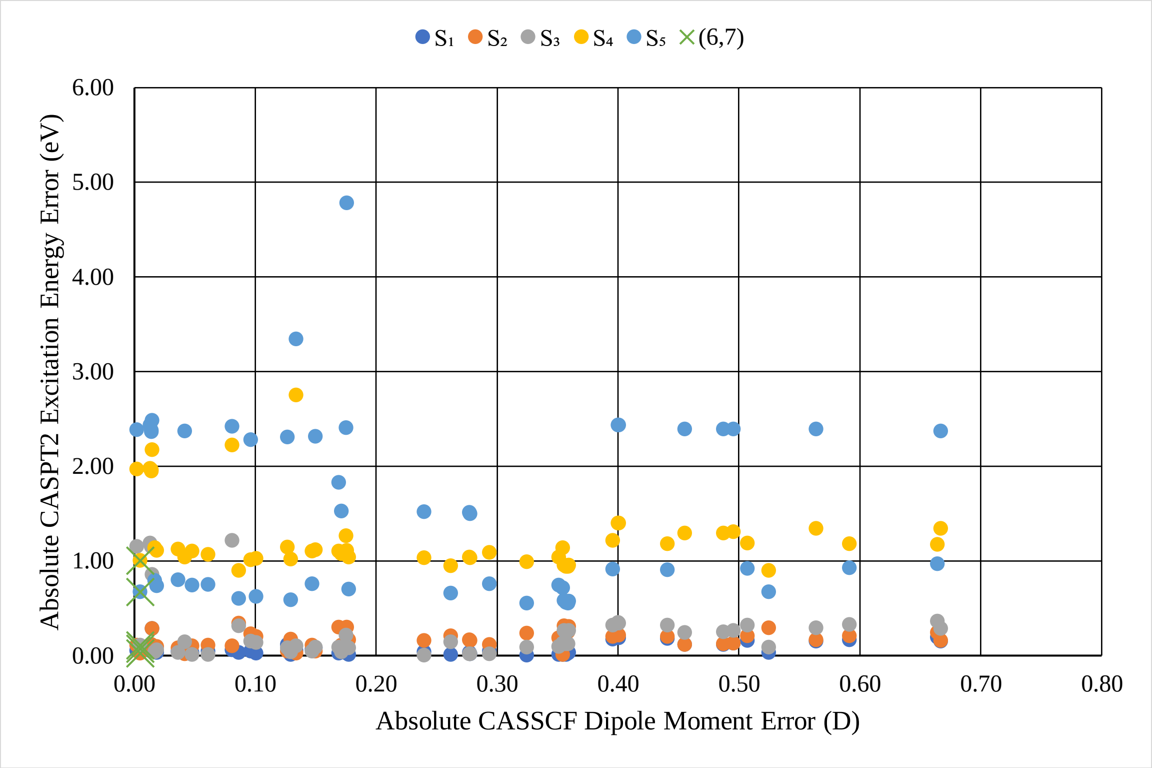

Figure S28: Plot of CASPT2 excitation energy error with respect to ground-state CASSCF dipole moment error for formaldehyde.

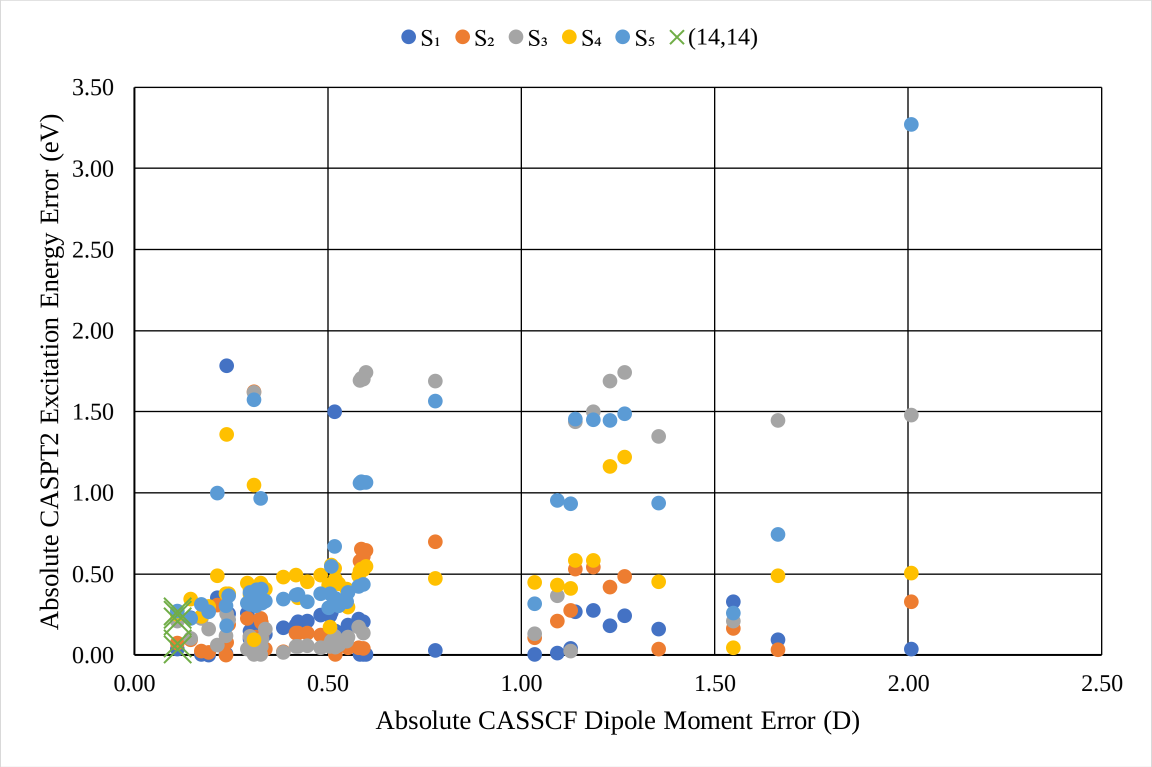

Figure S29: Plot of CASPT2 excitation energy error with respect to ground-state CASSCF dipole moment error for pyridine.

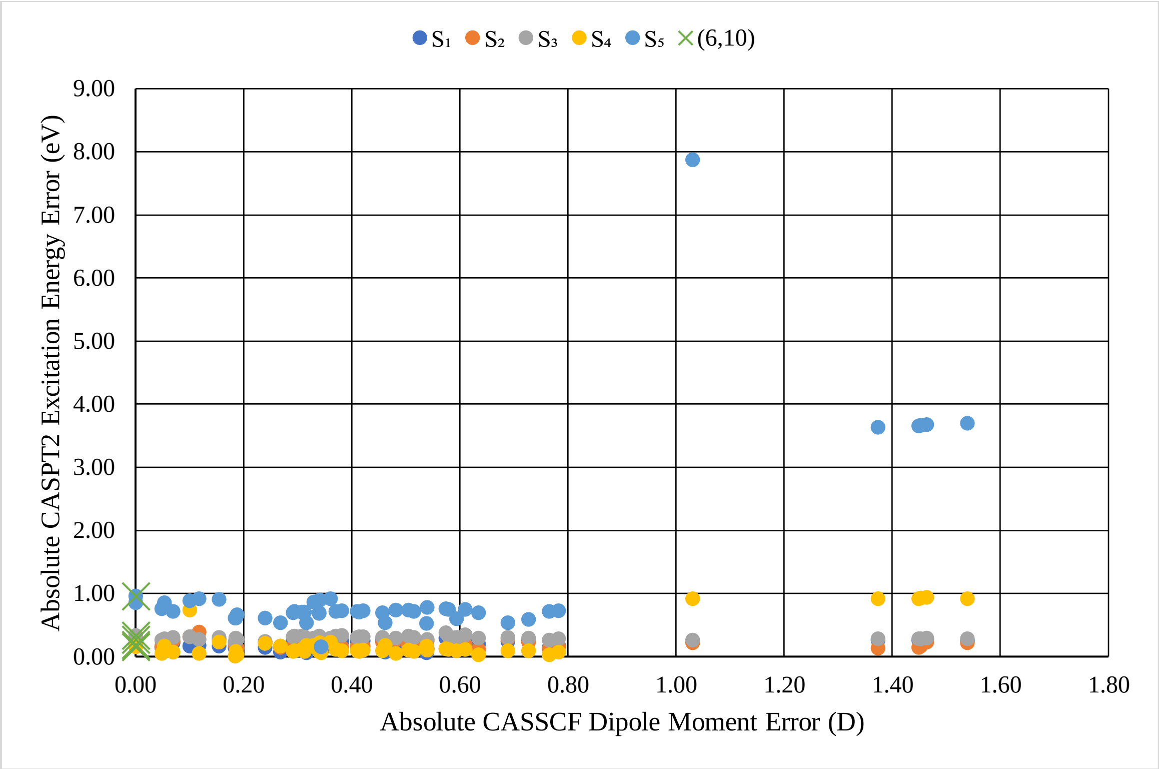

Figure S30: Plot of CASPT2 excitation energy error with respect to ground-state CASSCF dipole moment error for pyrrole.

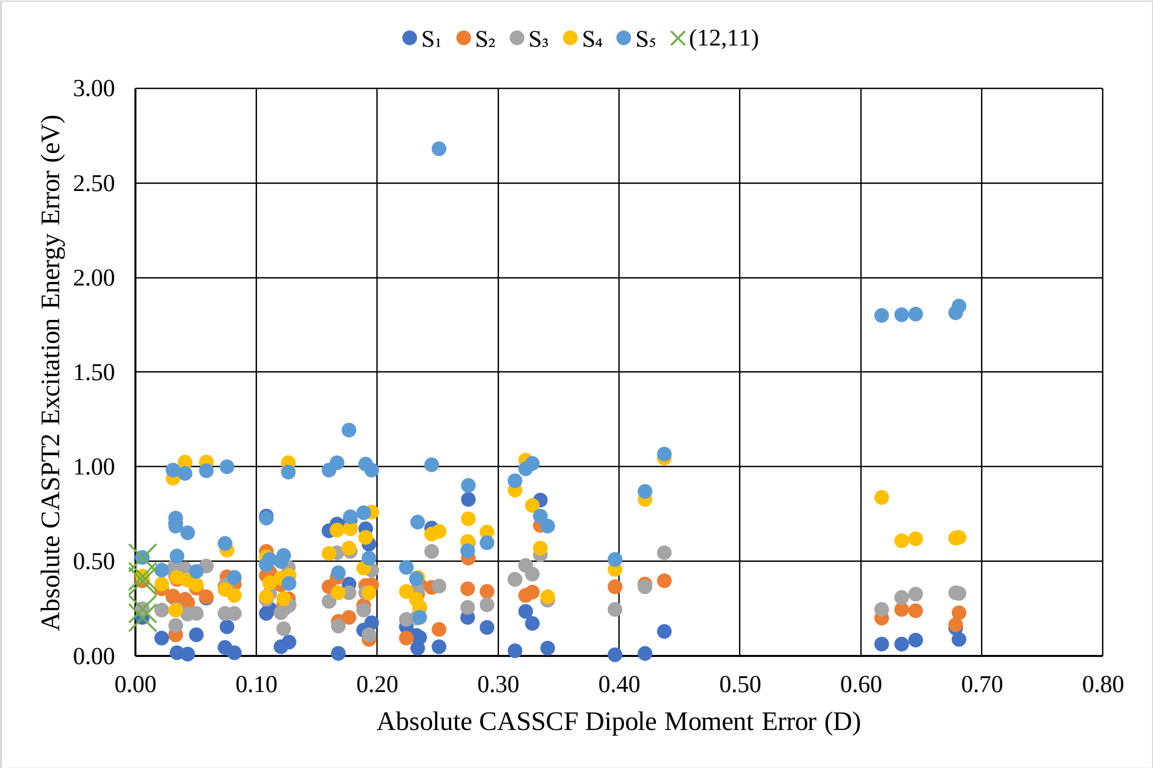

Figure S31: Plot of CASPT2 excitation energy error with respect to ground-state CASSCF dipole moment error for thiophene.

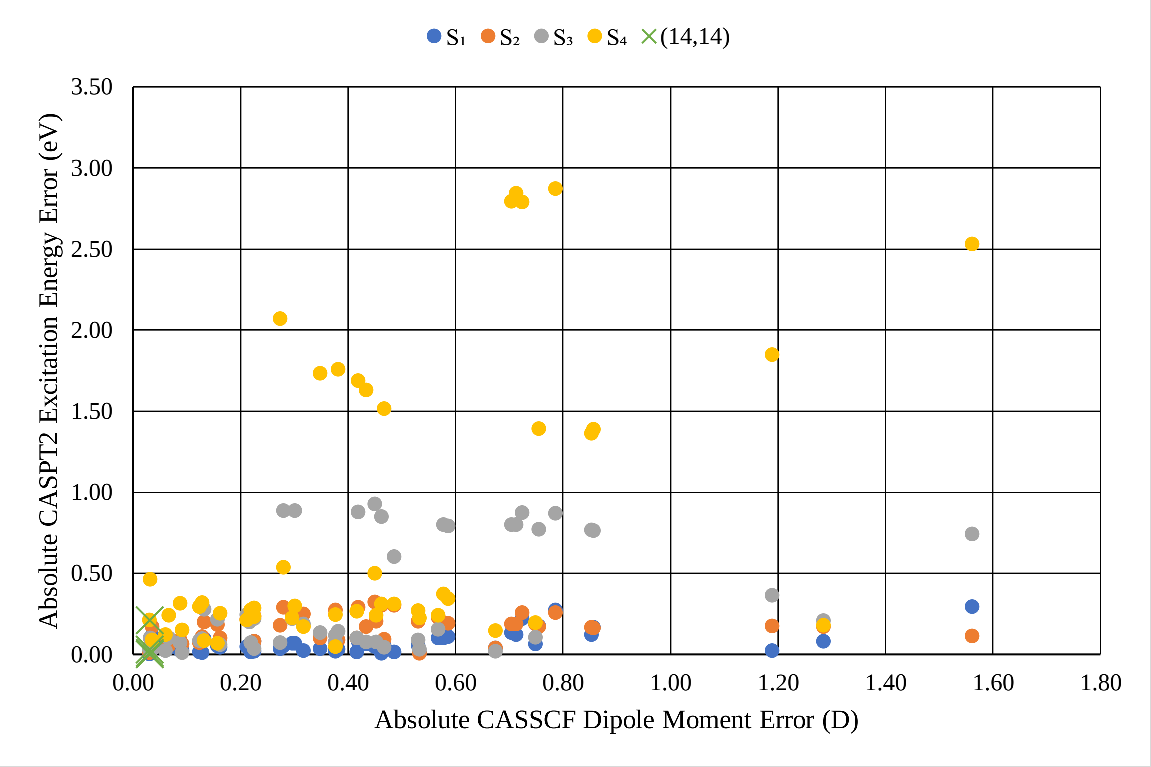

Figure S32: Plot of CASPT2 excitation energy error with respect to ground-state CASSCF dipole moment error for diazirine.

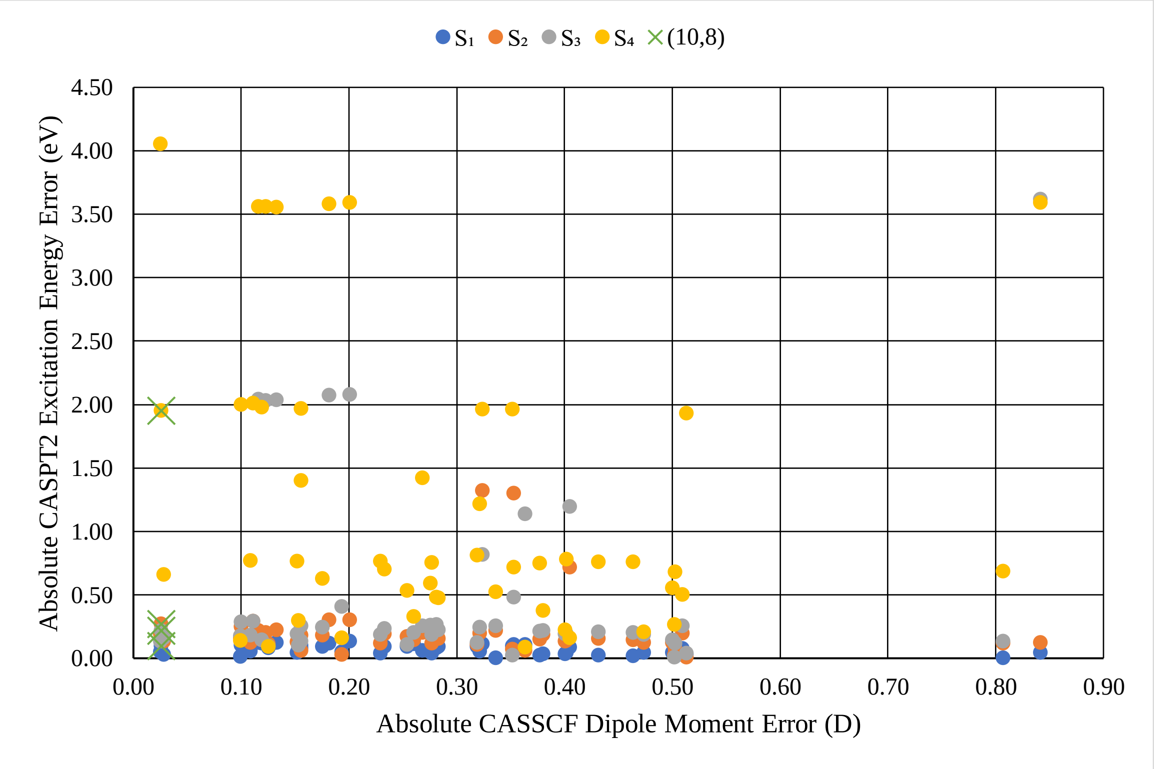

Figure S33: Plot of CASPT2 excitation energy error with respect to ground-state CASSCF dipole moment error for ketene.

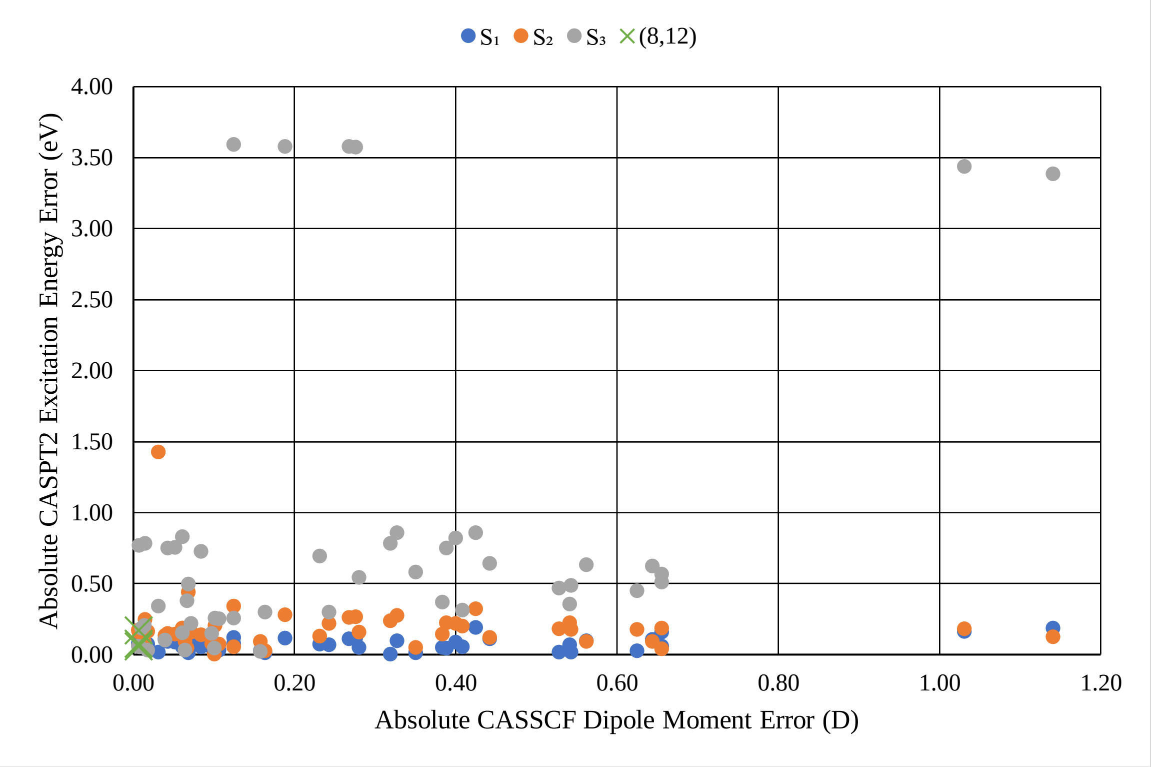

Figure S34: Plot of CASPT2 excitation energy error with respect to ground-state CASSCF dipole moment error for diazomethane.

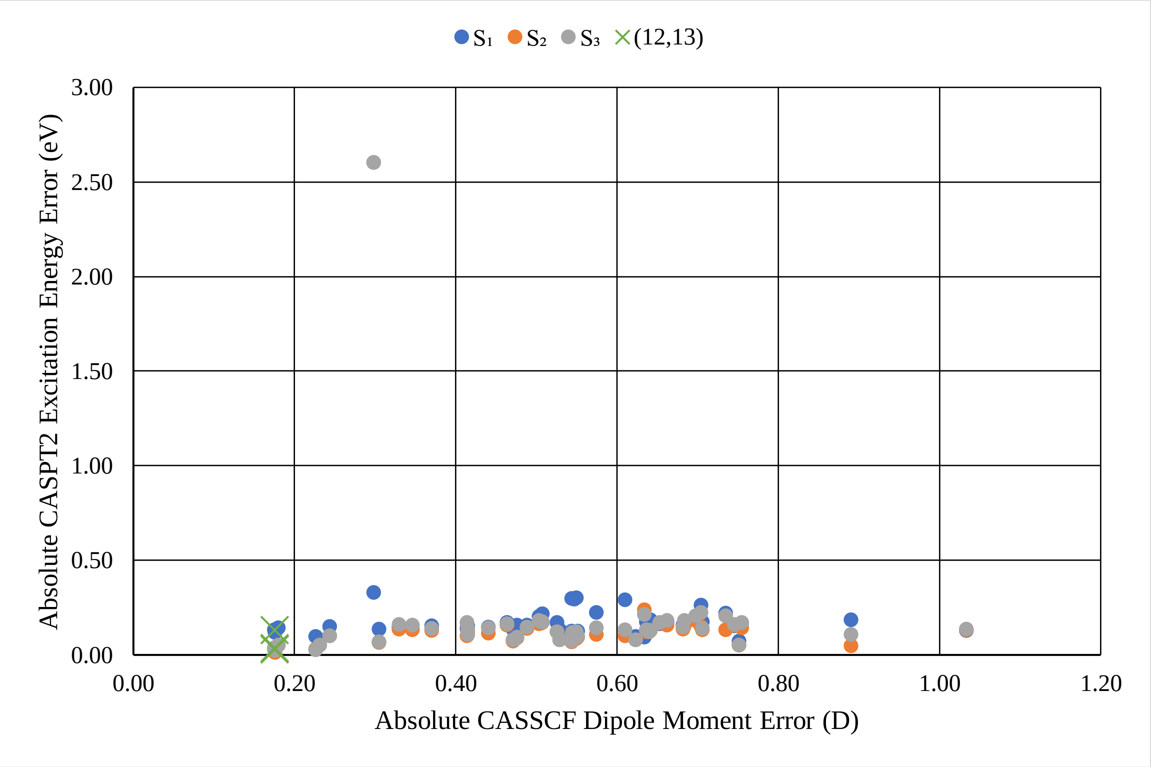

Figure S35: Plot of CASPT2 excitation energy error with respect to ground-state CASSCF dipole moment error for hydrogen cyaphide.

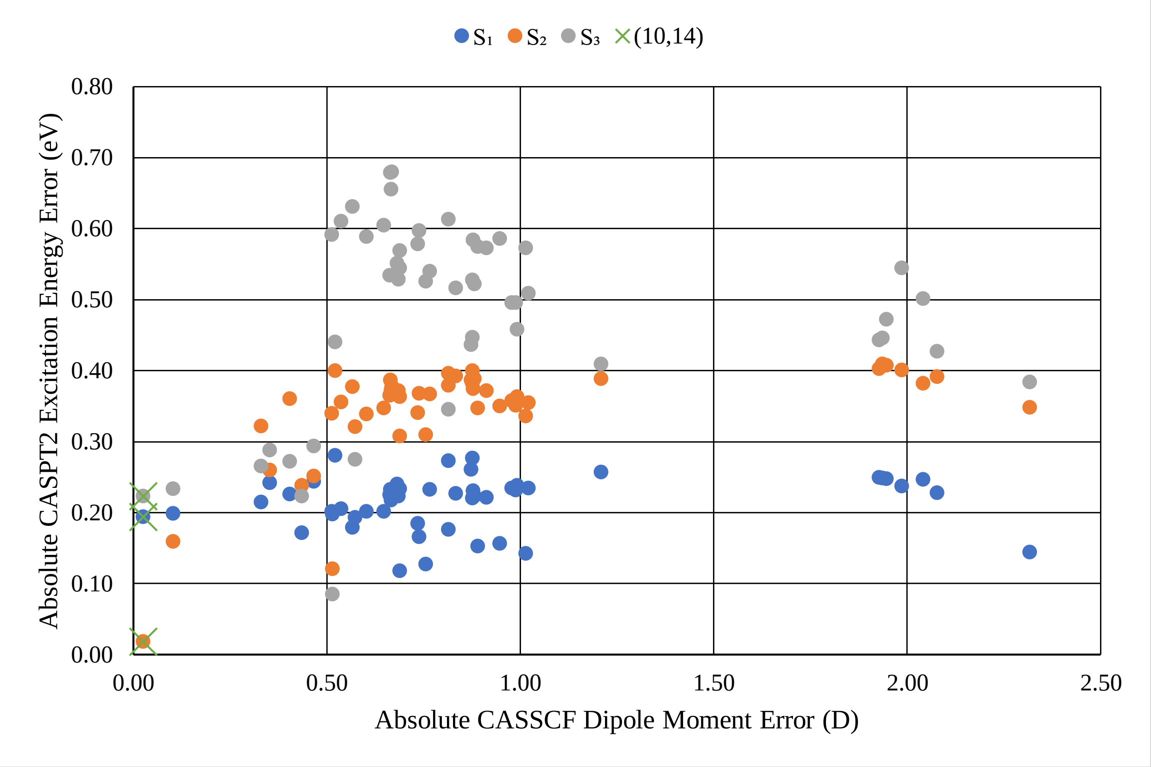

Figure S36: Plot of CASPT2 excitation energy error with respect to ground-state CASSCF dipole moment error for imidazole.

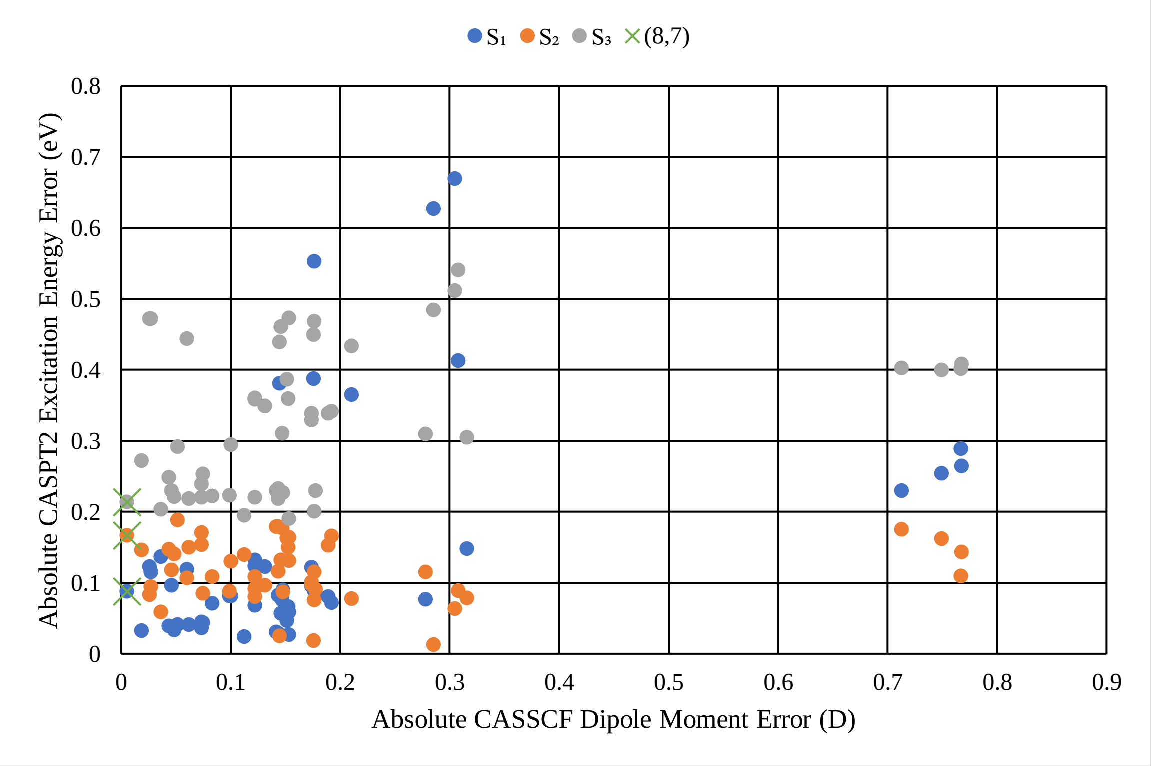

Figure S37: Plot of CASPT2 excitation energy error with respect to ground-state CASSCF dipole moment error for nitrosyl hydride.

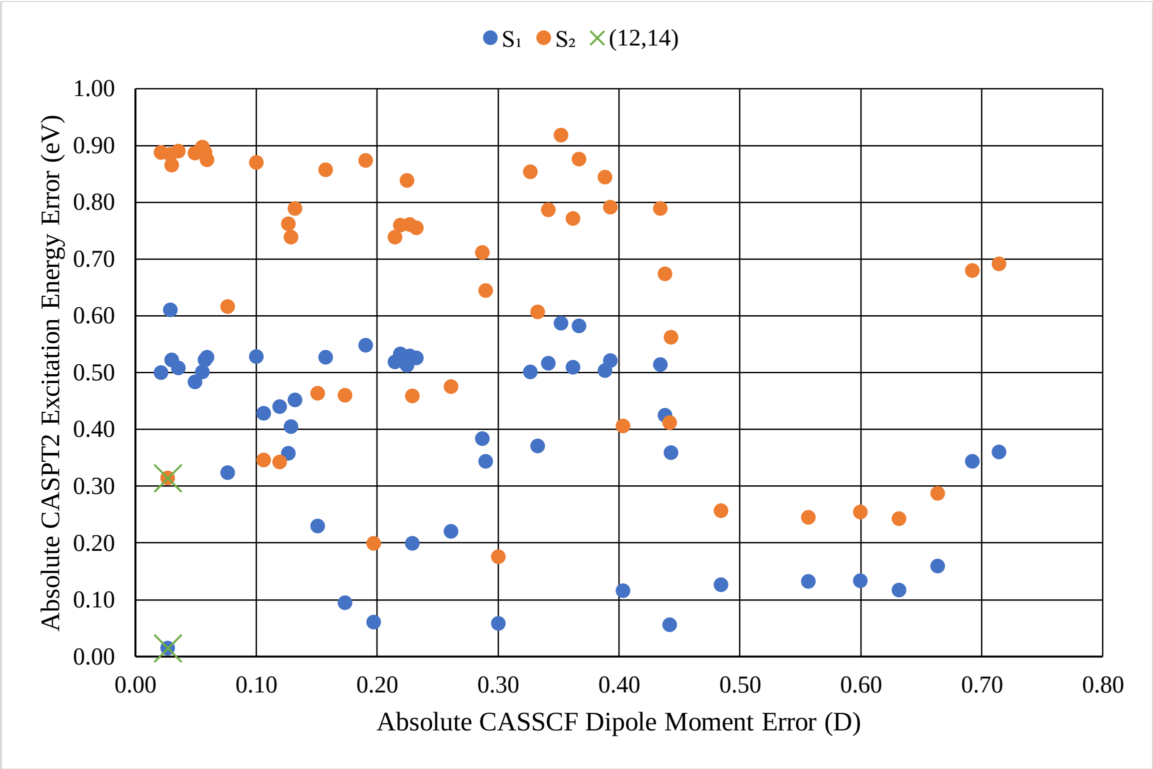

Figure S38: Plot of CASPT2 excitation energy error with respect to ground-state CASSCF dipole moment error for cyclopropene.

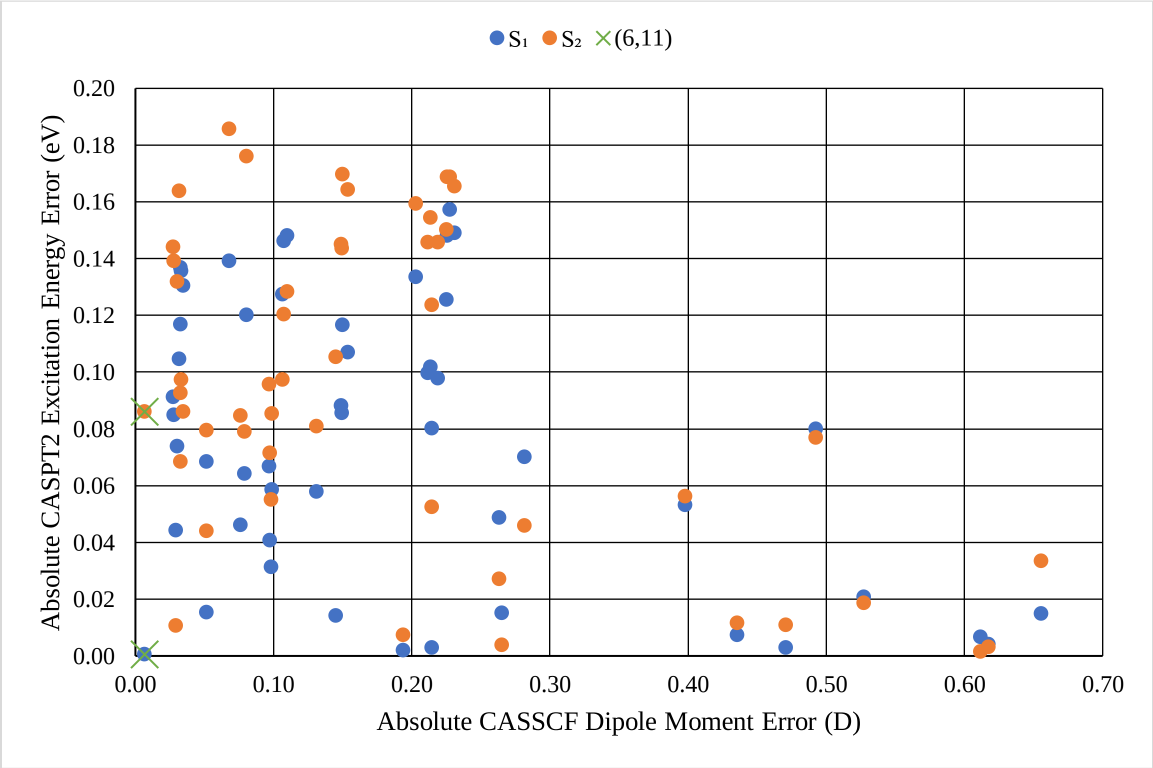

Figure S39: Plot of CASPT2 excitation energy error with respect to ground-state CASSCF dipole moment error for hydrogen sulfide.

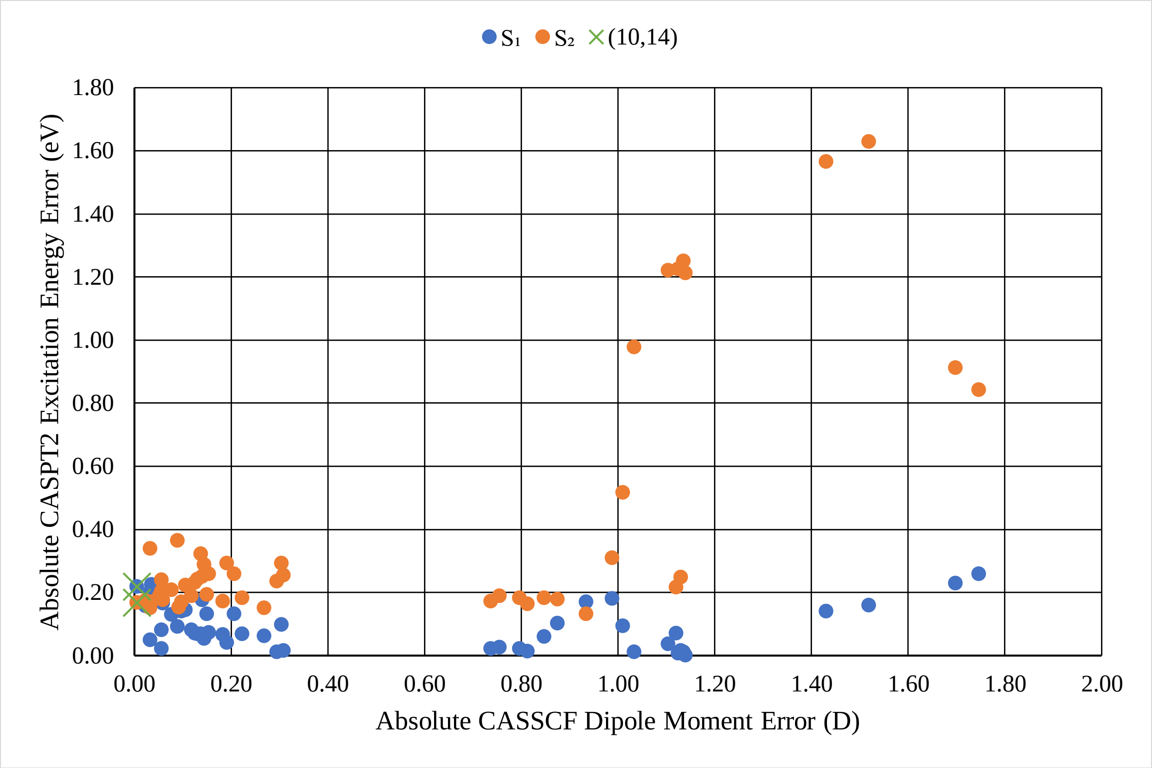

Figure S40: Plot of CASPT2 excitation energy error with respect to ground-state CASSCF dipole moment error for propynal.

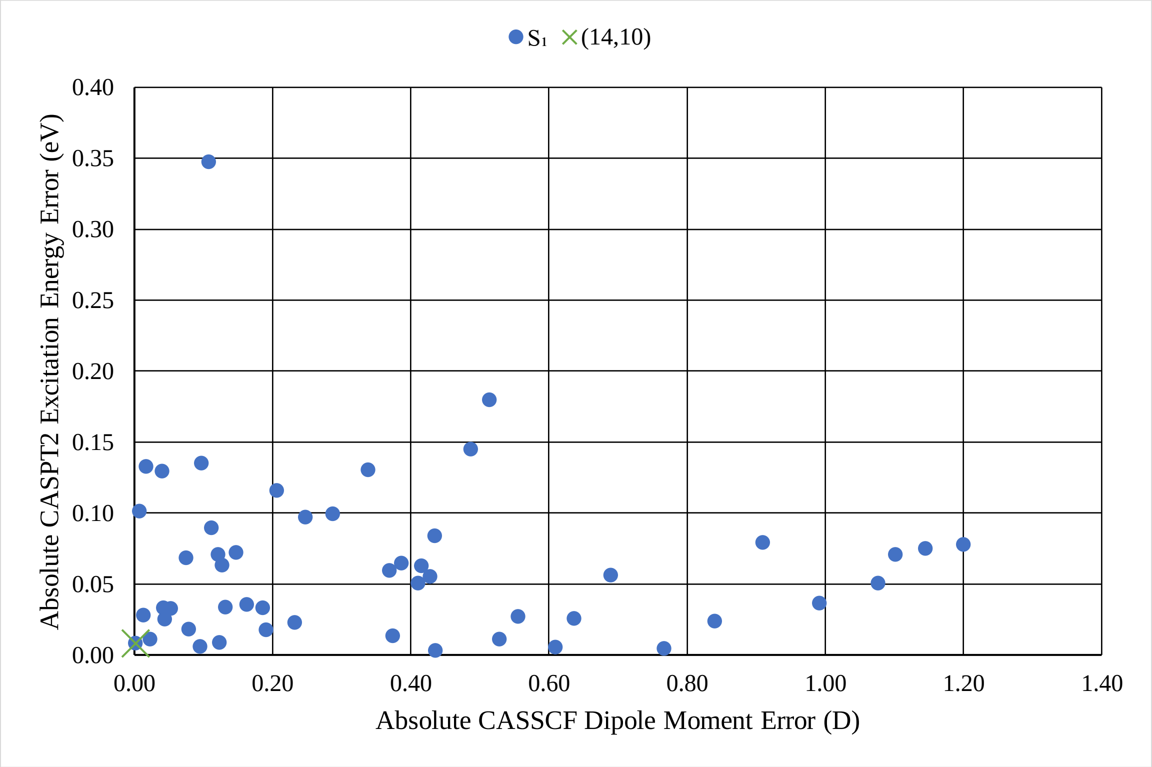

Figure S41: Plot of CASPT2 excitation energy error with respect to ground-state CASSCF dipole moment error for acetaldehyde.

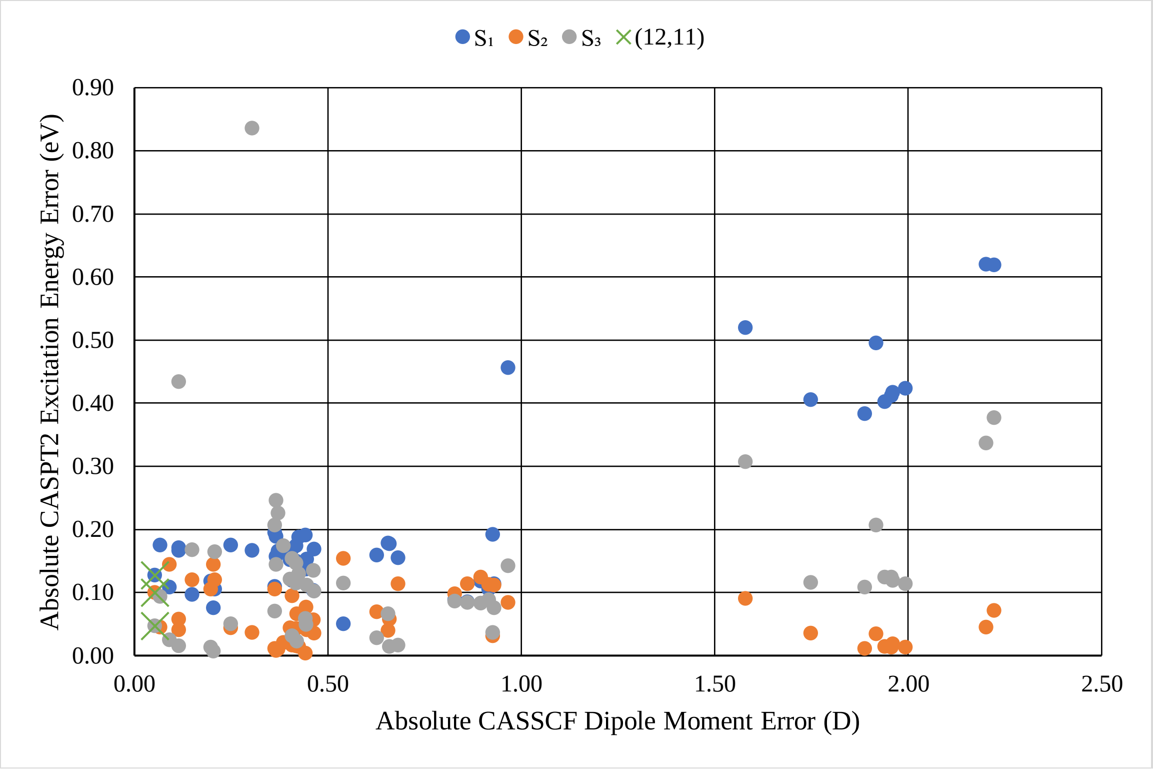

Figure S42: Plot of CASPT2 excitation energy error with respect to ground-state CASSCF dipole moment error for nitrosomethane.

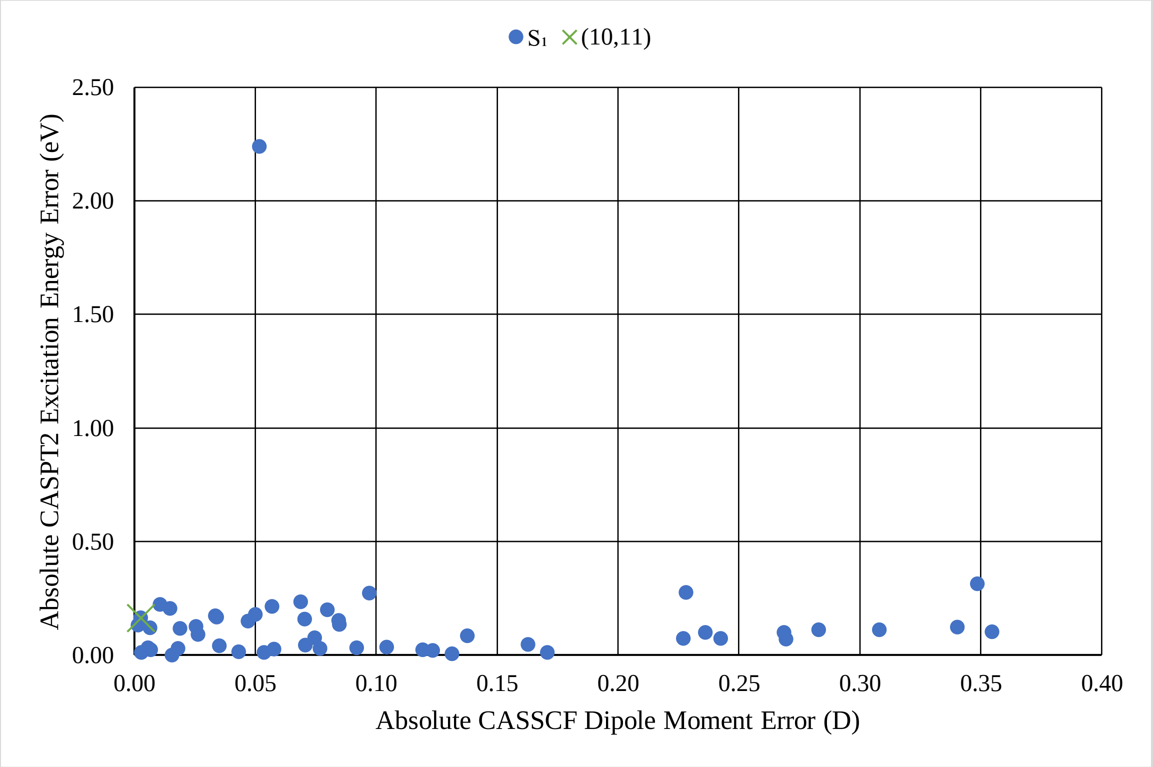

Figure S43: Plot of CASPT2 excitation energy error with respect to ground-state CASSCF dipole moment error for difluorocarbene.

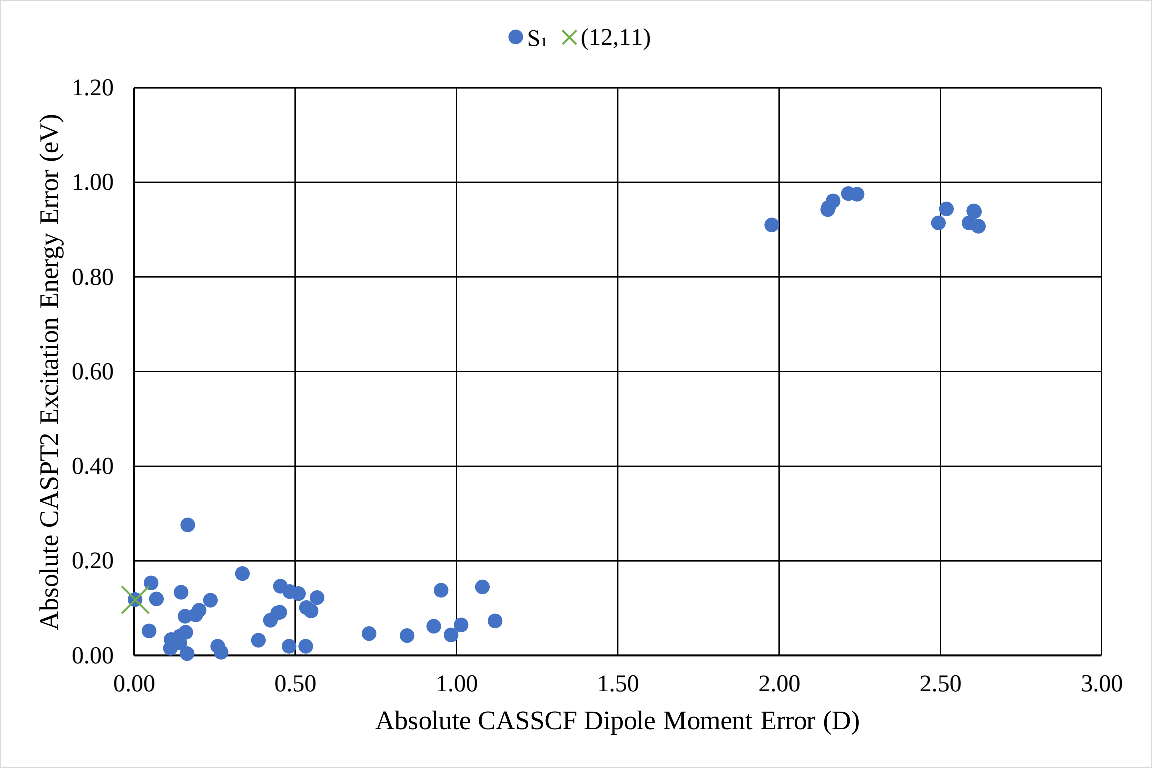

Figure S44: Plot of CASPT2 excitation energy error with respect to ground-state CASSCF dipole moment error for formamide.

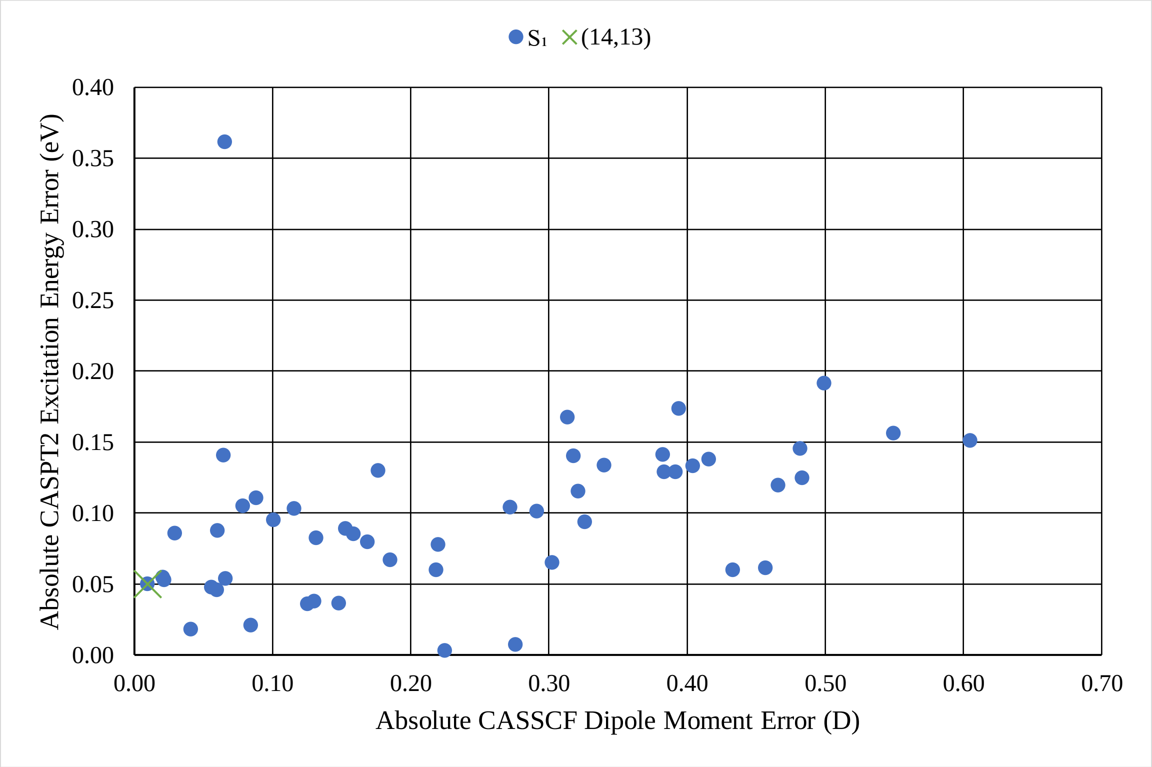

Figure S45: Plot of CASPT2 excitation energy error with respect to ground-state CASSCF dipole moment error for formyl fluoride.

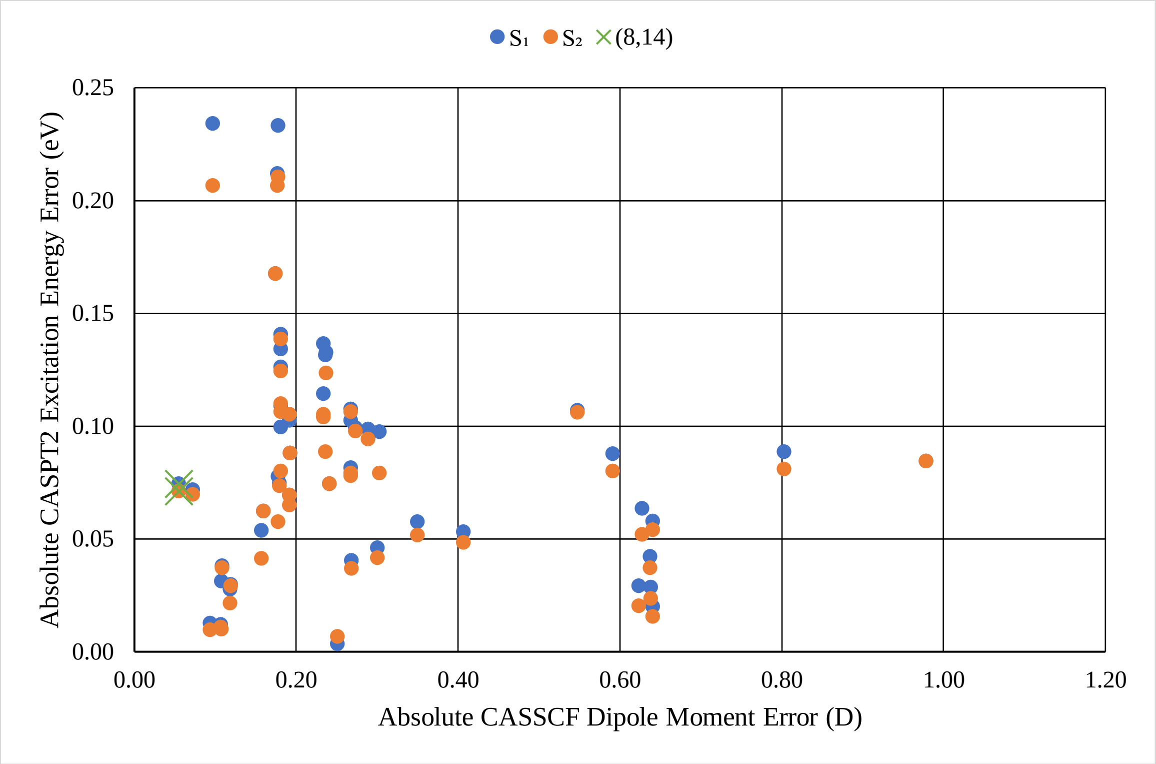

Figure S46: Plot of CASPT2 excitation energy error with respect to ground-state CASSCF dipole moment error for hydrogen chloride.

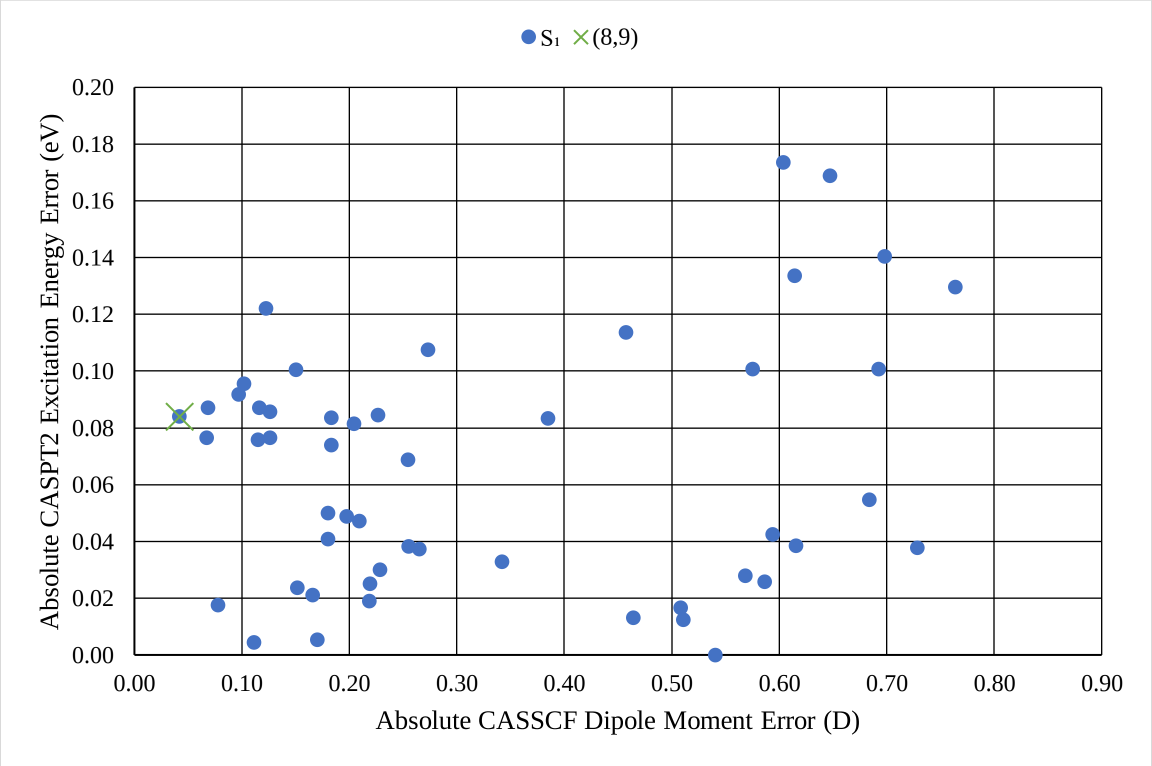

Figure S47: Plot of CASPT2 excitation energy error with respect to ground-state CASSCF dipole moment error for methanimine.

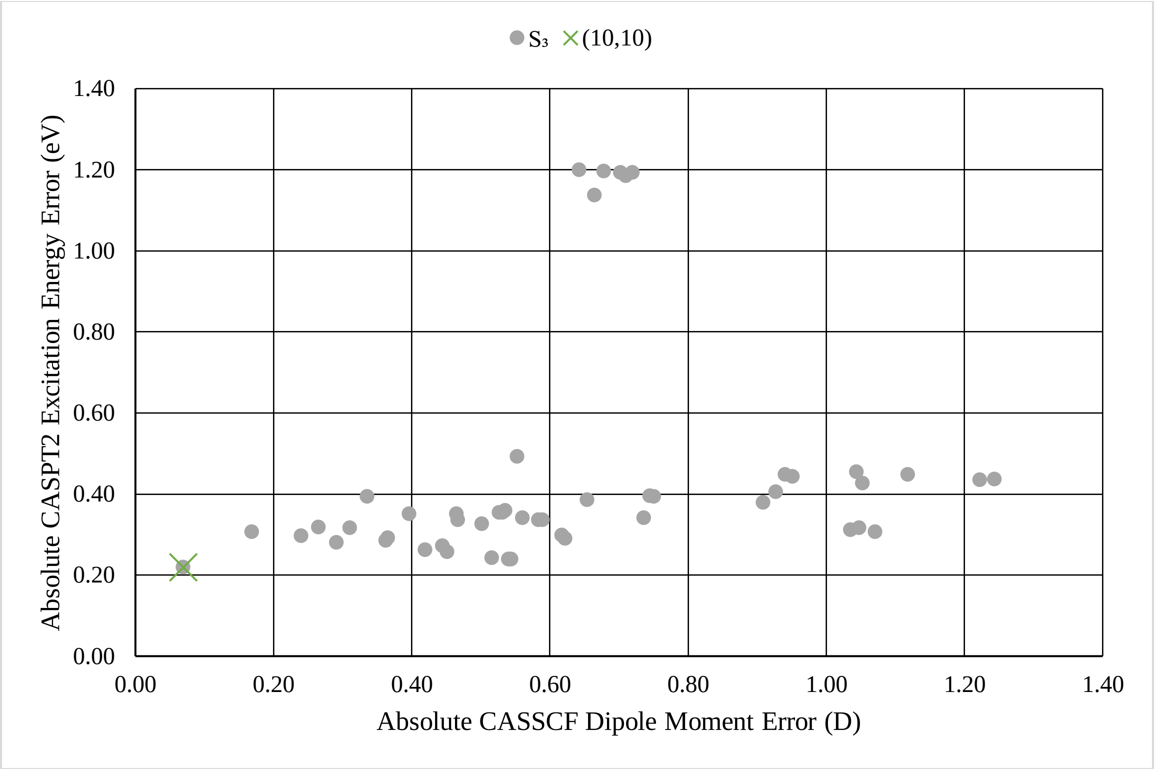

Figure S48: Plot of CASPT2 excitation energy error with respect to ground-state CASSCF dipole moment error for aniline.

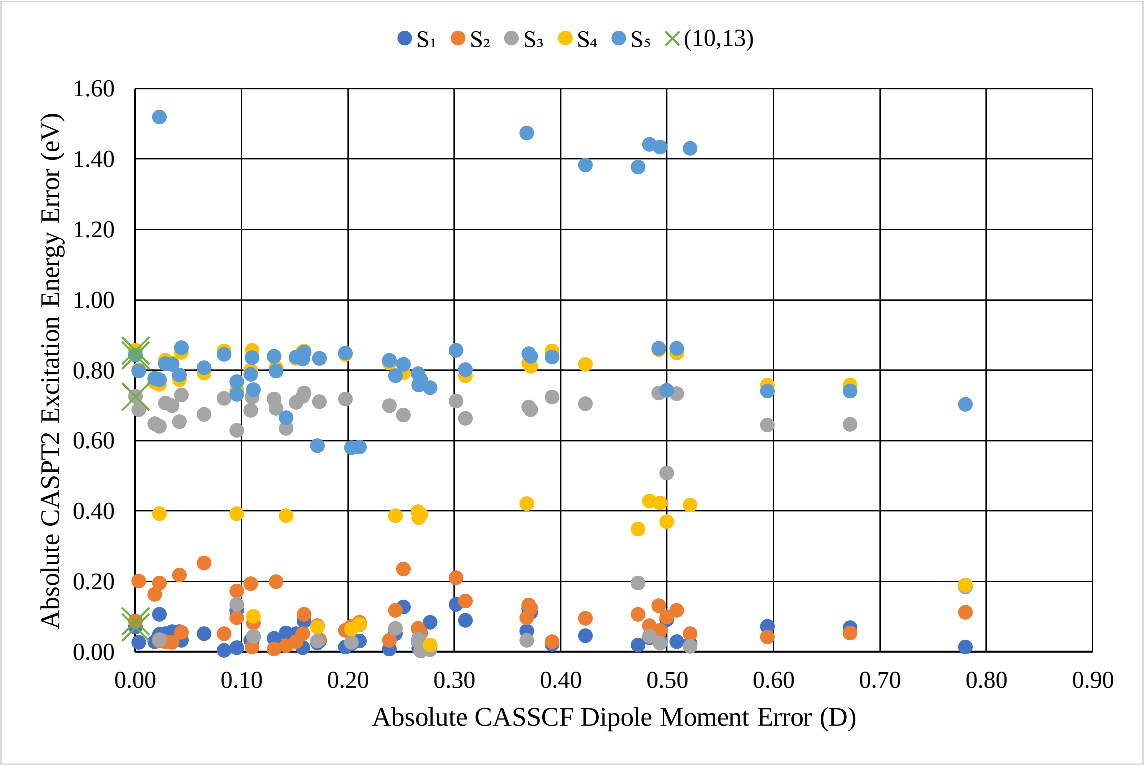

Figure S49: Plot of CASPT2 excitation energy error with respect to ground-state CASSCF dipole moment error for azulene.

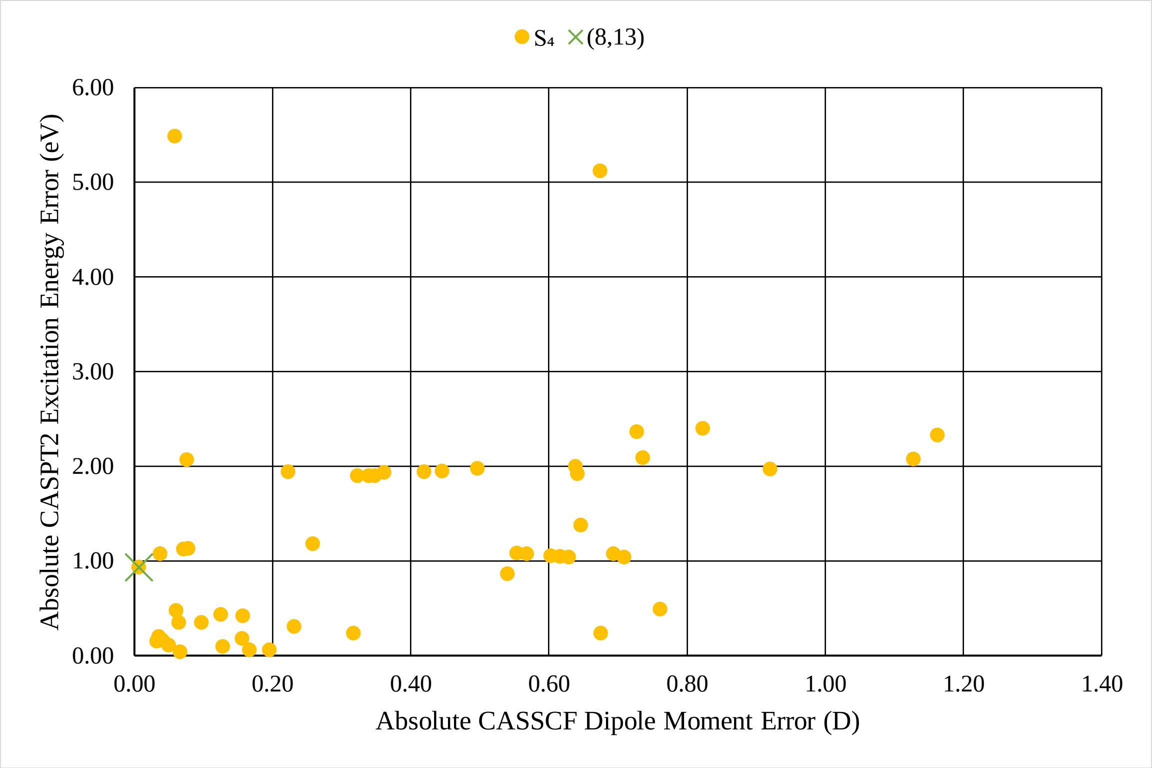

Figure S50: Plot of CASPT2 excitation energy error with respect to ground-state CASSCF dipole moment error for nitrobenzene.

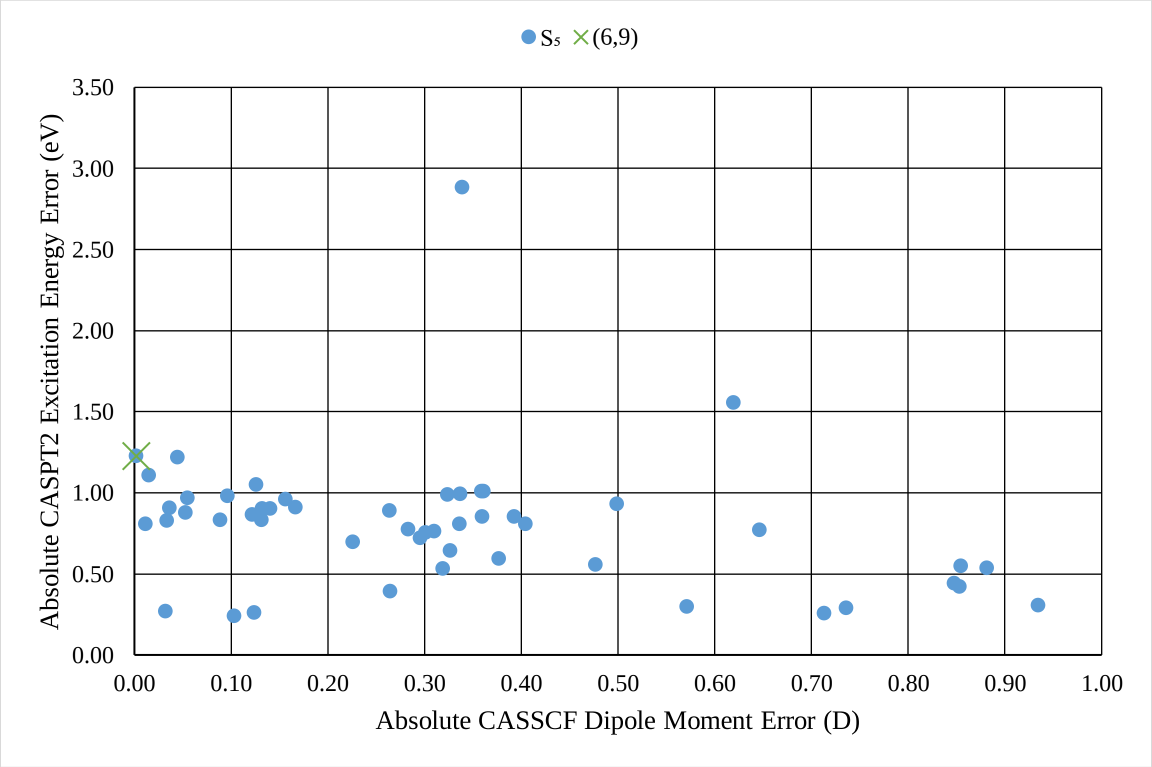

Figure S51: Plot of CASPT2 excitation energy error with respect to ground-state CASSCF dipole moment error for benzonitrile.

Comparisons of the Excitation Characters to Those in QUESTDB

Table S4: CAS-PDFT excitation energies (in eV), symmetries, characters, and oscillator strengths for the first three excitations of nitrosyl hydride, from using the active space selected by GDM-AS, compared to those in QUESTDB.

Exct.	Exc. Energy (eV)		Symmetry		Character		Osc. Strength	
	QUEST	(8,7)	QUEST	(8,7)	QUEST	(8,7)	QUEST	(8,7)
$S_0 \rightarrow S_1$	1.74	1.69	A"	A"	n→π*	n→π*	0.000	0.001
$S_0 \rightarrow S_2$	4.33	4.30	A'	A'	double	double	0.000	0.000
$S_0 \rightarrow S_3$	6.27	6.27	A'	A'	n.d.	n→3s	0.034	0.031

Table S5: CAS-PDFT excitation energies (in eV), symmetries, characters, and oscillator strengths for the first excitation of methanimine, from using the active space selected by GDM-AS, compared to those in QUESTDB.

Exct.	Exc. Energy (eV)		Symmetry		Charater		Osc. Strength	
	QUEST	(8,9)	QUEST	(8,9)	QUEST	(8,9)	QUEST	(8,9)
$S_0 \rightarrow S_1$	5.23	5.19	A"	A"	n→π*	n→π*	0.003	0.010

vGDM-AS (GDM-AS with the Direction of Dipole Moments Considered)

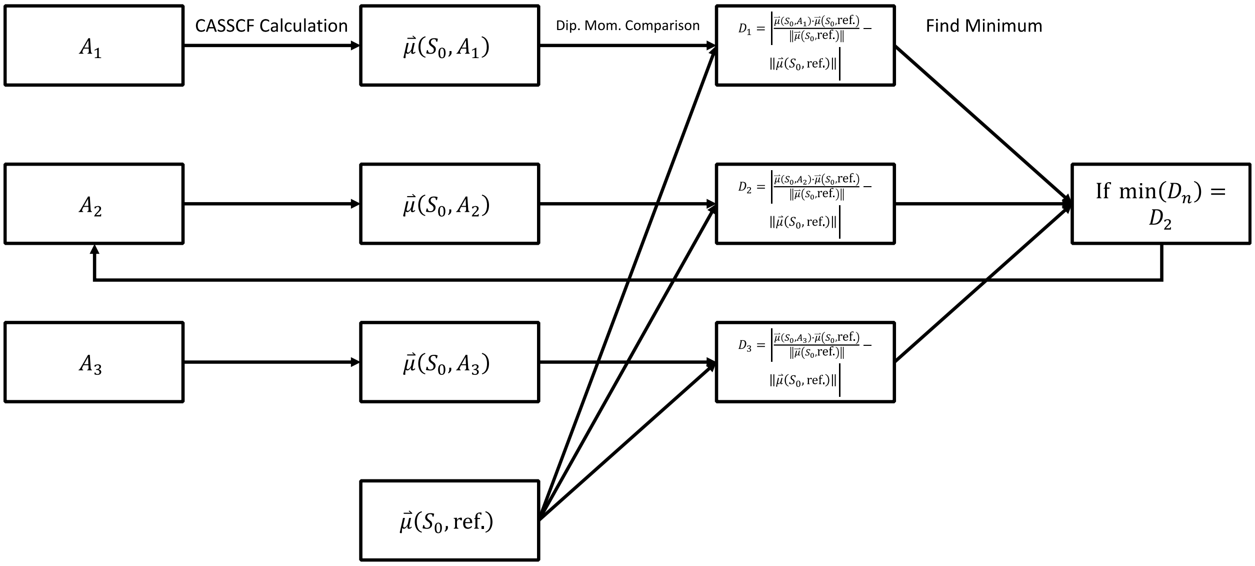

Figure S52: Schematic representation of vGDM-AS, which compares the direction of the reference and CASSCF dipole moments to choose an active space.

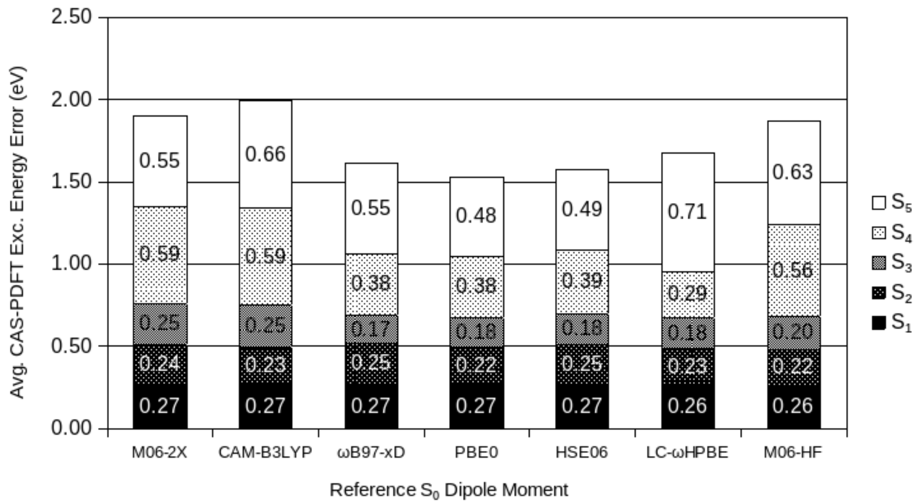

Figure S53: Average CAS-PDFT excitation energy error for active spaces chosen by vGDM-AS, accounting for the direction of the dipole moments and using reference dipole provided by a series of density functionals. The ANO-RCC-VTZP basis set is always used.

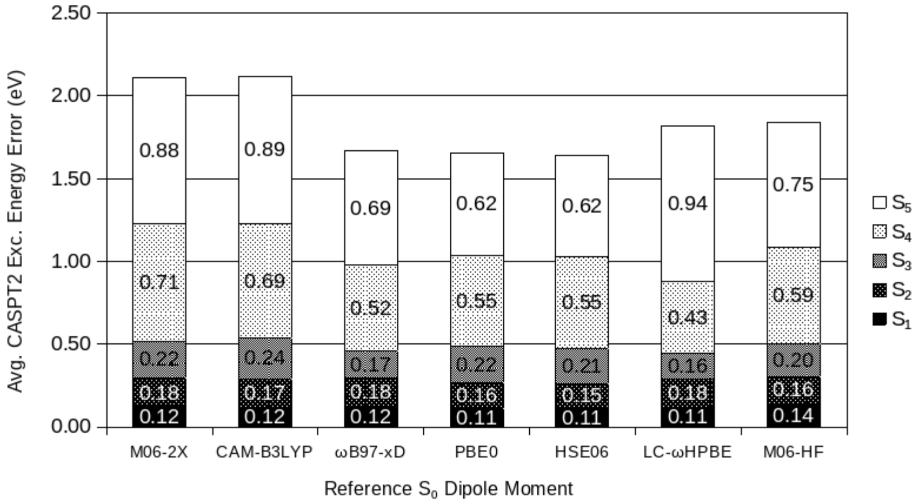

Figure S54: Average CASPT2 excitation energy error for active spaces chosen by vGDM-AS, accounting for the direction of the dipole moments and using reference dipole provided by a series of density functionals. The ANO-RCC-VTZP basis set is always used.

Table S6: Test systems with differences in active spaces recommended by GDM-AS accounting for only the total dipole moment and vGDM-AS accounting for the direction of the dipole moment, and the corresponding recommended active spaces and resulting excitation energy errors. Note the molecules with the same active spaces chosen for both protocols (22 for LC-ωPBE, 21 for HSE06, 20 for M06-2X, CAM-B3LYP and PBE0, 19 for ωB97-xD, and 18 for M06-HF) are omitted.

Original GDM-AS

M06-2X

Molecule	Active Space	CAS-PDFT Excitation Energy Error (eV)					CASPT2 Excitation Energy Error (eV)				
		S_1	S_2	S_3	S_4	S_5	S_1	S_2	S_3	S_4	S_5
Imidazole	(10,14)	0.04	0.12	0.14			0.19	0.02	0.22		
Nitrosyl Hydride	(6,6)	0.04	0.07	0.05			0.10	0.12	0.23		
Difluorocarbene	(8,7)	1.19					0.15				
Formamide	(8,8)	0.44					0.02				
Formyl Fluoride	(12,13)	0.05					0.10				

CAM-B3LYP

Molecule	Active Space	CAS-PDFT Excitation Energy Error (eV)					CASPT2 Excitation Energy Error (eV)				
		S_1	S_2	S_3	S_4	S_5	S_1	S_2	S_3	S_4	S_5
Nitrosyl Hydride	(10,8)	0.08	0.03	0.04			0.12	0.11	0.44		
Acetaldehyde	(8,7)	0.03					0.02				
Difluorocarbene	(8,11)	0.95					0.00				
Formyl Fluoride	(12,13)	0.05					0.10				
Methanimine	(12,13)	0.25					0.08				

ωB97-xD

Molecule	Active Space	CAS-PDFT Excitation Energy Error (eV)					CASPT2 Excitation Energy Error (eV)				
		S_1	S_2	S_3	S_4	S_5	S_1	S_2	S_3	S_4	S_5
Nitrosyl Hydride	(8,7)	0.05	0.03	0.00			0.09	0.17	0.21		
Hydrogen Sulfide	(6,6)	0.35	0.29				0.07	0.08			
Acetaldehyde	(6,9)	0.44					0.07				
Formyl Fluoride	(10,11)	0.10					0.14				
Methanimine	(12,13)	0.25					0.08				
Nitrobenzene	(14,12)				0.23					0.31	

PBE0

Molecule	Active Space	CAS-PDFT Excitation Energy Error (eV)					CASPT2 Excitation Energy Error (eV)				
		S_1	S_2	S_3	S_4	S_5	S_1	S_2	S_3	S_4	S_5
Nitrosyl Hydride	(10,14)	0.04	0.05	0.24			0.03	0.15	0.27		
Acetaldehyde	(8,11)	0.63					0.09				
Formyl Fluoride	(8,10)	0.00					0.09				
Methanimine	(8,9)	0.04					0.08				
Azulene	(12,12)	0.29	0.41	0.56	0.58	0.58	0.01	0.05	0.72	0.85	0.83

HSE06

Molecule	Active Space	CAS-PDFT Excitation Energy Error (eV)					CASPT2 Excitation Energy Error (eV)				
		S_1	S_2	S_3	S_4	S_5	S_1	S_2	S_3	S_4	S_5
Nitrosyl Hydride	(10,14)	0.04	0.05	0.24			0.03	0.15	0.27		
Acetaldehyde	(8,11)	0.63					0.09				
Formyl Fluoride	(8,10)	0.00					0.09				
Methanimine	(8,9)	0.04					0.08				

LC-ωHPBE

Molecule	Active Space	CAS-PDFT Excitation Energy Error (eV)					CASPT2 Excitation Energy Error (eV)				
		S_1	S_2	S_3	S_4	S_5	S_1	S_2	S_3	S_4	S_5
Nitrosyl Hydride	(6,12)	0.04	0.02	0.16			0.07	0.11	0.22		
Formyl Fluoride	(14,10)	0.11					0.11				
Methanimine	(8,14)	0.02					0.02				

M06-HF

Molecule	Active Space	CAS-PDFT Excitation Energy Error (eV)					CASPT2 Excitation Energy Error (eV)				
		S_1	S_2	S_3	S_4	S_5	S_1	S_2	S_3	S_4	S_5
Nitrosomethane	(12,10)	0.10	0.07	0.04			0.17	0.01	0.23		
Cyclopropene	(12,14)	0.12	0.16				0.01	0.31			
Acetaldehyde	(8,8)	0.16					0.05				
Difluorocarbene	(6,14)	0.87					0.02				
Formamide	(10,14)	0.30					0.02				
Formyl Fluoride	(6,9)	0.12					0.17				
Methanimine	(8,8)	0.04					0.08				

GDM-AS with Dipole Moment Direction

M06-2X

Molecule	Active Space	CAS-PDFT Excitation Energy Error (eV)					CASPT2 Excitation Energy Error (eV)				
		S_1	S_2	S_3	S_4	S_5	S_1	S_2	S_3	S_4	S_5
Imidazole	(10,13)	0.03	0.10	0.15			0.19	0.02	0.22		
Nitrosyl Hydride	(8,12)	0.06	0.00	0.21			0.04	0.15	0.22		
Difluorocarbene	(8,11)	0.95					0.00				
Formamide	(6,7)	0.48					0.01				
Formyl Fluoride	(6,7)	0.09					0.08				

CAM-B3LYP

Molecule	Active Space	CAS-PDFT Excitation Energy Error (eV)					CASPT2 Excitation Energy Error (eV)				
		S_1	S_2	S_3	S_4	S_5	S_1	S_2	S_3	S_4	S_5
Nitrosyl Hydride	(10,11)	0.08	0.04	0.18			0.04	0.19	0.29		
Acetaldehyde	(8,12)	0.06					0.02				
Difluorocarbene	(12,14)	0.80					0.05				
Formyl Fluoride	(8,13)	0.02					0.10				
Methanimine	(14,13)	0.18					0.09				

ωB97-xD

Molecule	Active Space	CAS-PDFT Excitation Energy Error (eV)					CASPT2 Excitation Energy Error (eV)				
		S_1	S_2	S_3	S_4	S_5	S_1	S_2	S_3	S_4	S_5
Nitrosyl Hydride	(6,7)	0.12	0.02	0.02			0.14	0.06	0.20		
Hydrogen Sulfide	(6,13)	0.38	0.35				0.05	0.08			
Acetaldehyde	(12,10)	0.18					0.04				
Formyl Fluoride	(12,9)	0.07					0.11				
Methanimine	(12,12)	0.18					0.08				
Nitrobenzene	(6,14)				0.58					1.08	

PBE0

Molecule	Active Space	CAS-PDFT Excitation Energy Error (eV)					CASPT2 Excitation Energy Error (eV)				
		S_1	S_2	S_3	S_4	S_5	S_1	S_2	S_3	S_4	S_5
Nitrosyl Hydride	(8,7)	0.05	0.03	0.00			0.09	0.17	0.21		
Acetaldehyde	(10,10)	0.17					0.06				
Formyl Fluoride	(6,6)	0.03					0.02				
Methanimine	(12,13)	0.25					0.08				
Azulene	(10,12)	0.36	0.05	0.48	0.50	0.52	0.09	0.11	0.74	0.85	0.85

HSE06

Molecule	Active Space	CAS-PDFT Excitation Energy Error (eV)					CASPT2 Excitation Energy Error (eV)				
		S_1	S_2	S_3	S_4	S_5	S_1	S_2	S_3	S_4	S_5
Nitrosyl Hydride	(8,7)	0.05	0.03	0.00			0.09	0.17	0.21		
Acetaldehyde	(10,10)	0.17					0.06				
Formyl Fluoride	(6,6)	0.03					0.02				
Methanimine	(12,13)	0.25					0.08				

LC-ωHPBE

Molecule	Active Space	CAS-PDFT Excitation Energy Error (eV)					CASPT2 Excitation Energy Error (eV)				
		S_1	S_2	S_3	S_4	S_5	S_1	S_2	S_3	S_4	S_5
Nitrosyl Hydride	(6,11)	0.07	0.04	0.15			0.08	0.09	0.22		
Formyl Fluoride	(6,7)	0.09					0.08				
Methanimine	(12,11)	0.12					0.08				

M06-HF

Molecule	Active Space	CAS-PDFT Excitation Energy Error (eV)					CASPT2 Excitation Energy Error (eV)				
		S_1	S_2	S_3	S_4	S_5	S_1	S_2	S_3	S_4	S_5
Nitrosomethane	(14,10)	0.03	0.00	0.01			0.17	0.02	0.17		
Cyclopropene	(6,14)	0.18	0.31				0.61	0.88			
Acetaldehyde	(6,7)	0.19					0.06				
Difluorocarbene	(6,13)	1.05					0.03				
Formamide	(6,9)	0.19					0.13				
Formyl Fluoride	(12,14)	0.07					0.14				
Methanimine	(6,7)	0.08					0.11				

Active Spaces Chosen by EDM-AS with TDDFT Reference Dipole Moments

Table S7: Active spaces chosen by EDM-AS for all molecules in the dataset, and the resulting CAS-PDFT and CASPT2 excitation energy errors. TDDFT reference dipole moments are calculated with Gaussian.

M06-2X

Molecule	Active Space	CAS-PDFT Excitation Energy Error (eV)					CASPT2 Excitation Energy Error (eV)				
		S_1	S_2	S_3	S_4	S_5	S_1	S_2	S_3	S_4	S_5
Carbon Monoxide	(8,9)	0.63	0.63	0.25	0.46	0.46	0.16	0.16	0.30	0.29	0.29
Formaldehyde	(10,8)	0.04	0.11	0.14	0.73	1.43	0.03	0.15	0.00	1.03	1.52
Pyridine	(14,10)	0.21	0.23	0.51	0.49	0.55	0.15	0.11	0.11	0.37	0.38
Pyrrole	(8,9)	0.13	0.28	0.17	0.30	0.10	0.24	0.22	0.29	0.09	0.59
Thiophene	(14,10)	0.30	0.29	0.19	0.34	0.70	0.38	0.20	0.33	0.57	1.19
Diazirine	(14,14)	0.08	0.05	0.21	0.29		0.00	0.01	0.03	0.21	
Ketene	(10,10)	0.28	0.34	0.23	0.69		0.10	0.10	0.10	0.30	
Diazomethane	(14,12)	0.21	0.36	0.47			0.01	0.00	0.04		
Hydrogen Cyaphide	(8,14)	0.04	0.05	0.02			0.14	0.07	0.07		
Imidazole	(14,13)	0.18	0.22	0.04			0.12	0.31	0.57		
Nitrosyl Hydride	(14,10)	0.07	0.04	0.04			0.12	0.08	0.47		
Nitrosomethane	(14,11)	0.08	0.07	0.07			0.16	0.01	0.25		
Cyclopropene	(12,13)	0.05	0.09				0.06	0.20			
Hydrogen Sulfide	(12,14)	0.34	0.15				0.15	0.13			
Propynal	(8,9)	0.22	0.02				0.07	0.23			
Acetaldehyde	(10,8)	0.17					0.01				
Difluorocarbene	(8,14)	0.92					0.13				
Formamide	(6,11)	0.21					0.07				
Formyl Fluoride	(14,12)	0.11					0.07				
Hydrogen Chloride	(8,14)	0.25	0.23				0.07	0.07			
Methanimine	(12,10)	0.03					0.07				
Aniline	(6,8)			0.12					0.39		
Azulene	(10,12)	0.36	0.05	0.48	0.50	0.52	0.09	0.11	0.74	0.85	0.85
Nitrobenzene	(14,12)				0.23					0.31	
Benzonitrile	(6,13)					0.51					0.53
Average		0.22	0.19	0.21	0.45	0.61	0.11	0.13	0.26	0.45	0.77

HSEH1PBE

Molecule	Active Space	CAS-PDFT Excitation Energy Error (eV)					CASPT2 Excitation Energy Error (eV)				
		S_1	S_2	S_3	S_4	S_5	S_1	S_2	S_3	S_4	S_5
Carbon Monoxide	(8,8)	0.64	0.59	0.34	0.23	0.37	0.14	0.17	0.36	0.24	0.25
Formaldehyde	(10,10)	0.07	0.21	0.32	0.81	0.43	0.02	0.20	0.14	1.02	0.62
Pyridine	(14,11)	0.19	0.28	0.45	0.47	0.51	1.78	0.08	0.25	1.36	0.18
Pyrrole	(6,9)	0.20	0.29	0.19	0.45	0.05	0.20	0.21	0.32	0.11	0.71
Thiophene	(14,10)	0.30	0.29	0.19	0.34	0.70	0.38	0.20	0.33	0.57	1.19
Diazirine	(14,14)	0.08	0.05	0.21	0.29		0.00	0.01	0.03	0.21	
Ketene	(12,12)	0.30	0.32	0.19	0.67		0.11	0.06	1.14	0.09	
Diazomethane	(14,12)	0.21	0.36	0.47			0.01	0.00	0.04		
Hydrogen Cyaphide	(8,14)	0.04	0.05	0.02			0.14	0.07	0.07		
Imidazole	(12,12)	0.16	0.18	0.05			0.15	0.35	0.57		
Nitrosyl Hydride	(14,10)	0.07	0.04	0.04			0.12	0.08	0.47		
Nitrosomethane	(6,12)	0.03	0.07	0.08			0.10	0.11	0.09		
Cyclopropene	(12,13)	0.05	0.09				0.06	0.20			
Hydrogen Sulfide	(8,14)	0.27	0.01				0.14	0.19			
Propynal	(8,14)	0.23	0.04				0.14	0.17			
Acetaldehyde	(12,14)	0.11					0.03				
Difluorocarbene	(14,10)	0.89					0.12				
Formamide	(8,13)	0.26					0.15				
Formyl Fluoride	(14,10)	0.11					0.11				
Hydrogen Chloride	(8,12)	0.51	0.49				0.21	0.21			
Methanimine	(14,10)	0.01					0.05				
Aniline	(10,14)			0.11					0.43		
Azulene	(8,11)	0.23	0.01	0.52	0.54	0.56	0.03	0.12	0.73	0.85	0.86
Nitrobenzene	(10,12)				0.09					0.16	
Benzonitrile	(6,14)					0.58					0.56
Average		0.23	0.20	0.23	0.43	0.46	0.19	0.14	0.36	0.51	0.62

CAM-B3LYP

Molecule	Active Space	CAS-PDFT Excitation Energy Error (eV)					CASPT2 Excitation Energy Error (eV)				
		S_1	S_2	S_3	S_4	S_5	S_1	S_2	S_3	S_4	S_5
Carbon Monoxide	(8,9)	0.63	0.63	0.25	0.46	0.46	0.16	0.16	0.30	0.29	0.29
Formaldehyde	(12,12)	0.15	0.25	0.30	0.76	0.46	0.03	0.29	0.08	0.90	0.67
Pyridine	(6,6)	0.27	0.29	0.77	0.52	0.45	0.25	0.19	0.22	0.38	0.36
Pyrrole	(12,11)	0.08	0.22	0.13	0.26	0.10	0.23	0.28	0.29	0.08	0.53
Thiophene	(14,10)	0.30	0.29	0.19	0.34	0.70	0.38	0.20	0.33	0.57	1.19
Diazirine	(14,14)	0.08	0.05	0.21	0.29		0.00	0.01	0.03	0.21	
Ketene	(12,12)	0.30	0.32	0.19	0.67		0.11	0.06	1.14	0.09	
Diazomethane	(14,12)	0.21	0.36	0.47			0.01	0.00	0.04		
Hydrogen Cyaphide	(8,14)	0.04	0.05	0.02			0.14	0.07	0.07		
Imidazole	(14,13)	0.18	0.22	0.04			0.12	0.31	0.57		
Nitrosyl Hydride	(14,10)	0.07	0.04	0.04			0.12	0.08	0.47		
Nitrosomethane	(14,11)	0.08	0.07	0.07			0.16	0.01	0.25		
Cyclopropene	(12,13)	0.05	0.09				0.06	0.20			
Hydrogen Sulfide	(8,14)	0.27	0.01				0.14	0.19			
Propynal	(8,10)	0.21	0.02				0.08	0.19			
Acetaldehyde	(12,10)	0.18					0.04				
Difluorocarbene	(10,14)	0.88					0.20				
Formamide	(6,11)	0.21					0.07				
Formyl Fluoride	(14,11)	0.00					0.05				
Hydrogen Chloride	(12,14)	0.18	0.17				0.07	0.07			
Methanimine	(12,10)	0.03					0.07				
Aniline	(12,14)			0.22					0.31		
Azulene	(10,12)	0.36	0.05	0.48	0.50	0.52	0.09	0.11	0.74	0.85	0.85
Nitrobenzene	(12,13)				0.19					5.48	
Benzonitrile	(12,14)					0.27					0.83
Average		0.22	0.18	0.24	0.44	0.42	0.12	0.14	0.35	0.98	0.68

LC-wHPBE

Molecule	Active Space	CAS-PDFT Excitation Energy Error (eV)					CASPT2 Excitation Energy Error (eV)				
		S_1	S_2	S_3	S_4	S_5	S_1	S_2	S_3	S_4	S_5
Carbon Monoxide	(8,9)	0.63	0.63	0.25	0.46	0.46	0.16	0.16	0.30	0.29	0.29
Formaldehyde	(10,8)	0.04	0.11	0.14	0.73	1.43	0.03	0.15	0.00	1.03	1.52
Pyridine	(6,6)	0.27	0.29	0.77	0.52	0.45	0.25	0.19	0.22	0.38	0.36
Pyrrole	(12,11)	0.08	0.22	0.13	0.26	0.10	0.23	0.28	0.29	0.08	0.53
Thiophene	(6,11)	0.24	0.08	0.11	0.00	0.15	0.01	0.18	0.16	0.33	0.44
Diazirine	(14,13)	0.08	0.06	0.28	0.28		0.01	0.05	0.10	0.46	
Ketene	(10,10)	0.28	0.34	0.23	0.69		0.10	0.10	0.10	0.30	
Diazomethane	(12,9)	0.34	0.32	0.69			0.17	0.25	0.78		
Hydrogen Cyaphide	(8,14)	0.04	0.05	0.02			0.14	0.07	0.07		
Imidazole	(6,9)	0.20	0.22	0.11			0.18	0.38	0.63		
Nitrosyl Hydride	(14,10)	0.07	0.04	0.04			0.12	0.08	0.47		
Nitrosomethane	(14,11)	0.08	0.07	0.07			0.16	0.01	0.25		
Cyclopropene	(12,13)	0.05	0.09				0.06	0.20			
Hydrogen Sulfide	(12,14)	0.34	0.15				0.15	0.13			
Propynal	(8,9)	0.22	0.02				0.07	0.23			
Acetaldehyde	(6,14)	0.01					0.01				
Difluorocarbene	(14,10)	0.89					0.12				
Formamide	(6,11)	0.21					0.07				
Formyl Fluoride	(12,12)	0.05					0.09				
Hydrogen Chloride	(8,14)	0.25	0.23				0.07	0.07			
Methanimine	(12,10)	0.03					0.07				
Aniline	(14,11)			0.08					0.38		
Azulene	(10,12)	0.36	0.05	0.48	0.50	0.52	0.09	0.11	0.74	0.85	0.85
Nitrobenzene	(12,12)				0.16					0.09	
Benzonitrile	(8,7)					0.34					0.29
Average		0.22	0.17	0.24	0.40	0.49	0.11	0.16	0.32	0.43	0.61

wB97XD

Molecule	Active Space	CAS-PDFT Excitation Energy Error (eV)					CASPT2 Excitation Energy Error (eV)				
		S_1	S_2	S_3	S_4	S_5	S_1	S_2	S_3	S_4	S_5
Carbon Monoxide	(8,8)	0.64	0.59	0.34	0.23	0.37	0.14	0.17	0.36	0.24	0.25
Formaldehyde	(8,7)	0.04	0.04	0.10	0.78	1.41	0.04	0.11	0.03	1.07	1.52
Pyridine	(6,6)	0.27	0.29	0.77	0.52	0.45	0.25	0.19	0.22	0.38	0.36
Pyrrole	(12,11)	0.08	0.22	0.13	0.26	0.10	0.23	0.28	0.29	0.08	0.53
Thiophene	(14,12)	0.51	0.24	0.37	0.38	0.64	0.72	0.67	0.55	0.67	0.74
Diazirine	(14,14)	0.08	0.05	0.21	0.29		0.00	0.01	0.03	0.21	
Ketene	(14,14)	0.21	0.26	0.55			0.01	0.44	0.49		
Diazomethane	(8,14)	0.04	0.05	0.02			0.14	0.07	0.07		
Hydrogen Cyaphide	(6,9)	0.20	0.22	0.11			0.18	0.38	0.63		
Imidazole	(6,9)	0.20	0.22	0.11			0.18	0.38	0.63		
Nitrosyl Hydride	(14,10)	0.07	0.04	0.04			0.12	0.08	0.47		
Nitrosomethane	(14,11)	0.08	0.07	0.07			0.16	0.01	0.25		
Cyclopropene	(12,13)	0.05	0.09				0.06	0.20			
Hydrogen Sulfide	(12,14)	0.34	0.15				0.15	0.13			
Propynal	(8,10)	0.21	0.02				0.08	0.19			
Acetaldehyde	(12,14)	0.11					0.03				
Difluorocarbene	(14,10)	0.89					0.12				
Formamide	(6,11)	0.21					0.07				
Formyl Fluoride	(12,9)	0.07					0.11				
Hydrogen Chloride	(12,14)	0.18	0.17				0.07	0.07			
Methanimine	(12,10)	0.03					0.07				
Aniline	(14,11)			0.08					0.38		
Azulene	(10,12)	0.36	0.05	0.48	0.50	0.52	0.09	0.11	0.74	0.85	0.85
Nitrobenzene	(12,12)				0.16					0.09	
Benzonitrile	(10,13)					0.27					0.84
Average		0.22	0.16	0.24	0.39	0.54	0.14	0.20	0.37	0.45	0.73

M06-HF

Molecule	Active Space	CAS-PDFT Excitation Energy Error (eV)					CASPT2 Excitation Energy Error (eV)				
		S_1	S_2	S_3	S_4	S_5	S_1	S_2	S_3	S_4	S_5
Carbon Monoxide	(6,12)	0.15	0.15	0.10	0.27	0.27	0.50	0.50	0.19	0.20	0.20
Formaldehyde	(10,8)	0.04	0.11	0.14	0.73	1.43	0.03	0.15	0.00	1.03	1.52
Pyridine	(10,10)	0.32	0.24	0.72	0.51	0.40	0.23	0.10	0.10	0.34	0.22
Pyrrole	(12,11)	0.08	0.22	0.13	0.26	0.10	0.23	0.28	0.29	0.08	0.53
Thiophene	(14,11)	0.28	0.35	0.31	0.31	0.68	0.23	0.42	0.30	0.31	0.48
Diazirine	(14,14)	0.08	0.05	0.21	0.29		0.00	0.01	0.03	0.21	
Ketene	(14,10)	0.44	0.34	0.34	2.26		0.10	0.06	0.14	1.97	
Diazomethane	(14,12)	0.21	0.36	0.47			0.01	0.00	0.04		
Hydrogen Cyaphide	(6,9)	0.02	0.11	0.11			0.16	0.14	0.15		
Imidazole	(6,9)	0.20	0.22	0.11			0.18	0.38	0.63		
Nitrosyl Hydride	(14,10)	0.07	0.04	0.04			0.12	0.08	0.47		
Nitrosomethane	(14,12)	0.05	0.08	0.07			0.10	0.12	0.17		
Cyclopropene	(12,13)	0.05	0.09				0.06	0.20			
Hydrogen Sulfide	(12,14)	0.34	0.15				0.15	0.13			
Propynal	(14,11)	0.12	0.09				0.01	0.23			
Acetaldehyde	(12,9)	0.06					0.01				
Difluorocarbene	(8,7)	1.19					0.15				
Formamide	(6,11)	0.21					0.07				
Formyl Fluoride	(10,8)	0.04					0.08				
Hydrogen Chloride	(8,14)	0.25	0.23				0.07	0.07			
Methanimine	(12,12)	0.18					0.08				
Aniline	(8,11)			0.15					0.39		
Azulene	(10,12)	0.36	0.05	0.48	0.50	0.52	0.09	0.11	0.74	0.85	0.85
Nitrobenzene	(12,12)				0.16					0.09	
Benzonitrile	(6,9)					0.60					1.23
Average		0.22	0.17	0.24	0.59	0.57	0.12	0.18	0.26	0.57	0.72

PBE1PBE

Molecule	Active Space	CAS-PDFT Excitation Energy Error (eV)					CASPT2 Excitation Energy Error (eV)				
		S_1	S_2	S_3	S_4	S_5	S_1	S_2	S_3	S_4	S_5
Carbon Monoxide	(8,8)	0.64	0.59	0.34	0.23	0.37	0.14	0.17	0.36	0.24	0.25
Formaldehyde	(10,10)	0.07	0.21	0.32	0.81	0.43	0.02	0.20	0.14	1.02	0.62
Pyridine	(14,10)	0.21	0.23	0.51	0.49	0.55	0.15	0.11	0.11	0.37	0.38
Pyrrole	(12,11)	0.08	0.22	0.13	0.26	0.10	0.23	0.28	0.29	0.08	0.53
Thiophene	(14,10)	0.30	0.29	0.19	0.34	0.70	0.38	0.20	0.33	0.57	1.19
Diazirine	(14,11)	0.24	0.00	0.21	0.24		0.04	0.05	0.10	0.24	
Ketene	(12,12)	0.30	0.32	0.19	0.67		0.11	0.06	1.14	0.09	
Diazomethane	(14,12)	0.21	0.36	0.47			0.01	0.00	0.04		
Hydrogen Cyaphide	(8,14)	0.04	0.05	0.02			0.14	0.07	0.07		
Imidazole	(12,12)	0.16	0.18	0.05			0.15	0.35	0.57		
Nitrosyl Hydride	(14,10)	0.07	0.04	0.04			0.12	0.08	0.47		
Nitrosomethane	(6,12)	0.03	0.07	0.08			0.10	0.11	0.09		
Cyclopropene	(12,13)	0.05	0.09				0.06	0.20			
Hydrogen Sulfide	(8,14)	0.27	0.01				0.14	0.19			
Propynal	(8,10)	0.21	0.02				0.08	0.19			
Acetaldehyde	(12,14)	0.11					0.03				
Difluorocarbene	(14,10)	0.89					0.12				
Formamide	(6,11)	0.21					0.07				
Formyl Fluoride	(14,10)	0.11					0.11				
Hydrogen Chloride	(8,12)	0.51	0.49				0.21	0.21			
Methanimine	(14,10)	0.01					0.05				
Aniline	(10,13)			0.11					0.44		
Azulene	(12,14)	0.34	0.07	0.43	0.45	0.49	0.06	0.03	0.70	0.82	0.82
Nitrobenzene	(6,14)				0.09					0.16	
Benzonitrile	(6,13)					0.51					0.53
Average		0.23	0.19	0.22	0.40	0.45	0.12	0.15	0.35	0.40	0.62

Plots of CAS-PDFT Excitation Energy Error w.r.t. S_n (n>0) CASSCF Dipole Moment Error at All Active Spaces; Application of EDM-AS

All active space sizes considered in this work (PASS+) are shown in the plots although only active spaces in PASS will be chosen by EDM-AS. Active spaces chosen by EDM-AS are labeled with green crosses. TD-M06-2X is used to provide reference excited-state dipole moments.

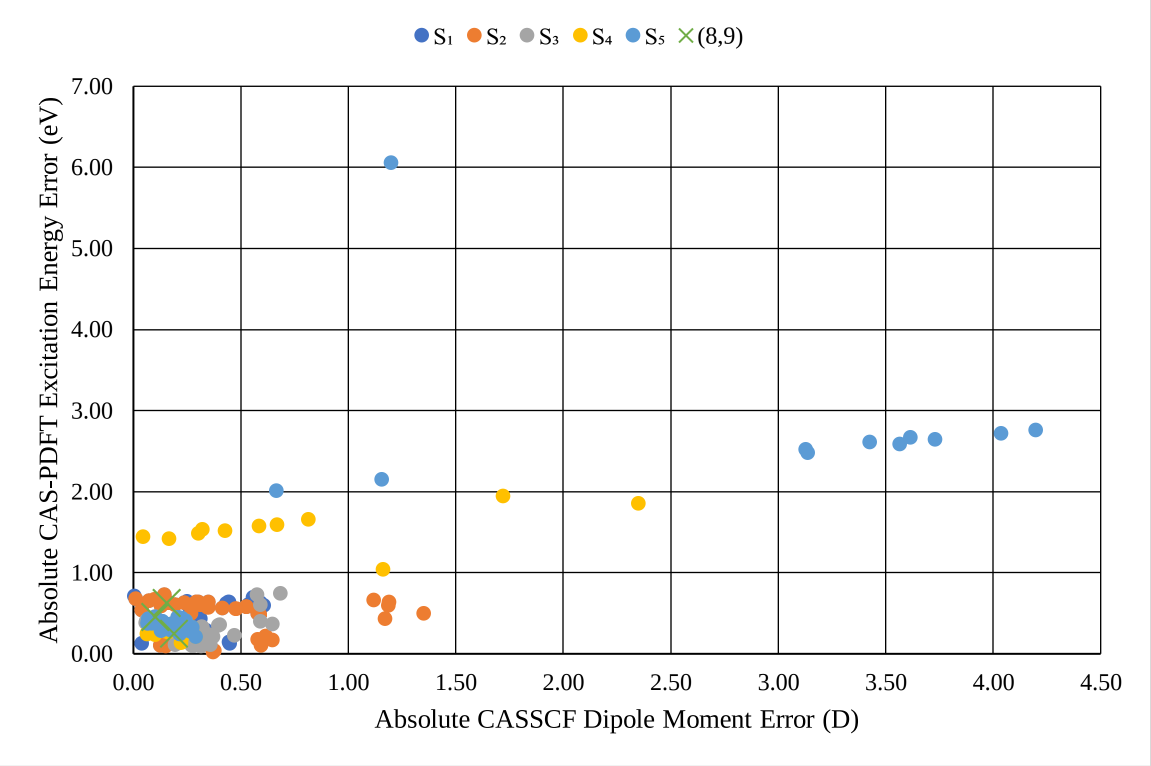

Figure S55: Plot of CAS-PDFT excitation energy error with respect to excited-state CASSCF dipole moment error for carbon monoxide.

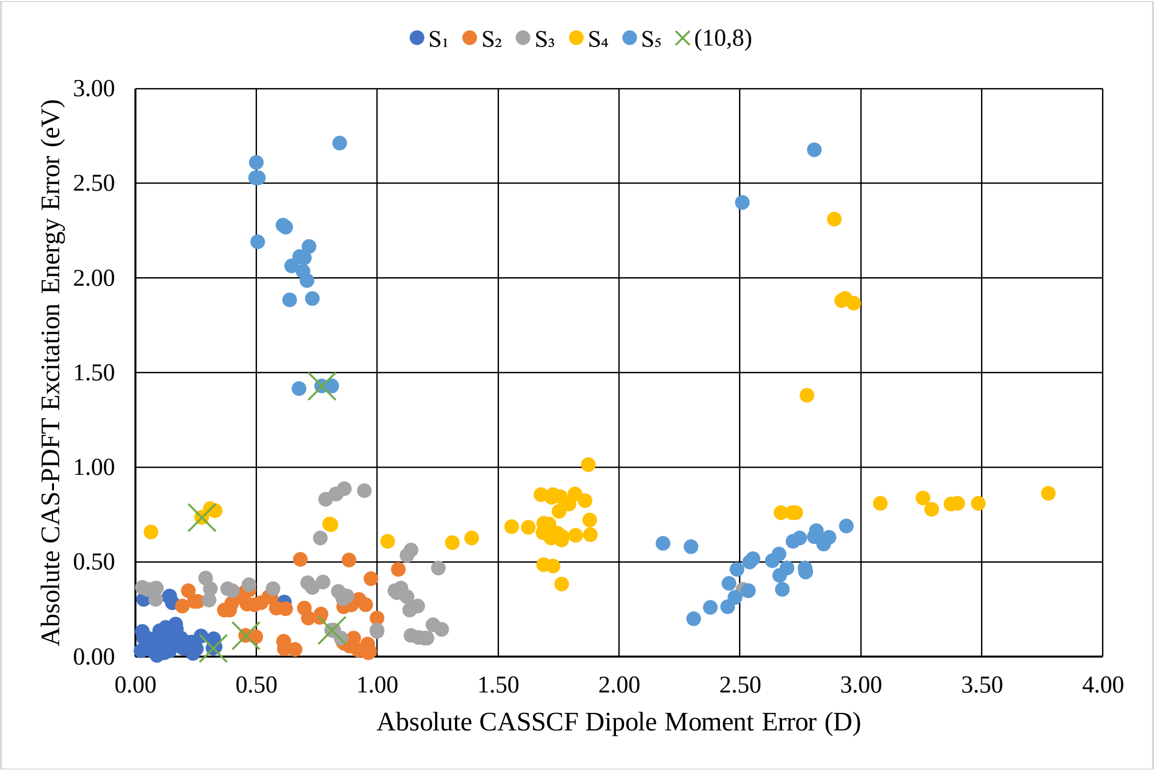

Figure S56: Plot of CAS-PDFT excitation energy error with respect to excited-state CASSCF dipole moment error for formaldehyde.

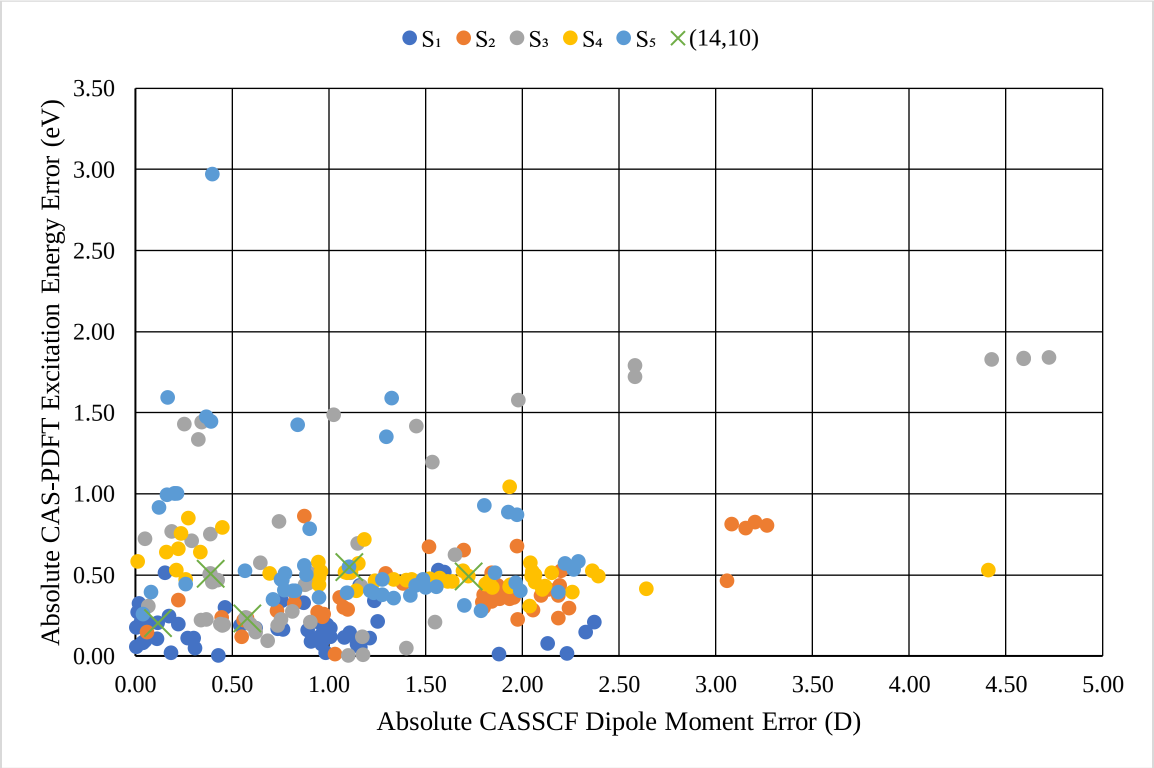

Figure S57: Plot of CAS-PDFT excitation energy error with respect to excited-state CASSCF dipole moment error for pyridine.

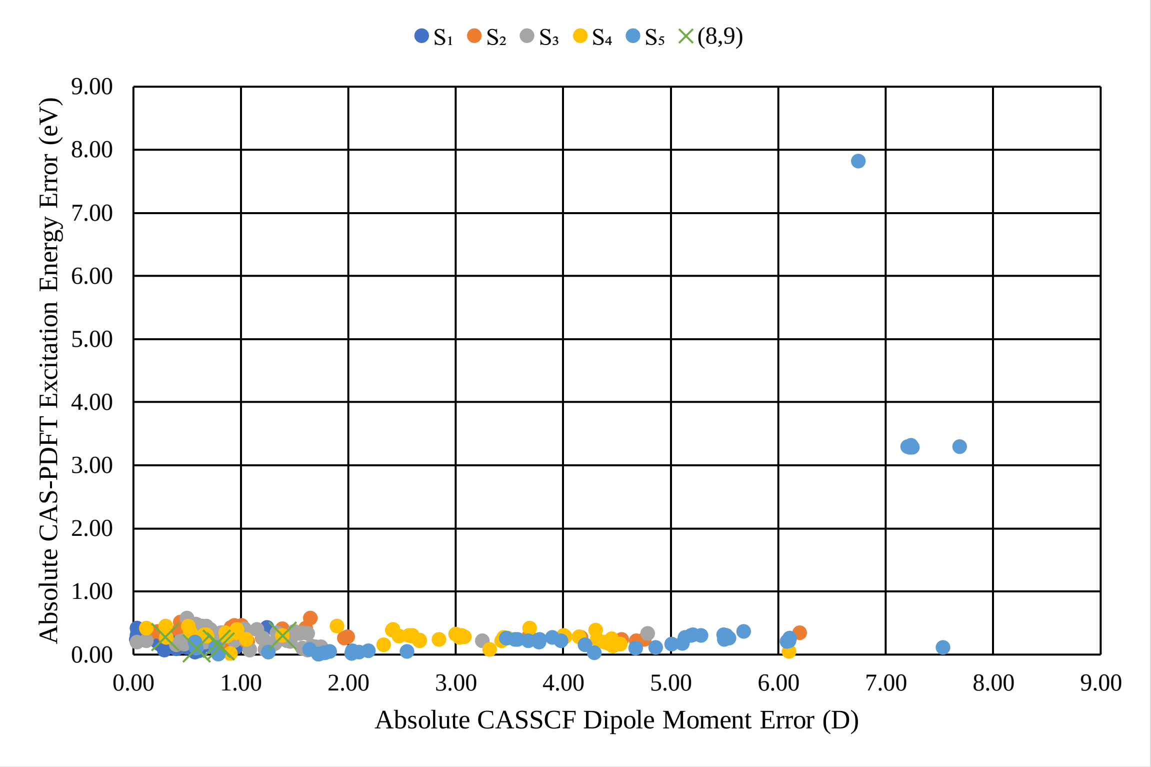

Figure S58: Plot of CAS-PDFT excitation energy error with respect to excited-state CASSCF dipole moment error for pyrrole.

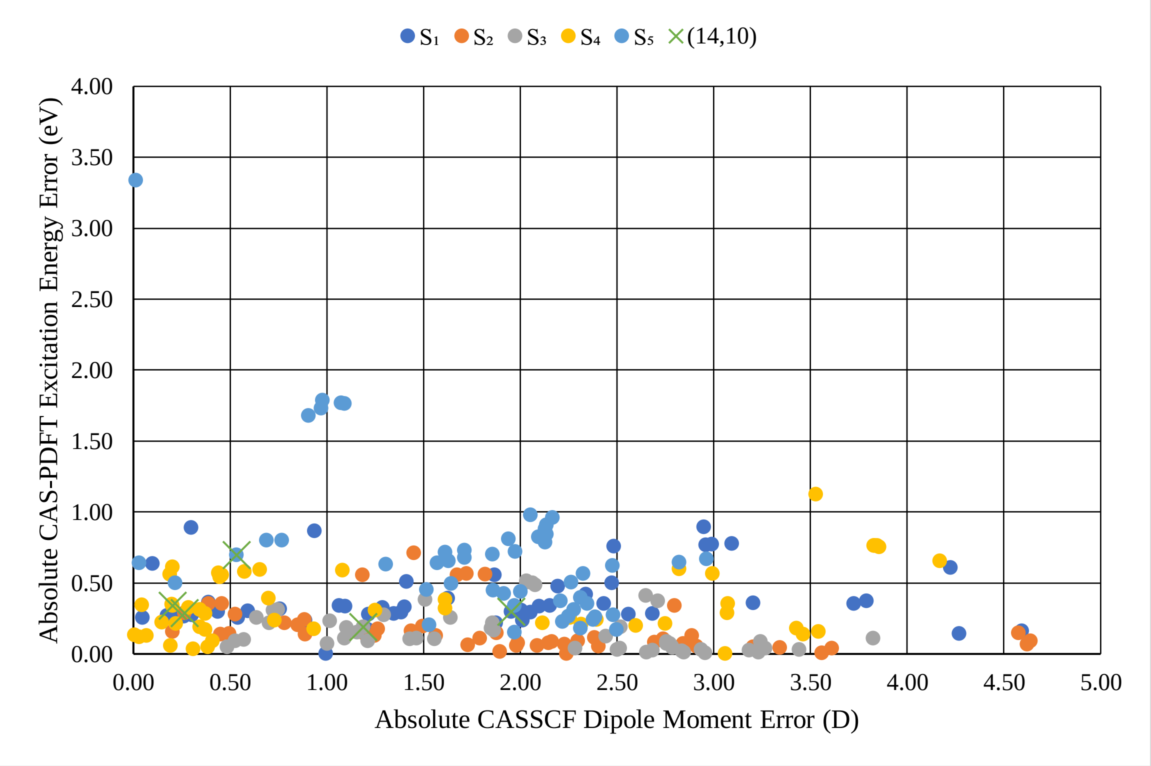

Figure S59: Plot of CAS-PDFT excitation energy error with respect to excited-state CASSCF dipole moment error for thiophene.

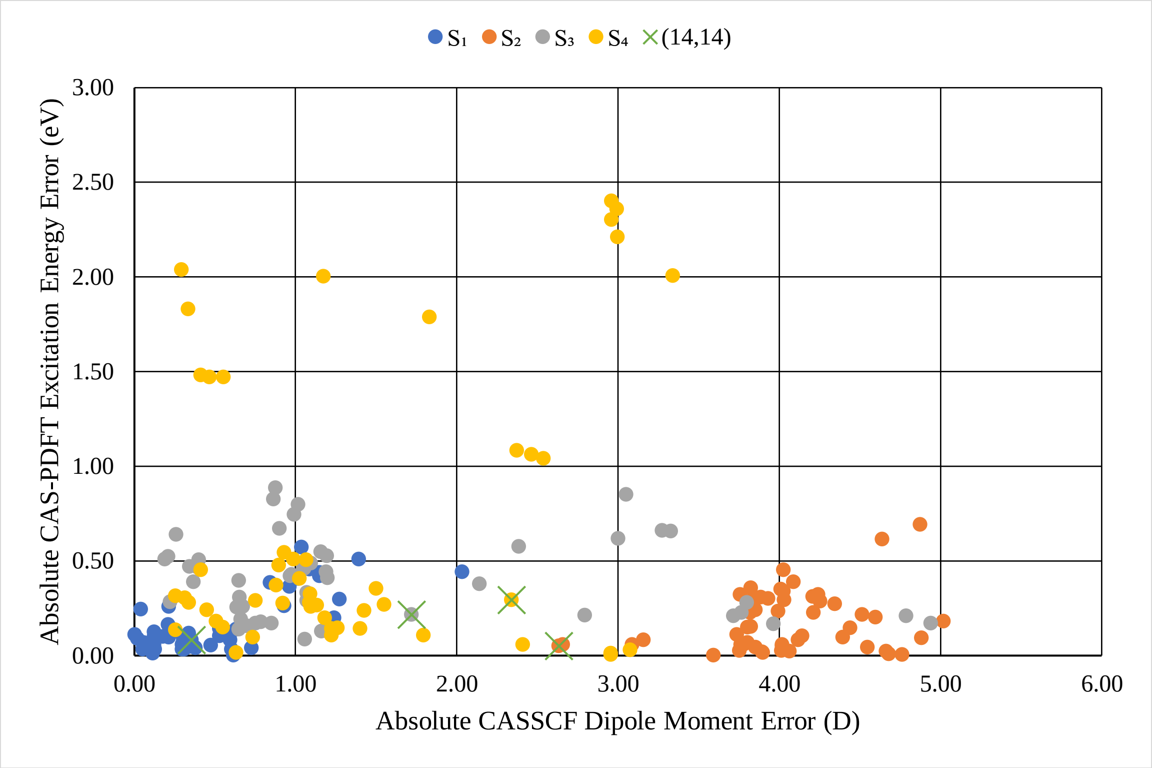

Figure S60: Plot of CAS-PDFT excitation energy error with respect to excited-state CASSCF dipole moment error for diazirine.

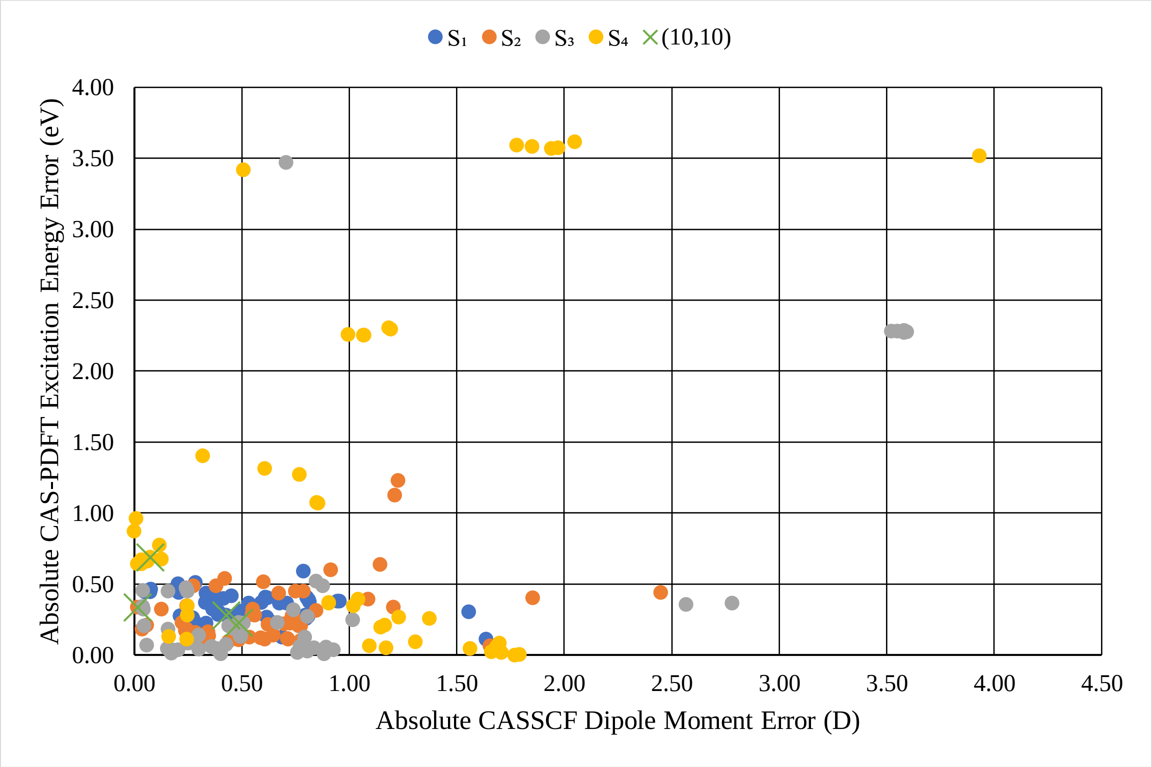

Figure S61: Plot of CAS-PDFT excitation energy error with respect to excited-state CASSCF dipole moment error for ketene.

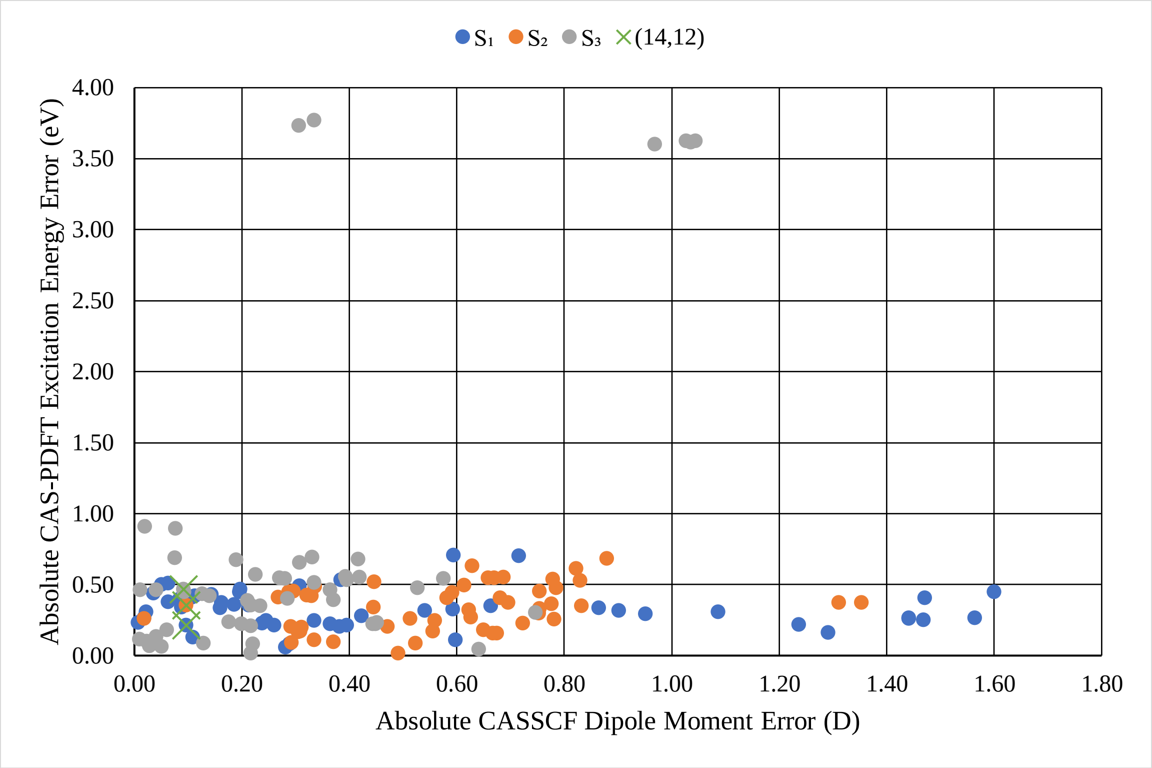

Figure S62: Plot of CAS-PDFT excitation energy error with respect to excited-state CASSCF dipole moment error for diazomethane.

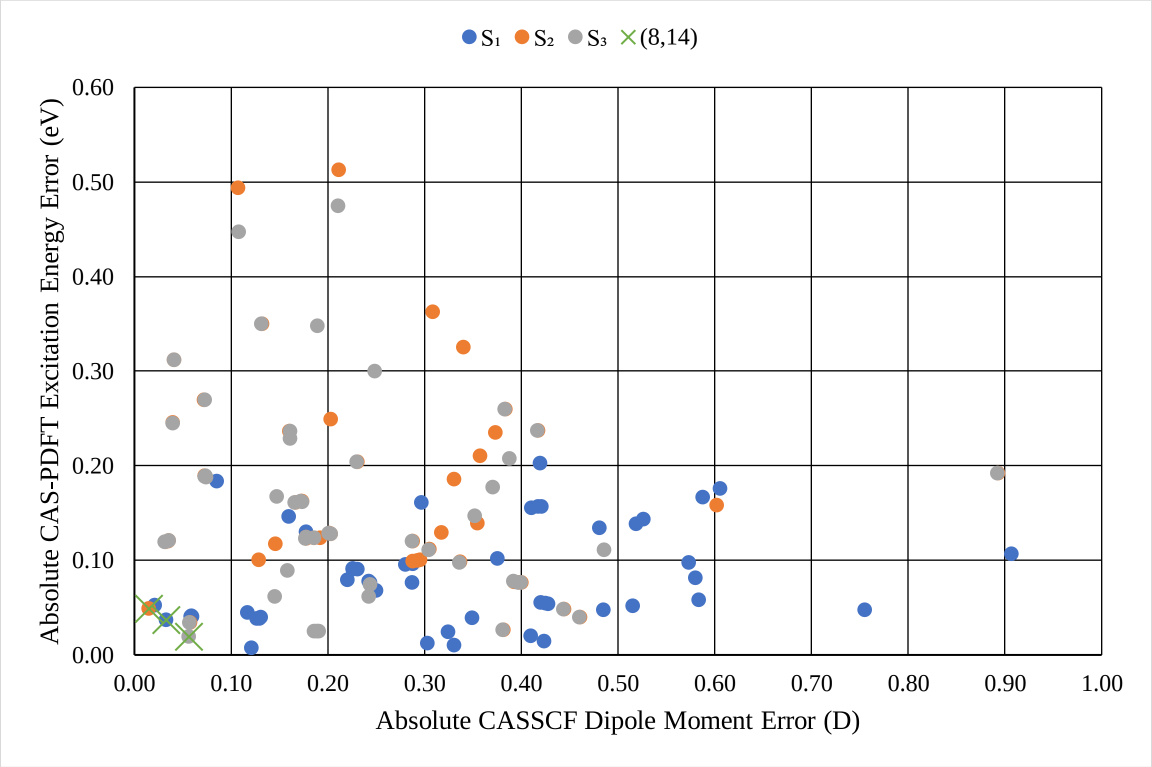

Figure S63: Plot of CAS-PDFT excitation energy error with respect to excited-state CASSCF dipole moment error for hydrogen cyaphide.

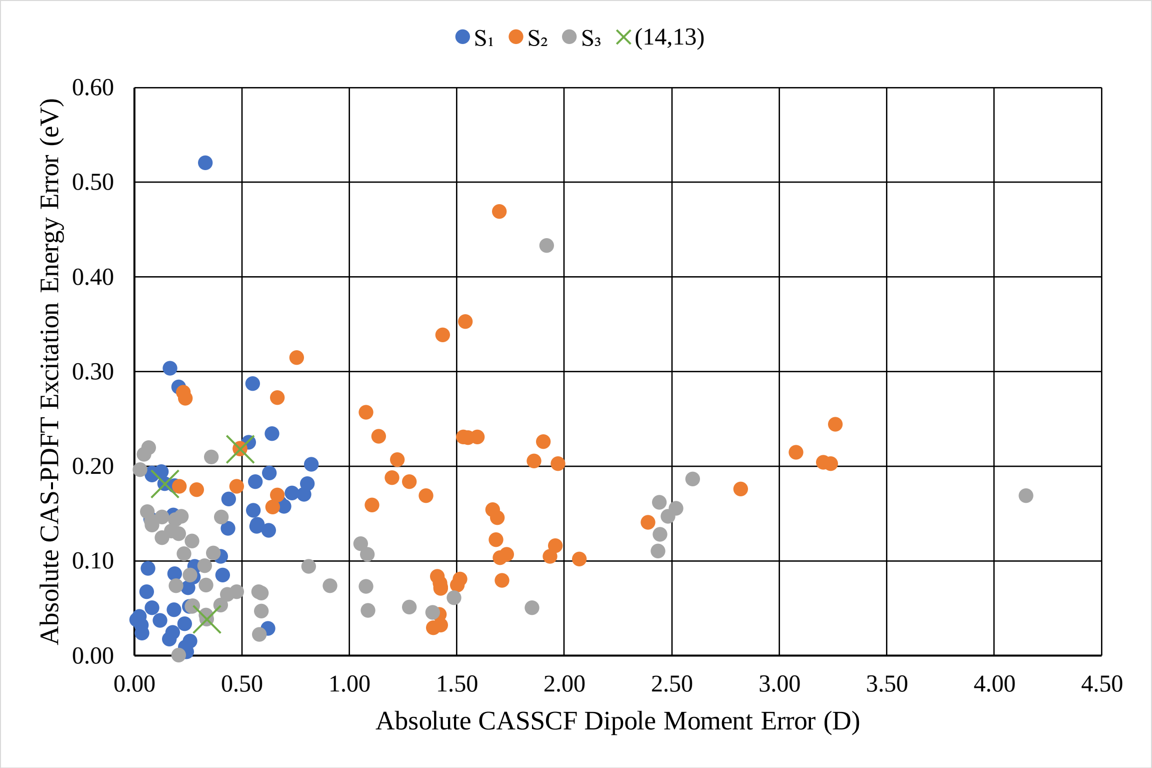

Figure S64: Plot of CAS-PDFT excitation energy error with respect to excited-state CASSCF dipole moment error for imidazole.

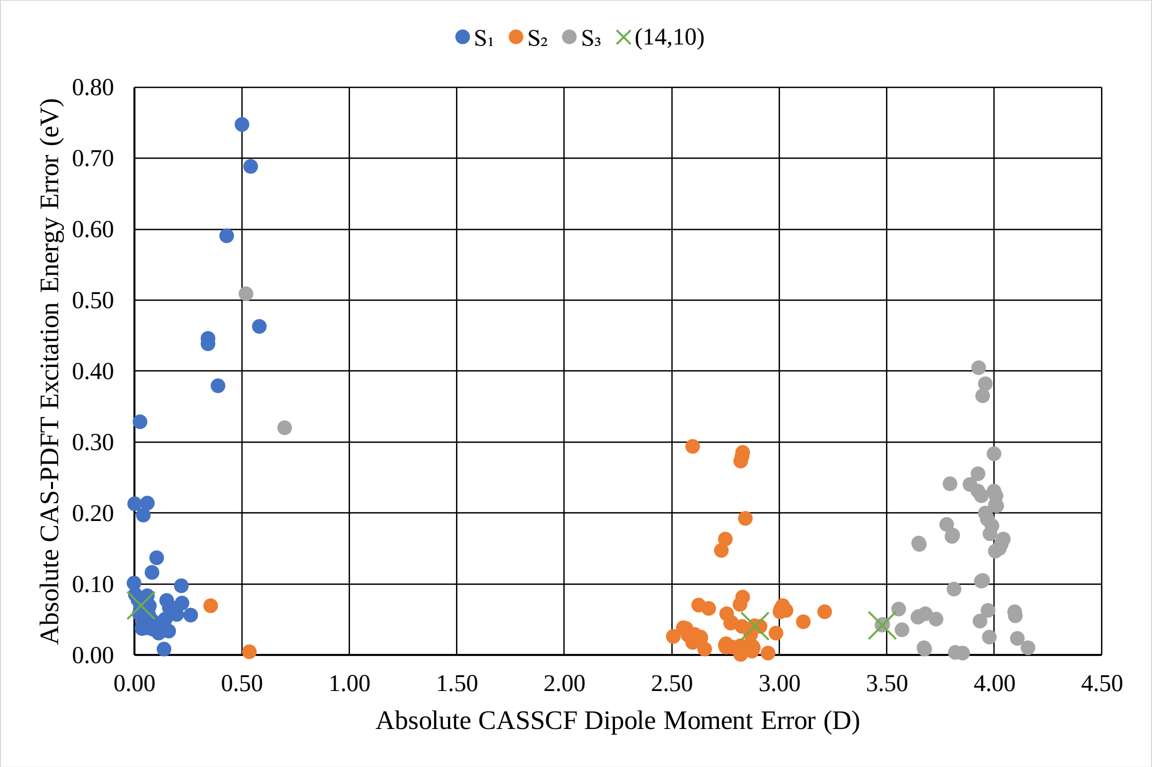

Figure S65: Plot of CAS-PDFT excitation energy error with respect to excited-state CASSCF dipole moment error for nitrosyl hydride.

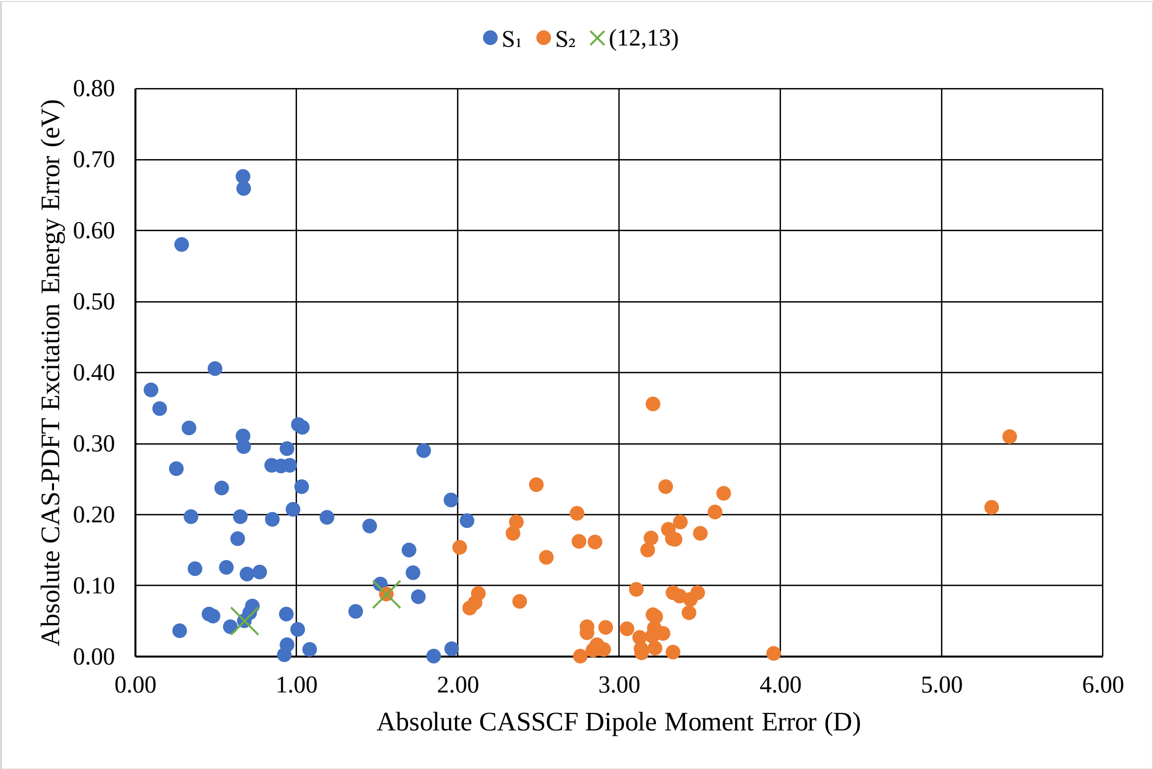

Figure S66: Plot of CAS-PDFT excitation energy error with respect to excited-state CASSCF dipole moment error for cyclopropene.

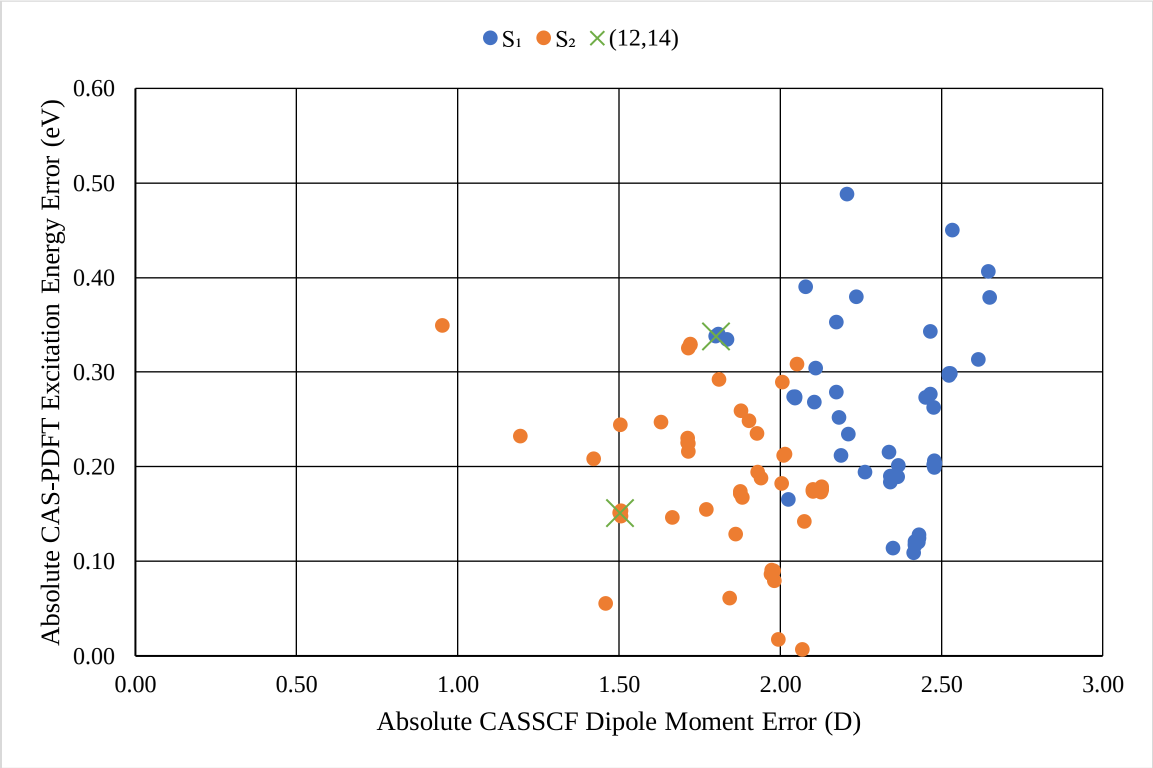

Figure S67: Plot of CAS-PDFT excitation energy error with respect to excited-state CASSCF dipole moment error for hydrogen sulfide.

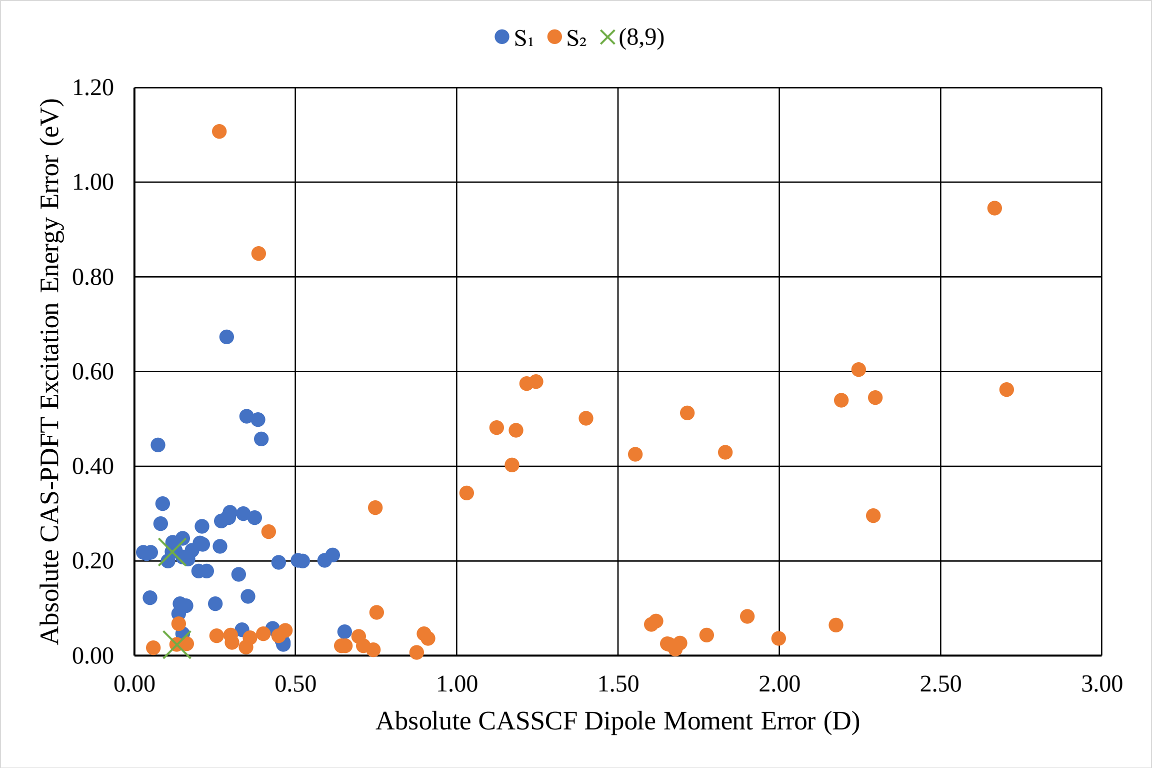

Figure S68: Plot of CAS-PDFT excitation energy error with respect to excited-state CASSCF dipole moment error for propynal.

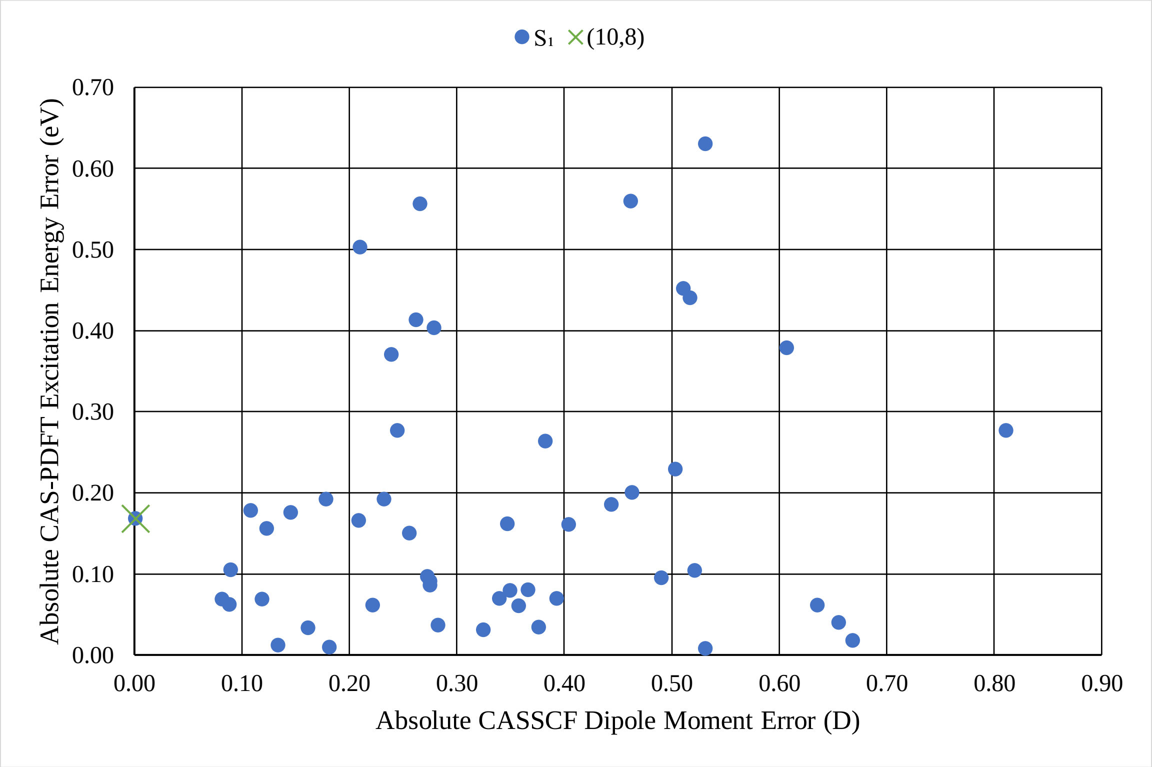

Figure S69: Plot of CAS-PDFT excitation energy error with respect to excited-state CASSCF dipole moment error for acetaldehyde.

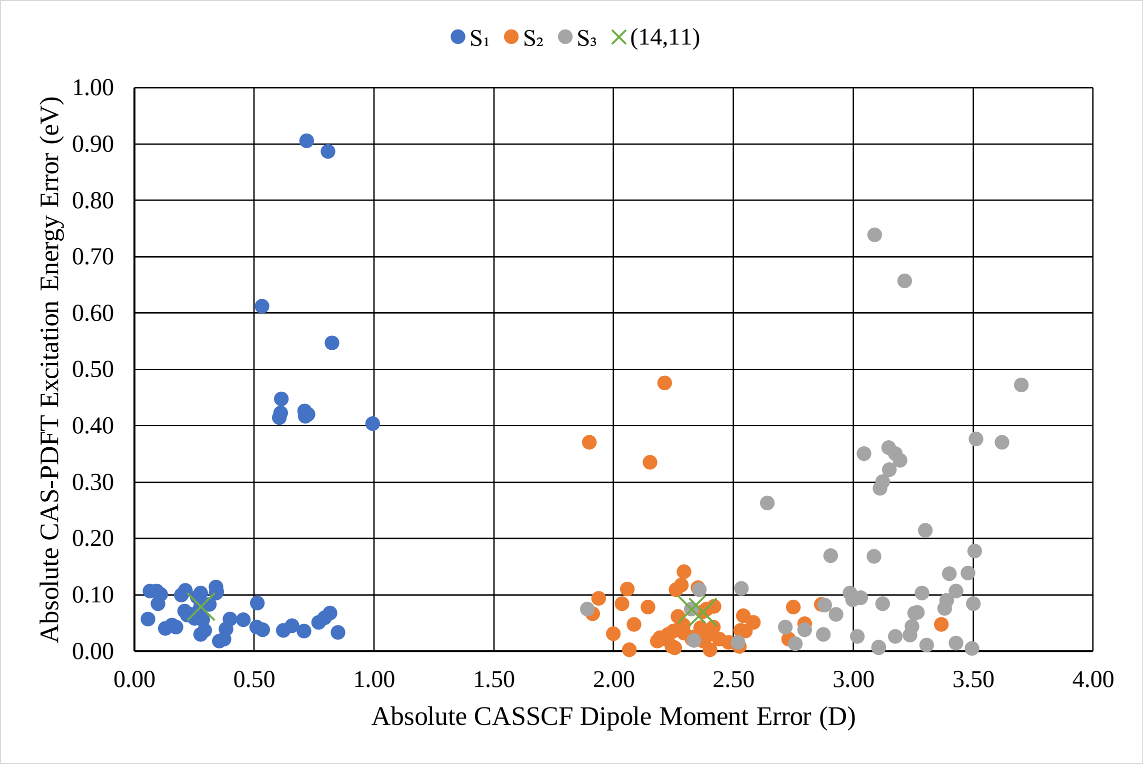

Figure S70: Plot of CAS-PDFT excitation energy error with respect to excited-state CASSCF dipole moment error for nitrosomethane.

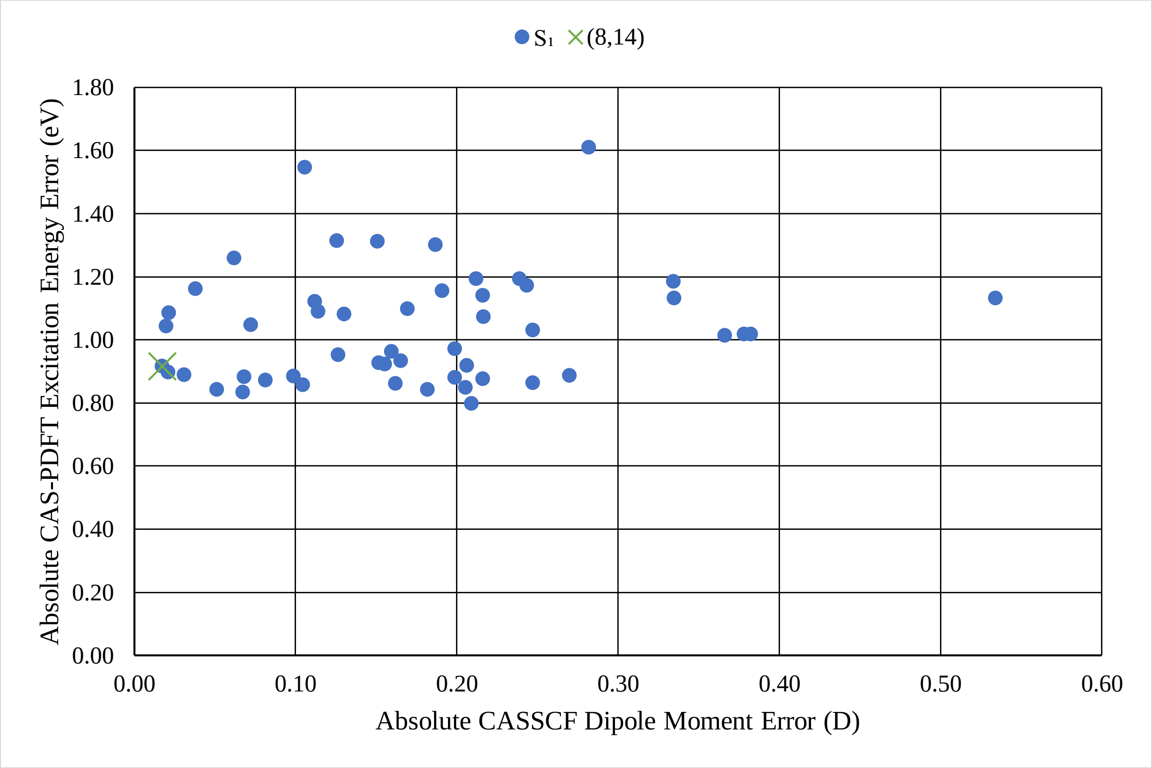

Figure S71: Plot of CAS-PDFT excitation energy error with respect to excited-state CASSCF dipole moment error for difluorocarbene.

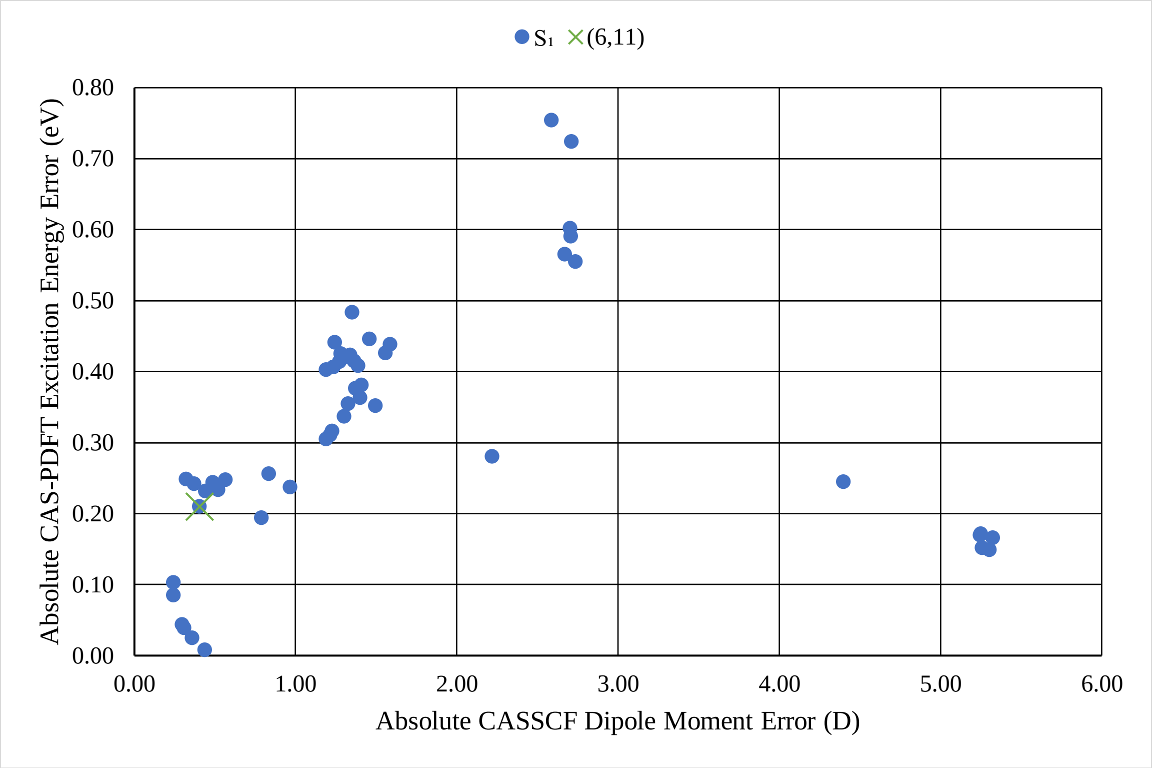

Figure S72: Plot of CAS-PDFT excitation energy error with respect to excited-state CASSCF dipole moment error for formamide.

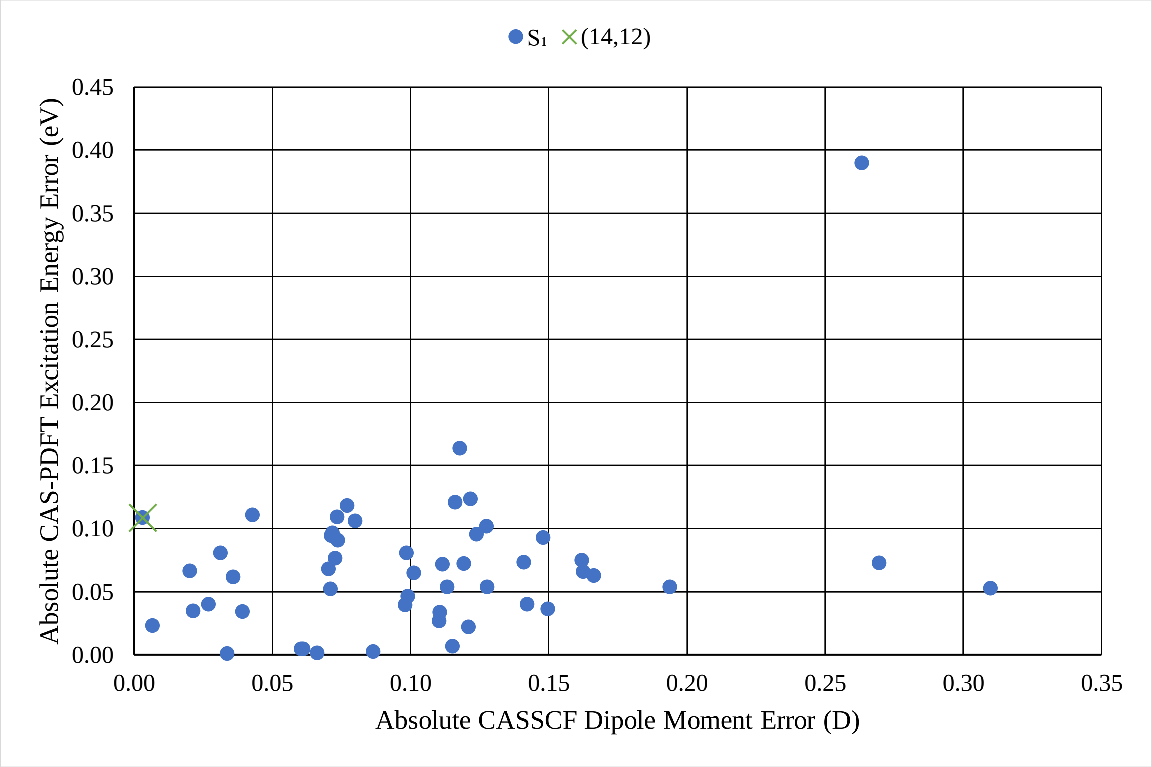

Figure S73: Plot of CAS-PDFT excitation energy error with respect to excited-state CASSCF dipole moment error for formyl fluoride.

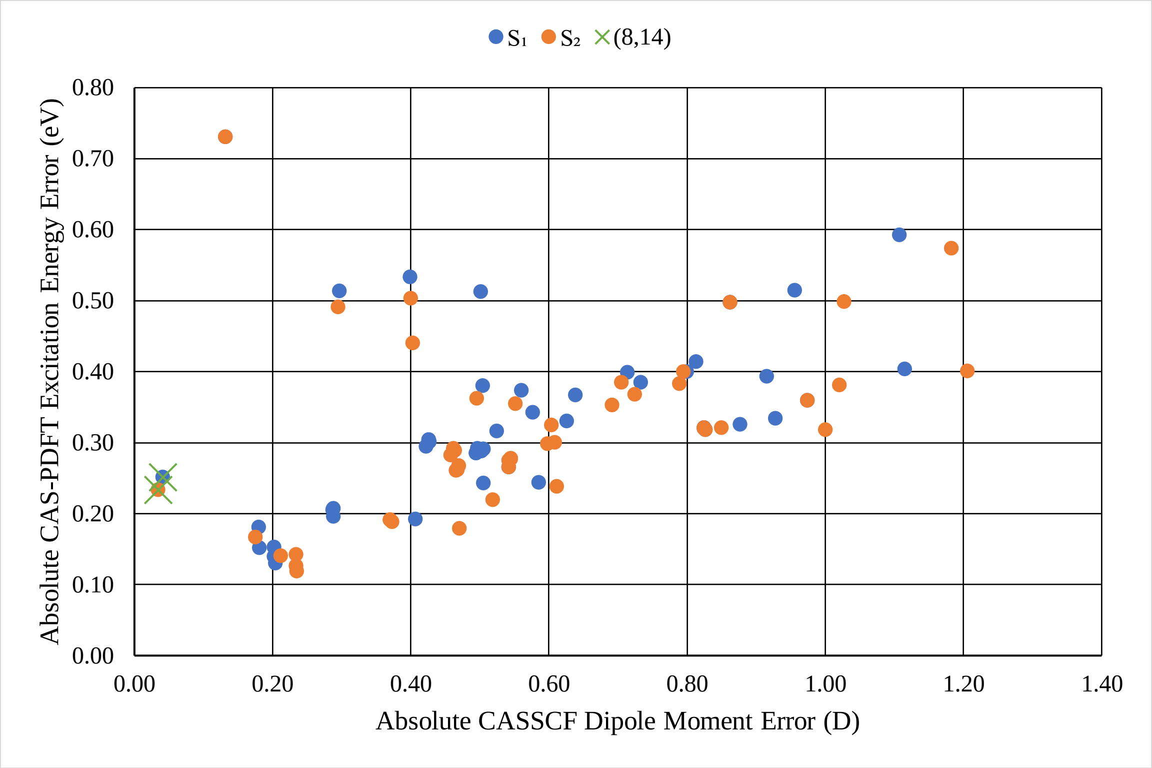

Figure S74: Plot of CAS-PDFT excitation energy error with respect to excited-state CASSCF dipole moment error for hydrogen chloride.

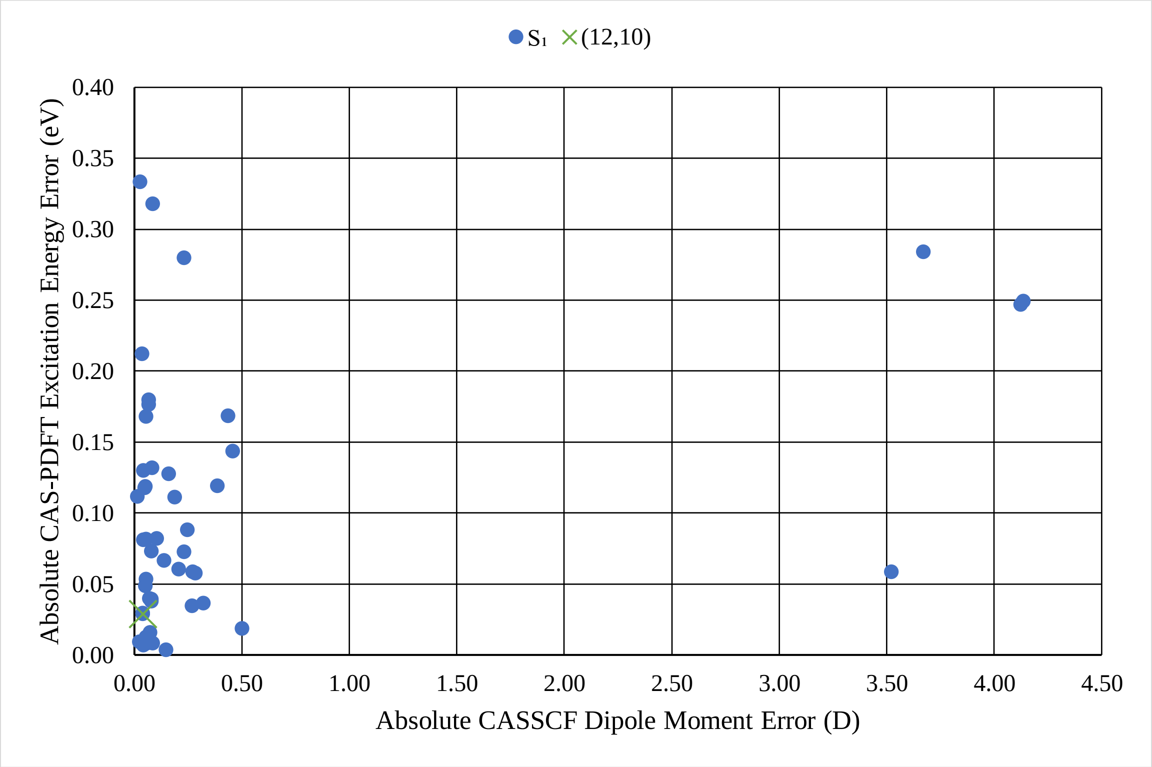

Figure S75: Plot of CAS-PDFT excitation energy error with respect to excited-state CASSCF dipole moment error for methanimine.

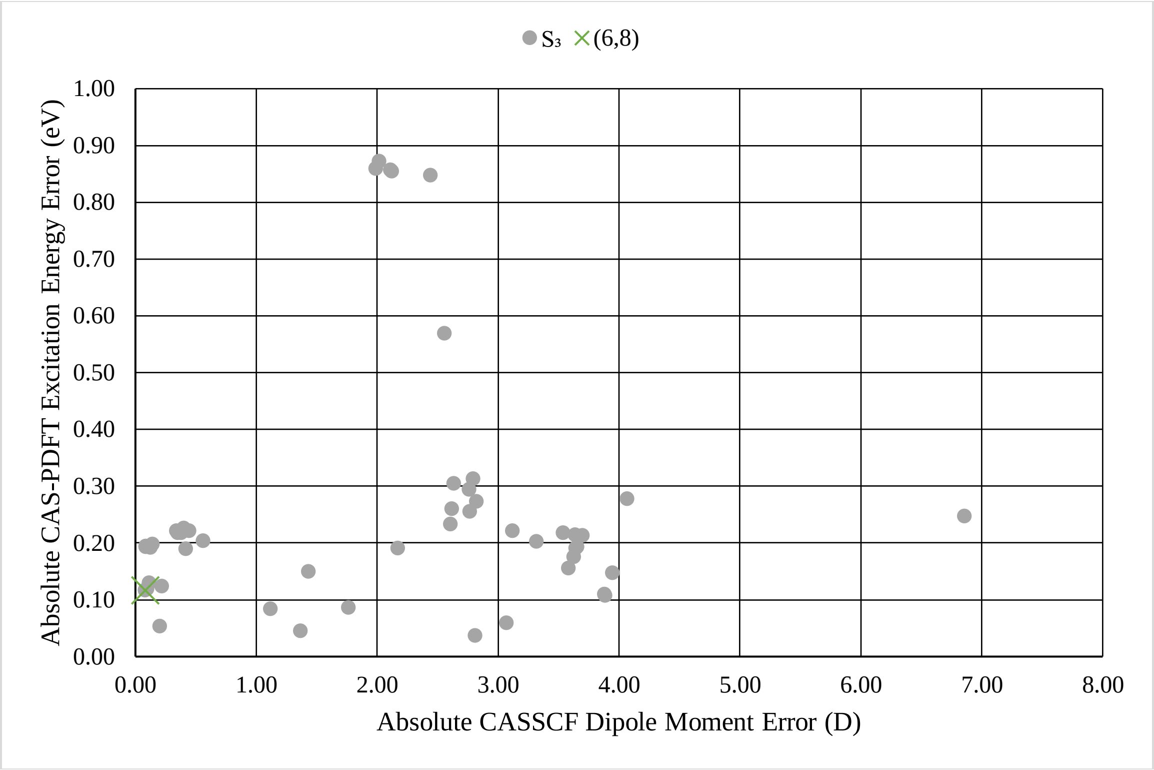

Figure S76: Plot of CAS-PDFT excitation energy error with respect to excited-state CASSCF dipole moment error for aniline.

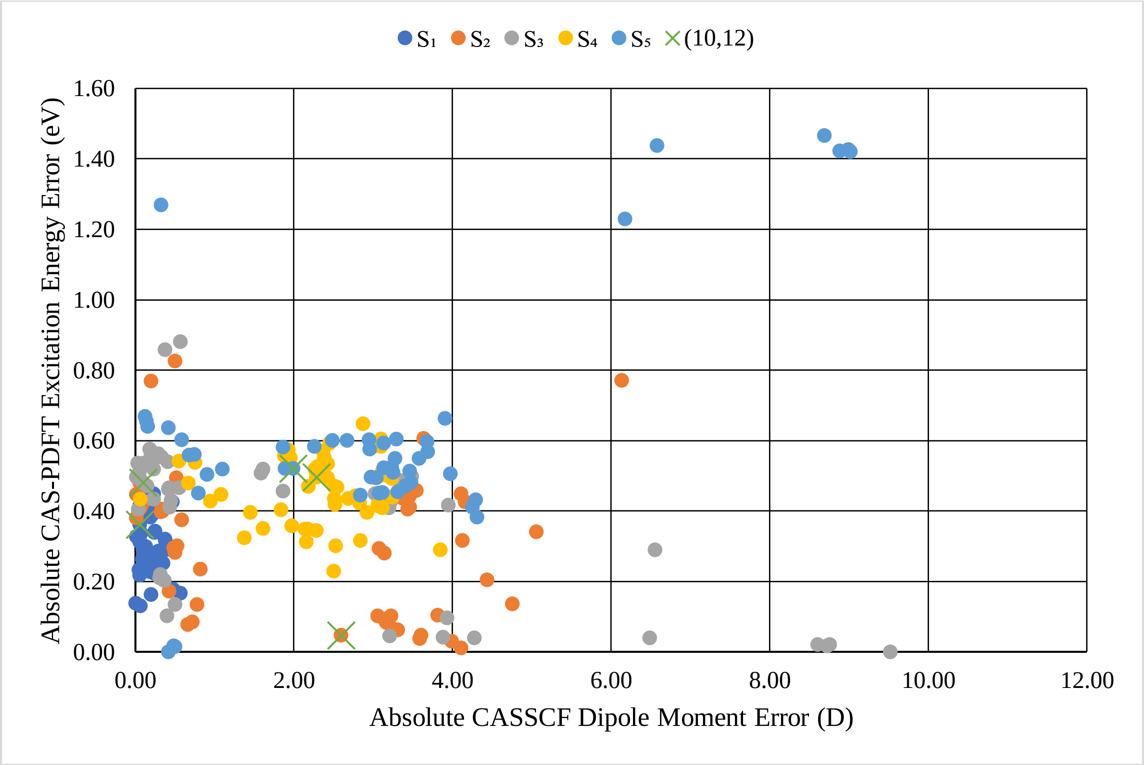

Figure S77: Plot of CAS-PDFT excitation energy error with respect to excited-state CASSCF dipole moment error for azulene.

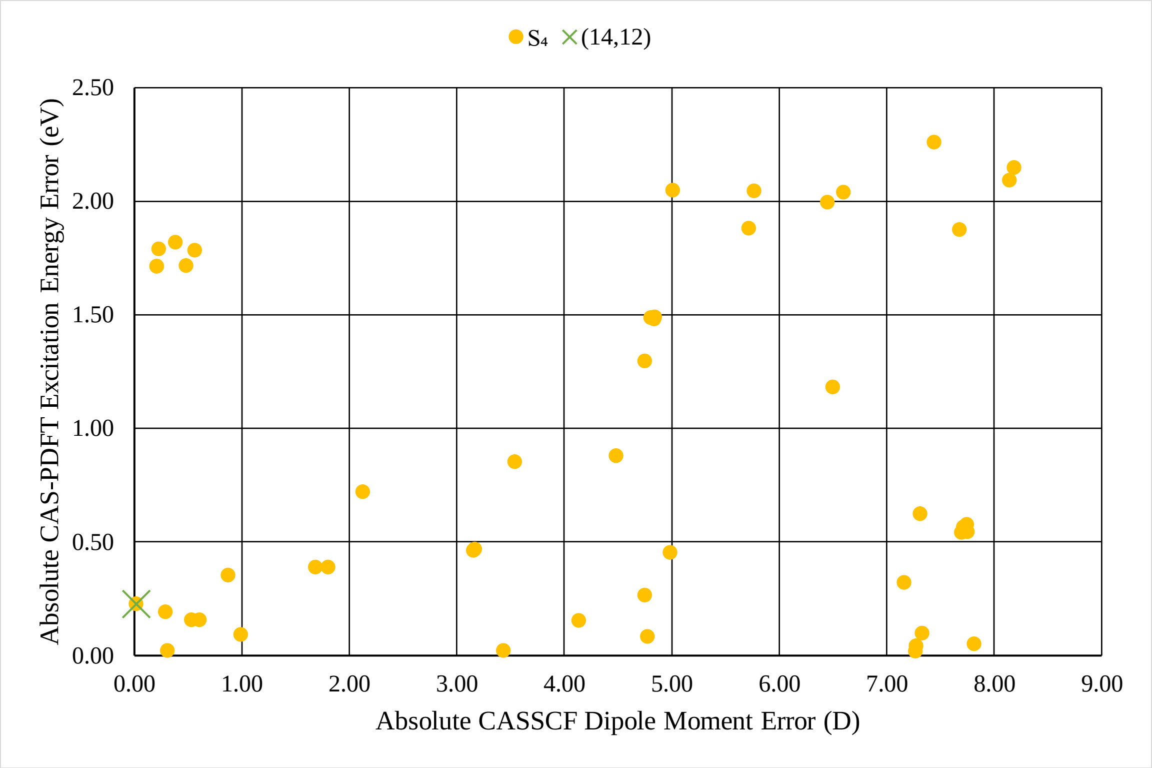

Figure S78: Plot of CAS-PDFT excitation energy error with respect to excited-state CASSCF dipole moment error for nitrobenzene.

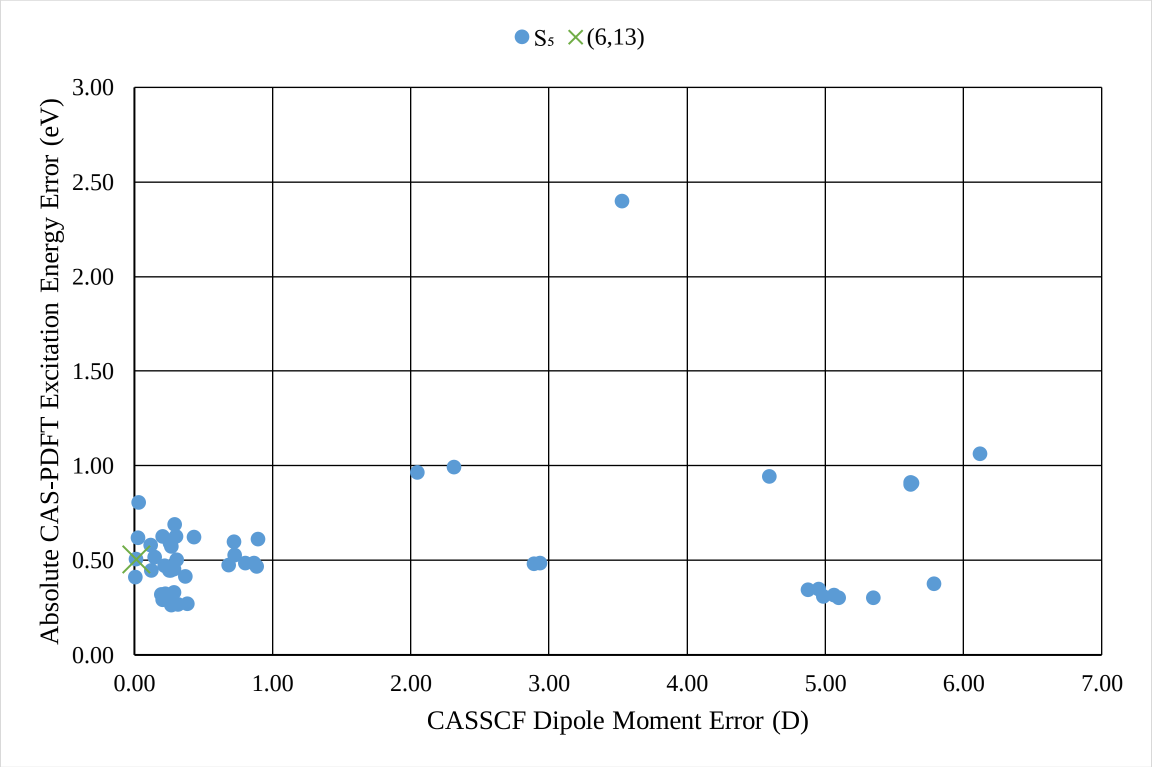

Figure S79: Plot of CAS-PDFT excitation energy error with respect to excited-state CASSCF dipole moment error for benzonitrile.

Plots of CASPT2 Excitation Energy Error w.r.t. S_n (n>0) CASSCF Dipole Moment Error at All Active Spaces; Application of EDM-AS

All active space sizes considered in this work (PASS+) are shown in the plots although only active spaces in PASS will be chosen by EDM-AS. Active spaces chosen by EDM-AS are labeled with green crosses.

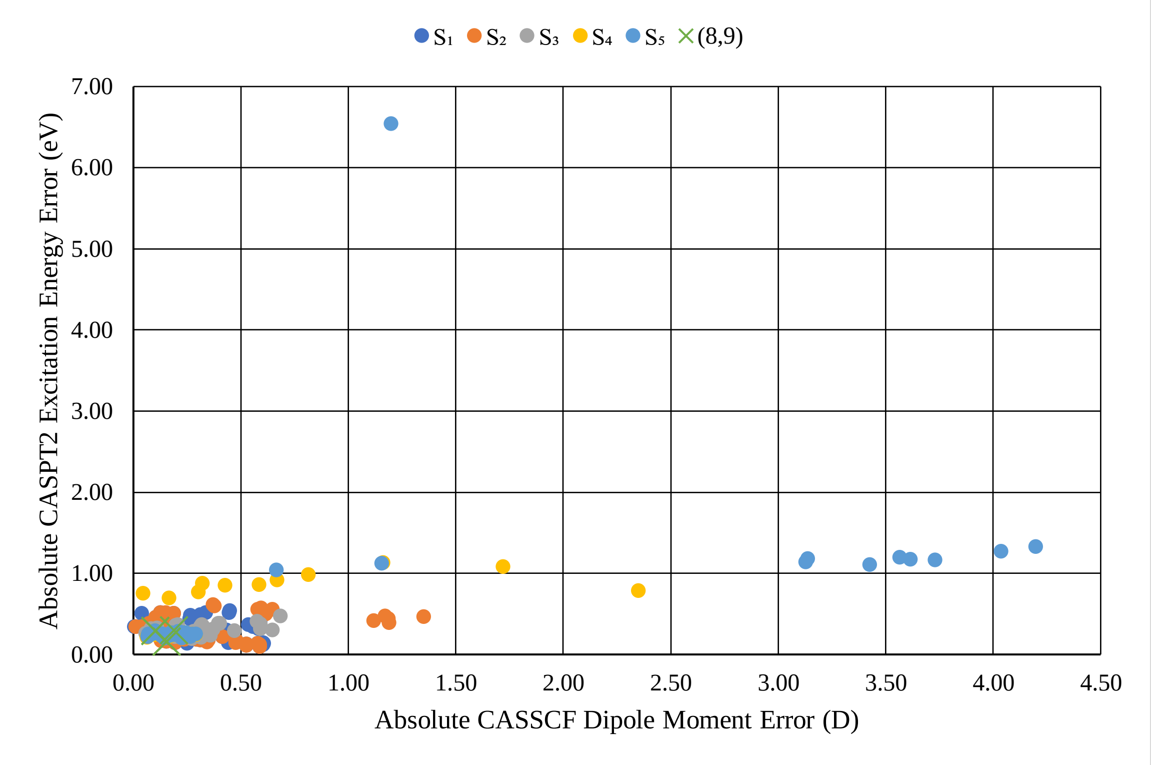

Figure S80: Plot of CASPT2 excitation energy error with respect to excited-state CASSCF dipole moment error for carbon monoxide.

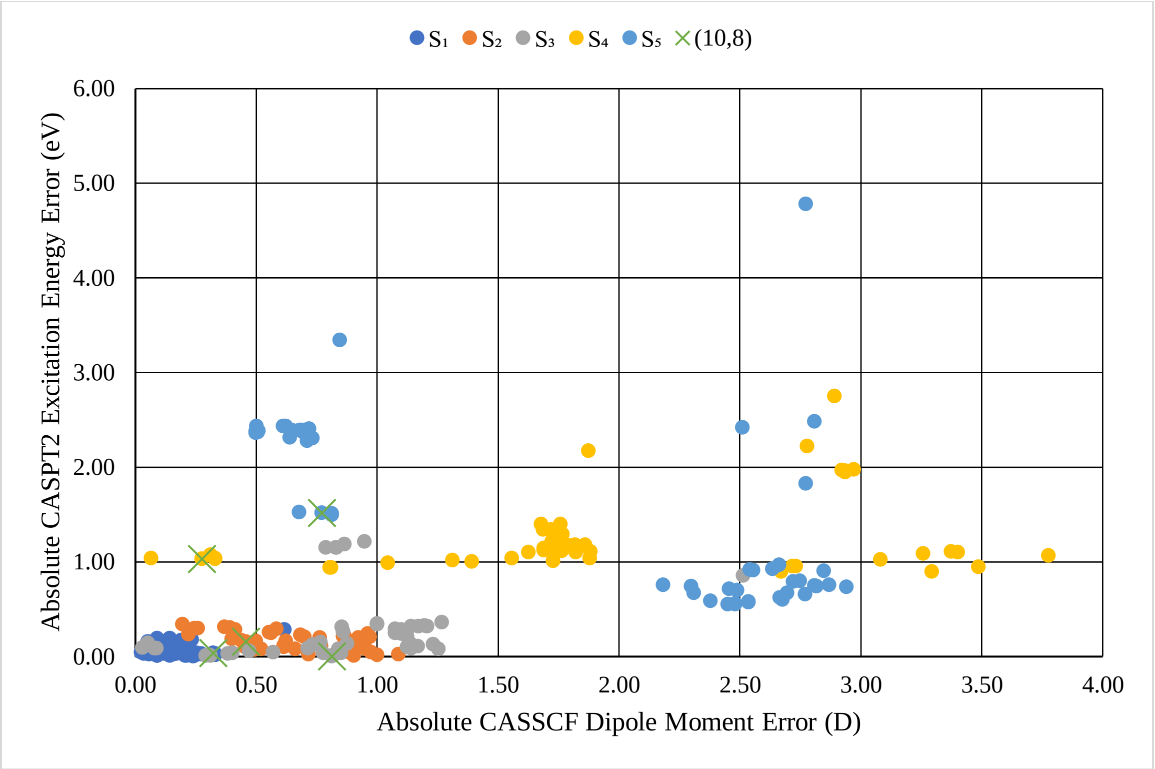

Figure S81: Plot of CASPT2 excitation energy error with respect to excited-state CASSCF dipole moment error for formaldehyde.

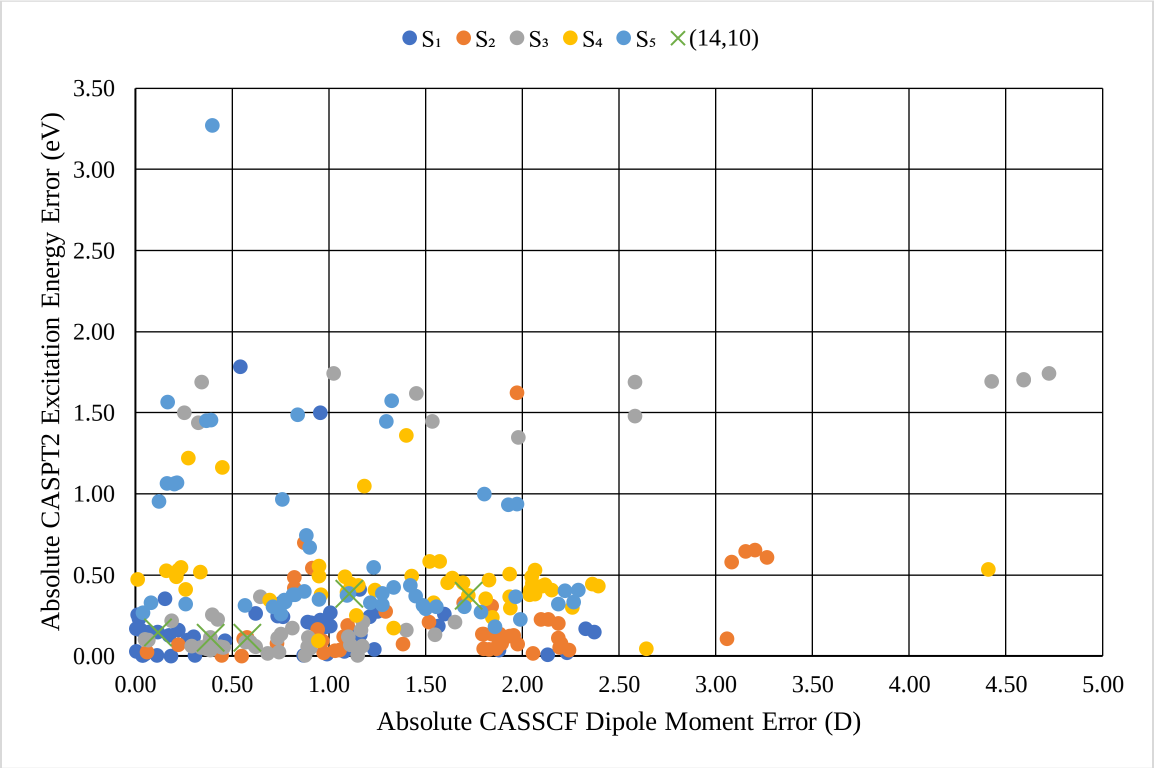

Figure S82: Plot of CASPT2 excitation energy error with respect to excited-state CASSCF dipole moment error for pyridine.

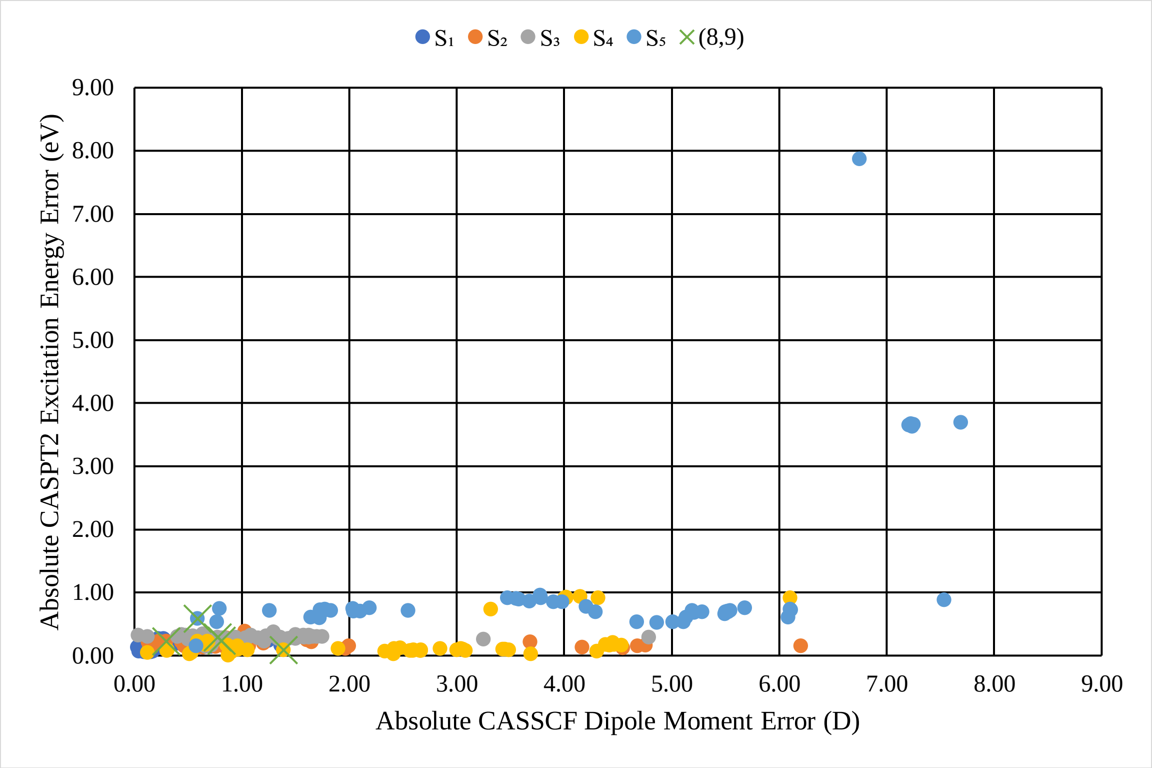

Figure S83: Plot of CASPT2 excitation energy error with respect to excited-state CASSCF dipole moment error for pyrrole.

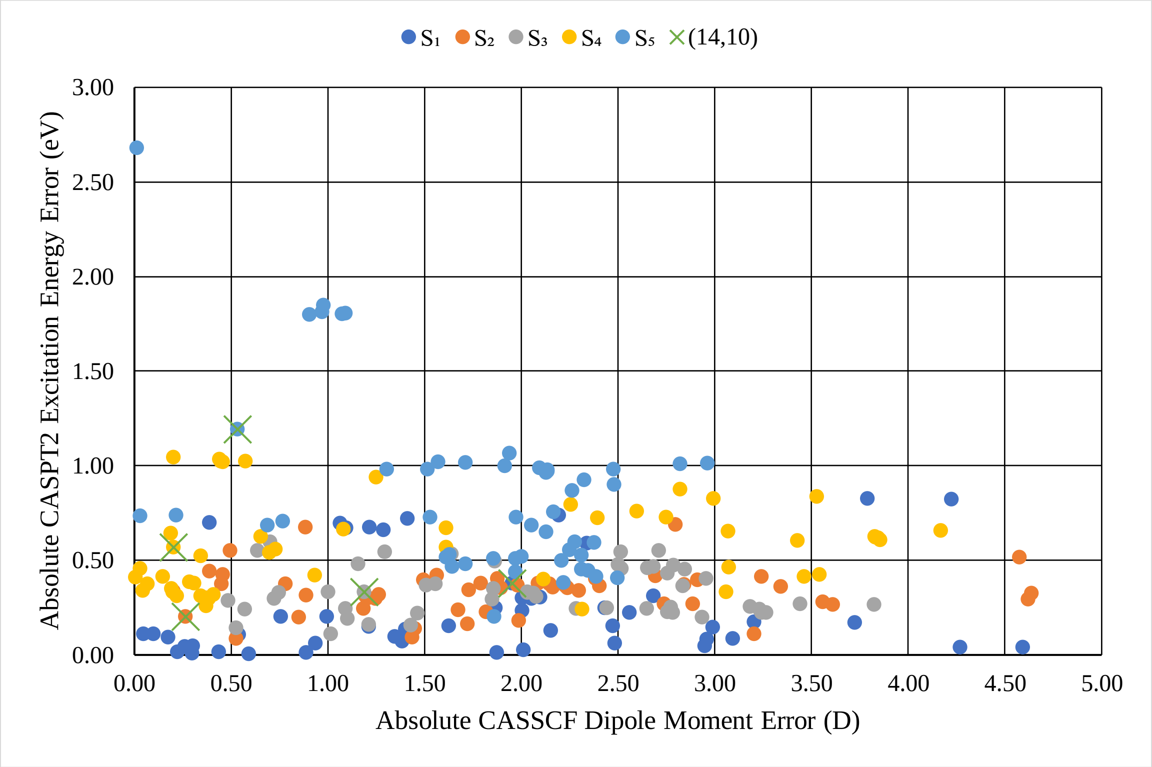

Figure S84: Plot of CASPT2 excitation energy error with respect to excited-state CASSCF dipole moment error for thiophene.

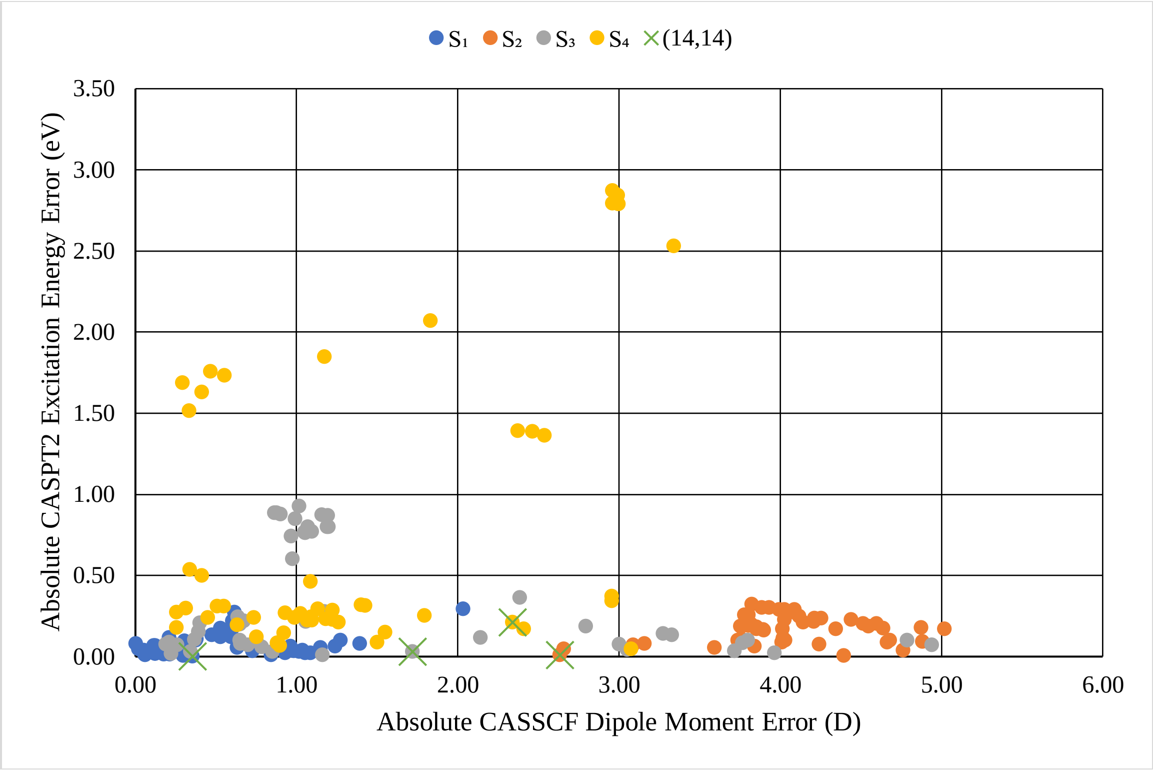

Figure S85: Plot of CASPT2 excitation energy error with respect to excited-state CASSCF dipole moment error for diazirine.

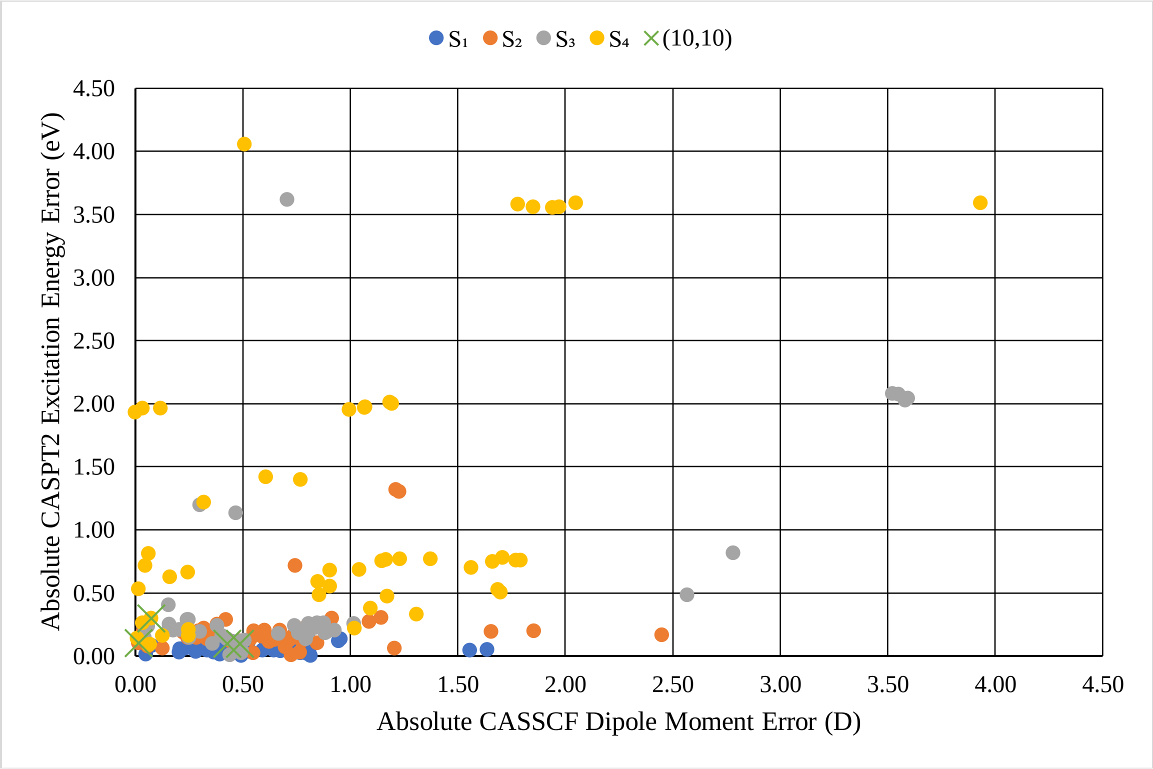

Figure S86: Plot of CASPT2 excitation energy error with respect to excited-state CASSCF dipole moment error for ketene.

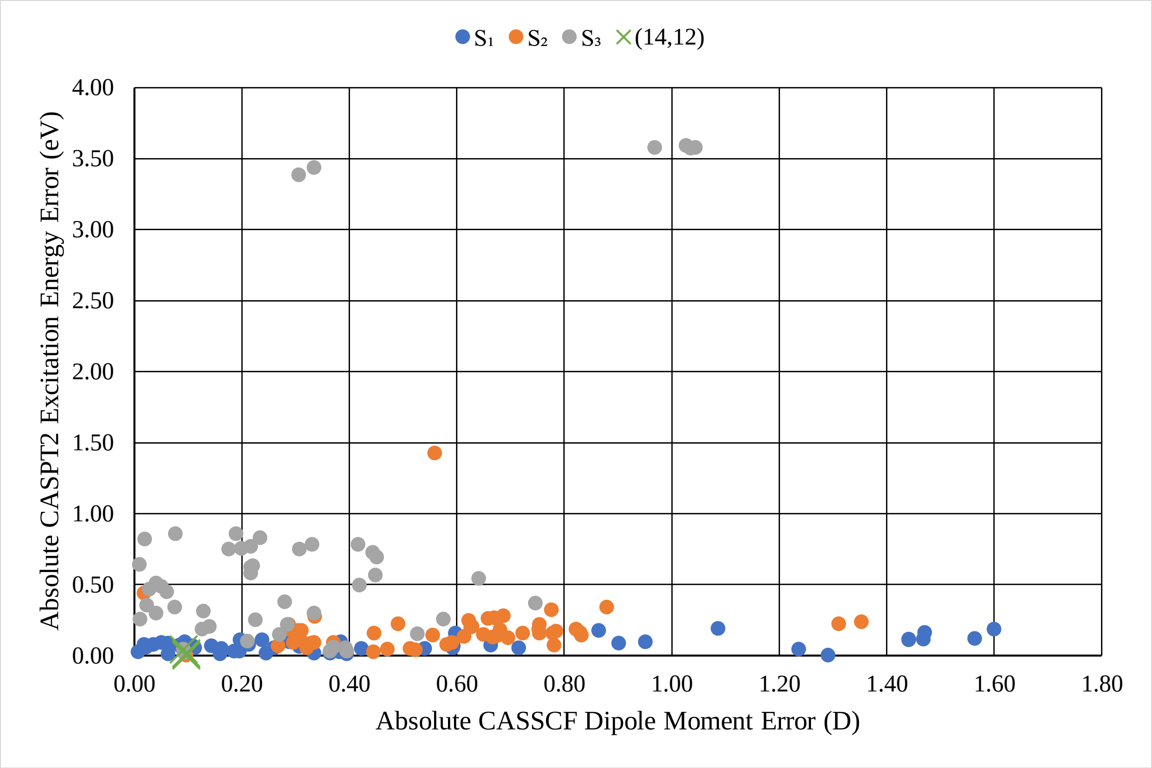

Figure S87: Plot of CASPT2 excitation energy error with respect to excited-state CASSCF dipole moment error for diazomethane.

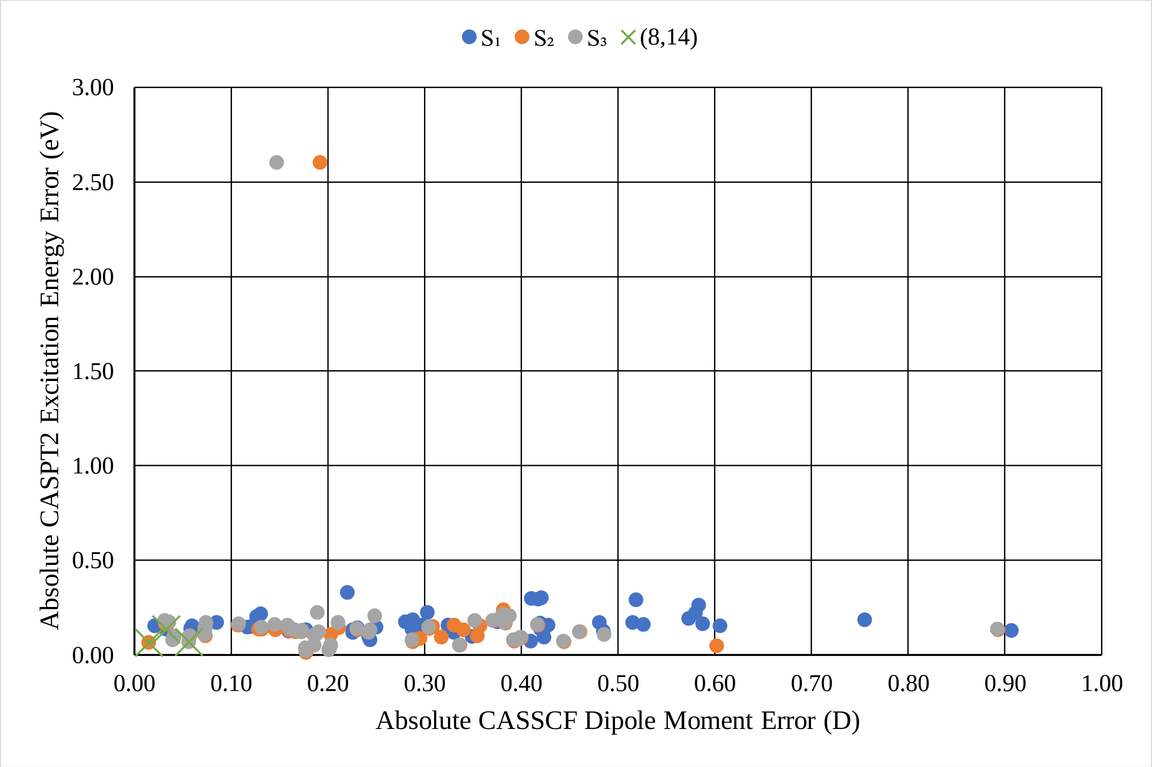

Figure S88: Plot of CASPT2 excitation energy error with respect to excited-state CASSCF dipole moment error for hydrogen cyaphide.

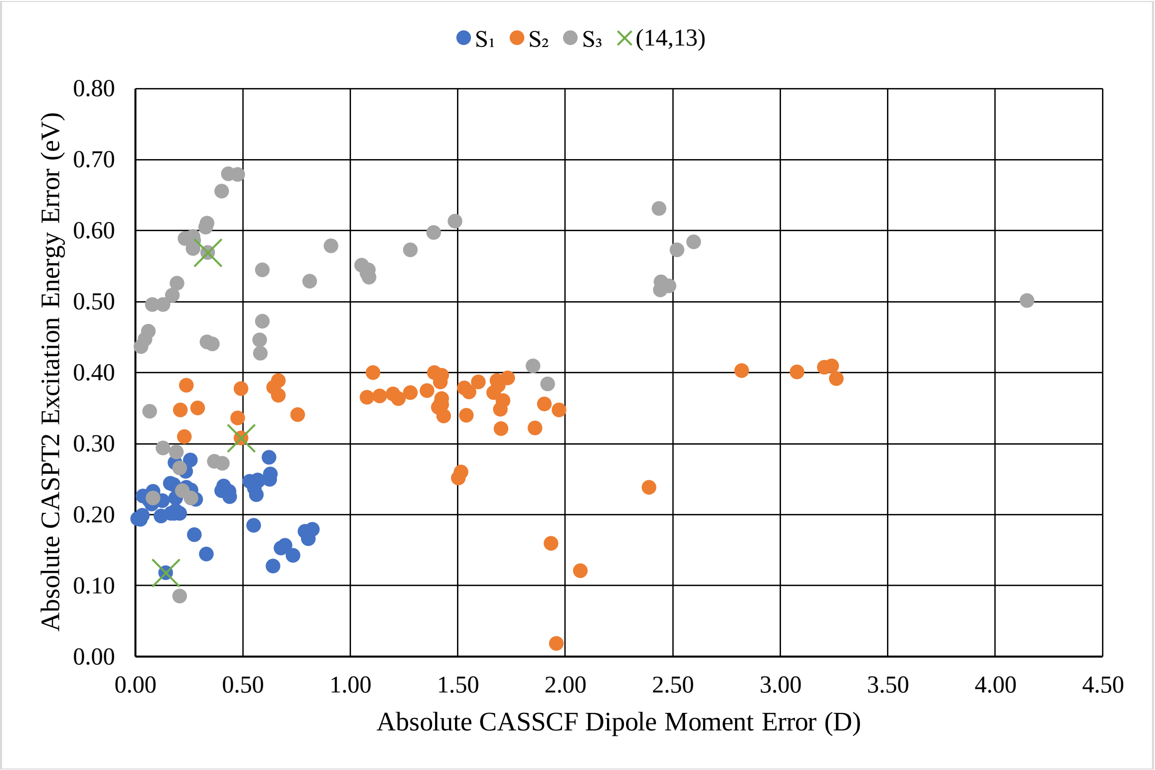

Figure S89: Plot of CASPT2 excitation energy error with respect to excited-state CASSCF dipole moment error for imidazole.

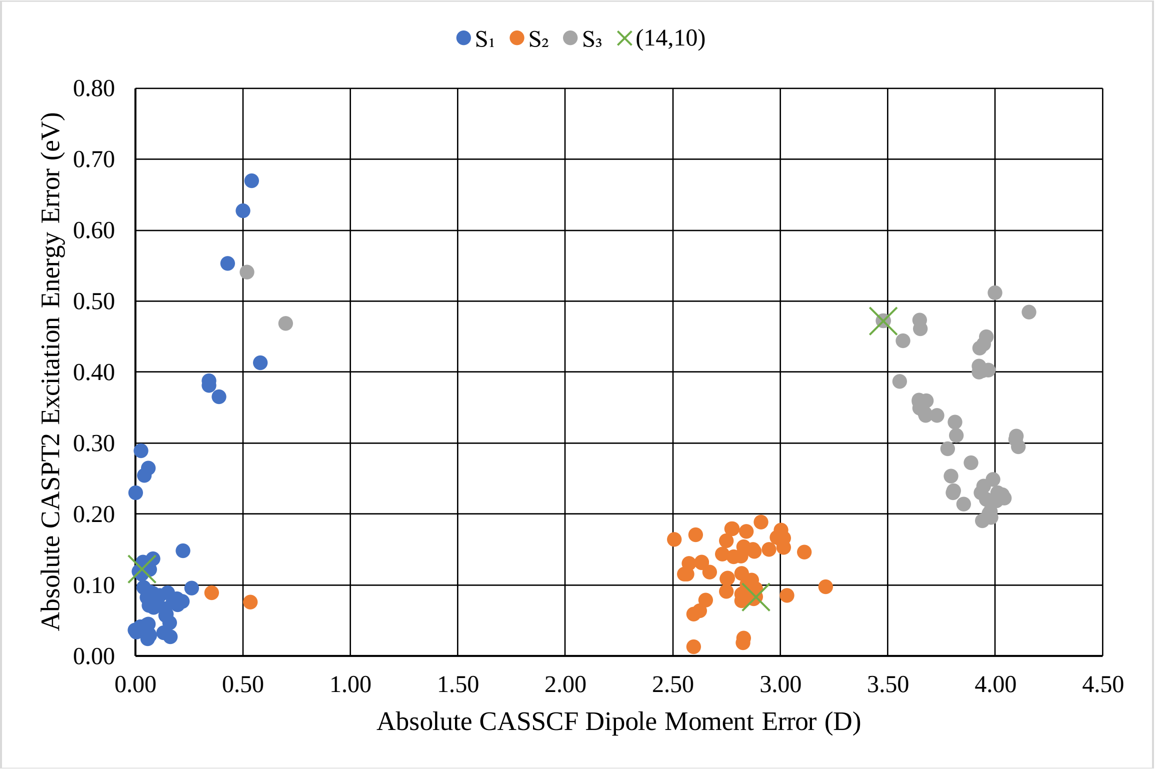

Figure S90: Plot of CASPT2 excitation energy error with respect to excited-state CASSCF dipole moment error for nitrosyl hydride.

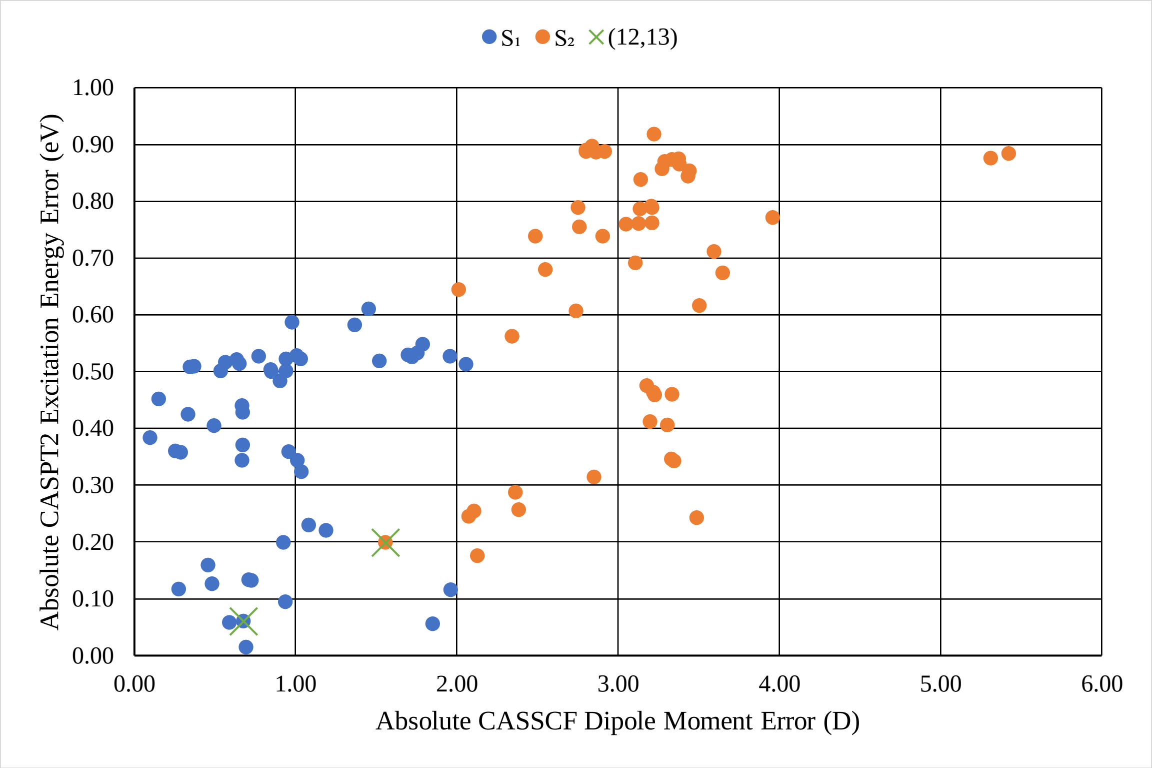

Figure S91: Plot of CASPT2 excitation energy error with respect to excited-state CASSCF dipole moment error for cyclopropene.

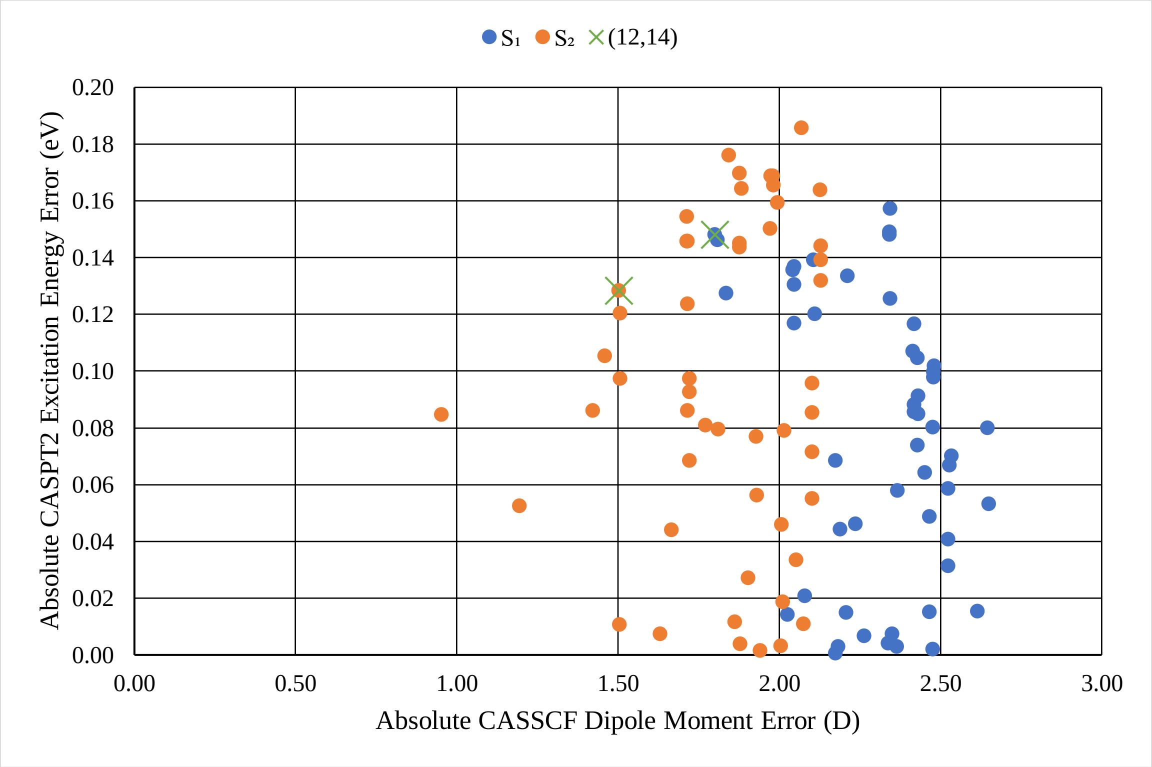

Figure S92: Plot of CASPT2 excitation energy error with respect to excited-state CASSCF dipole moment error for hydrogen sulfide.

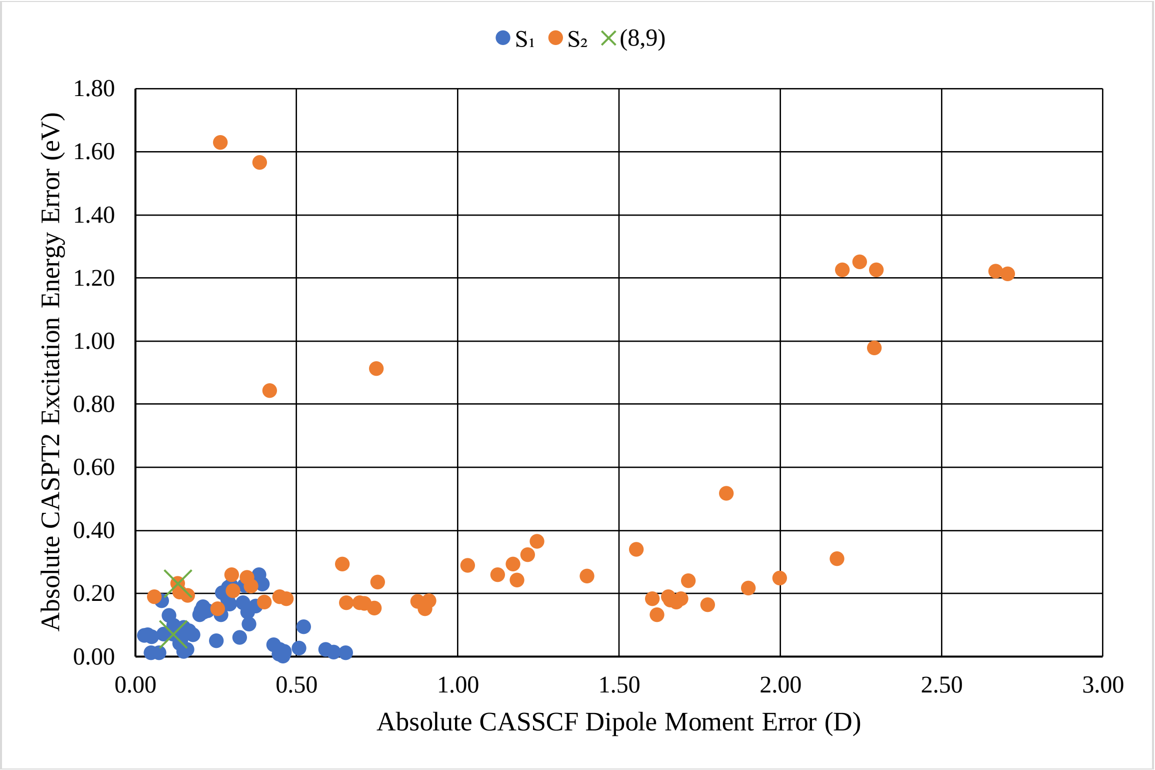

Figure S93: Plot of CASPT2 excitation energy error with respect to excited-state CASSCF dipole moment error for propynal.

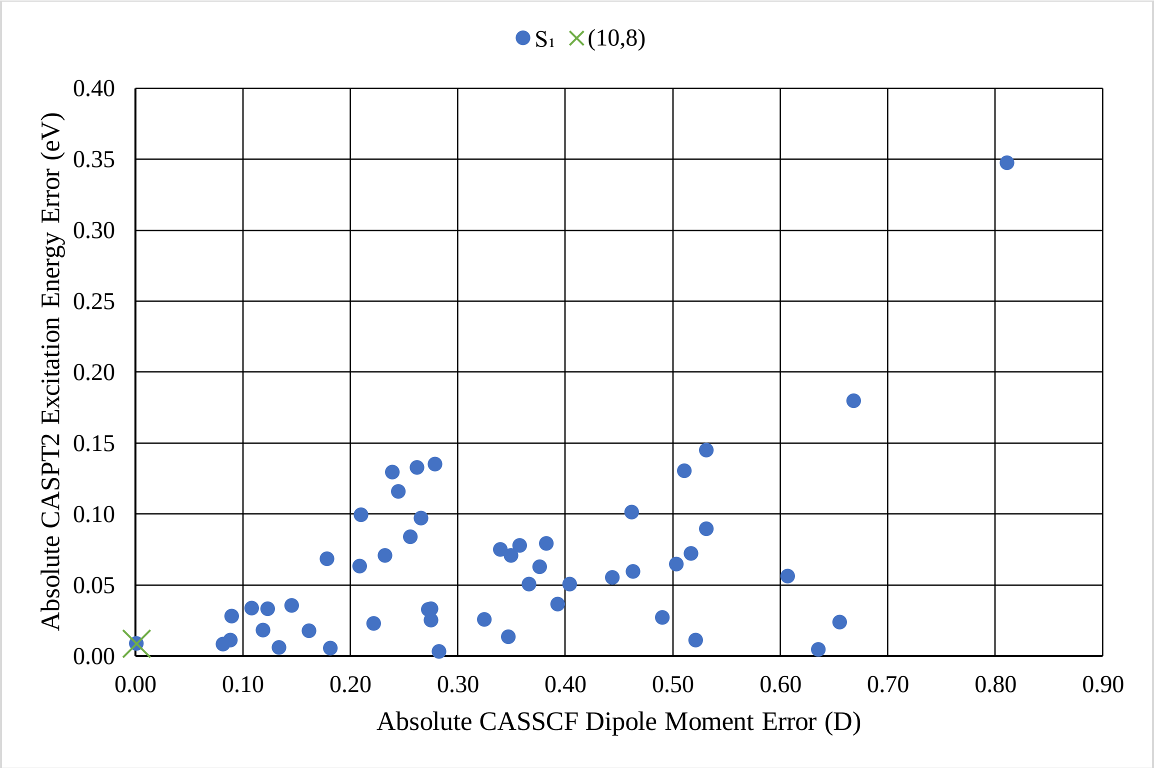

Figure S94: Plot of CASPT2 excitation energy error with respect to excited-state CASSCF dipole moment error for acetaldehyde.

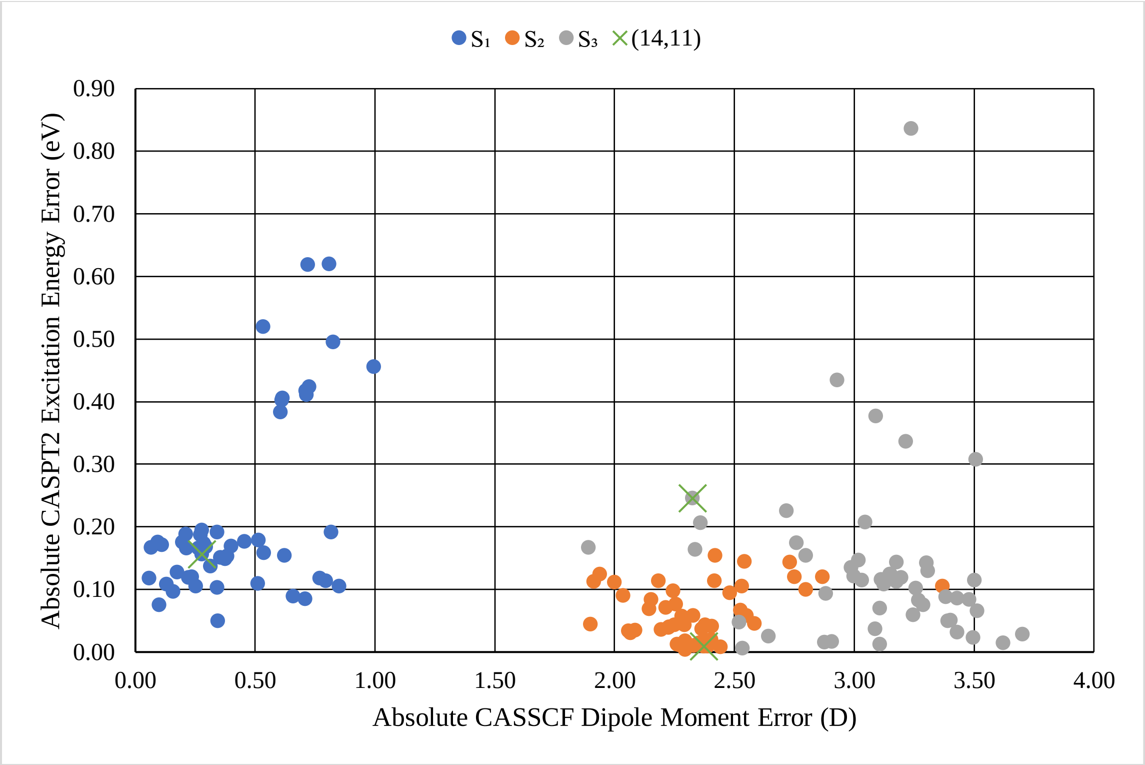

Figure S95: Plot of CASPT2 excitation energy error with respect to excited-state CASSCF dipole moment error for nitrosomethane.

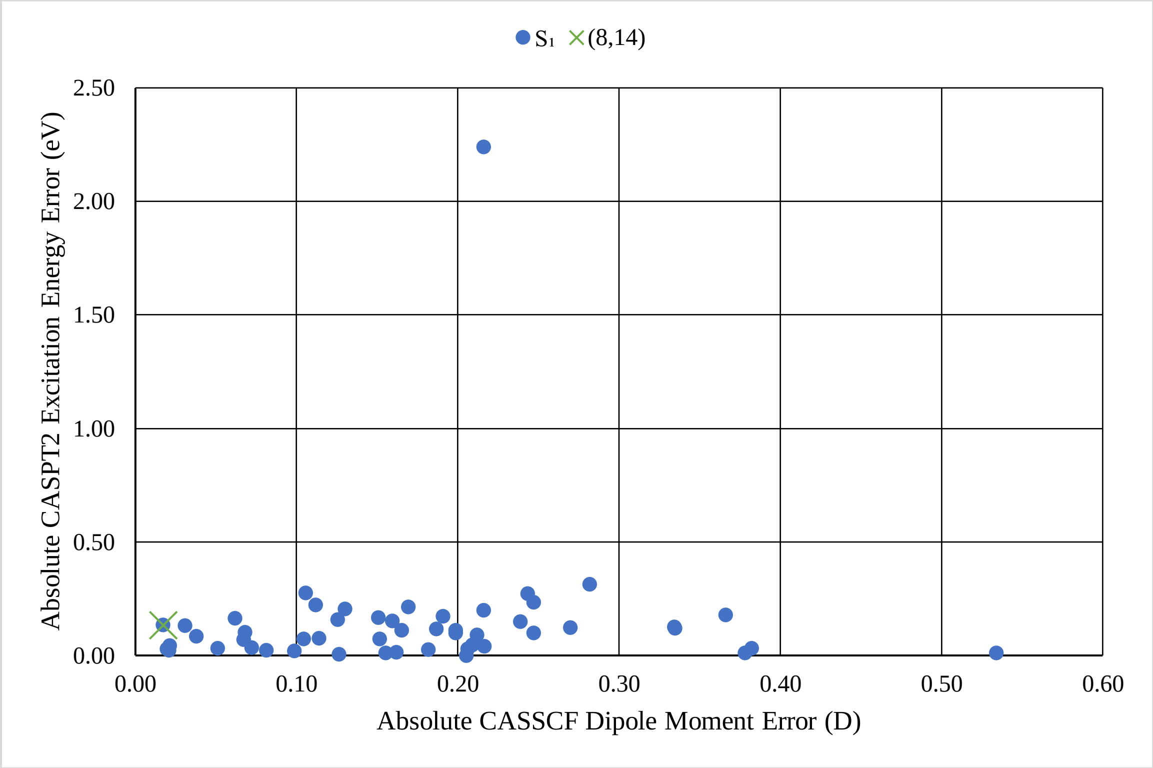

Figure S96: Plot of CASPT2 excitation energy error with respect to excited-state CASSCF dipole moment error for difluorocarbene.

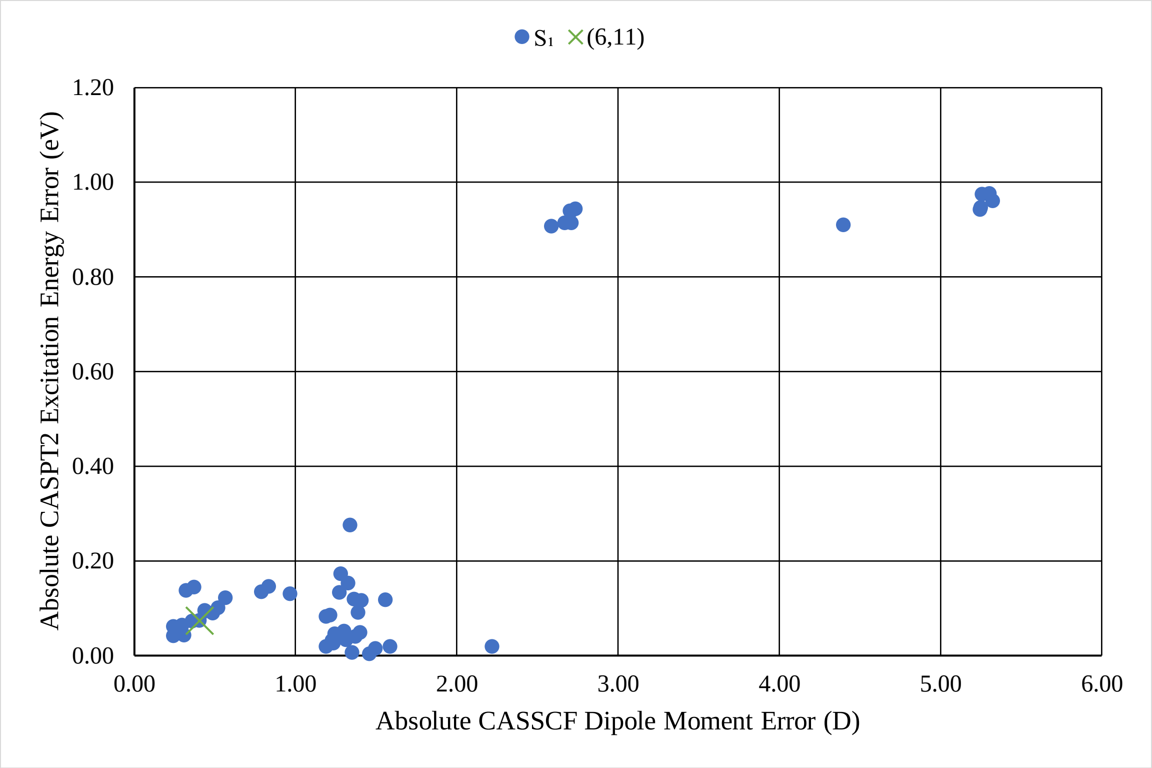

Figure S97: Plot of CASPT2 excitation energy error with respect to excited-state CASSCF dipole moment error for formamide.

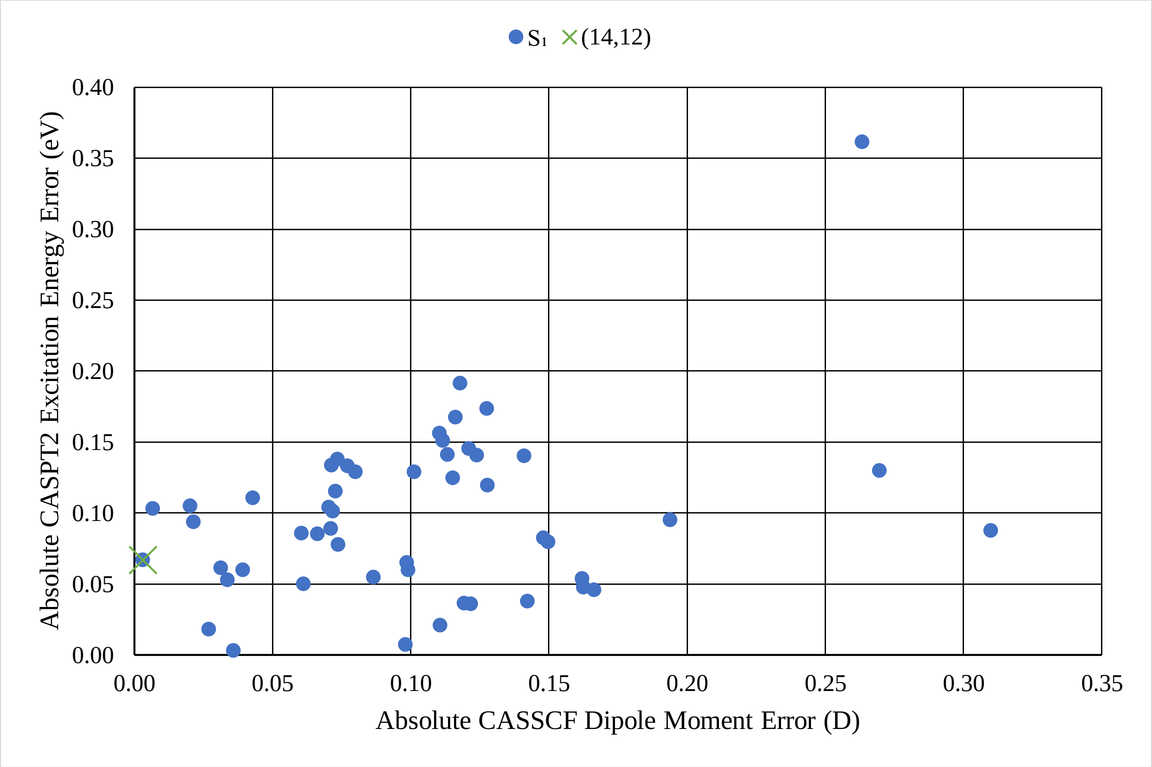

Figure S98: Plot of CASPT2 excitation energy error with respect to excited-state CASSCF dipole moment error for formyl fluoride.

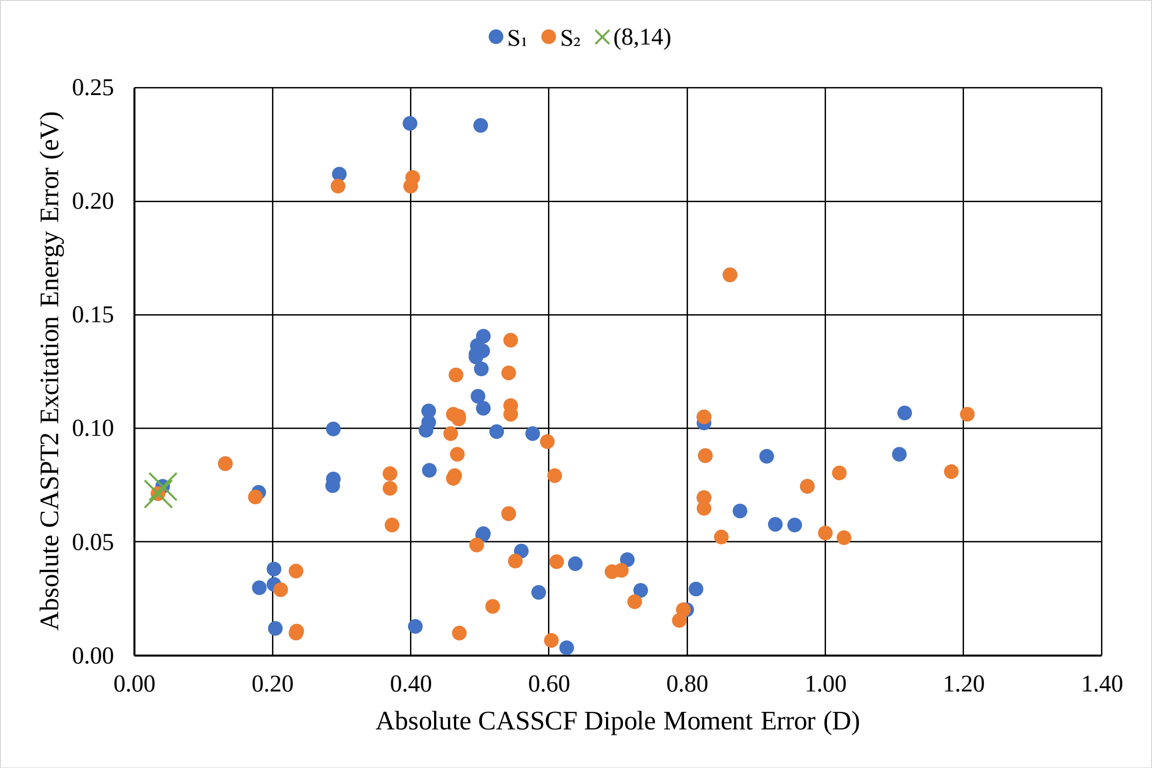

Figure S99: Plot of CASPT2 excitation energy error with respect to excited-state CASSCF dipole moment error for hydrogen chloride.

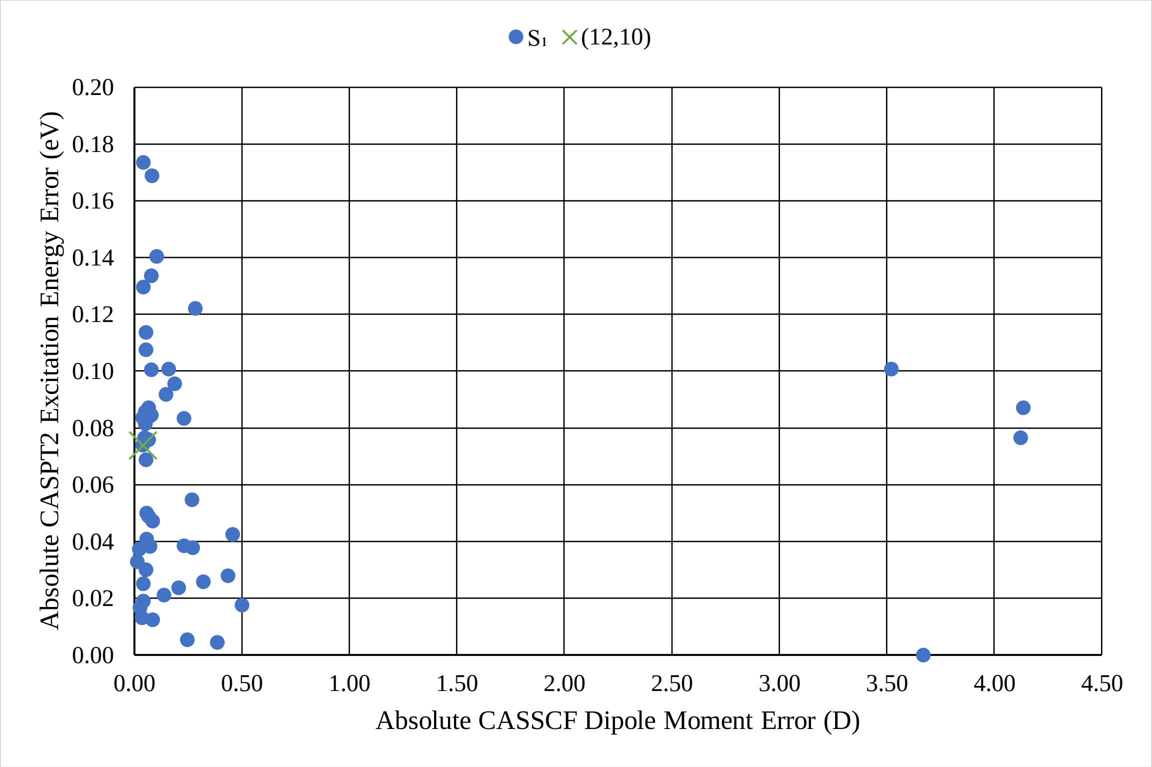

Figure S100: Plot of CASPT2 excitation energy error with respect to excited-state CASSCF dipole moment error for methanimine.

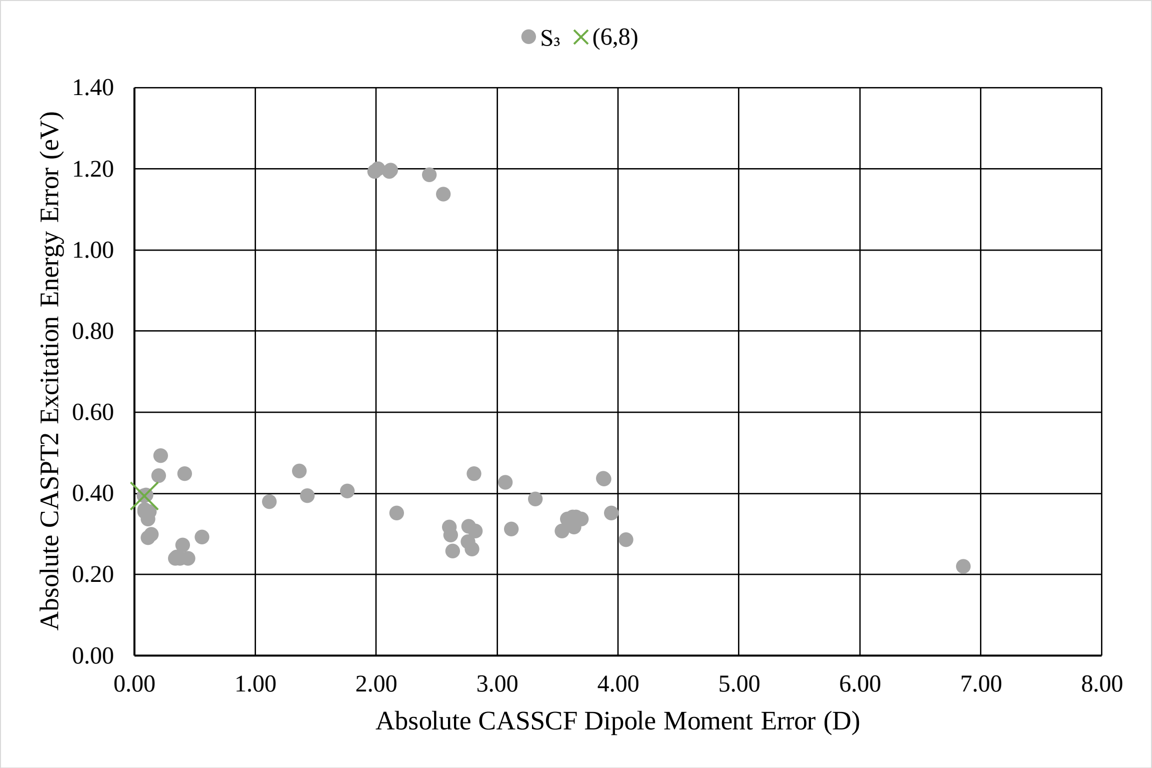

Figure S101: Plot of CASPT2 excitation energy error with respect to excited-state CASSCF dipole moment error for aniline.

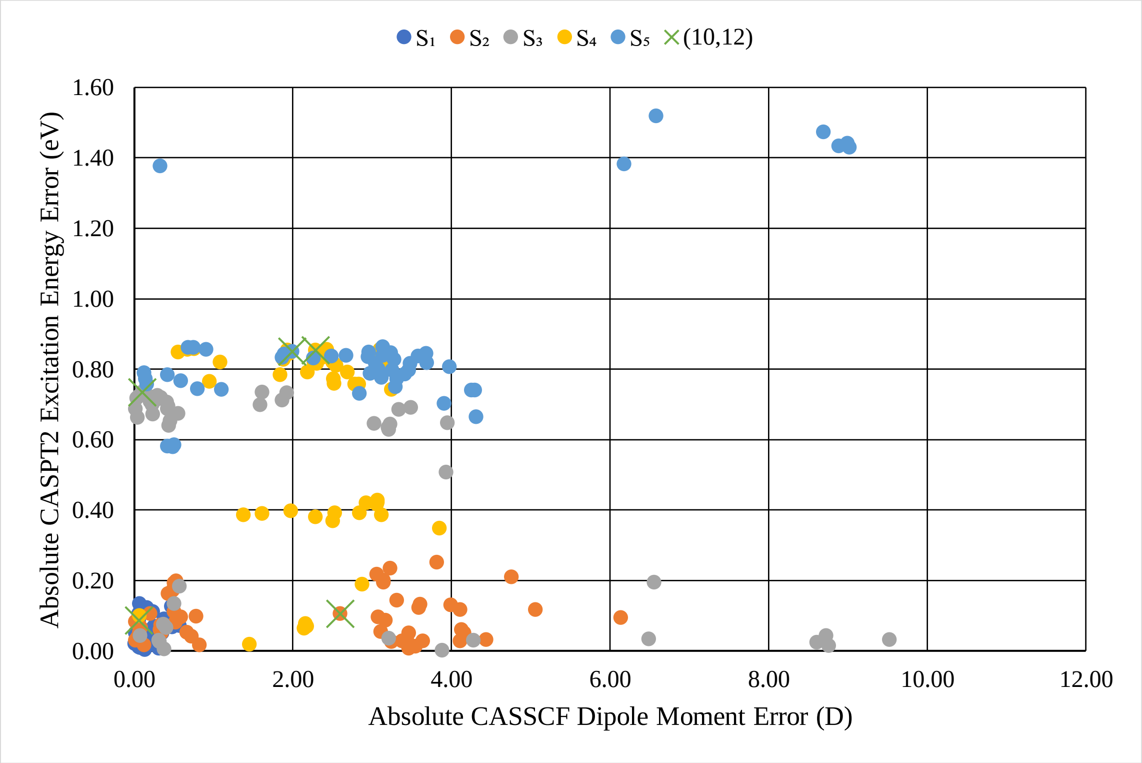

Figure S102: Plot of CASPT2 excitation energy error with respect to excited-state CASSCF dipole moment error for azulene.

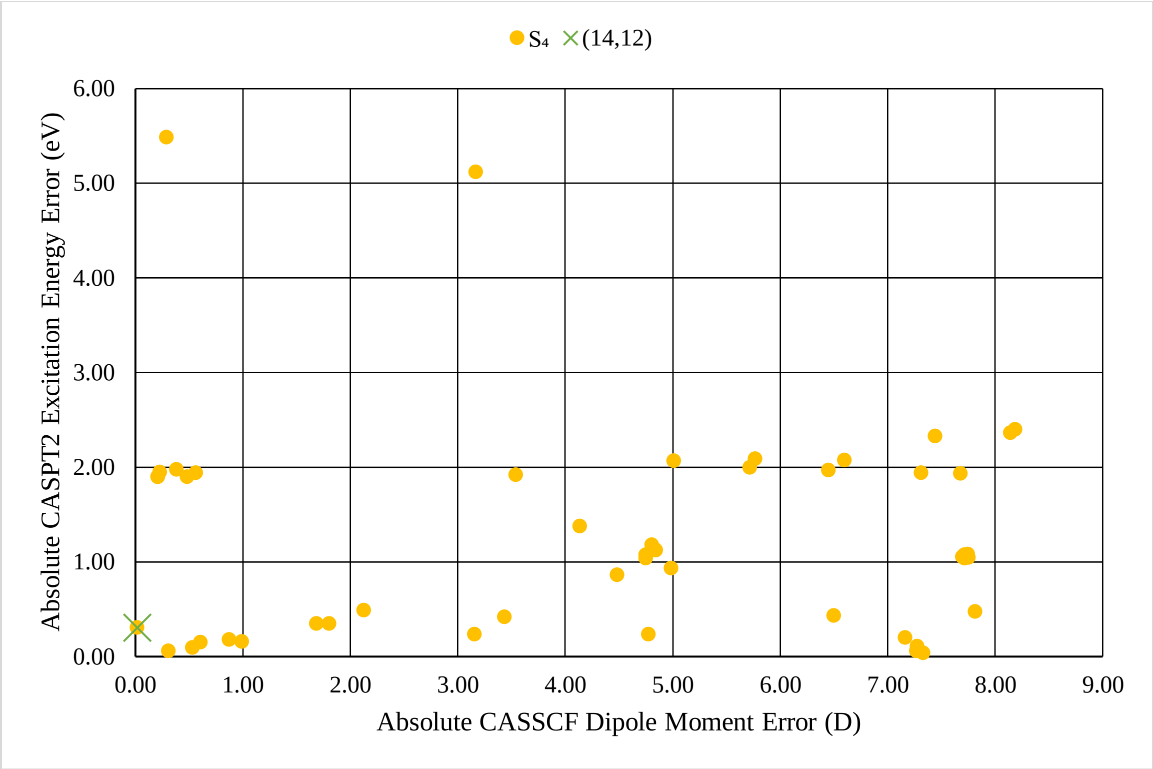

Figure S103: Plot of CASPT2 excitation energy error with respect to excited-state CASSCF dipole moment error for nitrobenzene.

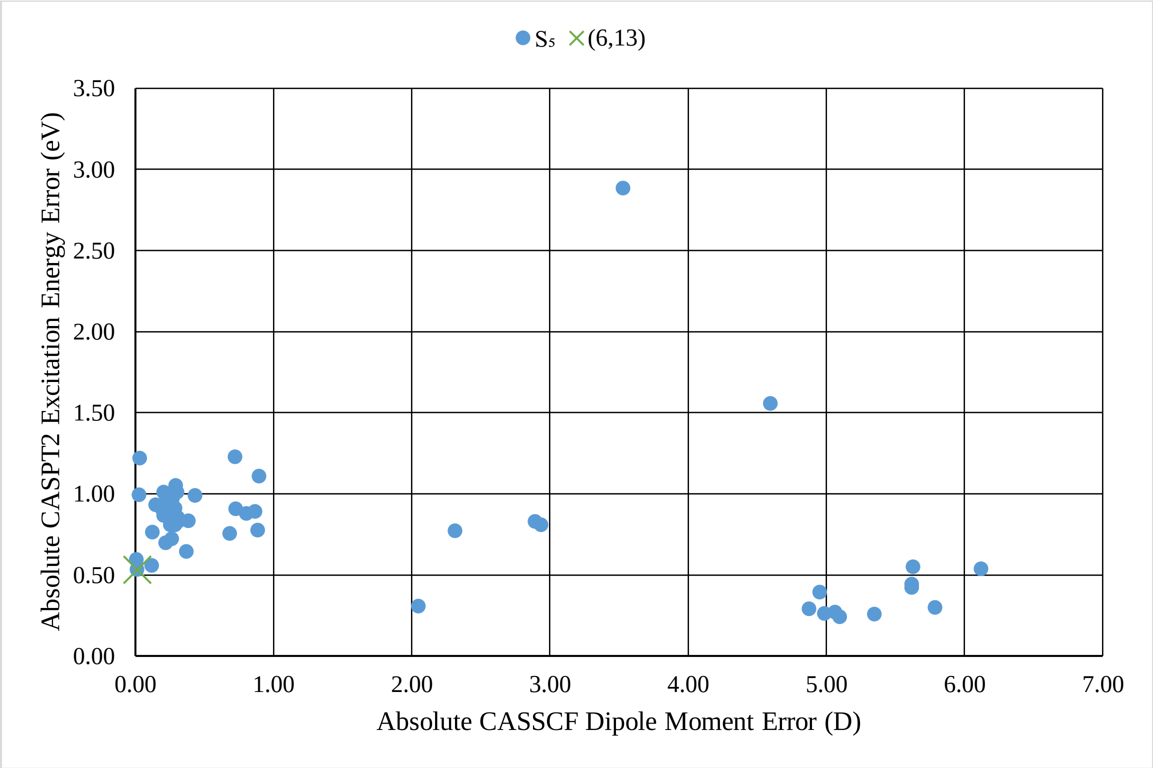

Figure S104: Plot of CASPT2 excitation energy error with respect to excited-state CASSCF dipole moment error for benzonitrile.

Performance of EDM-AS with S_4 and S_5 Excitations Ignored

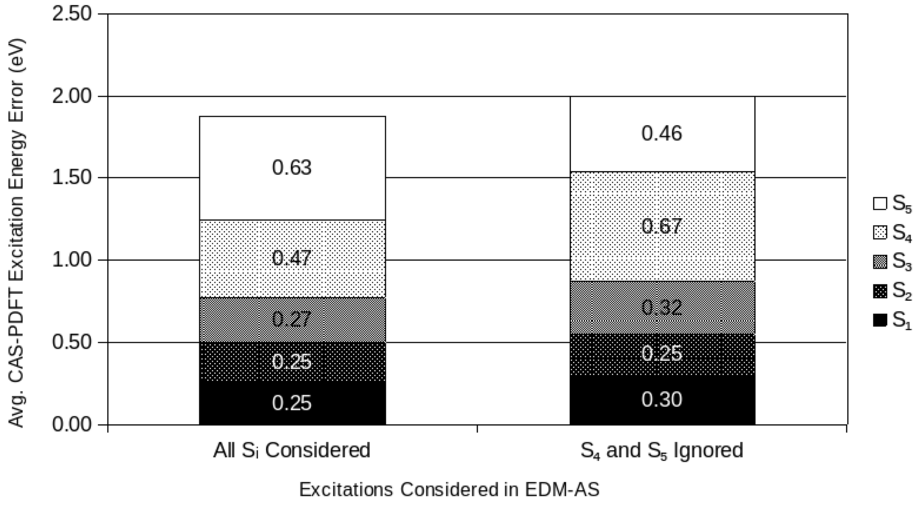

Figure S105: Average CAS-PDFT excitation energy errors for active spaces chosen by EDM-AS, as typically applied and with S_4 and S_5 dropped from consideration. Only molecules with four or more reference excitation energies are included here, as all other molecules will not be affected.

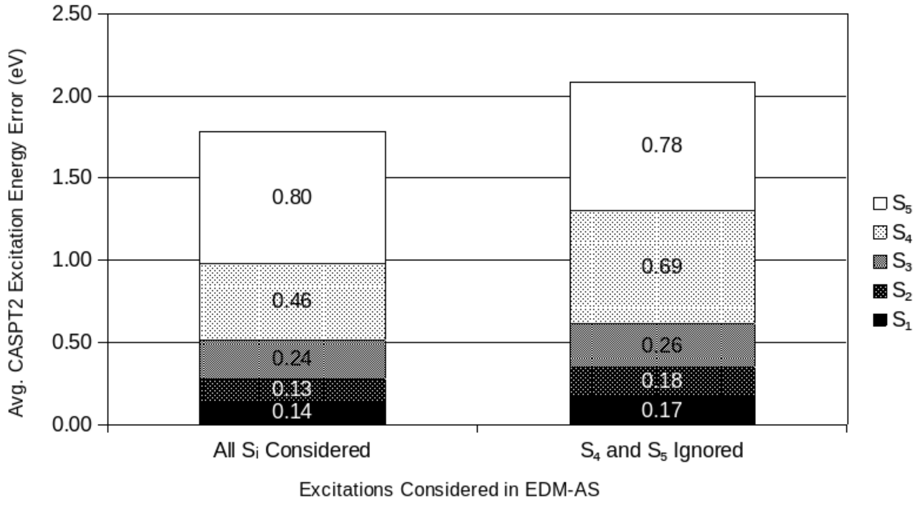

Figure S106: Average CASPT2 excitation energy errors for active spaces chosen by EDM-AS, as typically applied and with S_4 and S_5 dropped from consideration. Only molecules with four or more reference excitation energies are included here, as all other molecules will not be affected.

Performance of EDM-AS on Describing Double Excitations

Table S8: CAS-PDFT and CASPT2 excitation energy errors for the active spaces chosen by EDM-AS for the nitrosyl hydride and nitrosomethane molecules. S_2 is the double excitation in each case.

Molecule	Active Space	CAS-PDFT Excitation Energy Error (eV)			CASPT2 Excitation Energy Error (eV)		
		S_1	S_2	S_3	S_1	S_2	S_3
Nitrosyl Hydride	(14,10)	0.07	0.04	0.04	0.12	0.08	0.47
Nitroso-methane	(14,11)	0.08	0.07	0.07	0.16	0.01	0.25

vEDM-AS (EDM-AS with the Direction of Dipole Moments Considered)

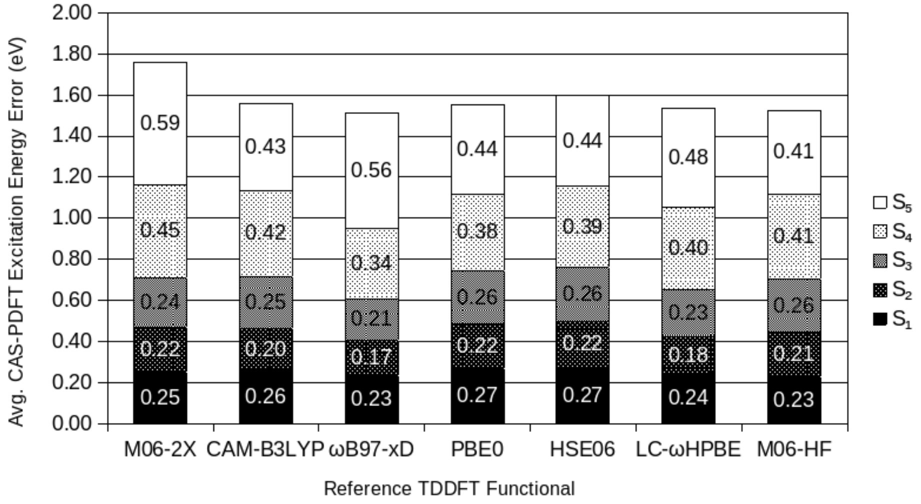

Figure S107: Average CAS-PDFT excitation energy error for active spaces chosen by vEDM-AS, accounting for the direction of the dipole moments and using reference dipole provided by a series of density functionals. The ANO-RCC-VTZP basis set is always used.

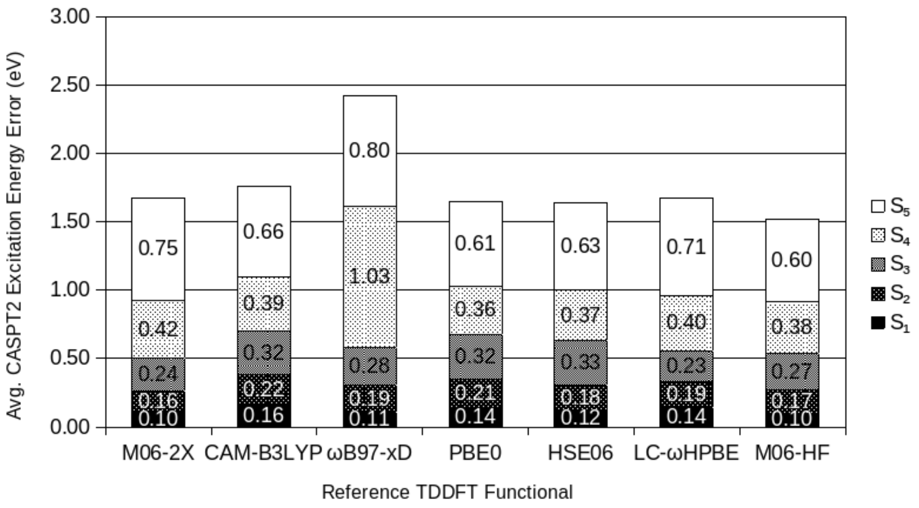

Figure S108: Average CASPT2 excitation energy error for active spaces chosen by vEDM-AS, accounting for the direction of the dipole moments and using reference dipole provided by a series of density functionals. The ANO-RCC-VTZP basis set is always used.

Table S9: Test systems with differences in active spaces recommended by EDM-AS accounting for only the total dipole moment and vEDM-AS accounting for the direction of the dipole moment, and the corresponding recommended active spaces and resulting excitation energy errors. Note the molecules with the same active spaces chosen for both protocols (17 for M06-HF, 15 for M06-2X, 13 for CAM-B3LYP, 12 for ωB97-xD, PBE0 and LC-ωHPBE, and 10 for HSE06) are omitted.

Original EDM-AS

M06-2X

Molecule	Active Space	CAS-PDFT Excitation Energy Error (eV)					CASPT2 Excitation Energy Error (eV)				
		S_1	S_2	S_3	S_4	S_5	S_1	S_2	S_3	S_4	S_5
Pyridine	(14,10)	0.21	0.23	0.51	0.49	0.55	0.15	0.11	0.11	0.37	0.38
Pyrrole	(8,9)	0.13	0.28	0.17	0.30	0.10	0.24	0.22	0.29	0.09	0.59
Thiophene	(14,10)	0.30	0.29	0.19	0.34	0.70	0.38	0.20	0.33	0.57	1.19
Imidazole	(14,13)	0.18	0.22	0.04			0.12	0.31	0.57		
Nitrosyl Hydride	(14,10)	0.07	0.04	0.04			0.12	0.08	0.47		
Nitrosomethane	(14,11)	0.08	0.07	0.07			0.16	0.01	0.25		
Cyclopropene	(12,13)	0.05	0.09				0.06	0.20			
Propynal	(8,9)	0.22	0.02				0.07	0.23			
Acetaldehyde	(10,8)	0.17					0.01				
Difluorocarbene	(8,14)	0.92					0.13				

CAM-B3LYP

Molecule	Active Space	CAS-PDFT Excitation Energy Error (eV)					CASPT2 Excitation Energy Error (eV)				
		S_1	S_2	S_3	S_4	S_5	S_1	S_2	S_3	S_4	S_5
Pyridine	(6,6)	0.27	0.29	0.77	0.52	0.45	0.25	0.19	0.22	0.38	0.36
Thiophene	(14,10)	0.30	0.29	0.19	0.34	0.70	0.38	0.20	0.33	0.57	1.19
Diazirine	(14,14)	0.08	0.05	0.21	0.29		0.00	0.01	0.03	0.21	
Imidazole	(14,13)	0.18	0.22	0.04			0.12	0.31	0.57		
Nitrosyl Hydride	(14,10)	0.07	0.04	0.04			0.12	0.08	0.47		
Nitrosomethane	(14,11)	0.08	0.07	0.07			0.16	0.01	0.25		
Cyclopropene	(12,13)	0.05	0.09				0.06	0.20			
Acetaldehyde	(12,10)	0.18					0.04				
Difluorocarbene	(10,14)	0.88					0.20				
Formyl Fluoride	(14,11)	0.00					0.05				
Aniline	(12,14)			0.22					0.31		
Nitrobenzene	(12,13)				0.19					5.48	

ωB97-xD

Molecule	Active Space	CAS-PDFT Excitation Energy Error (eV)					CASPT2 Excitation Energy Error (eV)				
		S_1	S_2	S_3	S_4	S_5	S_1	S_2	S_3	S_4	S_5
Pyridine	(6,6)	0.27	0.29	0.77	0.52	0.45	0.25	0.19	0.22	0.38	0.36
Pyrrole	(12,11)	0.08	0.22	0.13	0.26	0.10	0.23	0.28	0.29	0.08	0.53
Thiophene	(14,12)	0.51	0.24	0.37	0.38	0.64	0.72	0.67	0.55	0.67	0.74
Diazirine	(14,14)	0.08	0.05	0.21	0.29		0.00	0.01	0.03	0.21	
Nitrosyl Hydride	(14,10)	0.07	0.04	0.04			0.12	0.08	0.47		
Nitrosomethane	(14,11)	0.08	0.07	0.07			0.16	0.01	0.25		
Cyclopropene	(12,13)	0.05	0.09				0.06	0.20			
Acetaldehyde	(12,14)	0.11					0.03				
Difluorocarbene	(14,10)	0.89					0.12				
Formyl Fluoride	(12,9)	0.07					0.11				
Aniline	(14,11)			0.08					0.38		
Azulene	(10,12)	0.36	0.05	0.48	0.50	0.52	0.09	0.11	0.74	0.85	0.85
Nitrobenzene	(12,12)				0.16					0.09	

PBE0

Molecule	Active Space	CAS-PDFT Excitation Energy Error (eV)					CASPT2 Excitation Energy Error (eV)				
		S_1	S_2	S_3	S_4	S_5	S_1	S_2	S_3	S_4	S_5
Pyridine	(14,10)	0.21	0.23	0.51	0.49	0.55	0.15	0.11	0.11	0.37	0.38
Pyrrole	(12,11)	0.08	0.22	0.13	0.26	0.10	0.23	0.28	0.29	0.08	0.53
Thiophene	(14,10)	0.30	0.29	0.19	0.34	0.70	0.38	0.20	0.33	0.57	1.19
Diazirine	(14,11)	0.24	0.00	0.21	0.24		0.04	0.05	0.10	0.24	
Imidazole	(12,12)	0.16	0.18	0.05			0.15	0.35	0.57		
Nitrosyl Hydride	(14,10)	0.07	0.04	0.04			0.12	0.08	0.47		
Cyclopropene	(12,13)	0.05	0.09				0.06	0.20			
Acetaldehyde	(12,14)	0.11					0.03				
Difluorocarbene	(14,10)	0.89					0.12				
Formyl Fluoride	(14,10)	0.11					0.11				
Methanimine	(14,10)	0.01					0.05				
Aniline	(10,13)			0.11					0.44		
Nitrobenzene	(10,12)				0.09					0.16	

HSE06

Molecule	Active Space	CAS-PDFT Excitation Energy Error (eV)					CASPT2 Excitation Energy Error (eV)				
		S_1	S_2	S_3	S_4	S_5	S_1	S_2	S_3	S_4	S_5
Pyridine	(14,11)	0.19	0.28	0.45	0.47	0.51	1.78	0.08	0.25	1.36	0.18
Thiophene	(14,10)	0.30	0.29	0.19	0.34	0.70	0.38	0.20	0.33	0.57	1.19
Diazirine	(14,14)	0.08	0.05	0.21	0.29		0.00	0.01	0.03	0.21	
Imidazole	(12,12)	0.16	0.18	0.05			0.15	0.35	0.57		
Nitrosyl Hydride	(14,10)	0.07	0.04	0.04			0.12	0.08	0.47		
Nitrosomethane	(6,12)	0.03	0.07	0.08			0.10	0.11	0.09		
Cyclopropene	(12,13)	0.05	0.09				0.06	0.20			
Acetaldehyde	(12,14)	0.11					0.03				
Difluorocarbene	(14,10)	0.89					0.12				
Formamide	(8,13)	0.26					0.15				
Formyl Fluoride	(14,10)	0.11					0.11				
Methanimine	(14,10)	0.01					0.05				
Aniline	(10,14)			0.11					0.43		
Azulene	(8,11)	0.23	0.01	0.52	0.54	0.56	0.03	0.12	0.73	0.85	0.86
Nitrobenzene	(10,12)				0.09					0.16	

LC-ωHPBE

Molecule	Active Space	CAS-PDFT Excitation Energy Error (eV)					CASPT2 Excitation Energy Error (eV)				
		S_1	S_2	S_3	S_4	S_5	S_1	S_2	S_3	S_4	S_5
Pyridine	(6,6)	0.27	0.29	0.77	0.52	0.45	0.25	0.19	0.22	0.38	0.36
Pyrrole	(12,11)	0.08	0.22	0.13	0.26	0.10	0.23	0.28	0.29	0.08	0.53
Diazirine	(14,13)	0.08	0.06	0.28	0.28		0.01	0.05	0.10	0.46	
Diazomethane	(12,9)	0.34	0.32	0.69			0.17	0.25	0.78		
Imidazole	(6,9)	0.20	0.22	0.11			0.18	0.38	0.63		
Nitrosyl Hydride	(14,10)	0.07	0.04	0.04			0.12	0.08	0.47		
Nitrosomethane	(14,11)	0.08	0.07	0.07			0.16	0.01	0.25		
Cyclopropene	(12,13)	0.05	0.09				0.06	0.20			
Propynal	(8,9)	0.22	0.02				0.07	0.23			
Acetaldehyde	(6,14)	0.01					0.01				
Difluorocarbene	(14,10)	0.89					0.12				
Formyl Fluoride	(12,12)	0.05					0.09				
Aniline	(14,11)			0.08					0.38		

M06-HF

Molecule	Active Space	CAS-PDFT Excitation Energy Error (eV)					CASPT2 Excitation Energy Error (eV)				
		S_1	S_2	S_3	S_4	S_5	S_1	S_2	S_3	S_4	S_5
Carbon Monoxide	(6,12)	0.15	0.15	0.10	0.27	0.27	0.50	0.50	0.19	0.20	0.20
Formaldehyde	(10,8)	0.04	0.11	0.14	0.73	1.43	0.03	0.15	0.00	1.03	1.52
Pyrrole	(12,11)	0.08	0.22	0.13	0.26	0.10	0.23	0.28	0.29	0.08	0.53
Ketene	(14,10)	0.44	0.34	0.34	2.26		0.10	0.06	0.14	1.97	
Cyclopropene	(12,13)	0.05	0.09				0.06	0.20			
Formyl Fluoride	(10,8)	0.04					0.08				
Methanimine	(12,12)	0.18					0.08				
Aniline	(8,11)			0.15					0.39		

EDM-AS with Dipole Moment Direction

M06-2X

Molecule	Active Space	CAS-PDFT Excitation Energy Error (eV)					CASPT2 Excitation Energy Error (eV)				
		S_1	S_2	S_3	S_4	S_5	S_1	S_2	S_3	S_4	S_5
Pyridine	(8,9)	0.43	0.37	0.69	0.52	0.47	0.41	0.23	0.00	0.44	0.96
Pyrrole	(12,12)	0.24	0.31	0.26	0.31	0.08	0.13	0.18	0.22	0.00	0.60
Thiophene	(12,10)	0.36	0.35	0.31	0.33	0.70	0.25	0.44	0.33	0.39	0.51
Imidazole	(8,12)	0.29	0.31	0.07			0.18	0.34	0.58		
Nitrosyl Hydride	(10,10)	0.06	0.06	0.09			0.10	0.10	0.33		
Nitrosomethane	(14,12)	0.05	0.08	0.07			0.10	0.12	0.17		
Cyclopropene	(12,14)	0.12	0.16				0.01	0.31			
Propynal	(10,10)	0.20	0.07				0.08	0.20			
Acetaldehyde	(12,14)	0.11					0.03				
Difluorocarbene	(6,12)	1.09					0.04				

CAM-B3LYP

Molecule	Active Space	CAS-PDFT Excitation Energy Error (eV)					CASPT2 Excitation Energy Error (eV)				
		S_1	S_2	S_3	S_4	S_5	S_1	S_2	S_3	S_4	S_5
Pyridine	(8,9)	0.43	0.37	0.69	0.52	0.47	0.41	0.23	0.00	0.44	0.96
Thiophene	(12,10)	0.36	0.35	0.31	0.33	0.70	0.25	0.44	0.33	0.39	0.51
Diazirine	(14,10)	0.48	0.08	0.21	0.06		0.02	0.25	0.19	0.17	
Imidazole	(12,10)	0.08	0.19	0.12			0.24	0.37	0.55		
Nitrosyl Hydride	(10,10)	0.06	0.06	0.09			0.10	0.10	0.33		
Nitrosomethane	(14,12)	0.05	0.08	0.07			0.10	0.12	0.17		
Cyclopropene	(6,12)	0.06	0.21				0.58	0.88			
Acetaldehyde	(12,11)	0.19					0.07				
Difluorocarbene	(10,10)	1.17					0.27				
Formyl Fluoride	(6,14)	0.16					0.19				
Aniline	(12,13)			0.22					0.31		
Nitrobenzene	(14,12)				0.23					0.31	

ωB97-xD

Molecule	Active Space	CAS-PDFT Excitation Energy Error (eV)					CASPT2 Excitation Energy Error (eV)				
		S_1	S_2	S_3	S_4	S_5	S_1	S_2	S_3	S_4	S_5
Pyridine	(8,9)	0.43	0.37	0.69	0.52	0.47	0.41	0.23	0.00	0.44	0.96
Pyrrole	(14,12)	0.07	0.20	0.12	0.23	0.01	0.23	0.19	0.30	0.08	0.60
Thiophene	(6,14)	0.16	0.07	0.18	0.22	0.80	0.04	0.30	0.29	0.31	0.69
Diazirine	(14,11)	0.24	0.00	0.21	0.24		0.04	0.05	0.10	0.24	
Nitrosyl Hydride	(10,10)	0.06	0.06	0.09			0.10	0.10	0.33		
Nitrosomethane	(14,12)	0.05	0.08	0.07			0.10	0.12	0.17		
Cyclopropene	(12,14)	0.12	0.16				0.01	0.31			
Acetaldehyde	(10,9)	0.16					0.03				
Difluorocarbene	(14,14)	0.97					0.11				
Formyl Fluoride	(14,12)	0.11					0.07				
Aniline	(8,11)			0.15					0.39		
Azulene	(6,7)	0.39	0.29	0.14	0.30	0.60	0.12	0.10	0.13	0.39	0.77
Nitrobenzene	(12,13)				0.19					5.48	

PBE0

Molecule	Active Space	CAS-PDFT Excitation Energy Error (eV)					CASPT2 Excitation Energy Error (eV)				
		S_1	S_2	S_3	S_4	S_5	S_1	S_2	S_3	S_4	S_5
Pyridine	(8,9)	0.43	0.37	0.69	0.52	0.47	0.41	0.23	0.00	0.44	0.96
Pyrrole	(12,12)	0.24	0.31	0.26	0.31	0.08	0.13	0.18	0.22	0.00	0.60
Thiophene	(12,10)	0.36	0.35	0.31	0.33	0.70	0.25	0.44	0.33	0.39	0.51
Diazirine	(14,10)	0.48	0.08	0.21	0.06		0.02	0.25	0.19	0.17	
Imidazole	(12,14)	0.17	0.18	0.05			0.14	0.34	0.57		
Nitrosyl Hydride	(14,14)	0.04	0.05	0.11			0.11	0.11	0.21		
Cyclopropene	(6,12)	0.06	0.21				0.58	0.88			
Acetaldehyde	(14,11)	0.18					0.03				
Difluorocarbene	(14,14)	0.97					0.11				
Formyl Fluoride	(12,9)	0.07					0.11				
Methanimine	(12,10)	0.03					0.07				
Aniline	(10,14)			0.11					0.43		
Nitrobenzene	(12,14)				0.02					0.06	

HSE06

Molecule	Active Space	CAS-PDFT Excitation Energy Error (eV)					CASPT2 Excitation Energy Error (eV)				
		S_1	S_2	S_3	S_4	S_5	S_1	S_2	S_3	S_4	S_5
Pyridine	(8,9)	0.43	0.37	0.69	0.52	0.47	0.41	0.23	0.00	0.44	0.96
Thiophene	(12,10)	0.36	0.35	0.31	0.33	0.70	0.25	0.44	0.33	0.39	0.51
Diazirine	(14,10)	0.48	0.08	0.21	0.06		0.02	0.25	0.19	0.17	
Imidazole	(8,12)	0.29	0.31	0.07			0.18	0.34	0.58		
Nitrosyl Hydride	(10,10)	0.06	0.06	0.09			0.10	0.10	0.33		
Nitrosomethane	(14,14)	0.04	0.05	0.11			0.11	0.11	0.21		
Cyclopropene	(12,14)	0.12	0.16				0.01	0.31			
Acetaldehyde	(14,11)	0.18					0.03				
Difluorocarbene	(14,14)	0.97					0.11				
Formamide	(6,11)	0.21					0.07				
Formyl Fluoride	(6,8)	0.05					0.14				
Methanimine	(12,10)	0.03					0.07				
Aniline	(10,10)			0.25					0.22		
Azulene	(12,14)	0.34	0.07	0.43	0.45	0.49	0.06	0.03	0.70	0.82	0.82
Nitrobenzene	(12,14)				0.02					0.06	

LC-ωHPBE

Molecule	Active Space	CAS-PDFT Excitation Energy Error (eV)					CASPT2 Excitation Energy Error (eV)				
		S_1	S_2	S_3	S_4	S_5	S_1	S_2	S_3	S_4	S_5
Pyridine	(8,9)	0.43	0.37	0.69	0.52	0.47	0.41	0.23	0.00	0.44	0.96
Pyrrole	(14,12)	0.07	0.20	0.12	0.23	0.01	0.23	0.19	0.30	0.08	0.60
Diazirine	(14,14)	0.08	0.05	0.21	0.29		0.00	0.01	0.03	0.21	
Diazomethane	(14,14)	0.21	0.26	0.55			0.01	0.44	0.49		
Imidazole	(8,11)	0.04	0.10	0.13			0.20	0.12	0.08		
Nitrosyl Hydride	(10,10)	0.06	0.06	0.09			0.10	0.10	0.33		
Nitrosomethane	(14,12)	0.05	0.08	0.07			0.10	0.12	0.17		
Cyclopropene	(6,12)	0.06	0.21				0.58	0.88			
Propynal	(10,10)	0.20	0.07				0.08	0.20			
Acetaldehyde	(14,14)	0.56					0.10				
Difluorocarbene	(10,10)	1.17					0.27				
Formyl Fluoride	(10,14)	0.01					0.12				
Aniline	(8,11)			0.15					0.39		

M06-HF

Molecule	Active Space	CAS-PDFT Excitation Energy Error (eV)					CASPT2 Excitation Energy Error (eV)				
		S_1	S_2	S_3	S_4	S_5	S_1	S_2	S_3	S_4	S_5
Carbon Monoxide	(12,13)	0.64	0.64	0.21	0.40	0.40	0.21	0.21	0.31	0.27	0.27
Formaldehyde	(10,9)	0.02	0.35	0.39	0.61	0.26	0.00	0.23	0.09	0.99	0.55
Pyrrole	(14,12)	0.07	0.20	0.12	0.23	0.01	0.23	0.19	0.30	0.08	0.60
Ketene	(10,10)	0.28	0.34	0.23	0.69		0.10	0.10	0.10	0.30	
Cyclopropene	(12,14)	0.12	0.16				0.01	0.31			
Formyl Fluoride	(12,13)	0.05					0.10				
Methanimine	(6,12)	0.07					0.04				
Aniline	(12,11)			0.20					0.30		

Molecular Orbital Examples

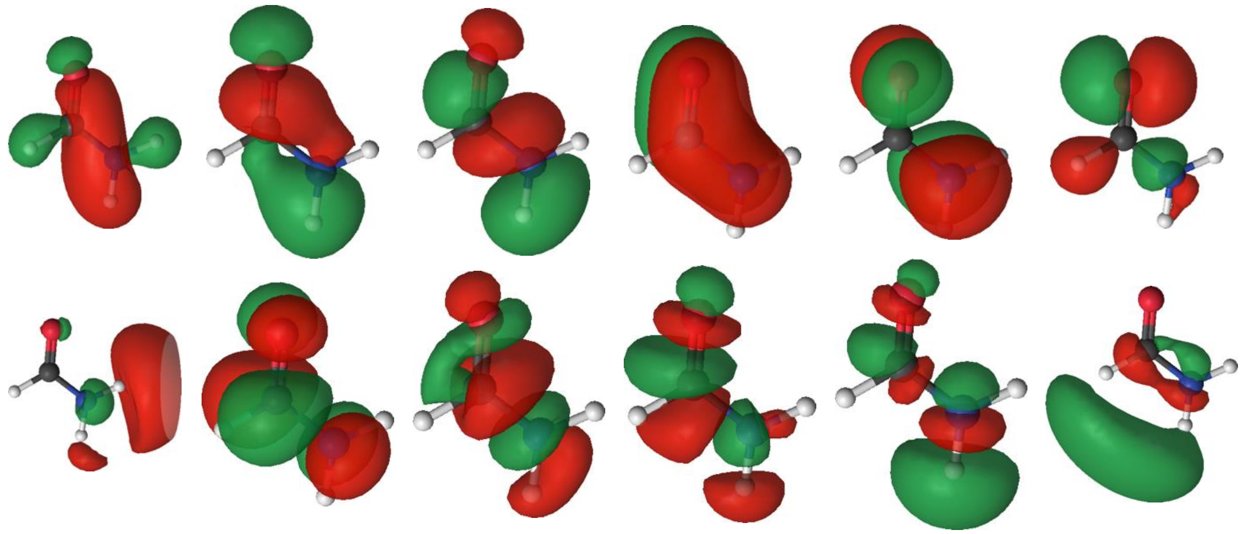

Figure S109: SA6-CASSCF orbitals in the (12,12) active space for the formamide molecule.

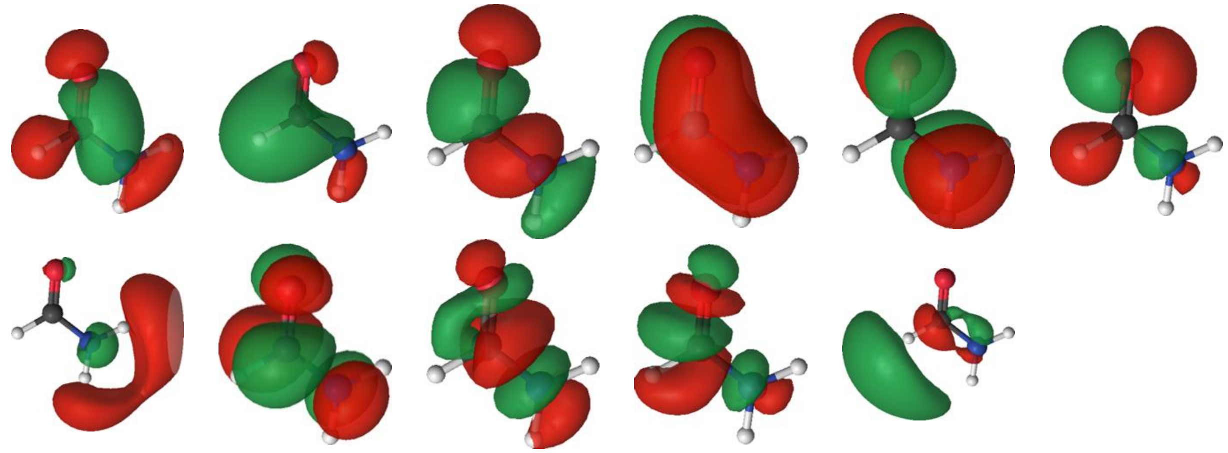

Figure S110: SA6-CASSCF orbitals in the (12,11) active space for the formamide molecule. There is one fewer valence orbital than in (12,12).

Active Spaces Chosen by Protocols Compared to (14,14)

Table S10: Excitation energy errors, solved with CAS-PDFT and CASPT2, for active spaces chosen by GDM-AS with experimental reference dipole moments and EDM-AS with TD-M06-2X reference dipole moments compared to (14,14).

GDM-AS with Experimental Dipole Moments

Molecule	Active Space	CAS-PDFT Excitation Energy Error (eV)					CASPT2 Excitation Energy Error (eV)				
		S_1	S_2	S_3	S_4	S_5	S_1	S_2	S_3	S_4	S_5
Carbon Monoxide	(12,13)	0.64	0.64	0.21	0.40	0.40	0.21	0.21	0.31	0.27	0.27
Formaldehyde	(6,7)	0.15	0.20	0.26	0.63	0.20	0.07	0.02	0.11	1.00	0.67
Pyridine	(14,14)	0.00	0.22	0.01	0.40	0.28	0.04	0.07	0.21	0.25	0.27
Pyrrole	(6,10)	0.33	0.45	0.36	0.35	0.20	0.18	0.27	0.33	0.15	0.95
Thiophene	(12,11)	0.32	0.06	0.12	0.18	0.44	0.20	0.40	0.25	0.42	0.52
Diazirine	(14,14)	0.08	0.05	0.21	0.29		0.00	0.01	0.03	0.21	
Ketene	(10,8)	0.59	0.39	0.18	2.26		0.10	0.27	0.22	1.95	
Diazomethane	(8,12)	0.35	0.48	0.39			0.08	0.17	0.06		
Hydrogen Cyaphide	(12,13)	0.09	0.12	0.12			0.13	0.03	0.04		
Imidazole	(10,14)	0.04	0.12	0.14			0.19	0.02	0.22		
Nitrosyl Hydride	(8,7)	0.05	0.03	0.00			0.09	0.17	0.21		
Nitrosomethane	(12,11)	0.04	0.05	0.01			0.13	0.10	0.05		
Cyclopropene	(12,14)	0.12	0.16				0.01	0.31			
Hydrogen Sulfide	(6,11)	0.28	0.21				0.00	0.09			
Propynal	(10,14)	0.29	0.02				0.22	0.17			
Acetaldehyde	(14,10)	0.07					0.01				
Difluorocarbene	(10,11)	1.26					0.16				
Formamide	(12,11)	0.43					0.12				
Formyl Fluoride	(14,13)	0.00					0.05				
Hydrogen Chloride	(8,14)	0.25	0.23				0.07	0.07			
Methanimine	(8,9)	0.04					0.08				
Aniline	(10,10)			0.25					0.22		
Azulene	(10,13)	0.34	0.08	0.47	0.49	0.52	0.07	0.09	0.73	0.86	0.84
Nitrobenzene	(8,13)				0.45					0.93	
Benzonitrile	(6,9)					0.60					1.23
Average		0.26	0.21	0.20	0.61	0.38	0.10	0.14	0.21	0.67	0.68

EDM-AS with TD-M06-2X Reference Dipole Moments

Molecule	Active Space	CAS-PDFT Excitation Energy Error (eV)					CASPT2 Excitation Energy Error (eV)				
		S_1	S_2	S_3	S_4	S_5	S_1	S_2	S_3	S_4	S_5
Carbon Monoxide	(8,9)	0.63	0.63	0.25	0.46	0.46	0.16	0.16	0.30	0.29	0.29
Formaldehyde	(10,8)	0.04	0.11	0.14	0.73	1.43	0.03	0.15	0.00	1.03	1.52
Pyridine	(14,10)	0.21	0.23	0.51	0.49	0.55	0.15	0.11	0.11	0.37	0.38
Pyrrole	(8,9)	0.13	0.28	0.17	0.30	0.10	0.24	0.22	0.29	0.09	0.59
Thiophene	(14,10)	0.30	0.29	0.19	0.34	0.70	0.38	0.20	0.33	0.57	1.19
Diazirine	(14,14)	0.08	0.05	0.21	0.29		0.00	0.01	0.03	0.21	
Ketene	(10,10)	0.28	0.34	0.23	0.69		0.10	0.10	0.10	0.30	
Diazomethane	(14,12)	0.21	0.36	0.47			0.01	0.00	0.04		
Hydrogen Cyaphide	(8,14)	0.04	0.05	0.02			0.14	0.07	0.07		
Imidazole	(14,13)	0.18	0.22	0.04			0.12	0.31	0.57		
Nitrosyl Hydride	(14,10)	0.07	0.04	0.04			0.12	0.08	0.47		
Nitrosomethane	(14,11)	0.08	0.07	0.07			0.16	0.01	0.25		
Cyclopropene	(12,13)	0.05	0.09				0.06	0.20			
Hydrogen Sulfide	(12,14)	0.34	0.15				0.15	0.13			
Propynal	(8,9)	0.22	0.02				0.07	0.23			
Acetaldehyde	(10,8)	0.17					0.01				
Difluorocarbene	(8,14)	0.92					0.13				
Formamide	(6,11)	0.21					0.07				
Formyl Fluoride	(14,12)	0.11					0.07				
Hydrogen Chloride	(8,14)	0.25	0.23				0.07	0.07			
Methanimine	(12,10)	0.03					0.07				
Aniline	(6,8)			0.12					0.39		
Azulene	(10,12)	0.36	0.05	0.48	0.50	0.52	0.09	0.11	0.74	0.85	0.85
Nitrobenzene	(6,14)				0.58					1.08	
Benzonitrile	(6,13)					0.51					0.53
Average		0.22	0.19	0.21	0.49	0.61	0.11	0.13	0.26	0.53	0.77

(14,14)

Molecule	Active Space	CAS-PDFT Excitation Energy Error (eV)					CASPT2 Excitation Energy Error (eV)				
		S_1	S_2	S_3	S_4	S_5	S_1	S_2	S_3	S_4	S_5
Carbon Monoxide	(14,14)	0.64	0.64	0.20	0.40	0.40	0.18	0.18	0.30	0.26	0.26
Formaldehyde	(14,14)	0.07	0.29	0.36	0.81	0.45	0.03	0.30	0.08	1.10	1.83
Pyridine	(14,14)	0.00	0.22	0.01	0.40	0.28	0.04	0.07	0.21	0.25	0.27
Pyrrole	(14,14)	0.14	0.23	0.22	0.41	0.25	0.14	0.12	0.25	0.02	0.71
Thiophene	(14,14)	0.26	0.06	0.03	0.05	0.17	0.11	0.34	0.20	0.30	0.41
Diazirine	(14,14)	0.08	0.05	0.21	0.29		0.00	0.01	0.03	0.21	
Ketene	(14,14)	0.25	0.22	0.14	0.77		0.10	0.07	0.02	1.97	
Diazomethane	(14,14)	0.21	0.26	0.55			0.01	0.44	0.49		
Hydrogen Cyaphide	(14,14)	0.09	0.12	0.12			0.12	0.01	0.02		
Imidazole	(14,14)	0.23	0.28	0.07			0.13	0.31	0.53		
Nitrosyl Hydride	(14,14)	0.07	0.05	0.17			0.03	0.18	0.23		
Nitrosomethane	(14,14)	0.04	0.05	0.11			0.11	0.11	0.21		
Cyclopropene	(14,14)	0.04	0.09				0.06	0.18			
Hydrogen Sulfide	(14,14)	0.18	0.08				0.15	0.17			
Propynal	(14,14)	0.18	0.02				0.13	0.19			
Acetaldehyde	(14,14)	0.56					0.10				
Difluorocarbene	(14,14)	0.97					0.11				
Formamide	(14,14)	0.42					0.27				
Formyl Fluoride	(14,14)	0.05					0.06				
Hydrogen Chloride	(14,14)	0.15	0.14				0.03	0.03			
Methanimine	(14,14)	0.25					0.09				
Aniline	(14,14)			0.19					0.45		
Azulene	(14,14)	0.25	0.45	0.54	0.56	0.57	0.05	0.03	0.71	0.83	0.82
Nitrobenzene	(14,14)				0.10					0.04	
Benzonitrile	(14,14)					0.30					0.24
Average		0.23	0.19	0.21	0.42	0.35	0.09	0.16	0.27	0.55	0.65

Excitation Energy Errors of Protocols with Respect to Reduced Active Space Sizes

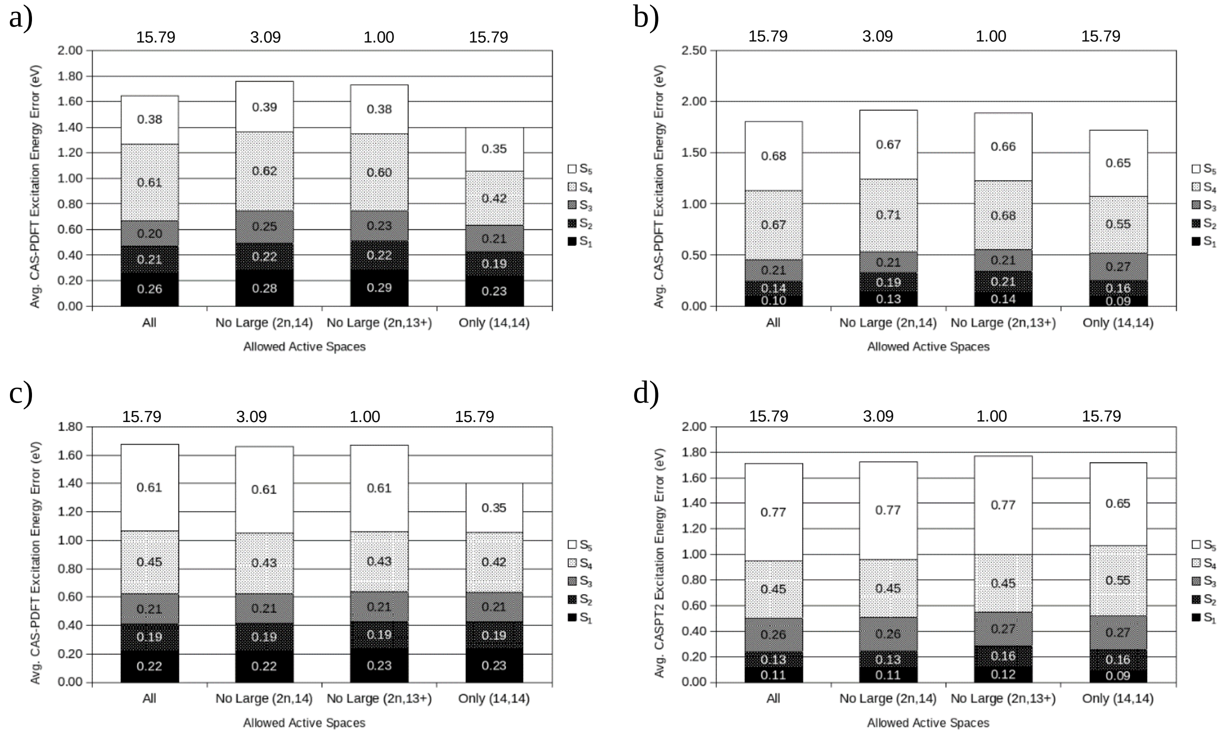

Figure S111: Average (a) CAS-PDFT and (b) CASPT2 excitation energy errors of active spaces chosen by GDM-AS with experimental reference dipole moments, and average (c) CAS-PDFT and (d) CASPT2 excitation energy errors of active spaces chosen by EDM-AS with TD-M06-2X reference dipole moments. From left to right: all active spaces in PASS are allowed; (10,14), (12,14) and (14,14) are removed; (10,13), (12,13) and (14,13) are additionally removed; only (14,14) is used. Numbers above the bar graphs represent the average run time needed to perform the scans, relative to the minimum.

Comparison of Intuitive Active Space with Specific Orbitals vs. with "Automatic" Active Spaces

Table S11: CAS-PDFT excitation energy errors, in eV, for two calculations on the methanimine molecule with the (12,11) active space. The "Intuitive" calculation starts from an active space with full valence orbitals and symmetry restrictions according to the molecular point group, while the "DFT Starting" calculation uses the orbital ordering from DFT and only restricts the size of the active space, without symmetry restrictions.

Choice	Active Space	S_1 Exc. Energy Error (eV)
Intuitive	(12,11)	1.44
DFT Starting	(12,11)	0.12

To obtain the data in Table S11, we have assumed that we are dealing with a molecule with unknown symmetry for each state. One may be able to find excitation energies with smaller errors if the symmetry of each state is known.

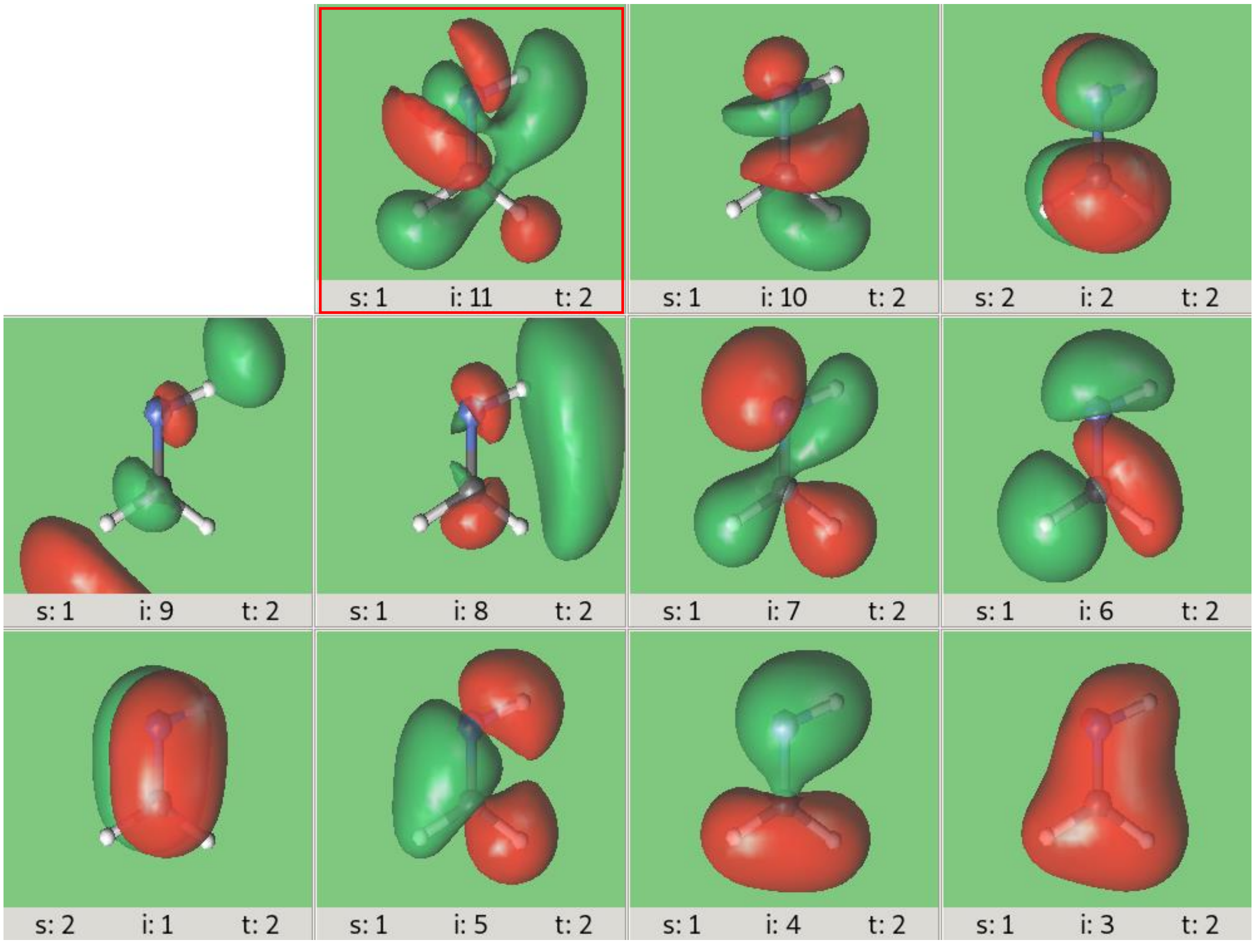

Figure S112: SA6-CASSCF orbitals in the intuitive (12,11) active space of methanimine. There is one more valence orbital (boxed in red) and one fewer Rydberg-like orbital than in the automatic (12,11) active space.

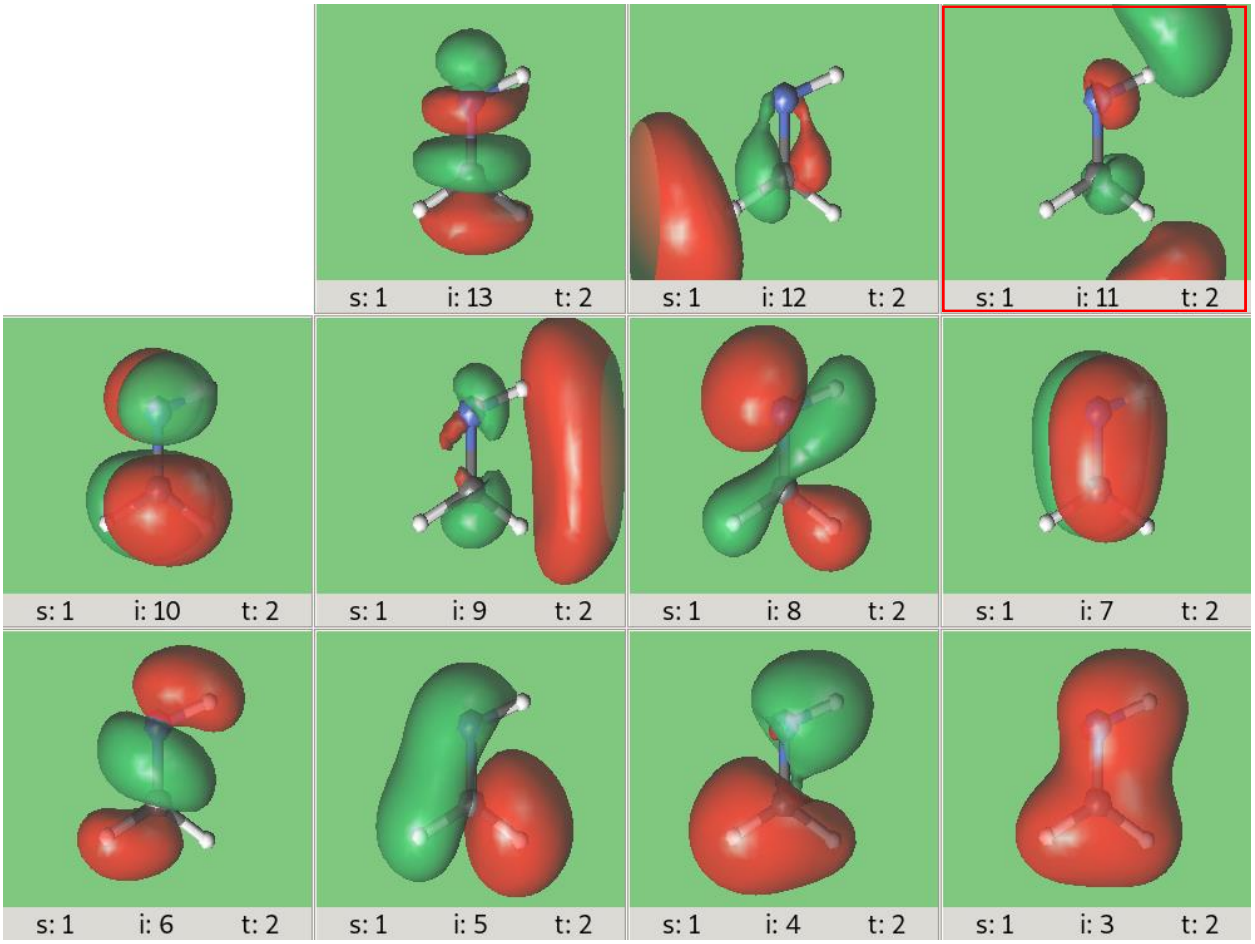

Figure S113: SA6-CASSCF orbitals in the automatic (12,11) active space of methanimine. There is one more Rydberg-like orbital (boxed in red) and one fewer valence orbital than in the intuitive (12,11) active space.

Hulburt-Hirschfelder Curve of CO Bond Dissociation with GDM-AS Run at Every Point

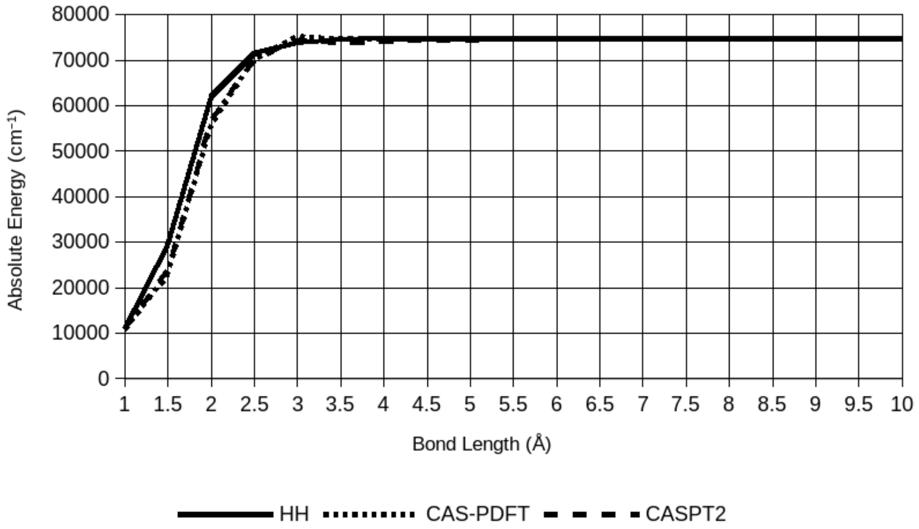

Figure S114: Potential energy curves of CO bond dissociation as calculated by the active spaces chosen by GDM-AS with CAS-PDFT and CASPT2, with the active space chosen at every point in the scan, as compared to that calculated by the Hulburt-Hirschfelder equation. The curves from CAS-PDFT and CASPT2 have been scaled and shifted so that they can be compared directly to the Hulburt-Hirschfelder curve.

Performance of GDM-AS on Transition Metal Oxides

Table S12: Active spaces chosen by GDM-AS for a set of transition metal oxide complexes provided by Wagner and Mitas,[4] and the absolute energies (in Hartrees) of the complex in eight different multiplicities, as calculated with the active space chosen by GDM-AS. Active spaces used for molecules with even multiplicities are those in PASS with one electron removed. Energies are calculated at all of the following levels of theory: a) CASSCF, b) CASPT2, and c) CAS-PDFT. The energy corresponding to the correct ground-state multiplicity for each complex is emphasized with bold numbers.

a)

Complex	Active Space	CASSCF Energy for Each Multiplicity (Eh)							
		Singlet	Doublet	Triplet	Quartet	Quintet	Sextet	Septet	Octet
ScO	(5,11)		***-838.37***		-838.23		-837.96		
TiO	(6,12)	-927.80		***-927.82***		-927.66		-927.41	

VO	(5,14)		-1,023.10		***-1,023.13***		-1,023.02		
CrO	(6,10)	-1,124.51		-1,124.55		***-1,124.58***		-1,124.54	
MnO	(11,14)		-1,232.30		-1,232.34		***-1,232.39***		-1,232.34

b)

Complex	Active Space	CASPT2 Energy for Each Multiplicity (Eh)							
		Singlet	Doublet	Triplet	Quartet	Quintet	Sextet	Septet	Octet
ScO	(5,11)		***-838.80***		-838.65		-838.36		
TiO	(6,12)	-928.27		***-928.29***		-928.12		-927.85	
VO	(5,14)		-1,023.67		***-1,023.70***		-1,023.54		
CrO	(6,10)	-1,125.10		-1,125.15		***-1,125.19***		-1,125.10	
MnO	(11,14)		-1,232.84		-1,232.89		***-1,232.95***		-1,232.88

c)

Complex	Active Space	CAS-PDFT Energy for Each Multiplicity (Eh)							
		Singlet	Doublet	Triplet	Quartet	Quintet	Sextet	Septet	Octet
ScO	(5,11)		***-839.26***		-839.11		-838.80		
TiO	(6,12)	-928.75		***-928.77***		-928.61		-928.33	
VO	(5,14)		-1,024.18		***-1,024.21***		-1,024.04		
CrO	(6,10)	-1,125.64		-1,125.67		***-1,125.71***		-1,125.61	
MnO	(11,14)		-1,233.38		-1,233.40		***-1,233.47***		-1,233.37

References

(1) Véril, M.; Scemama, A.; Caffarel, M.; Lipparini, F.; Boggio-Pasqua, M.; Jacquemin, D.; Loos, P.-F. QUESTDB: A Database of Highly Accurate Excitation Energies for the Electronic Structure Community. *WIREs Comput. Mol. Sci.* **2021**, *11* (5), e1517.

(2) Loos, P.-F.; Comin, M.; Blase, X.; Jacquemin, D. Reference Energies for Intramolecular Charge-Transfer Excitations. *J. Chem. Theory Comput.* **2021**, *17* (6), 3666–3686.

(3) Loos, P.-F.; Jacquemin, D. A Mountaineering Strategy to Excited States: Highly Accurate Energies and Benchmarks for Bicyclic Systems. *J. Phys. Chem. A* **2021**, *125* (47), 10174–10188.
(4) Wagner, L. K.; Mitas, L. Energetics and Dipole Moment of Transition Metal Monoxides by Quantum Monte Carlo. *J. Chem. Phys.* **2007**, *126* (3), 034105.
